# Supplementary material for: Characterization of elusive rhamnosyl dioxanium ions and their application in complex oligosaccharide synthesis
Source: Nat Commun. 2024 Mar 13;15:2257. doi: 10.1038/s41467-024-46522-2 (PMC10937939; doi:10.1038/s41467-024-46522-2)
Supplement: Supplementary file 1 — Supplementary Information [file 41467_2024_46522_MOESM1_ESM.pdf]

Supplementary information

Characterization of Elusive Rhamnosyl Dioxanium Ions and Their Utilization in Oligosaccharide Synthesis

Peter H. Moons#[a], Floor ter Braak#[a], Frank F.J. de Kleijne#[a], Bart Bijleveld[a], Sybren J.R. Corver[a], Kas J. Houthuijs[b], Hero R. Almizori[a], Jonathan Martens[b], Jos Oomens[b], Paul B. White[a], Thomas J. Boltje*[a]

[a] Department of Synthetic Organic Chemistry, Institute for Molecules and Materials, Radboud University, Heyendaalseweg 135, 6525 AJ, Nijmegen, The Netherlands

[b] FELIX Laboratory, Institute for Molecules and Materials, Radboud University, Toernooiveld 7, 6525 ED, Nijmegen, The Netherlands

#equal contribution

Table of Contents

Supplementary Methods ***3***

Experimental IRIS ***3***

Ion spectroscopy in a modified ion trap mass spectrometer 3

Generation of computational IR spectra 3

MS spectra 3

Experimental NMR ***4***

Instrumentation 4

Determining stereochemistry and selectivity with ^{13}C-coupled and quantitative HSQC 4

CEST & EXSY experiment settings 7

Chemical Exchange Saturation Transfer NMR (CEST NMR) spectroscopy 7

CEST Profile Acquisition 8

CEST Kinetics Acquisition 8

Selective ^{19}F Exchange NMR (EXSY NMR) spectroscopy 8

Determining exchange rates with EXSY and CEST 8

Initial rate approximation 8

Chemical Exchange Saturation Transfer NMR (CEST NMR) kinetic experiments 10

Standard deviations 12

Sample preparation VT NMR 12

VT-NMR studies 13

Synthesis materials & methods ***24***

General synthetic conditions 24

Supplementary NMR data ***41***

Synthesis NMR spectra ***45***

Supplementary References ***74***

Supplementary Methods

Experimental IRIS

Ion spectroscopy in a modified ion trap mass spectrometer

IRIS experiments were performed in a quadrupole ion trap mass spectrometer (Bruker, AmaZon Speed ETD) that has been modified to provide optical access to the stored ions for spectroscopy experiments. Details of these modifications and operation of the experiment are described elsewhere.[1] Ammonium adducts of compounds **1** and **3** were generated by electrospray ionization from solutions of 10^{-6} M (in 1:1 Acetonitrile/water) containing 2% ammonium acetate and introduced at 2 µl min^{-1}. In order to generate the relevant oxonium products, mass-selected precursor ions of interest were collisionally activated for 40ms with an amplitude parameter of 0.2-0.4V. The oxonium fragments are then mass isolated in an additional MS/MS stage and ultimately irradiated by the tunable FELIX mid-infrared laser beam.[2] The FEL was tuned to provide 10 µs optical pulses at 10 Hz having 30–60 mJ pulse energy over the entire tuning range (bandwidth ~0.4% of the centre frequency). Hereby the pulse energy used for experiment was attenuated accordingly to avoid saturation of the signal. Upon absorption of a sufficient number of photons unimolecular dissociation is induced and frequency-dependent fragmentation is observed by monitoring fragment ion intensities with the mass spectrometer. An infrared vibrational spectrum can be generated by relating the precursor ion intensity to the summed fragment intensities in the observed mass spectra (yield=ΣI(fragment ions)/ΣI(parent+fragment ions)) for each frequency position (3 cm^{-1} step size). The yield is obtained from several averaged mass spectra and is linearly corrected for laser power. The IR frequency is calibrated using a grating spectrometer.

Generation of computational IR spectra

Vibrational spectra of candidate structures were generated using a previously reported workflow.[3] representations for the oxocarbenium and C-1,C-3 dioxanium ions served as the input for the cheminformatics toolbox RDKit.[4] A series of 500 random conformations were generated for each ion using the distance geometry algorithm, which were subsequently minimized using the MMFF94 classic forcefield. Based on the root-mean-squared distance between them the 40 most distinctive geometries were selected which served as an input for semi-empirical PM6 minimization and vibrational analysis with Gaussian16 Rev. C.01.[5] After being filtered for duplicates, the remaining structures were minimized at the B3LYP/6-31++G(d,p) level, followed by vibrational analysis and a single point energy calculation at the MP2/6-31++G(d,p) level. Relative energies are based on the combined MP2 electronic energy and the Gibbs free energy (T=298.15K) from the B3LYP vibrational analysis. The harmonic vibrational line spectra were frequency scaled using a scaling factor of 0.975 and broadened using a Gaussian function with a full-width at half-maximum of 25 cm^{-1} to resemble experimental peak widths.

MS spectra

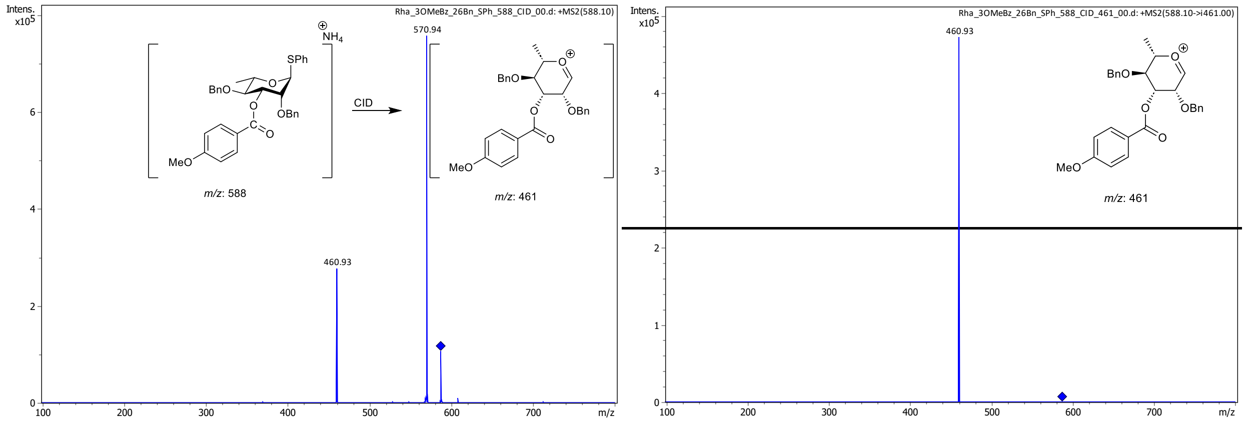

Supplementary Figure 1. MS-spectra of CID of compound **1** (left) and isolation of fragment m/z = 461 (right).

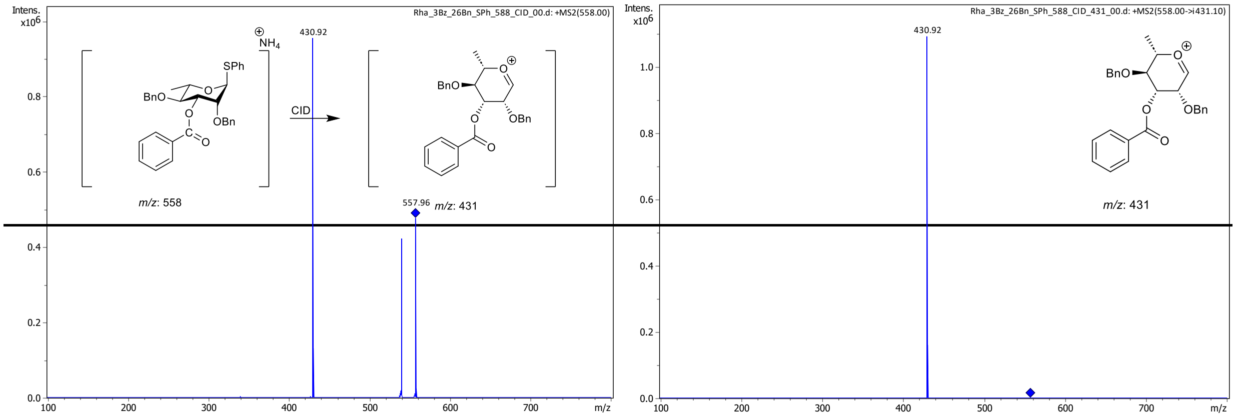

Supplementary Figure 2. MS-spectra of CID of compound **3** (left) and isolation of fragment m/z = 431 (right).

Experimental NMR

Instrumentation

Variable temperature NMR (VT NMR) experiments were conducted on a Bruker 300 MHz Avance III HD nanobay equipped with a BBFO probe and on the JEOL 500 ECZ-R spectrometer equipped with a ROYAL-HFX or ROYAL probe. Low temperature VT operations were achieved with the aid of LN2 evaporator to supply the cold gas, which the probe heated to the desired temperature. The temperature for VT experiments was calibrated against a pure MeOH standard to accurately determine the probe temperature.

Determining stereochemistry and selectivity with ^{13}C-coupled and quantitative HSQC

^{13}C-coupled HSQC spectra were obtained by turning off the ^{13}C-decoupler function prior to measurements. Equatorial-oriented hydrogen have been reported to have a higher $^{1}J_{C,H}$-coupling than axial-oriented hydrogen.[6,7] Quantitative HSQC measurements were carried out according to pulse sequences and obtained from literature.[8,9]

Supplementary Table 1: glycosylation behaviour of rhamnosyl donors **1, 2** and **3**. **5-Azidopentanol was used as glycosyl acceptor.**

Donor	Promoter	*T*	Yield	α:β ratio	$^{1}J_{C,H}$-coupling (Hz)	H-1 (ppm)	C-1 (ppm)
**1**	NIS, TfOH	-78°C - 0°C	94%	37:1	171 (α)<br>152 (β)	4.77 (α)<br>4.55 (β)	97.99 (α)<br>101.14 (β)
**1**	Ph_2SO, Tf_2O, TTBP	-78°C	85%	α only	171 (α)	4.77 (α)	97.98 (α)
**2**	NIS, TfOH	-78°C - 0°C	68%	1.8:1	171 (α)<br>152 (β)	4:73 (α)<br>4.33 (β)	98.00 (α)<br>101.57 (β)
**2**	Ph_2SO, Tf_2O, TTBP	-78°C	81%	1.4:1	171 (α)<br>152 (β)	4.73 (α)<br>4.33 (β)	97.99 (α)<br>101.56 (β)
3	NIS, TfOH	-78°C - 0°C	84%	21:1	170 (α)<br>155 (β)	4.77 (α)<br>4.56 (β)	98.03 (α)<br>101.27 (β)
3	Ph_2SO, Tf_2O, TTBP	-78°C	92%	α only	170 (α)	4.77 (α)	98.02 (α)

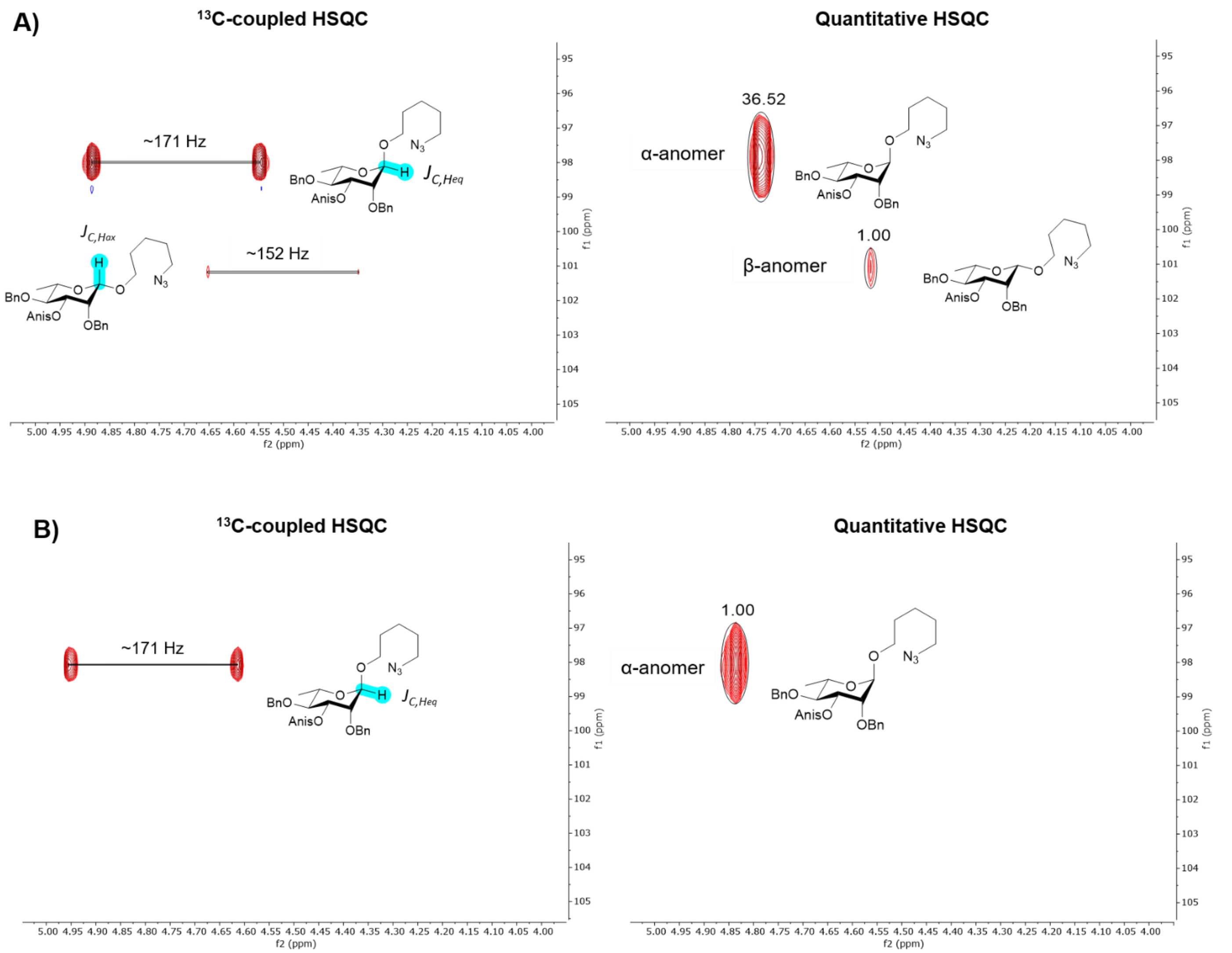

Supplementary Figure 3A,B: Determining stereochemistry of glycosylation products by measuring $^{1}J_{C,H}$-couplings (left)[6,7] and selectivity with quantitative HSQC (right)[8,9]. α/β-selectivity was determined using quantitative HSQC prior to purification with silica-flash column chromatography. (A) **1**, NIS, TfOH; (B) **1**, Ph_2SO, Tf_2O, TTBP.

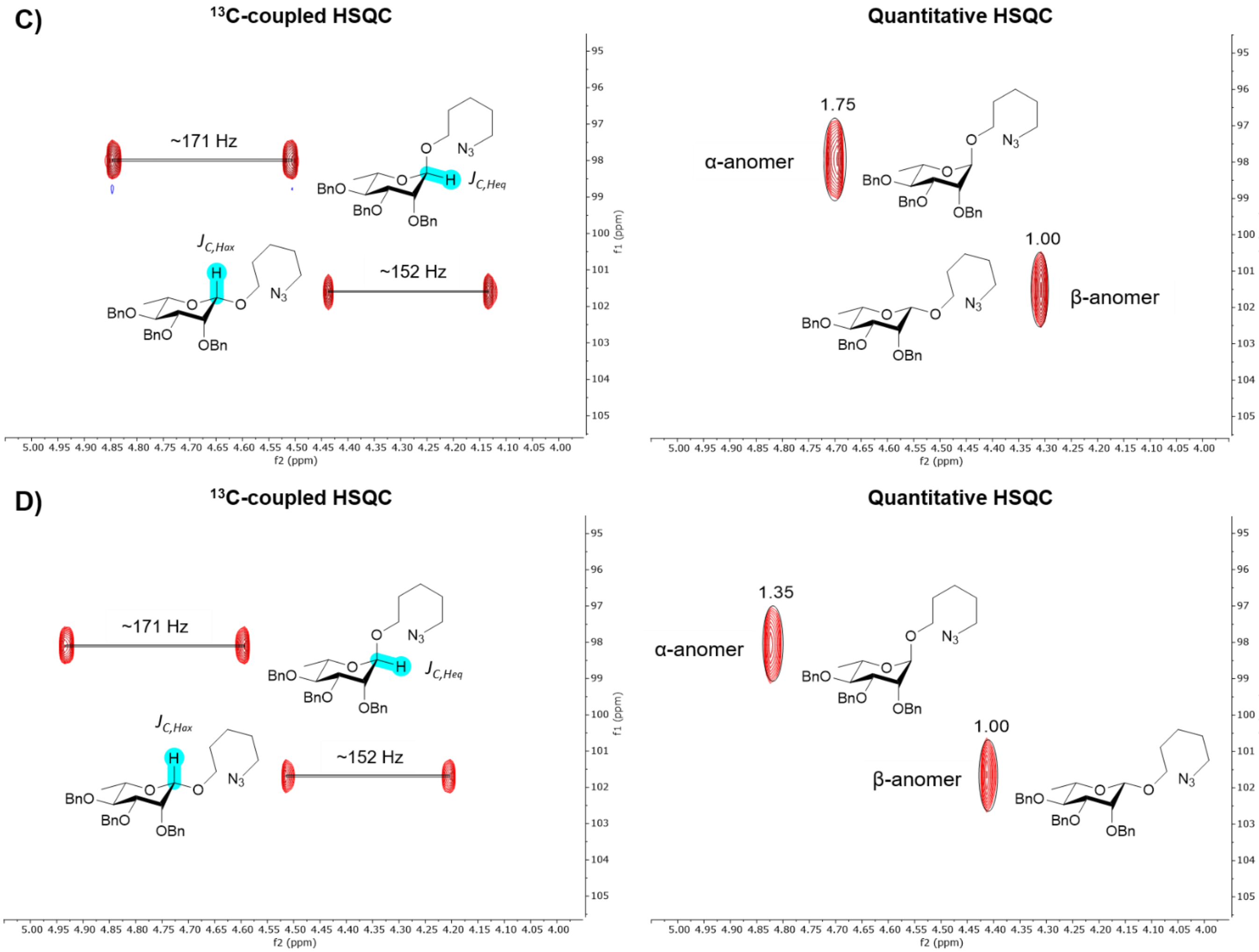

Supplementary Figure 3C,D: Determining stereochemistry of glycosylation products by measuring $^{1}J_{C,H}$-couplings (left)[6,7] and selectivity with quantitative HSQC (right)[8,9]. α/β-selectivity was determined using quantitative HSQC prior to purification with silica-flash column chromatography. (C) **2**, NIS, TfOH; (D) **2**, Ph_2SO, Tf_2O, TTBP.

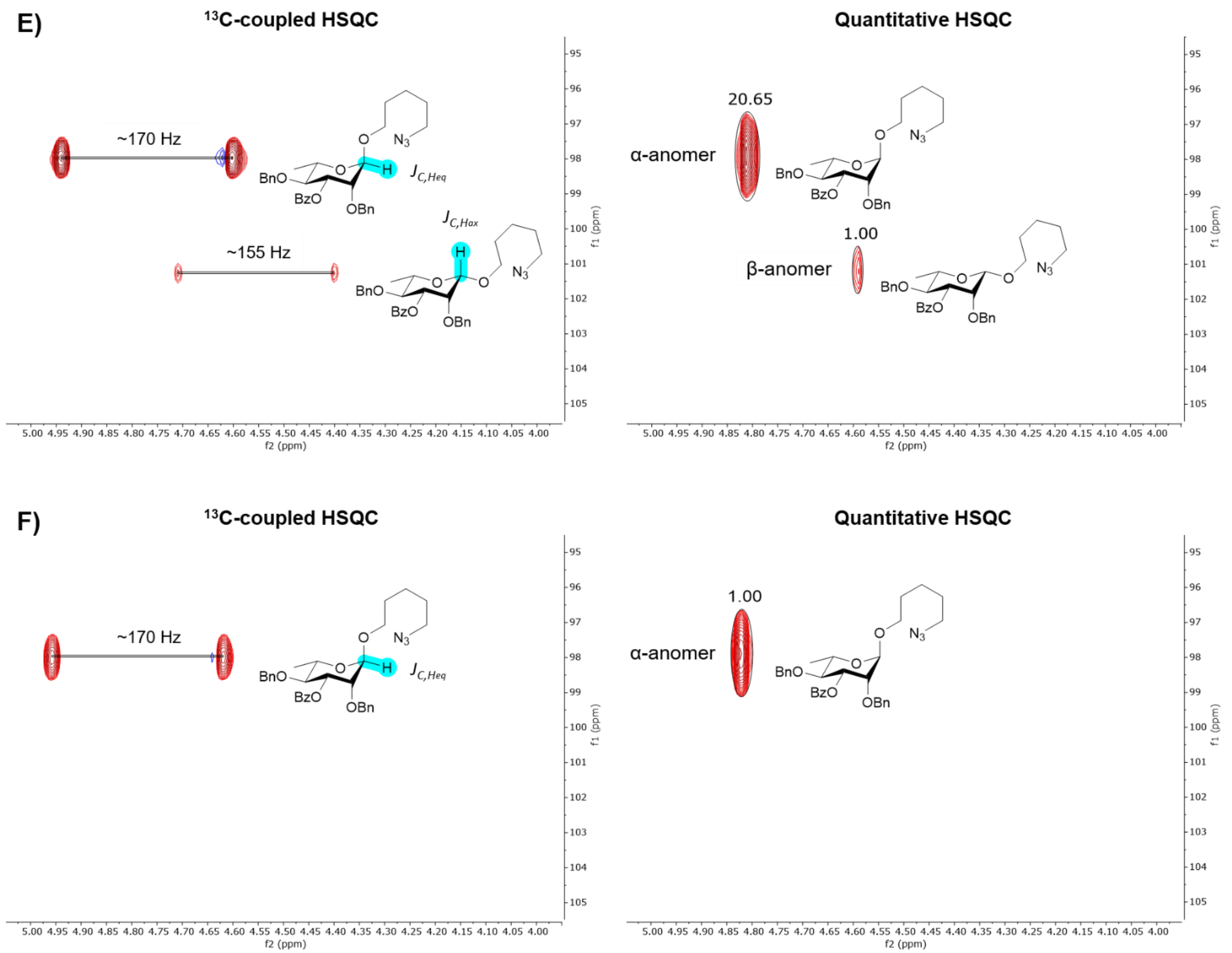

Supplementary Figure 3E,F: Determining stereochemistry of glycosylation products by measuring $^{1}J_{C,H}$-couplings (left)[6,7] and selectivity with quantitative HSQC (right)[8,9]. α/β-selectivity was determined using quantitative HSQC prior to purification with silica-flash column chromatography. (E) **3**, NIS, TfOH; (F) **3**, Ph_2SO, Tf_2O, TTBP.

CEST & EXSY experiment settings

Chemical Exchange Saturation Transfer NMR (CEST NMR) spectroscopy

CEST NMR was utilized in ^{13}C, NMR spectroscopy. CEST NMR spectra were recorded by incrementing the saturation over a domain of interest, for ^{13}C CEST was typically performed in a window between 190 and 160 ppm. Before each experiment, the 90-degree pulse was calibrated. A saturation field strength was chosen with respect to experimental duration, resolution, and signal intensity (eg. high resolution (small saturation field strengths) leads to weak signals and long experimental times, in contrast, low resolution (large saturation field strength) leads to stronger signals, faster experiments but low resolution). Typically, saturation field strengths were chosen to be 80 Hz. The saturation was achieved either by CW saturation (Bruker) or by pulsed saturation using laminar pulses (JEOL). Finally, typical saturation times were set to 2-3 seconds; the relaxation delay was set one second longer then the saturation time (3-4 seconds); number of scans were typically 2-4 per frequency; Two dummy scans (Bruker) or 2 (JEOL) were executed.

CEST Profile Acquisition

CEST profiles were constructed using 1D ^{13}C spectra with saturation at variable positions. A spectrum with no saturation or with off-resonance saturation to both the major and minor observable signal is required as reference named $M_z(0)$ (typically a 1D spectrum with saturation at δ_C = 190 ppm; δ_H = 9.0 ppm; or δ_F = -72 ppm). The peak intensity for the α-triflate (^{13}C and ^{1}H CEST) and ^{-}OTf (^{19}F) resonances were determined for all individual spectra after phasing and applying baseline correction. The peak intensity of every spectrum was divided by the peak intensity of the unaltered spectrum ($M_z(0)$) to obtain the relative peak intensity of the main observable species as function of saturation frequency. Plotting the relative intensity of the α-triflate as function of the saturation frequency gives the CEST spectrum.

CEST Kinetics Acquisition

Dioxanium ion formation rates ($R_{\alpha \rightarrow d,\ CEST}$) were determined by using saturation-transfer. The saturation frequency offset was set to be on-resonance with the minor exchangeable species (e.g. the dioxanium C=O 13C resonance) as discovered by first obtaining a CEST profile. The saturation offset frequency was held constant while the saturation duration was varied. Deriving the exchange rate required a spectrum having no saturation ($M_z(0)$), a spectrum where the exchange and relaxation rates are in equilibrium ($M_z(inf)$), and a number of spectra where a decay of the main observable is visible as function of saturation time ($M_z(\tau)$). Typically, the saturation times were set to 0 seconds, 0.1 seconds incrementing to 3 seconds, and 4 seconds. The resulting 1D ^{13}C spectra were phased and baseline correction was applied. The maximum peak intensity was determined for all individual 1D ^{13}C NMR spectra and was plotted against the saturation time to yield the kinetic build-up curves. The crude 1D ^{13}C peak intensities were converted as described below to yield the exchange rate.

Selective ^{19}F Exchange NMR (EXSY NMR) spectroscopy

EXSY NMR was utilized in ^{19}F NMR spectroscopy. Before each experiment, the 90-degree pulse was calibrated and then the selective excitation offset was set to the resonance of interest. The selection pulse typically spanned either 0.1 or 0.2 ppm and was on-resonance with the glycosyl α-triflate ($\delta_F \approx$ -75.9 ppm). The power levels of the excitation pulse were calculated against the actual 90-degree pulse and the selection window. Relaxation delays were typically set to 5xT1 of the triflates, and the number of scans were set to 8 with 2 dummy scans. The longest mix times were determined empirically so that they fit within the initial rate approximation (~10% conversion). For experiments where the shortest mix time was > 80 ms, a pulse sequence that contains a Z-gradient element to crush zero-quantum magnetization and clean up artifacts was used (Bruker: selnogp, JEOL: noesy_1d). However, frequently the shortest mix time was << 80 ms, therefore the versions of the above experiments without the Z-gradient element were utilized.

Determining exchange rates with EXSY and CEST

Initial rate approximation

As outlined in the main text, the α-triflate dissociates to form triflate anion in two possible mechanisms (Eq. S1 and S2). The rate laws of the corresponding reactions are described in equation S3 and S4 for the intramolecular glycosyl stabilization and intermolecular glycosyl stabilization respectively. Both processes can proceed simultaneously, hence the overall rate law could be a combination of both (Eq. S5).

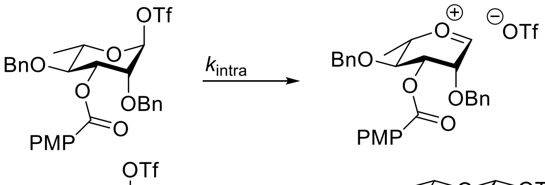

Eq. S1

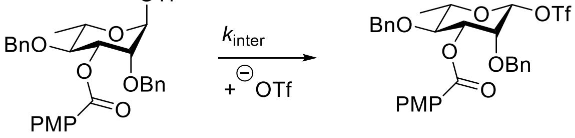

Eq. S2

$$\frac{d[OTf]}{dt} = -\frac{d[\alpha]}{dt} = R_{a\to OTf} = k_{inter}[\alpha]$$ Eq. S3

$$\frac{d[OTf]}{dt} = -\frac{d[\alpha]}{dt} = R_{a\to OTf} = k_{intra}[\alpha][^{-}OTf]$$ Eq. S4

$$\frac{d[OTf]}{dt} = -\frac{d[\alpha]}{dt} = R_{a\to OTf} = k_{inter}[\alpha] + k_{intra}[\alpha][^{-}OTf]$$ Eq. S5

Selective 1D ^{19}F EXSY NMR is a suitable method to study the exchange. By applying a selective excitation pulse on the α-triflate resonance, formation of triflate anion can be measured despite the high population triflate already present in the reaction mixture. This is possible for two main reasons: 1) the resonances of both the α-triflate and triflate anion are sufficiently separated to selectively excite the α-triflate in ^{19}F NMR, and, 2) only excited-state nuclei are detected in EXSY NMR. Varying the delay (mix time, τ_m) between the excitation of the α-triflate resonance and the spectrum acquisition allows to measure different degrees of conversion for the α-triflate into the triflate anion resonance. Eventually, by applying an 'infinitely long' mix time, the equilibrium ratio of α-triflate and triflate anion is obtained Figure S4).

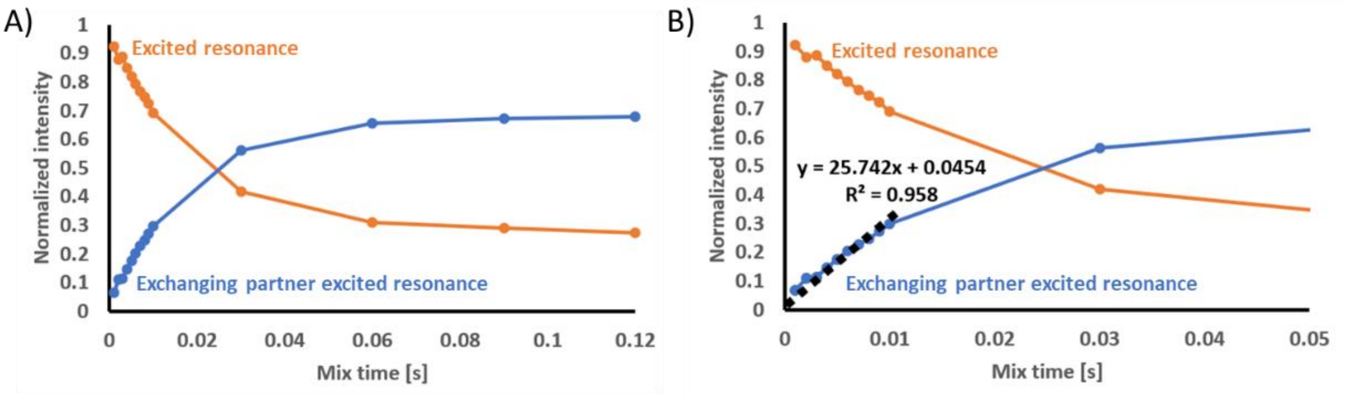

Supplementary Figure 4: The normalized extent of magnetization transfer from the excited CF_3-group of the α-triflate to the unbound triflate is plotted against the mix time. (A) Correlation of EXSY mix time to intensity selected/excited resonance and its exchanging partner that is forming; (B) Displaying the linear initial rate approximation.

The initial triflate formation is linear and kinetics could be described according to the initial rate approximation. Over the initial linear interval, the reaction rate is the α-triflate consumption and triflate anion formation (Eq. S6 and S7). Herein, $[\alpha]_t$ = concentration excited state α-triflate a set mix time after applying the excitation pulse on the α-triflate resonance; $[\alpha]_0$ = concentration excited state α-triflate directly after applying a selective excitation pulse on the α-triflate resonance; $[^{-}OTf]_t$ = concentration excited state $^{-}$OTf a set mix time after applying the excitation pulse on the α-triflate resonance; $[^{-}OTf]_0$ = concentration excited state $^{-}$OTf directly after applying a selective excitation pulse on the α-triflate resonance (hence, $[^{-}OTf]_0 = 0$). Substituting equation S5 into equation S6 gives the concentration excited state α-triflate in terms of concentration and mix time (Eq. S8).

$$\frac{[\alpha]_t - [\alpha]_0}{\tau_m} = -R_{\alpha\to OTf}$$ Eq. S6

$$\frac{[^{-}OTf]_t - [^{-}OTf]_0}{\tau_m} = R_{\alpha\to OTf}$$ Eq. S7

$$[\alpha]_t = [\alpha]_0 - (k_{inter}[\alpha] + k_{intra}[\alpha][^{-}OTf]) \times \tau_m$$ Eq. S8

At the very start of the reaction, the concentration α-triflate deviates only marginally compared to the starting concentration ($[\alpha]_0$). In accordance to the initial rate approximation, equation S8 becomes equation S9. Additionally, within the initial rate approximation, only an α-triflate conversion of about 5-15% is recorded. Therefore, the concentration excited state triflate ($[^{-}OTf]$)

is sufficiently small (especially compared to the bulk concentration non-excited triflate anion ([$^-$OTf])) such that the backwards reaction can be neglected. Subsequently, dividing the equation by $[\alpha]_0$ simplifies the equation to S10.

$$[\alpha]_t = [\alpha]_0 - (k_{inter}[\alpha]_0 + k_{intra}[\alpha]_0[^-OTf]) \times \tau_m \qquad \text{Eq. S9}$$

$$\frac{[\alpha]_t}{[\alpha]_0} = 1 - (k_{inter} + k_{intra}[^-OTf]) \times \tau_m \qquad \text{Eq. S10}$$

Within NMR spectroscopy, the concentration is proportional (with a constant, c) related to the absolute integral ($\int I_x$) of the observed resonances (Eq. S11 and S12). Substituting equation S10 with S11 and S12 gives the absolute integral of the excited state α-triflate resonance as function of mix time (Eq. S13).

$$\int I_{\alpha,t} = c \times [\alpha]_t \qquad \text{Eq. S11}$$

$$\int I_{\alpha,0} = c \times [\alpha]_0 \qquad \text{Eq. S12}$$

$$\frac{\int I_{\alpha,t}}{\int I_{\alpha,0}} = 1 - (k_{inter} + k_{intra}[^-OTf]) \times \tau_m \qquad \text{Eq. S13}$$

One complication is that T_1 relaxation occurs during the mixing time, which will reduce the absolute integral or intensity of the selected and exchanged resonances over time and plotting S13 will result in a multiexponential decay process if $k \leq T_1^{-1}$. This can be easily taken into account if the T_1 is known for each in the absence of exchange. However, if the T_1s for the species are very similar and thus experience similar rates of relaxation, then an approximation can be made where the integral of the α-triflate at t=0 ($I_{\alpha,0}$) is the sum of the integrals of the α-triflate and triflate anion at a given mix time (Eq. S14). This then allows Equation S13 to be rewritten in an internally-consistent manner where the decay is normalized by the measurable peaks for each given mix time. The slope of the plot (Eq. S16) is directly related to the rate constants of both exchange processes (Eq. S1 and S2).

$$\int I_{\alpha,0} = \int I_{\alpha,t} + \int I_{OTf,t} \qquad \text{Eq. S14}$$

$$\frac{\int I_{\alpha,t}}{\int I_{\alpha,t} + \int I_{OTf,t}} = 1 - (k_{inter} + k_{intra}[^-OTf]) \times \tau_m \qquad \text{Eq. S15}$$

$$Slope = k_{inter} + k_{intra}[^-OTf] \qquad \text{Eq. S16}$$

The rates measured and determined by selective ^{19}F EXSY spectroscopy is, as described above, directly the slope of the normalised absolute integral of the α-triflate ($\int I_{\alpha,t}$) versus mixing time. Hence, equation S16 is in the main text referred to as $R_{\alpha \rightarrow OTf,\ EXSY}$ (Eq. S17).

$$R_{a \rightarrow OTf,EXSY} = Slope = k_{inter} + k_{intra}[^-OTf] \qquad \text{Eq. S17}$$

Chemical Exchange Saturation Transfer NMR (CEST NMR) kinetic experiments

Saturation transfer experiments to derive the exchange constant from the α-triflate to dioxanium ion were conducted as reported by Serianni *et al*.[10] The system is considered as in Figure S5, and their corresponding formation rates are described in eq. S18 and S19.

$$\frac{d[\alpha]}{dt} = R_{diox \rightarrow a} = k_{d \rightarrow \alpha}[diox][OTf] \qquad \text{Eq. S18}$$

$$\frac{d[diox]}{dt} = R_{a \rightarrow diox} = k_{\alpha \rightarrow d}[\alpha] \qquad \text{Eq. S19}$$

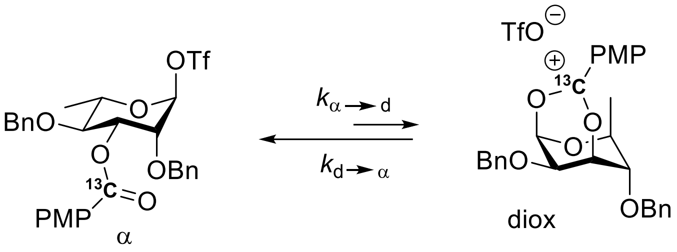

Supplementary Figure 5: Equilibrium considered for determining the reaction rate constant of dioxanium ion formation ($k_{\alpha\to d}$).

Equation S20 describes the modified Bloch equation that accounts for change in longitudinal magnetization (M_z) due to T_1 relaxation and chemical exchange as a function of time (t).

$$\frac{dM_z^{trif}(t)}{dt} = \frac{-\left(M_z^{trif}(t)-M_z^{trif}(0)\right)}{T_{1,trif}} - \frac{M_z^{trif}(t)}{\tau_{trif}} + \frac{M_z^{diox}(t)}{\tau_{diox}} \qquad \text{Eq. S20}$$

Herein: $T_{1,trif}$ is the spin-lattice relaxation time of the α-triflate resonance, τ_{trif} is the lifetime of the triflate, and τ_{diox} is the lifetime of the dioxanium ion. Due to saturation of the dioxanium resonance, the magnetization of the dioxanium (M_z^{diox}) becomes zero, and, as a result, M_z^{diox}/τ_{diox} also goes to zero. The resulting equation can then be integrated to yield equation S21.

$$M_z^{trif}(\tau) = M_z^{trif}(0) \times \left(\frac{\tau_{1,trif}}{\tau_{trif}} e^{\frac{-\tau}{\tau_{1,trif}}} + \frac{\tau_{1,trif}}{T_{1,trif}}\right) \qquad \text{Eq. S21}$$

Here, $M_z^{trif}(\tau)$ and $M_z^{trif}(0)$ are the intensities of the α-triflate resonance after applying a saturation pulse for the duration of τ at the dioxanium ion resonance ($M_z^{trif}(\tau)$), and the intensity of the α-triflate resonance after applying a saturation pulse for the duration of 0 seconds at the dioxanium ion resonance ($M_z^{trif}(0)$). Subtracting the peak intensity of the α-triflate with 'infinitely long' saturation at the dioxanium ion ($M_z^{trif}(\infty)$) from $M_z^{trif}(\tau)$ gives the net saturation effect on the peak decay. Typically, $M_z^{trif}(\infty)$ is determined by saturating the dioxanium ion for five times the relaxation time of the carbonyl. Plotting the natural logarithm of the net peak decay resulting from saturation ($\ln(M_z^{trif}(\tau) - M_z^{trif}(\infty))$) against the saturation time (τ) gives a linear correlation where the slope is related to $\tau_{1,trif}$ according to equation S22.

$$slope = \frac{-1}{\tau_{1,trif}} \qquad \text{Eq. S22}$$

The observed lifetime ($\frac{1}{\tau_{1,trif}}$, Equation S23) describes the relation between the α-triflate lifetime (τ_{trif}) and its T1. $T_{1,trif}$, can be determined from the acquired data according to equation S24.

$$\frac{1}{\tau_{1,trif}} = \frac{1}{\tau_{trif}} + \frac{1}{T_{1,trif}} \qquad \text{Eq. S23}$$

$$T_{1,trif} = \frac{M_z^{trif}(0)}{M_z^{trif}(\infty)} \times \frac{1}{\tau_{1,trif}} \qquad \text{Eq. S24}$$

Applying equations S22, S23, and S24 yields the lifetime of the α-triflate. As described by McConnell,[11] the lifetime is to be related to pseudo first-order rate constants. As a consequence, in the system as described by Figure S5, the lifetimes of the α-triflate and dioxanium ion are given by equations S25 and S26. Hence, the rate constant for the formation of dioxanium ion from the α-triflate ($k_{\alpha\to d}$) is directly obtained from τ_{trif} (Eq. 25).

$$\frac{1}{\tau_{trif}} = k_{\alpha\to d} \qquad \text{Eq. S25}$$

$$\frac{1}{\tau_{diox}} = k_{d\to\alpha}[^{-}OTf] \qquad \text{Eq. S26}$$

Finally, in the main article $R_{\alpha \rightarrow d, CEST}$ is used to describe equation S26. Hence, $R_{\alpha \rightarrow d, CEST}$ is described according to equation S27.

$$R_{\alpha \rightarrow d,CEST} = \frac{1}{\tau_{trif}} = k_{\alpha \rightarrow d} \qquad \text{Eq. S27}$$

Standard deviations

The rates determined by EXSY are directly the slope of the normalized α-triflate peak integral against mix time (Eq. S17). The corresponding graph is based on the measured data analysed with the least square regression method. Equations S28 is used to determine the error in the determined EXSY rate based on the measured input data.

$$s_x = \sqrt{\frac{\sum(X_i - \bar{X})^2}{n-1}} \qquad \text{Eq. S28}$$

Rates determined by CEST are based on a linear relationship between the natural logarithm of the net saturation effect versus saturation time. The slope represents ${\tau_{1trif}}^{-1}$ and the standard deviation is calculated using equation S28. The relative error of ${\tau_{1trif}}^{-1}$ is determined according to equation S29 and the resulting relative error is applied to the determined rate (Eq. S30).

$$Relative\ error\ slope = \frac{e_{Slope}}{Slope} \qquad \text{Eq. S29}$$

$$e_{R_{\alpha \rightarrow d,CEST}} = \frac{e_{Slope}}{Slope} \times R_{\alpha \rightarrow d,CEST} \qquad \text{Eq. S30}$$

Sample preparation VT NMR

Glycosyl thioether donor (1.0 eq, typically 15 mg) and Ph_2SO (1.1 eq) were weighted and dissolved in dried DCM-d_2 (500 μL). Two spherical molecular sieves (4 or 5 Å) were added to the NMR tube and the tube was transferred to an analytical scale where internal standard (either trimethyl(trifluoromethyl)silane, or trimethyl(4-trifluoromethylphenyl)silane) was added. A stock solution of Tf_2O was prepared in DCM-d_2 such that upon addition of stock solution (0.1 mL), the desired amount Tf_2O (1.5 eq) could be added. The access Tf_2O was added to assure full consumption of Ph_2SO. When the NMR sample and Tf_2O stock solution were ready, the NMR tube was cooled to -80 °C (dry ice/acetone bath) and to the cold tube was added the freshly prepared Tf_2O stock solution (50 μL). The solution generally becomes (light) yellow upon addition of Tf_2O, was shaken quickly (3x) and was carefully transferred to the NMR. In the probe the temperature was heated to -40ºC until full consumption of the initially formed species with a resonance around $\delta_H \approx 6.5$ ppm was observed (typically 1 – 2 hrs). The sample was then cooled to -80°C at which a battery of kinetic and characterization experiments were conducted.

A 1.0 M solution of tetrabutylammonium triflate (TBAT) was prepared in DCM-d_2. To the solution was added activated molecular sieves (4 Å) and the solution was stored under argon at -80°C. This solution was removed from the -80°C fridge 60 min before the NMR experiment.

NMR experiments at various concentrations triflate anion were executed as described above with respect to sample preparation. After activation at the desired temperature, the probe was set to -80°C. At this temperature, the sample displayed an exchange ($R_{\alpha \rightarrow OTf, EXSY}$) of about 1 s^{-1} to allow sufficient exchange at the lowest concentration and sufficient opportunity to increase as a consequence of the increased triflate concentration before falling out the window of EXSY NMR (see main text). After recording the triflate dissociation under standard conditions, the sample was removed from the probe, quickly stored in a dry ice/acetone bath (-80°C) and the TBAT solution was added (20 μL). The sample was quickly shaken to homogenize the solution (3x) and was carefully transferred to the probe. The sample was locked to DCM-d_2, tuned, and shimmed before performing NMR experiments. After finishing the EXSY and CEST experiments, the cycle was repeated for two more time (by adding 30 μL and 50 μL TBAT solution). In the data workup, the internal standard was used to accurately correct the concentration to volume and TBAT added.

VT-NMR studies

A) Activation scheme

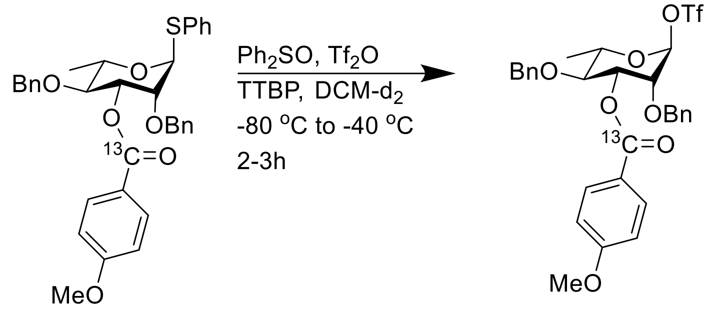

B) ^1H after full activation at -40 °C and cooled to -80 °C

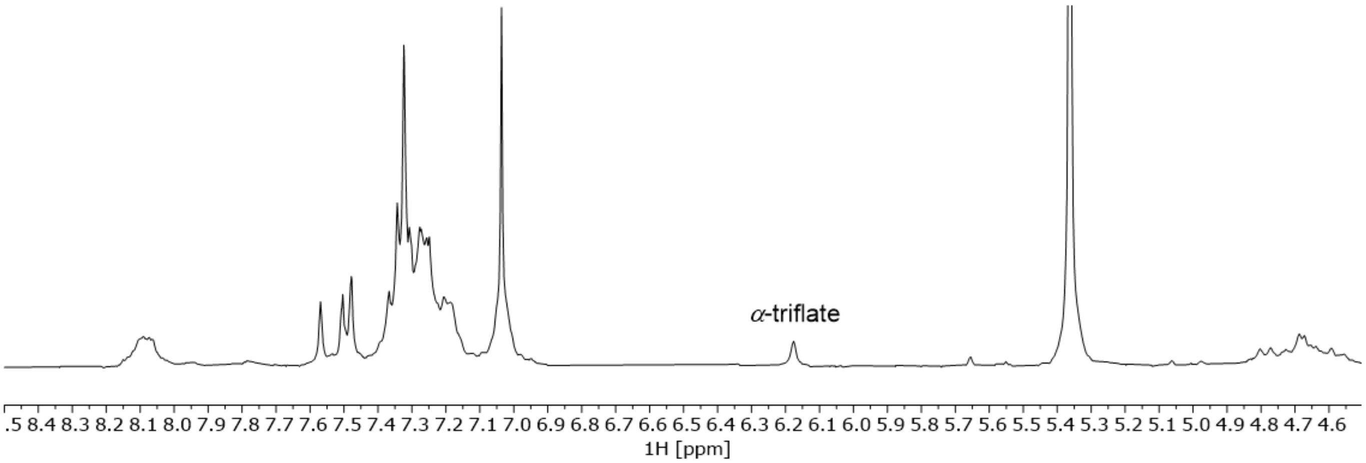

C) ^{19}F after full activation at -40 °C and cooled to -80 °C

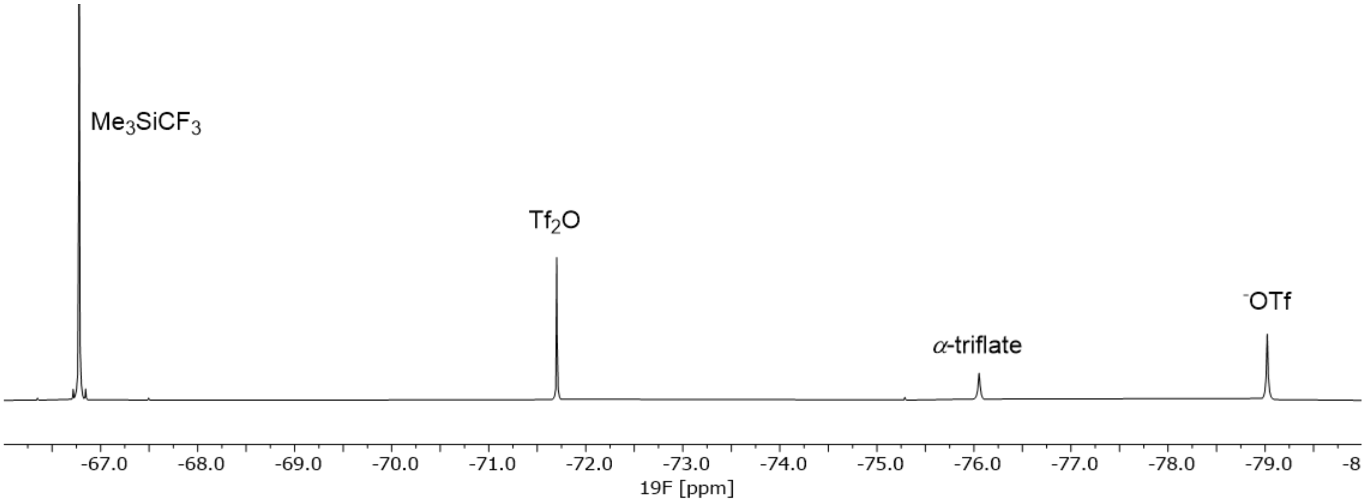

Supplementary Figure 6: VT NMR studies of the activation of rhamnosyl donor 1. The donor was activated at -80°C, heated to -40°C to obtain the α-triflate and then cooled to -80°C again. (A) Activation scheme of C-3 *p*-anisoyl-protected rhamnosyl donor **1**; (B) ^1H NMR spectrum recorded 3 hrs after activation in dry ice/acetonitrile bath (-50 to -40 °C). The spectrum was recorded at -80°C; (C) ^{19}F after full activation recorded at -80 °C.

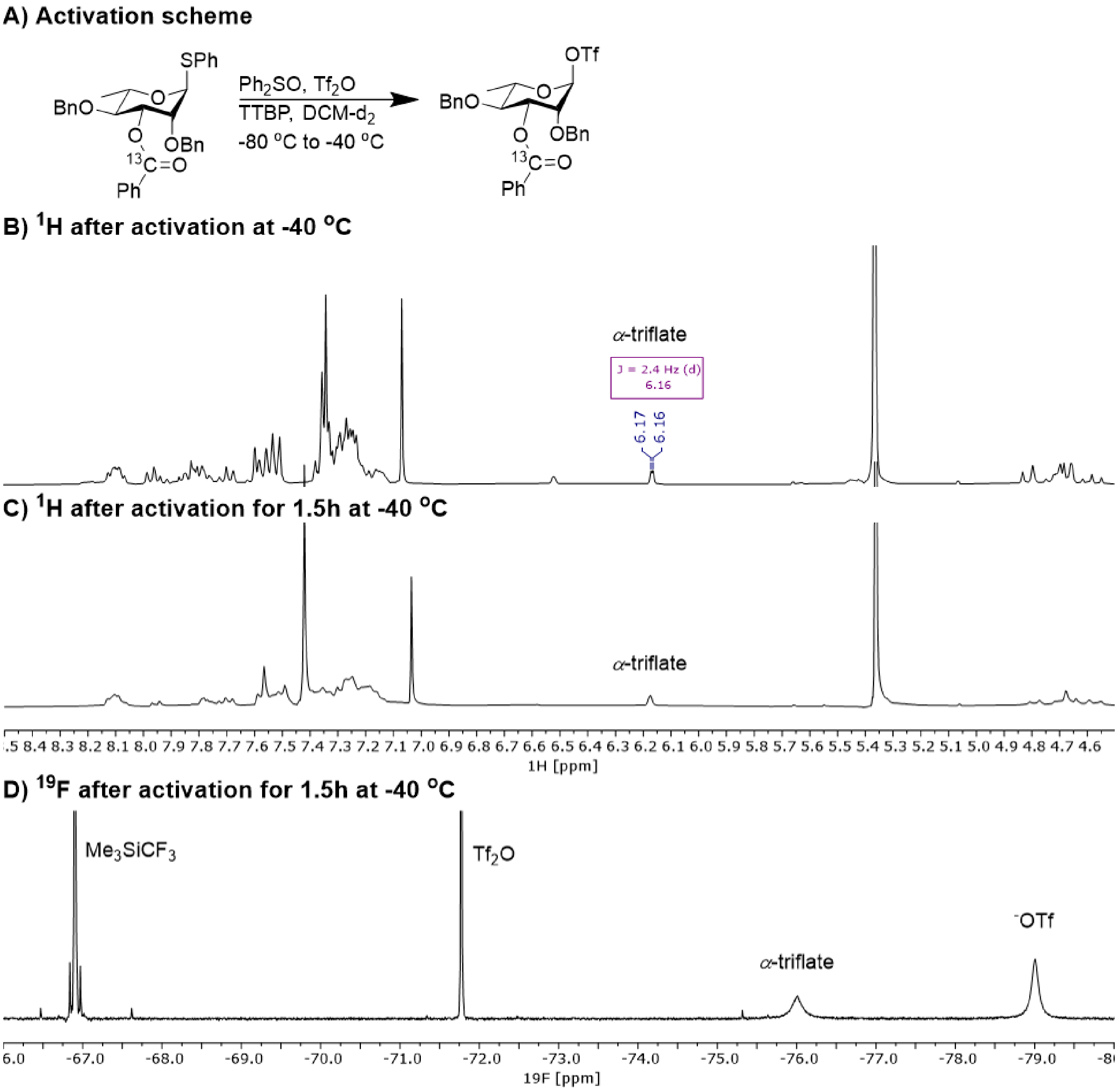

Supplementary Figure 7: VT NMR studies of the activation of rhamnosyl donor 3. The donor was activated at -80°C and heated to -40°C to obtain full conversion to the α-triflate after 1.5 hours. (A) Activation scheme of C-3 benzoyl-protected rhamnosyl donor **3**; (B) ^{1}H NMR spectrum after activation in a dry ice/acetonitrile bath (-50 to -40 °C); (C) ^{1}H NMR spectrum recorded 1.5 hrs after activation at -40°C; (D) ^{19}F-NMR spectrum recorded 1.5 hrs after activation at -40°C.

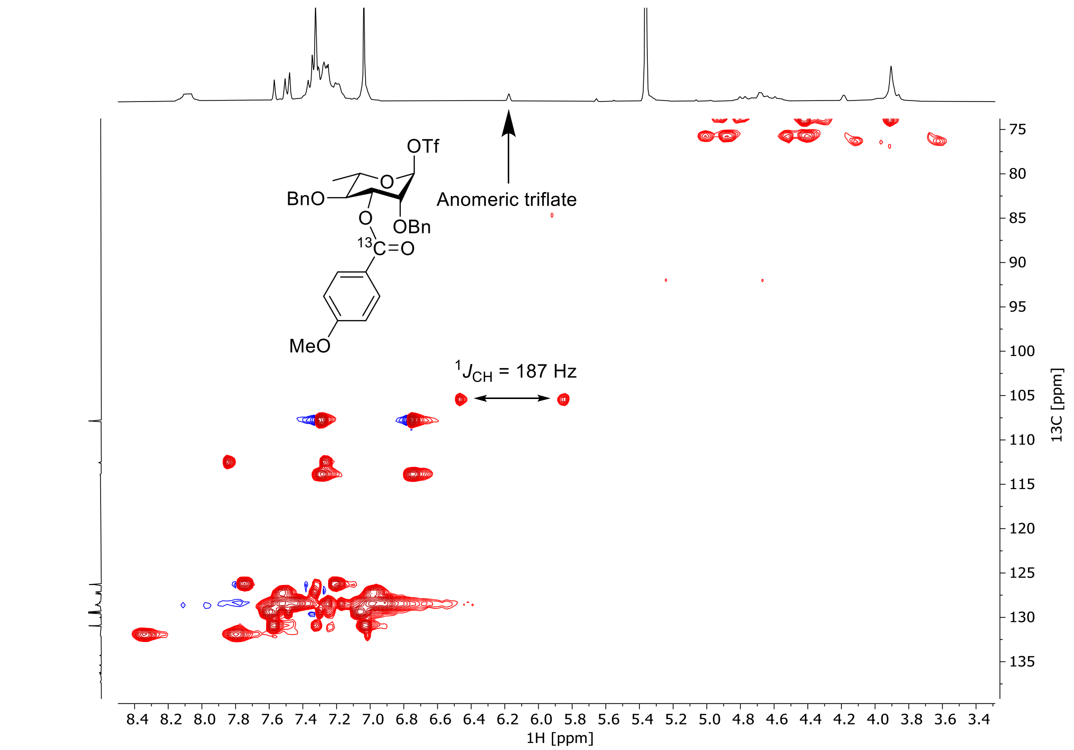

Supplementary Figure 8: ^{13}C-coupled HSQC spectrum of activated donor 4. The HSQC spectrum does not suggest the presence of a dioxanium ion because no cross signals that are typical for dioxanium ions ($\delta_H/\delta_C \approx 6.4/101$ ppm) were observed.[12] The $^1J_{CH}$ coupling of the anomeric triflate confirms that its H-1 is in equatorial position.[13]

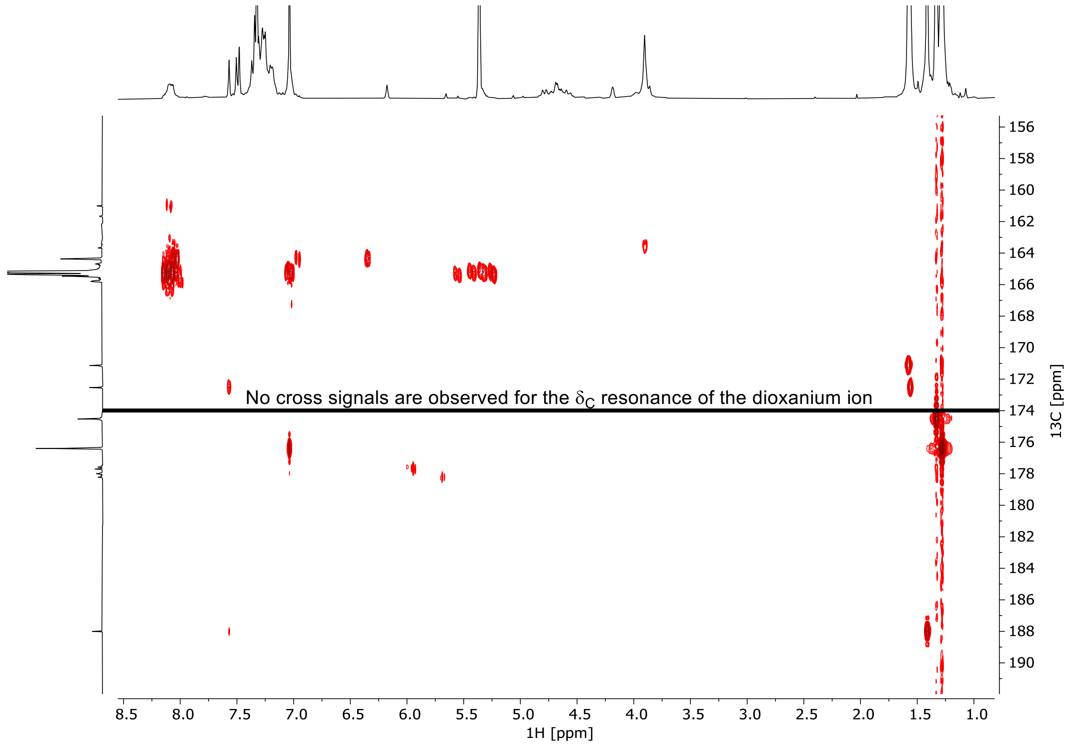

Supplementary Figure 9: HMBC spectrum of activated donor 4. The HMBC spectrum does not suggest the presence of a dioxanium ion because no cross signals that are typical for dioxanium ions ($\delta_C \approx 175$ ppm) were observed.[10]

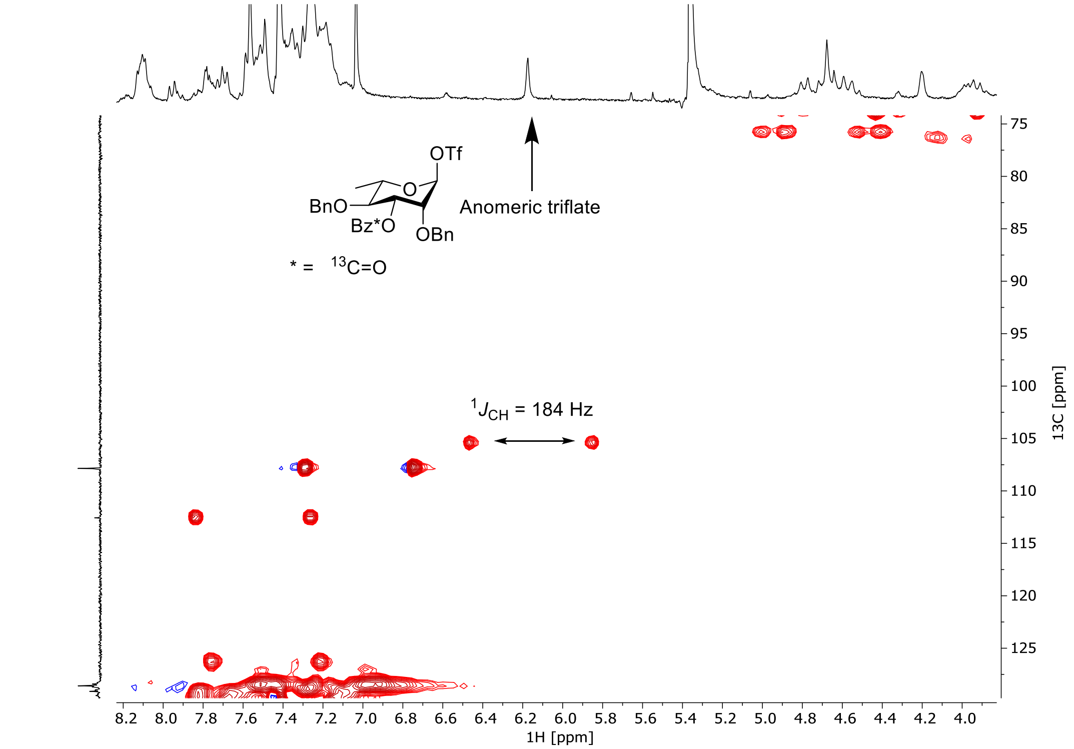

Supplementary Figure 10: ^{13}C-coupled HSQC of activated donor 5. The HSQC spectrum does not suggest the presence of a dioxanium ion because no cross signals that are typical for dioxanium ions ($\delta_H/\delta_C \approx$ 6.4/101 ppm) were observed.[12] The $^1J_{CH}$ coupling of the anomeric triflate confirms that its H-1 is in equatorial position.[13]

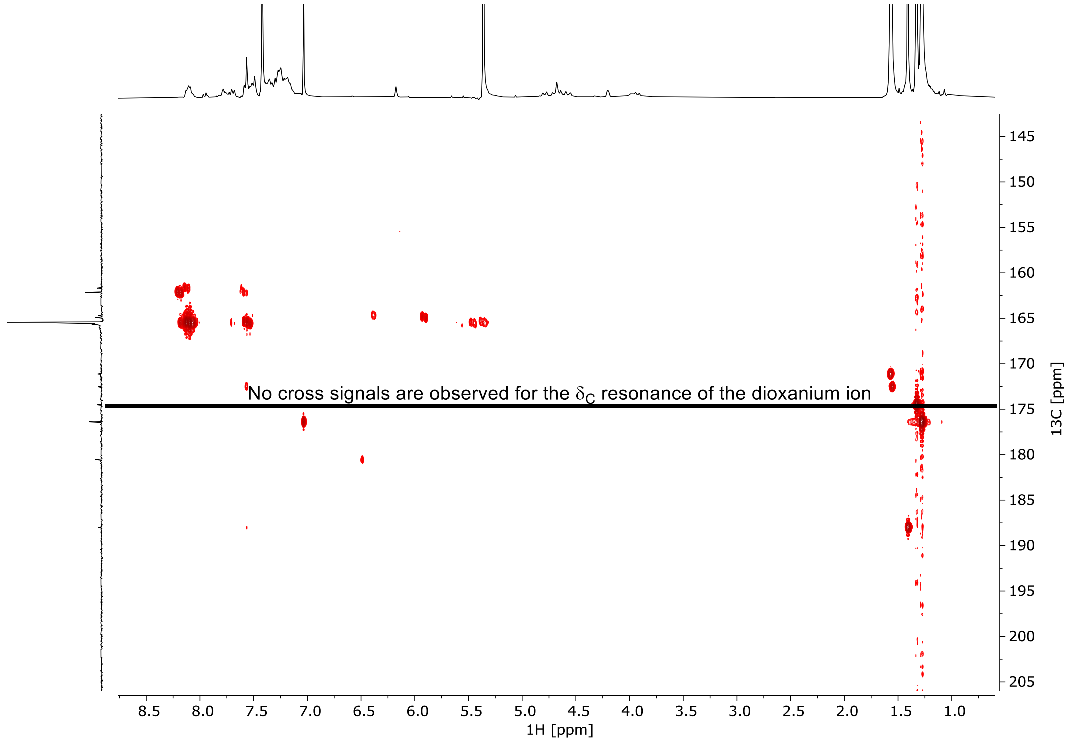

Supplementary Figure 11: HMBC recorded at -80 °C after activation of donor 5. The HMBC spectrum does not suggest the presence of a dioxanium ion because no cross signals that are typical for dioxanium ions ($\delta_C \approx$ 175 ppm) were observed.[12]

Structures

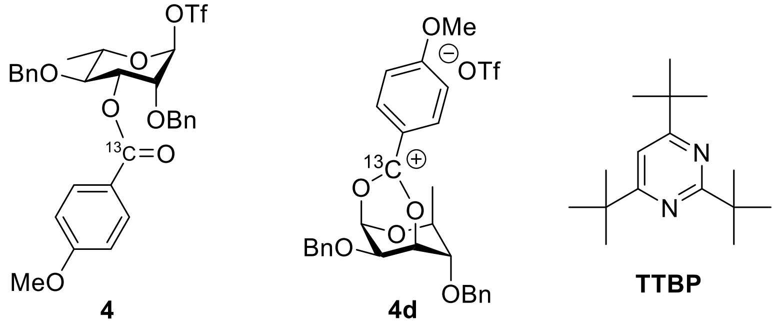

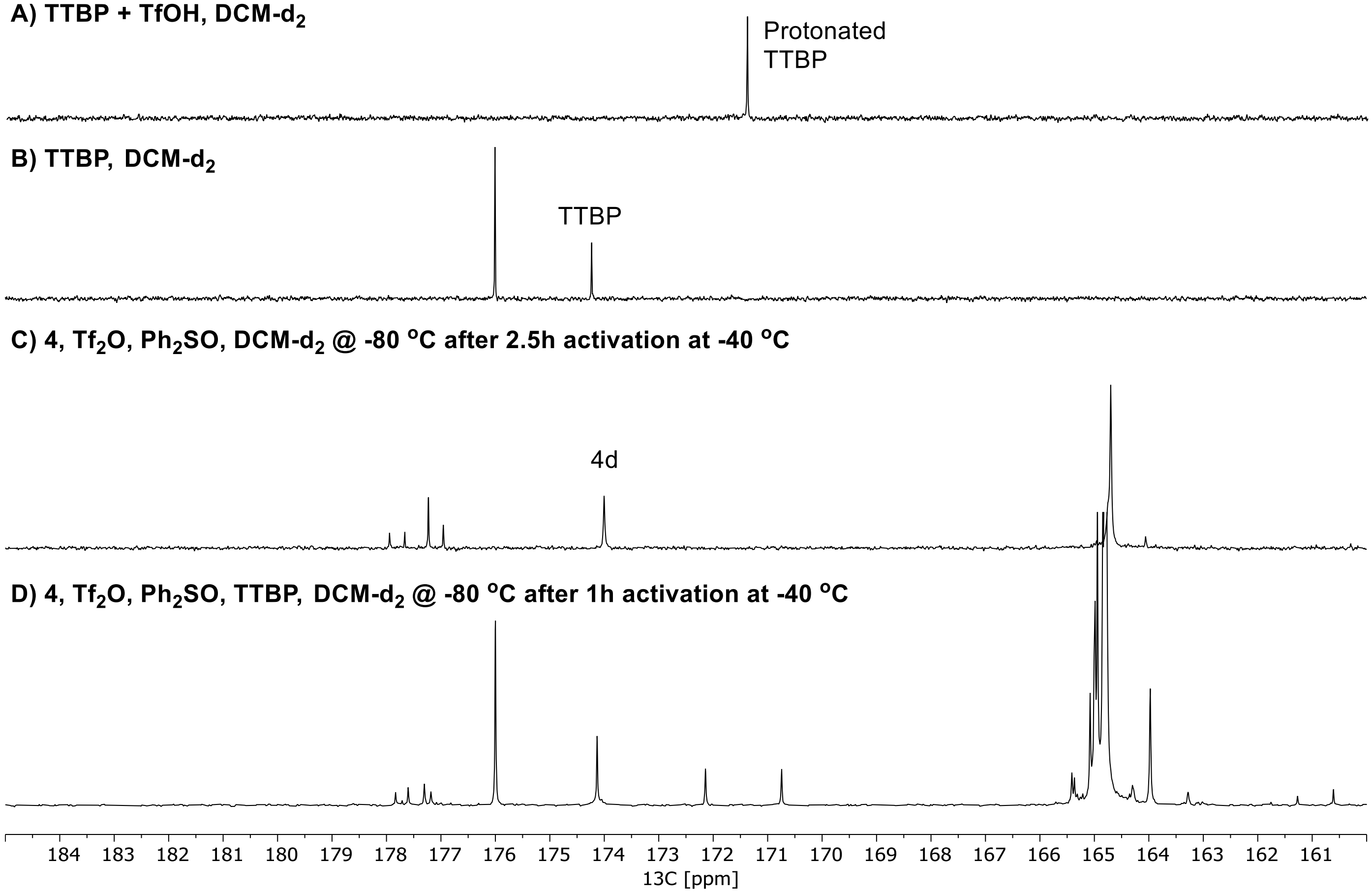

Supplementary Figure 12: Selected ^{13}C-spectra. A) TTBP + TfOH (1:1); **B)** TTBP; **C)** Rhamonosyl donor **4** activated with Tf_2O and Ph_2SO in the absence of TTBP; ***D)*** Rhamonsyl donor **4** activated with Tf_2O and Ph_2SO in the presence of TTBP. From spectrum B+C it becomes apparent that both TTBP and the observable dioxanium ion labelled carbon resonance are in close chemical shift. Comparing spectra B, C and D it suggests that no dioxanium ion resonance is observed. Rather, the signal at 174.2 ppm is attributed to TTBP present in the sample. Compound **4d** is the rhamnosyl dioxanium ion that is rotated 180° for visual purposes.

A) Reaction scheme

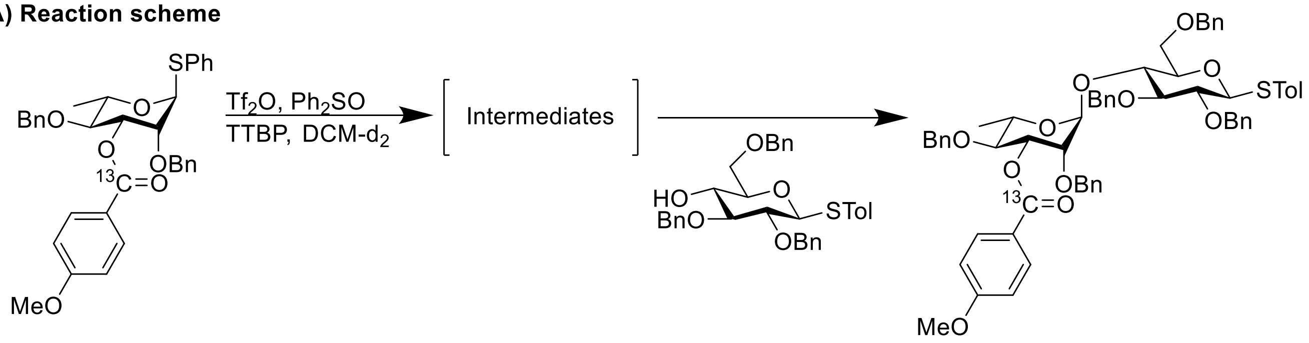

B) HSQC and ^{13}C-coupled HSQC overlayed and zoomed to the anomeric region

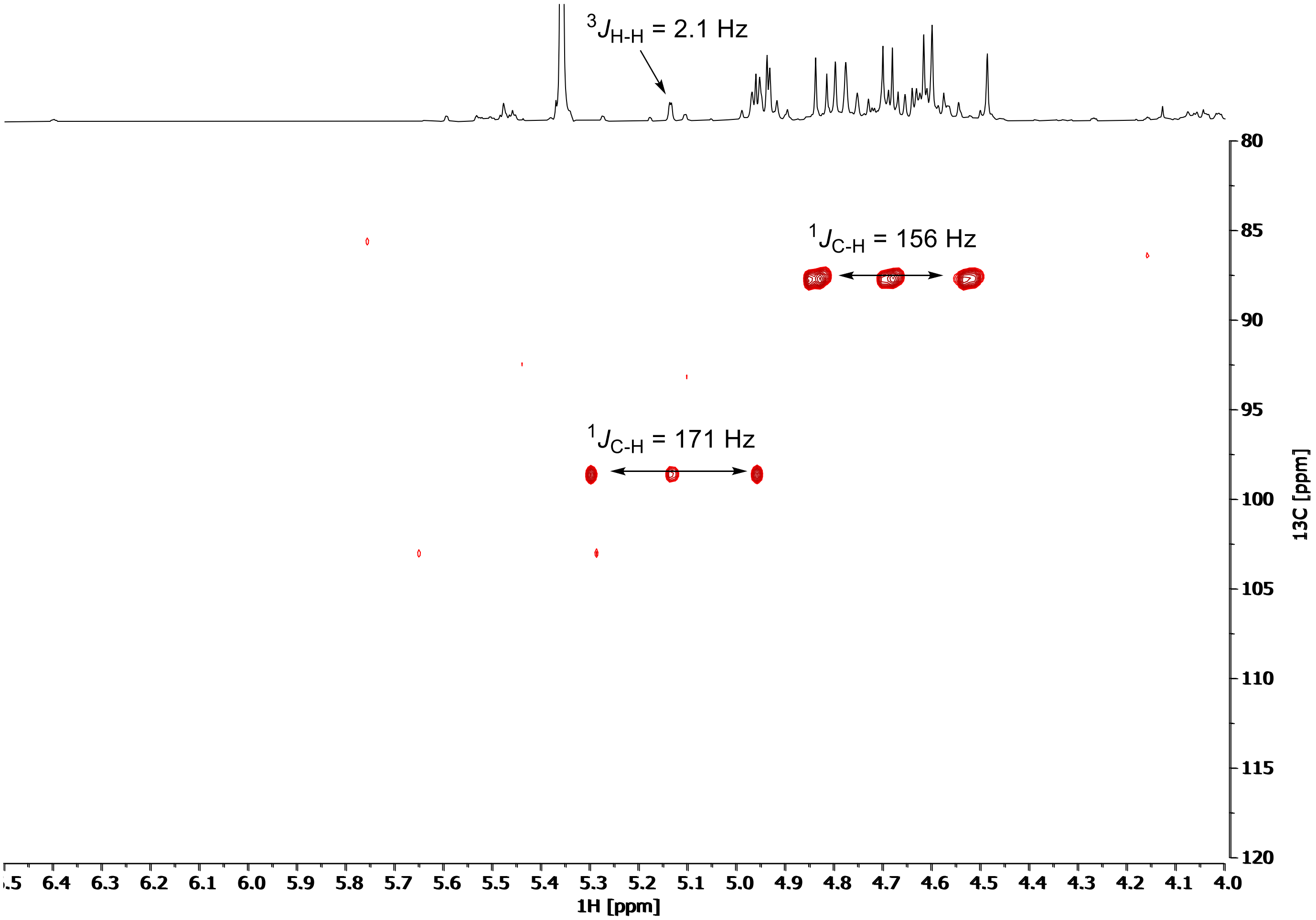

Supplementary Figure 13: Addition of 2,3,6-tri-*O*-benzyl-1-thio-β-D-glucopyranoside to rhamnosyl donor 1. (A) Reaction scheme of activating rhamnosyl donor **1** during VT NMR studies and subsequent addition of the glycosyl acceptor; (B) A HSQC spectrum overlaid with ^{13}C coupled HSQC spectrum that is zoomed in on the anomeric region. The resonance at δ_H/δ_C 4.65/87 ppm is attributed to the remaining acceptor and reducing end of the product. The resonance at δ_H/δ_C 5.12/98 is attributed to the *α*-rhamnose 1→4 glucose linkage ($^1J_{CH} \approx$ 160 for axial ^{1}H; $^1J_{CH} \approx$ 170 for equatorial ^{1}H).[13]

A) Reaction scheme

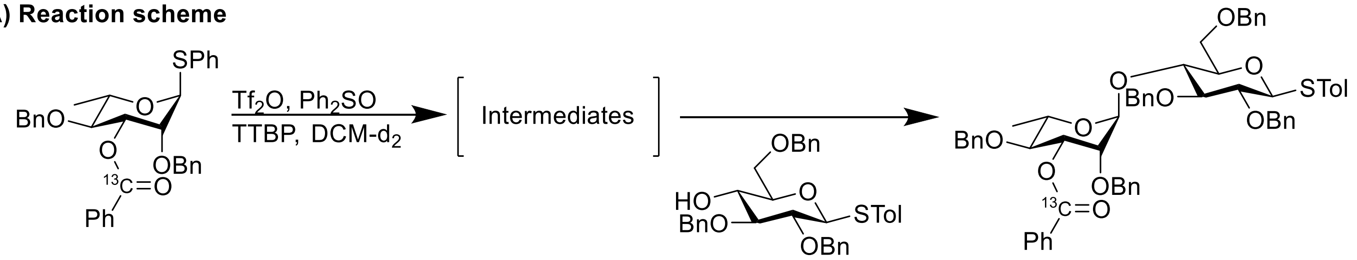

B) HSQC and ^{13}C coupled HSQC overlayed and zoomed to the anomeric region

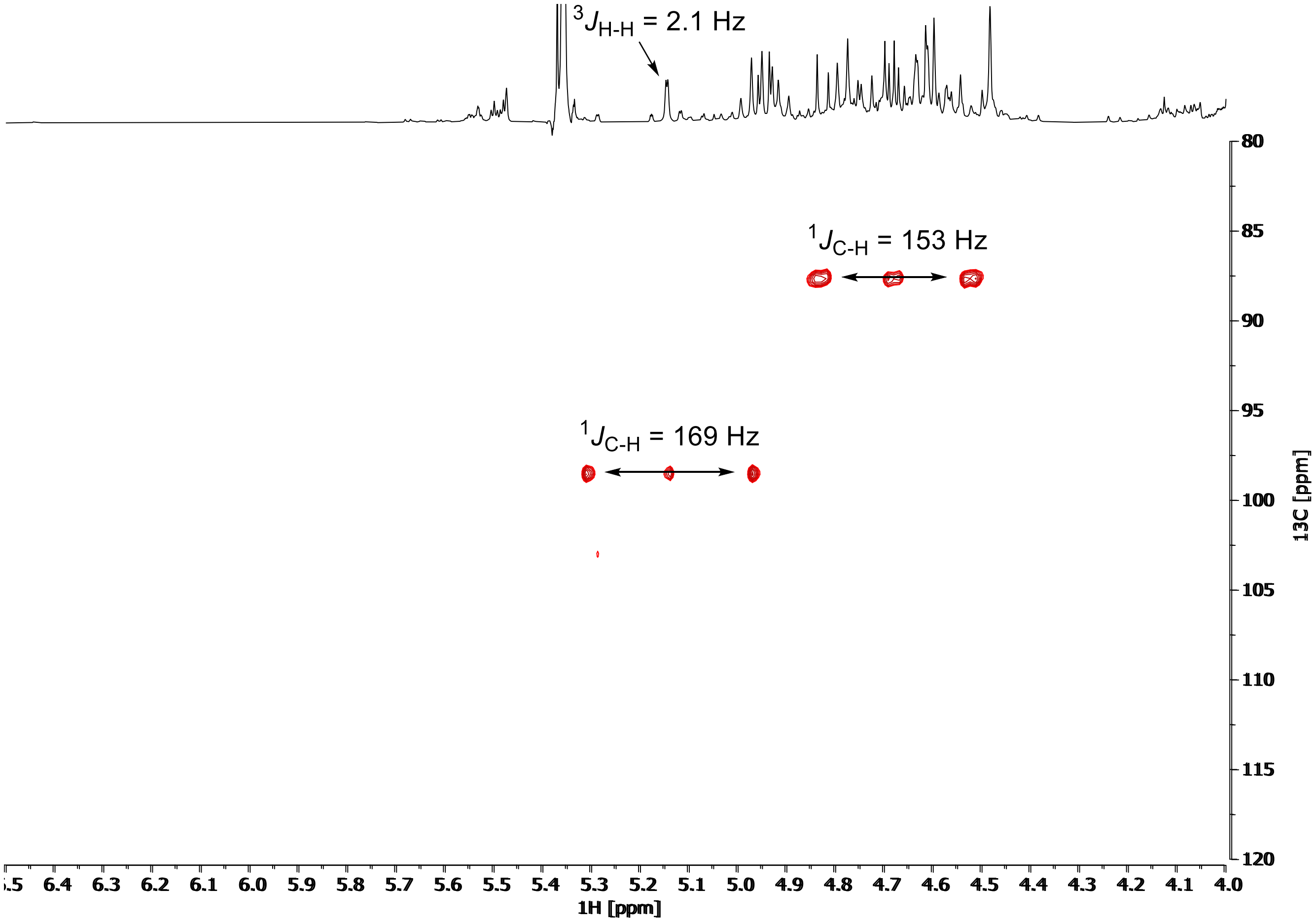

Supplementary Figure 14: Addition of 2,3,6-tri-*O*-benzyl-1-thio-β-D-glucopyranoside to rhamnosyl donor 3. (A) Reaction scheme of activating rhamnosyl donor **3** during VT NMR studies and subsequent addition of the glycosyl acceptor; (B) A HSQC spectrum overlaid with ^{13}C coupled HSQC spectrum that is zoomed in on the anomeric region. The resonance at δ_H/δ_C 4.65/87 ppm is attributed to the remaining acceptor and reducing end of the product. The resonance at δ_H/δ_C 5.12/98 is attributed to the *α*-rhamnose 1→4 glucose linkage ($^1J_{CH} \approx$ 160 for axial ^{1}H; $^1J_{CH} \approx$ 170 for equatorial ^{1}H).[13]

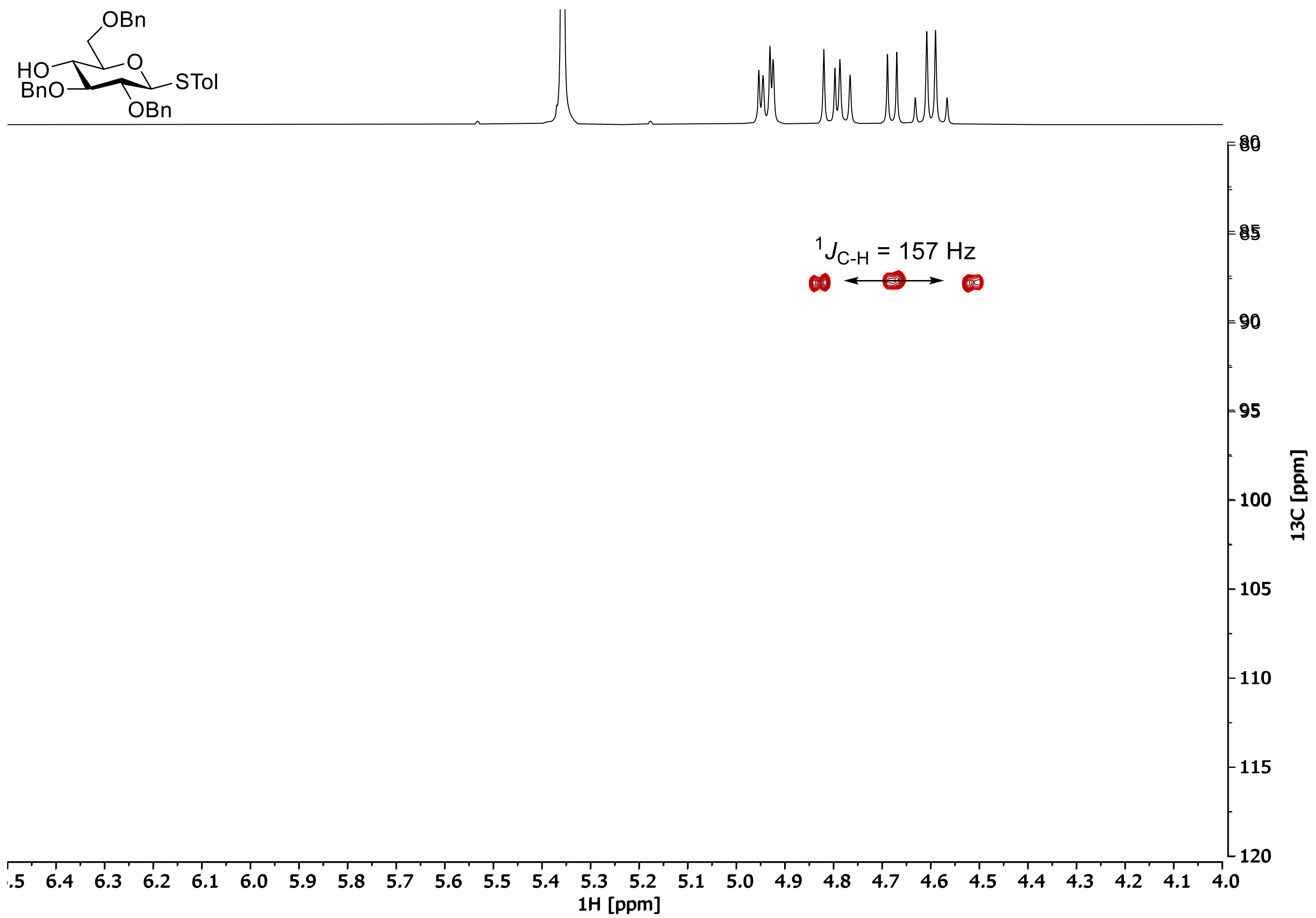

Supplementary Figure 15: A HSQC spectrum overlaid with ^{13}C coupled HSQC spectrum of acceptor 19. Zoomed in on the anomeric region of the acceptor as reference ($^{1}J_{CH}$ ≈ 160 for axial ^{1}H; $^{1}J_{CH}$ ≈ 170 for equatorial ^{1}H).[13]

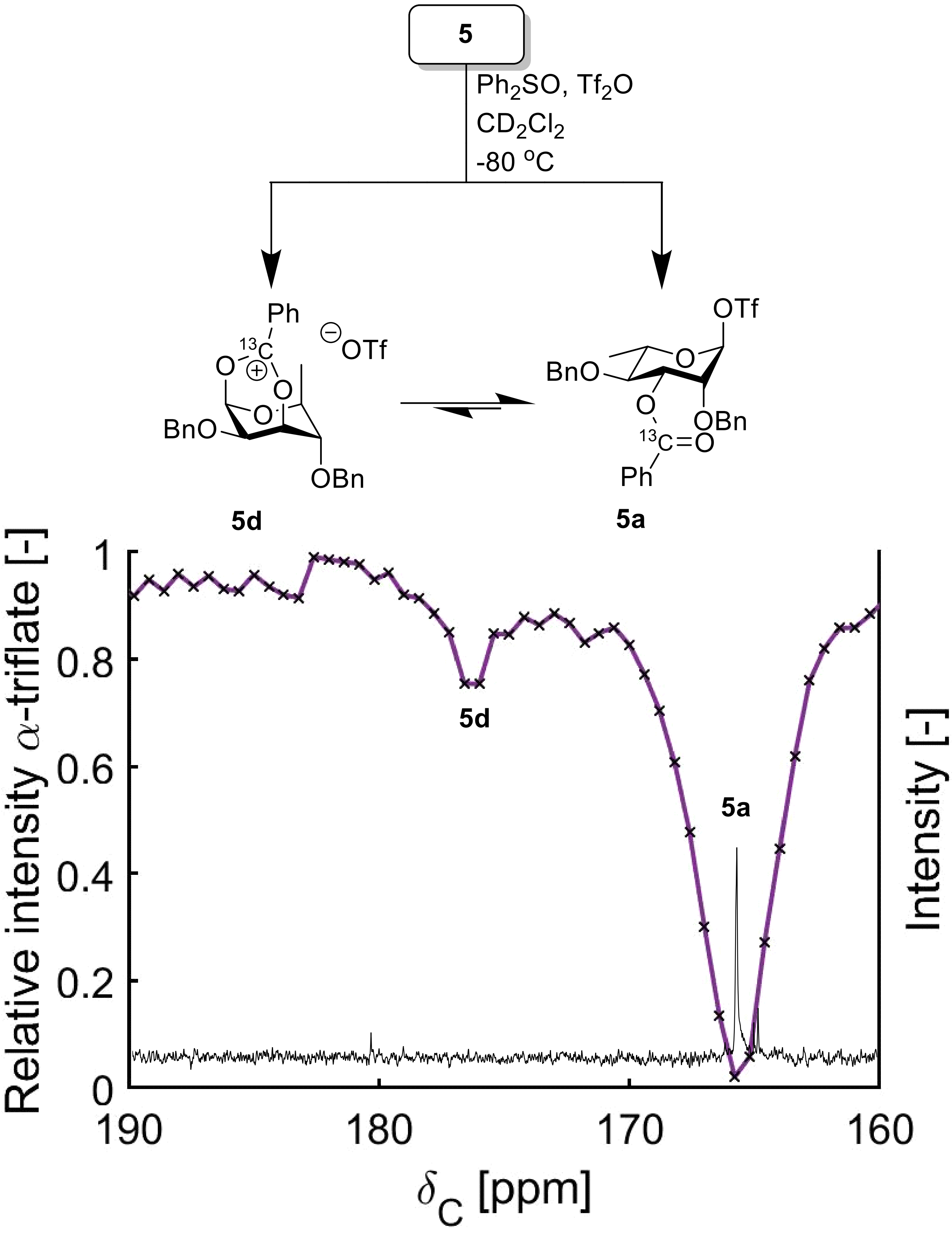

Supplementary Figure 16: Activation scheme plus the ^{13}C CEST spectrum overlaid with the 1D ^{13}C NMR spectrum. Both spectra were recorded at -80°C in the absence of TTBP. ^{13}C-CEST NMR shows the dioxanium ion as minor signal at δ_C = 176.2 ppm. The dioxanium ion resonance is still not visible in 1D ^{13}C NMR (512 scans).

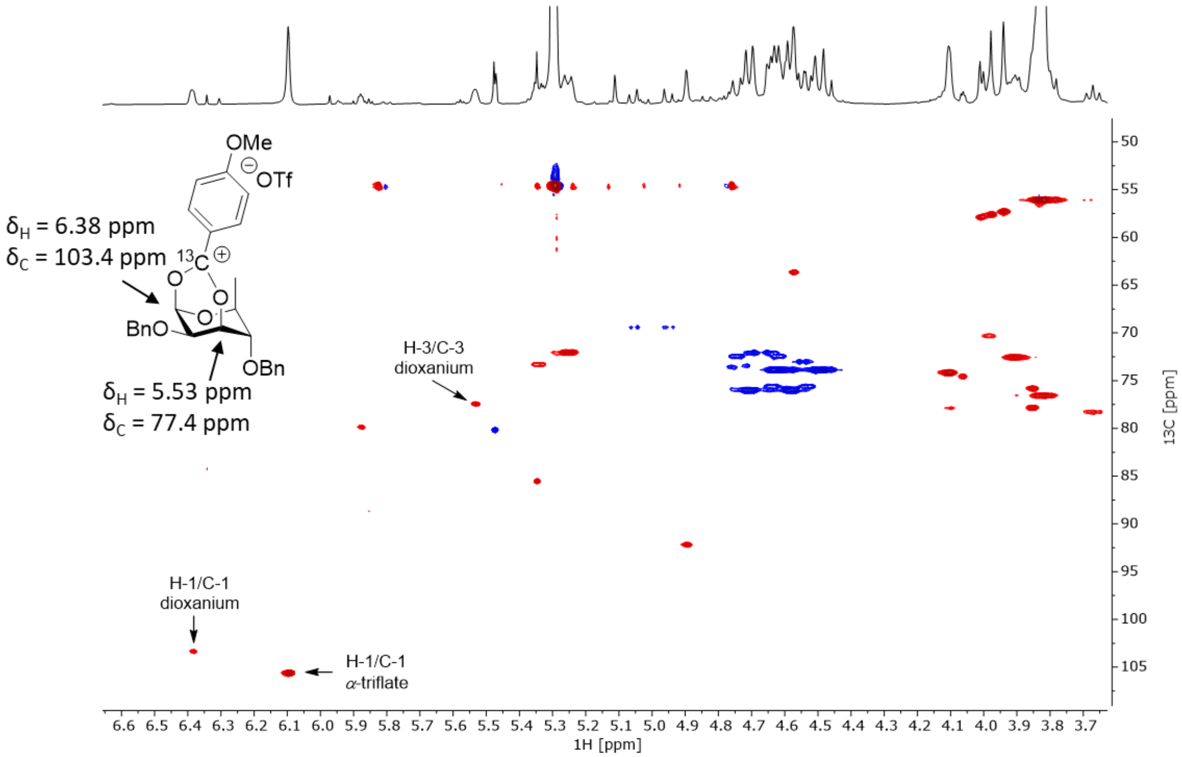

Supplementary Figure 17: HSQC recorded at -80°C. Dioxanium C1/H1 and C3/H3 were conveniently determined using the HMBC and COSY spectra. Further characterization was limited due to the typical low *J*-coupling for axial-equatorial and equatorial-equatorial coupling.

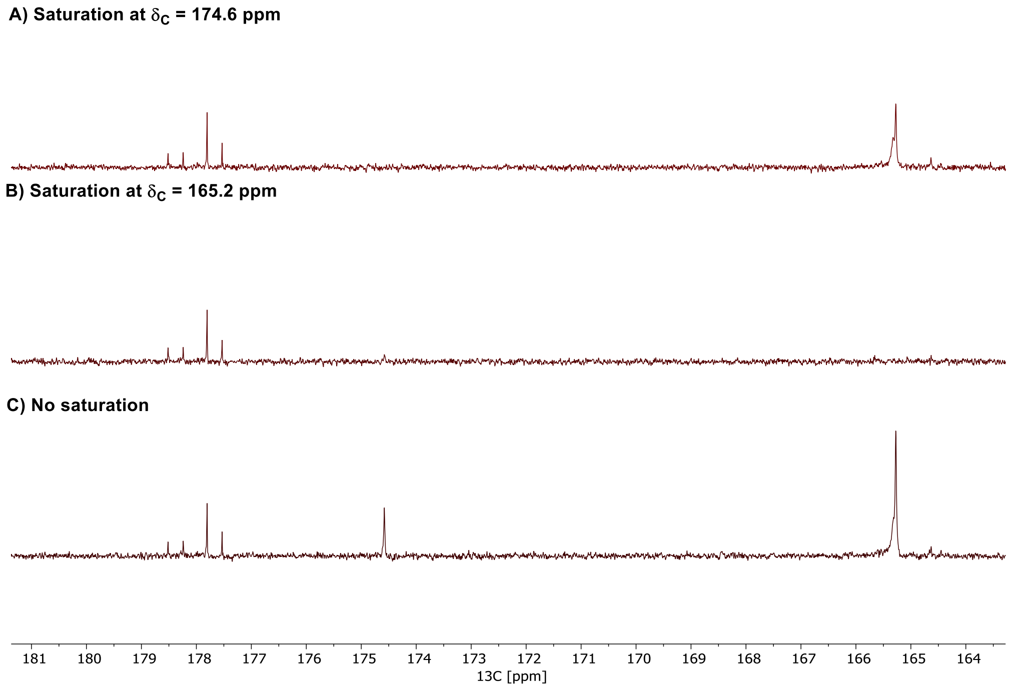

Supplementary Figure 18: Three-point CEST NMR. (A) Saturation at δ_C = 174.6 ppm; (B) Saturation at δ_C = 165.2 ppm; (C) No saturation applied. Upon comparing the spectra it becomes apparent that saturating the resonance at δ_C = 174.6 ppm affects the carbonyl peak at δ_C = 165.2 and vice versa. Therefore, both are in chemical exchange.

Supplementary Table 2: Formamide-adduct assisted glycosylations.

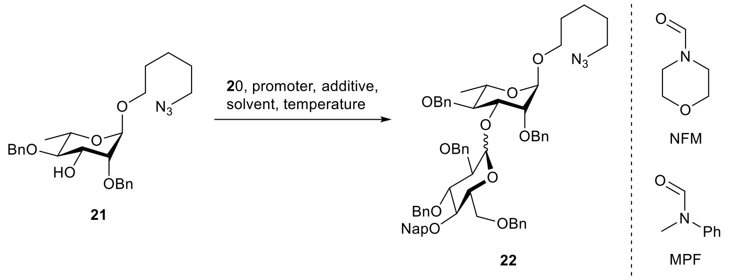

Entry	Promoter	Solvent	Additive	Yield[e] (%)	Ratio (α:β)
1[a]	TfOH/NIS	DCM	Et_2O	94	1.9:1
2[a]	TfOH/NIS	THF	-	N.D.[f]	2.5:1[h]
3[a]	TMSOTf/NIS	DCM	Et_2O	75	1.6:1
4[a]	TMSOTf/NIS	DCM	THF	62	2:1
5[b]	TMSOTf/NIS	DCM	DMF	88	10:1
6[b]	TMSOTf/NIS	DCM	DMF	68	5:1
7[c]	TMSOTf/NIS	DCM	NFM/TBAI	56	>20:1
8[c]	TMSOTf/NIS	DCM	MPF	N.D.[g]	3:1[h]
9[c]	TMSOTf/NIS	DCM	NFM/TBAI	30	>20:1
10[d]	TMSOTf/NIS	DCM	DMF	85	6:1

[a] Reaction performed at -78°C – R.T.; [b] Reaction performed at -10°C – R.T.;
[c] Reaction performed at -20°C – R.T.; [d] Reaction performed at 0°C – R.T.;
[e] Isolated yields of the disaccharide as a mixture of anomers; [f] Product was not isolated due to a large amount of impurities as a result of the TfOH-catalyzed ring-opening of THF; [g] Separation of the product from MPF was unsuccessful;
[h] Ratios were determined in the crude reaction mixture using key integrals in the ^{1}H NMR spectra.

Synthesis materials & methods

General synthetic conditions

Synthetic product characterisations were recorded with a Bruker 500 MHz AVANCE III spectrometer or JEOL 500 ECZ-R spectrometer. The Bruker 500 MHz Avance III spectrometer is equipped with a Prodigy BB cryoprobe. The JEOL 500 ECZ-R spectrometers were equipped with either a SuperCOOL broadband probe, ROYAL broadband probe, or ROYAL HFX broadband probe. Chemical shifts are reported in parts per million (ppm) with tetramethylsilane (TMS) as the internal standard or solvent residual signals (SRP) if stated otherwise. ^{1}H NMR spectroscopic data is presented as follows: chemical shift, multiplicity (s = singlet, d = doublet, t = triplet, dd = doublet of doublets, dt = doublet of triplets, m = multiplet and/or multiple resonances), coupling constant (*J*) in hertz (Hz), integration and assignments. All NMR signals were assigned based on ^{1}H NMR, ^{13}C NMR, COSY, HSQC, HMBC, TOCSY, NOESY and ROESY experiments. Mass spectra were recorded with a JEOL JMST100CS AccuTOF mass spectrometer. Automatic silica-flash column chromatography was done with a Biotage Isolera Spektra One, using pre-packed cartridges ultrapure irregular silica gel (Screening Devices, 40-63 µm, 60 Å). Gel-filtration chromatography was performed using polyacrylamide Bio-Gel P2 beads (Bio-rad, Milli-Q as eluent) or styrene divinylbenzene Bio-beads S-X1 resin (Bio-rad, DCM as eluent). TLC analysis was conducted on Silica gel F254 (Merck KGaA) with detection by UV absorption (254 nm) where applicable and by dipping in a stain followed by heating. Stains used for TLC analysis were either 10% sulphuric acid in MeOH, cerium molybdate stain (0.03 M $(NH_4)_6Mo_7O_{24}\cdot 4H_2O$; 6 mM $Ce(NH_4)_4(SO_4)_4\cdot 2H_2O$; 1 M H_2SO_4 in H_2O), potassium permanganate (0.06 M $KMnO_4$; 0.5 M, K_2CO_3; 0.02 M NaOH in H_2O) or ninhydrin (0.08 M ninhydrin in *n*-BuOH:AcOH, 97:3 v/v). Primary azides were stained by dipping them in 10% PPh_3 in DCM prior to dipping them in a ninhydrin stain. Reactions that used anhydrous solvents were performed under Schlenk conditions and were conducted under an argon atmosphere. Molecular Sieves (0.4 nm) were activated overnight by heating *in vacuo* at 150°C. Reactions with 2,3-dichloro-5,6-dicyano-1,4-benzoquinone (DDQ) were washed with an aqueous solution of 0.7% ascorbic acid, 1.5% citric acid and 0.9% NaOH (0.9%), abbreviated as DDQ mixture.

Pre-mix activation conditions of rhamnosyl donor 1 with 5-azidopentanol

Donor (81 mg, 0.14 mmol, 1.0 eq) was dissolved in anh. DCM (3.3 mL). 5-Azidopentanol (23 µL, 0.19 mmol, 1.3 eq) was added. The solution was cooled down to 0°C. Molecular sieves (4Å) were added, after which the solution was stirred for 90 min. The solution was cooled down to -78°C. NIS (35 mg, 0.16 mmol, 1.0 eq) and TfOH (2.0 µL, 23 µmol, 0.16 eq) were added, respectively. The solution was stirred at -78°C to 0°C for 30 min, turning bright red over time. The solution was quenched with TEA (0.15 mL) and stirred for an additional 10 min. The mixture was filtered over celite, after which the filtrate was washed with 10% aq. $Na_2S_2O_3$ (5.0 mL). The organic layer was dried with $MgSO_4$, filtered and evaporated *in vacuo*. The residue was purified using silica-flash column chromatography (0 – 6% EtOAc in Tol), yielding monosaccharide **19** as a colourless oil (79 mg, 0.13 mmol, 94%, 37:1 α:β).

Pre-activation conditions of rhamnosyl donor 1

Donor (49 mg, 86 µmol, 1.0 eq), Ph_2SO (27 mg, 0.13 mmol, 1.6 eq) and TTBP (44 mg, 0.17 mmol, 2.0 eq) were dissolved in anh. DCM (2.0 mL). The solution was cooled down to 0°C. Molecular sieves (4Å) were added, after which the solution was stirred for 90 min. The solution was cooled down to -78°C. Tf_2O (19 µL, 0.11 mmol, 1.3 eq) was added, after which the solution was stirred for 15 min. 5-azidopentanol (14 µL, 0.11 mmol, 1.3 eq) was added. The solution was stirred at -78°C for 30 min and subsequently stirred at -78°C – 0°C for an additional 90 min. The mixture was filtered, after which the filtrate was diluted and washed with 10% aq. $NaHCO_3$ (sat.) (10 mL). The organic layer was dried with $MgSO_4$, filtered and evaporated *in vacuo*. The residue was purified using silica-flash column chromatography (0 – 6% EtOAc in Tol), yielding monosaccharide **19** as a colourless oil (50 mg, 73 µmol, 85%, α only).

5-Azidopentyl 3-*O*-(*p*-anisoyl)-2,4-di-*O*-benzyl-α-L-rhamnopyranoside (6)

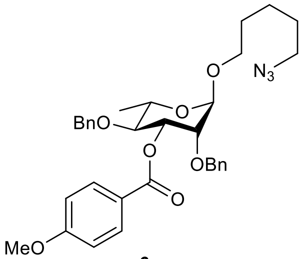

6

<u>TLC</u>: (EtOAc:Tol, 6:94 v/v): R_f =0.42; **<u>^{1}H NMR</u>** (500 MHz, $CDCl_3$): δ 8.02 (d, *J* = 8.9 Hz, 2H, 2x Ar***H***, OAnis), 7.29 – 7.25 (m, 2H, 2x Ar***H***, OBn), 7.24 – 7.17 (m, 8H, 8x Ar***H***, OBn), 6.93 (d, *J* = 8.9 Hz, 2H, 2x Ar***H***, OAnis), 5.46 (dd, *J* = 3.4, 1.9 Hz, 1H, ***H-3***), 4.77 – 4.73 (m, 2H, ***H-1***, PhC$\boldsymbol{H_a}H_b$, 4-OBn), 4.64 – 4.61 (m, 3H, PhC$\boldsymbol{H_2}$, 2-OBn; PhC$H_a\boldsymbol{H_b}$-, 4-OBn), 3.95 (dd, *J* = 3.4, 1.9 Hz, 1H, ***H-2***), 3.88 (s, 3H, OC$\boldsymbol{H_3}$, OAnis), 3.85 – 3.76 (m, 2H, ***H-4***, ***H-5***), 3.67 (dt, *J* = 9.6, 6.5 Hz, 1H, -OC$\boldsymbol{H_a}H_bCH_2$), 3.38 (dt, *J* = 9.6, 6.3 Hz, 1H, OC$H_a\boldsymbol{H_b}CH_2$), 3.27 (t, *J* = 6.9 Hz, 2H, C$\boldsymbol{H_2}N_3$), 1.64 – 1.56 (m, 4H, $OCH_2C\boldsymbol{H_2}$, C$\boldsymbol{H_2}CH_2N_3$), 1.43 (ddd, *J* = 11.8, 9.2, 5.7 Hz, 2H, C$\boldsymbol{H_2}CH_2CH_2N_3$), 1.36 (d, *J* = 5.8 Hz, 3H, 6-C$\boldsymbol{H_3}$); **<u>^{13}C NMR</u>** (126 MHz, $CDCl_3$): δ 165.45 (***C***=O, OAnis), 163.52 (Ar***C***OMe), 137.98 (Ar***C***CH_2, OBn), 137.94 (Ar***C***CH_2, OBn), 131.79 (OAnis), [128.34, 128.31, 128.00, 127.78, 127.73, 127.70 (OBn)], 122.54 (Ar***C***CO_2, OAnis), 113.75 (OAnis), 98.00 (***C-1***), 79.23 (***C-4***), 76.47 (***C-2***), 75.19 (Ph***C***H_2, 4-OBn), 74.23 (***C-3***), 73.23 (Ph***C***H_2, 2-OBn), 67.79 (***C-5***), 67.34 (O***C***H_2CH_2), 55.48 (O***C***H_3, OAnis), 51.32 (***C***H_2N_3), 29.00 (OCH_2***C***H_2), 28.66 (***C***$H_2CH_2N_3$), 23.44 (***C***$H_2CH_2CH_2N_3$), 18.08 (***C-6***); **<u>HR-ESI-TOF/MS (m/z)</u>:** $[M+Na]^+$ calcd. for $C_{33}H_{39}N_{14}O_{16}Na$, 612.26857; found, 612.26899.

Pre-mix activation conditions of rhamnosyl donor 2 with 5-azidopentanol

Donor (54 mg, 0.10 mmol, 1.0 eq) was dissolved in anh. DCM (2.4 mL). 5-Azidopentanol (16 µL, 0.13 mmol, 1.3 eq) was added. The solution was cooled down to 0°C. Molecular sieves (4Å) were added, after which the solution was stirred for 90 min. The solution was cooled down to -78°C. NIS (25 mg, 0.11 mmol, 1.1 eq) and TfOH (2.0 µL, 23 µmol, 0.23 eq) were added, respectively. The solution was stirred at -78°C to 0°C for 30 min, turning bright red over time. The solution was quenched with TEA (0.15 mL) and stirred for an additional 10 min. The mixture was filtered over celite, after which the filtrate was washed with 10% aq. $Na_2S_2O_3$ (5.0 mL). The organic layer was dried with $MgSO_4$, filtered and evaporated *in vacuo*. The residue was purified using silica-flash column chromatography (0 – 6% EtOAc in Tol), yielding monosaccharide **30** as a pale yellow oil (38 mg, 70 µmol, 68%, 1.8:1 α:β).

Pre-activation conditions of rhamnosyl donor 2

Donor (56 mg, 0.11 mmol, 1.0 eq), Ph_2SO (33 mg, 0.16 mmol, 1.5 eq) and TTBP (54 mg, 0.21 mmol, 1.9 eq) were dissolved in anh. DCM (2.4 mL). The solution was cooled down to 0°C. Molecular sieves (4Å) were added, after which the solution was stirred for 90 min. The solution was cooled down to -78°C. Tf_2O (23 µL, 0.14 mmol, 1.3 eq) was added, after which the solution was stirred for 15 min. 5-azidopentanol (18 µL, 0.14 mmol, 1.3 eq) was added. The solution was stirred at -78°C for 30 min and subsequently stirred at -78°C – 0°C for an additional 90 min. The mixture was filtered, after which the filtrate was diluted and washed with 10% aq. $NaHCO_3$ (sat.) (10 mL). The organic layer was dried with $MgSO_4$, filtered and evaporated *in vacuo*. The residue was purified using silica-flash column chromatography (0 – 6% EtOAc in Tol), yielding monosaccharide **30** as a colourless oil (47 mg, 86 µmol, 81%, 1.4:1 α:β).

5-Azidopentyl 2,3,4-tri-*O*-benzyl-α-L-rhamnopyranoside (7)

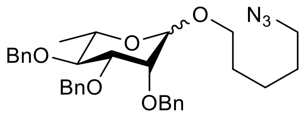

7

<u>α-Anomer:</u> <u>TLC</u>: (EtOAc:Tol, 6:94 v/v): R_f =0.40; **<u>^{1}H NMR</u>** (500 MHz, $CDCl_3$): δ 7.39 – 7.25 (m, 15H, Ar***H***, OBn), 5.00 – 4.92 (m, 1H, PhC$\boldsymbol{H_a}H_b$, 4-OBn), 4.77 (d, *J* = 12.5 Hz, 1H, PhC$\boldsymbol{H_a}H_b$, 2-OBn), 4.74 – 4.70 (m, 2H, ***H-1***, PhC$H_a\boldsymbol{H_b}$, 2-OBn), 4.67 – 4.62 (m, 3H, PhC$\boldsymbol{H_2}$, 3-OBn; Ph-C$H_a\boldsymbol{H_b}$, 4-OBn), 3.85 (dd, *J* = 9.1, 3.1 Hz, 1H, ***H-3***), 3.76 (dd, *J* = 3.1, 1.9 Hz, 1H, ***H-2***), 3.71 – 3.58 (m, 3H, ***H-4***, **H-5**, OC$\boldsymbol{H_a}H_bCH_2$), 3.35 – 3.22 (m, 3H, C$\boldsymbol{H_2}N_3$, OC$H_a\boldsymbol{H_b}CH_2$), 1.73 – 1.40 (m, 6H, 1-O-CH_2-**CH_2-CH_2-CH_2**-CH_2-N_3), 1.33 (d, *J* = 6.0 Hz, 3H, 6-C$\boldsymbol{H_3}$); **<u>^{13}C NMR</u>** (126 MHz, $CDCl_3$): δ 138.62 (Ar***C***CH_2, OBn), 138.58 (Ar***C***CH_2, OBn), 138.40 (Ar***C***CH_2, OBn), [128.42, 128.38, 128.36, 128.10, 127.91, 127.67, 127.65, 127.63, 127.53 (OBn)], 97.99 (***C-1***), 80.55 (***C-4***), 80.18 (***C-3***), 75.45 (Ph***C***H_2, 4-OBn), 75.04 (***C-2***), 72.82 (Ph***C***H_2, 2-OBn), 72.17 (Ph***C***H_2, 2-OBn), 68.03 (***C-5***), 67.10 (O***C***H_2CH_2), 51.28 (-***C***H_2N_3), 28.96 (OCH_2***C***H_2), 28.63 (***C***$H_2CH_2N_3$), 23.40 (***C***$H_2CH_2CH_2N_3$), 18.01 (***C-6***).

<u>β-Anomer:</u> <u>TLC</u>: (EtOAc:Tol, 6:94 v/v): R_f =0.37; **<u>^{1}H NMR</u>** (500 MHz, $CDCl_3$): δ 7.48 – 7.45 (m, 2H, Ar***H***, OBn), 7.39 – 7.25 (m, 13H, Ar***H***, OBn), 5.00 – 4.92 (m, 2H, PhC$\boldsymbol{H_a}H_b$, 2-OBn; PhC$\boldsymbol{H_a}H_b$, 4-OBn), 4.86 (d, *J* = 12.6 Hz, 1H, PhC$H_a\boldsymbol{H_b}$, 2-OBn), 4.67 – 4.62 (m, 1H, PhC$H_a\boldsymbol{H_b}$, 4-OBn), 4.51 (d, *J* = 11.9 Hz, 1H, PhC$\boldsymbol{H_a}H_b$, 3-OBn), 4.45 (d, *J* = 11.8 Hz, 1H, Ph-C$H_a\boldsymbol{H_b}$, 3-OBn), 4.32 (d, *J* = 0.8 Hz, 1H, ***H-1***), 3.95 (dt, *J* = 9.3, 6.3 Hz, 1H, OC$\boldsymbol{H_a}H_bCH_2$), 3.89 (d, *J* = 3.0 Hz, 1H, ***H-2***), 3.71 – 3.58 (m, 1H, ***H-4***), 3.45 (dd, *J* = 9.4, 3.0 Hz, 1H, ***H-3***), 3.40 (dt, *J* = 9.4, 6.5 Hz, 1H, OC$H_a\boldsymbol{H_b}CH_2$), 3.35 – 3.22 (m, 3H, ***H-5***, C$\boldsymbol{H_2}N_3$), 1.73 – 1.40 (m, 6H, 1-O-CH_2-**CH_2-CH_2-CH_2**-CH_2-N_3), 1.38

(d, *J* = 6.3 Hz, 3H, 6-C***H***$_3$); **^{13}C NMR** (126 MHz, $CDCl_3$): δ 138.78 (Ar***C***CH$_2$, OBn), 138.51 (Ar***C***CH$_2$, OBn), 138.25 (Ar***C***CH$_2$, OBn), [128.42, 128.38, 128.36, 128.13, 128.08, 127.69, 127.56, 127.55, 127.42 (OBn)], 101.56 (***C-1***), 82.20 (***C-3***), 80.55 (***C-4***), 75.45 (Ph***C***H$_2$, 4-OBn), 73.93 (***C-2***), 73.88 (Ph***C***H$_2$, 3-OBn), 71.94 (***C-5***), 71.40 (Ph***C***H$_2$, 3-OBn), 69.41 (O***C***H$_2$CH$_2$), 51.38 (***C***H$_2$N$_3$), 29.26 (OCH$_2$***C***H$_2$), 28.65 (***C***H$_2$CH$_2$N$_3$), 23.42 (***C***H$_2$CH$_2$CH$_2$N$_3$), 17.98 (***C-6***).
HR-ESI-TOF/MS (m/z): $[M+Na]^+$ calcd. for $C_{32}H_{39}N_3O_5Na$, 568.2782; found, 568.2787.

Pre-mix activation conditions of rhamnosyl donor 3 with 5-azidopentanol
Donor (61 mg, 0.11 mmol, 1.0 eq) was dissolved in anh. DCM (2.5 mL). 5-Azidopentanol (20 µL, 0.16 mmol, 1.4 eq) was added. The solution was cooled down to 0°C. Molecular sieves (4Å) were added, after which the solution was stirred for 90 min. The solution was cooled down to -78°C. NIS (26 mg, 0.12 mmol, 1.0 eq) and TfOH (2.0 µL, 23 µmol, 0.20 eq) were added, respectively. The solution was stirred at -78°C to 0°C for 30 min, turning bright red over time. The solution was quenched with TEA (0.15 mL) and stirred for an additional 10 min. The mixture was filtered over celite, after which the filtrate was washed with 10% aq. $Na_2S_2O_3$ (10 mL) and aq. $NaHCO_3$ (sat.) (10 mL), respectively. The organic layer was dried with $MgSO_4$, filtered and evaporated *in vacuo*. The residue was purified using silica-flash column chromatography (0 – 6% EtOAc in Tol), yielding monosaccharide **31** as a colorless oil (53 mg, 95 µmol, 84%, 21:1 α:β).
Pre-activation conditions of rhamnosyl donor 3
Donor (59 mg, 0.11 mol, 1.0 eq), Ph_2SO (33 mg, 0.16 mmol, 1.5 eq) and TTBP (55 mg, 0.22 mmol, 2.0 eq) were dissolved in anh. DCM (2.5 mL). The solution was cooled down to 0°C. Molecular sieves (4Å) were added, after which the solution was stirred for 90 min. The solution was cooled down to -78°C. Tf_2O (24 µL, 0.14 mmol, 1.3 eq) was added, after which the solution was stirred for 15 min. 5-azidopentanol (21 µL, 0.16 mmol, 1.5 eq) was added. The solution was stirred at -78°C for 30 min and subsequently stirred at -78°C – 0°C for an additional 90 min. The mixture was filtered over celite, after which the filtrate was diluted with DCM and washed with 10% aq. $NaHCO_3$ (sat.) (10 mL). The organic layer was dried with $MgSO_4$, filtered and evaporated *in vacuo*. The residue was purified using silica-flash column chromatography (0 – 6% EtOAc in Tol), yielding monosaccharide **31** as a colourless oil (56 mg, 0.10 mmol, 92%, α only).

5-Azidopentyl 3-*O*-benzoyl-2,4-di-*O*-benzyl-α-L-rhamnopyranoside (31)

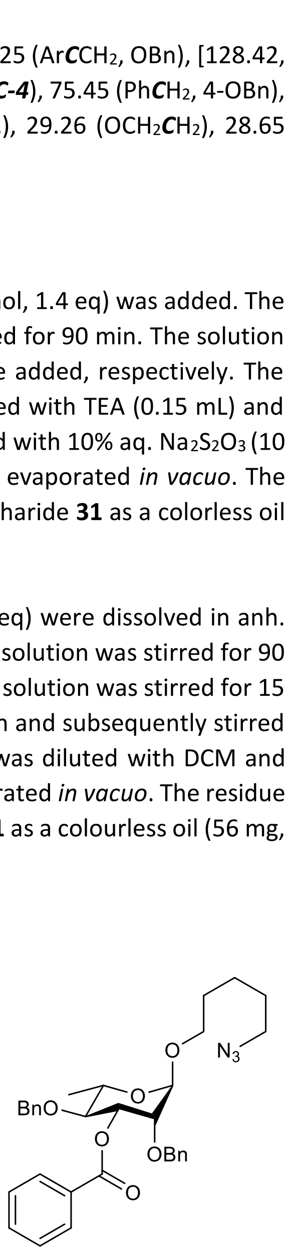

31

TLC: (EtOAc:Tol, 10:90 v/v): R_f = 0.55; **^{1}H NMR** (500 MHz, $CDCl_3$): δ 8.07 – 8.04 (m, 2H, 2x Ar***H***, OBz), 7.60 – 7.56 (m, 1H, Ar***H***, OBz), 7.48 – 7.43 (m, 2H, 2x Ar***H***, OBz), 7.28 – 7.15 (m, 10H, 10x Ar***H***, OBn), 5.48 (dd, *J* = 9.0, 3.3 Hz, 1H, ***H-3***), 4.77 (d, *J* = 1.9 Hz, 1H, ***H-1***), 4.75 (d, *J* = 11.0 Hz, 1H, PhC***H$_a$***H$_b$, 4-OBn), 4.66 – 4.62 (m, 2H, PhC***H$_a$***H$_b$, 2-OBn; PhCH$_a$***H$_b$***, 4-OBn), 4.60 (d, *J* = 12.3 Hz, 1H, PhCH$_a$***H$_b$***, 2-OBn), 3.97 (dd, *J* = 3.4, 1.9 Hz, 1H, ***H-2***), 3.86 – 3.77 (m, 2H, ***H-4***, ***H-5***), 3.68 (dt, *J* = 9.8, 6.5 Hz, 1H, OC***H$_a$***H$_b$CH$_2$), 3.39 (dt, *J* = 9.8, 6.4 Hz, 1H, OCH$_a$***H$_b$***CH$_2$), 3.27 (t, *J* = 6.9 Hz, 2H, C***H$_2$***N$_3$), 1.65 – 1.55 (m, 4H, OCH$_2$C***H$_2$***, C***H$_2$***CH$_2$N$_3$), 1.47 – 1.39 (m, 2H, C***H$_2$***CH$_2$CH$_2$N$_3$), 1.37 (d, *J* = 5.8 Hz, 3H, 6-C***H$_3$***); **^{13}C NMR** (126 MHz, $CDCl_3$; solvent peak ref'd to 77.16): δ 165.82 (***C***=O, OBz), [138.04, 137.97 (Ph***C***CH$_2$, OBn)], 133.23 (OBz), 130.23 (Ar***C***C, OBz), 129.85 (OBz), [128.59, 128.46, 128.42, 128.08, 127.91, 127.86, 127.85 (OBz; OBn)], 98.03 (***C-1***), 79.33 (***C-4***), 76.51 (***C-2***), 75.33 (Ph***C***H$_2$, 4-OBn), 74.63 (***C-3***), 73.32 (Ph***C***H$_2$, 2-OBn), 67.92 (***C-5***), 67.48 (O***C***H$_2$CH$_2$), 51.42 (***C***H$_2$N$_3$), 29.11 (OCH$_2$***C***H$_2$), 28.77 (***C***H$_2$CH$_2$N$_3$), 23.56 (***C***H$_2$CH$_2$CH$_2$N$_3$), 18.19 (***C-6***); **HR-ESI-TOF/MS (m/z):** $[M+Na]^+$ calcd. for $C_{32}H_{37}N_3O_6Na$, 582.2575; found, 582.2567.

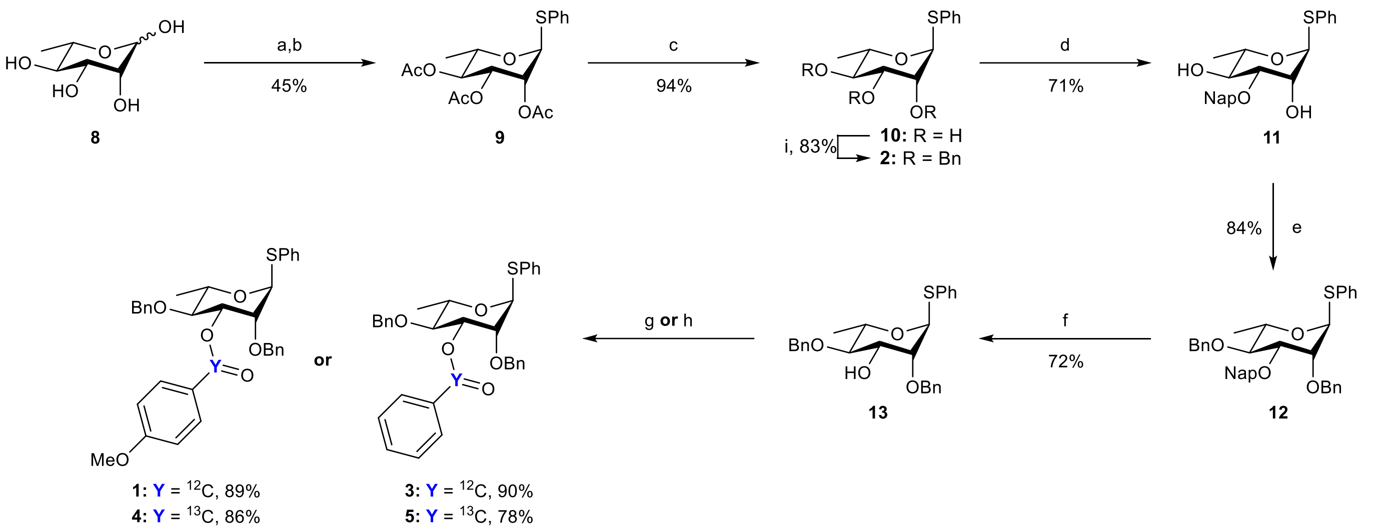

Supplementary Figure 19. Rhamnosyl donor **1** - **5** synthesis. [a] Ac_2O, Pyr, DCM; [b] HSPh, $BF_3{\cdot}OEt_2$, DCM; [c] K_2CO_3, MeOH; [d] i) Bu_2SnO, Tol; ii) NapBr, CsF, DMF; [e] BnBr, NaH, DMF; [f] DDQ, DCM, H_2O; [g] BzOH, CDI, DBU, ACN; [h] BzOH or *p*-AnisOH, EDCI·HCl, DMAP, DCM; [i] BnBr, NaH, DMF.

Phenyl 2,3,4-tri-*O*-acetyl-1-thio-α-L-rhamnopyranoside (9)

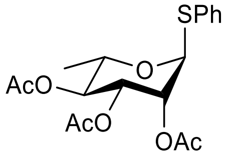

9

L-rhamnose (34 g, 0.21 mol, 1.0 eq) was suspended in DCM:Pyr (250 mL, 9:1 v/v). DMAP (1.3 g, 10 mmol, 0.050 eq) was added, after which the mixture was cooled down to 0°C. Ac_2O (98 mL, 1.0 mol, 4.8 eq) was added. The reaction was stirred at 0°C – R.T. for 16 hrs. The solution was cooled down to 0 °C. H_2O (200 mL) was added, after which the mixture was stirred for an additional 30 min. The mixture was extracted with $CHCl_3$ (4 x 150 mL). The combined organic layers were washed with 1.0 M aq. HCl (750 mL), water (750 mL), aq. $NaHCO_3$ (sat.) (750 mL) and brine (750 mL), respectively. The organic layer was dried with $MgSO_4$, filtered and evaporated *in vacuo.* The residue was dissolved in anh. DCM (430 mL). $BF_3{\cdot}OEt_2$ (64 mL, 0.52 mol, 2.5 eq) and thiophenol (43 mL, 0.42 mol, 2.0 eq) were added, after which the solution was stirred at R.T. for 2 days. The solution was neutralized with TEA. The solution was diluted with DCM (1.2 L) and was washed with aq. $NaHCO_3$ (sat.) (2 x 900 mL), 5% aq. NaOH (3 x 300 mL) and brine (900 mL), respectively. The organic layer was dried with $MgSO_4$, filtered and evaporated *in vacuo*. The residue was recrystallized from EtOH, yielding monosaccharide **8** as white crystals (36 g, 94 mmol, 45%).

TLC: (EtOAc:Hept, 60:40 v/v): R_f = 0.53; **^{1}H NMR** (500 MHz, $CDCl_3$): δ 7.47 (dd, *J* = 8.1, 1.5 Hz, 2H, 2x Ar***H***, SPh), 7.35 – 7.28 (m, 3H, 3x Ar***H***, SPh), 5.50 (dd, J = 3.4, 1.6 Hz, 1H, ***H-2***), 5.41 (d, *J* = 1.3 Hz, 1H, ***H-1***), 5.29 (dd, *J* = 10.1, 3.3 Hz, 1H, ***H-3***), 5.15 (t, *J* = 9.9 Hz, 1H, ***H-4***), 4.42 – 4.32 (m, 1H, ***H-5***), 2.15 (s, 3H, C***H₃***, OAc), 2.09 (s, 3H, C***H₃***, OAc), 2.02 (s, 3H, C***H₃***, OAc), 1.25 (d, *J* = 6.2 Hz, 3H, 6-C***H₃***); **^{13}C NMR** (126 MHz, $CDCl_3$): δ 170.06 (***C***=O, OAc), 170.04 (***C***=O, OAc), 169.97 (***C***=O, OAc), 133.23 (Ar***C***S, SPh), 131.80 (SPh), 129.18 (SPh), 127.88 (SPh), 85.66 (***C-1***), 71.27 (***C-2***), 71.08 (***C-4***), 69.34 (***C-3***), 67.71 (***C-5***), 20.95 (***C***H_3, OAc), 20.85 (***C***H_3, OAc), 20.73 (***C***H_3, OAc), 17.33 (***C-6***); **HR-ESI-TOF/MS (m/z):** $[M+Na]^+$ calcd for $C_{18}H_{22}O_7SNa$, 405.09839; found, 405.09735.

Phenyl 1-thio-α-L-rhamnopyranoside (10)

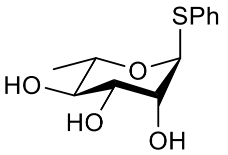

10

Thioglycoside **9** (36 g, 94 mmol, 1.0 eq) was dissolved in THF:MeOH (220 mL, 1:1 v/v). K_2CO_3 (2.7 g, 20 mmol, 0.21 eq) was added, after which the solution was stirred overnight. The mixture was filtered over a pad of silica. The silica was washed with THF. The filtrate was evaporated *in vacuo.* The residue was co-evaporated three times with DCM, yielding monosaccharide **10** as a white solid (24 g, 26 mmol, 94%).

TLC: (Acetone:DCM, 40:60 v/v): R_f = 0.23; **^{1}H NMR** (500 MHz, $CDCl_3$): δ 7.39 – 7.34 (m, 2H, 2x Ar***H***, SPh), 7.22 – 7.12 (m, 3H, 3x Ar***H***, SPh), 5.49 (s, 1H, ***H-1***), 4.45 (bs, 3H, 2-O***H***, 3-O***H***, 4-O***H***), 4.28 – 4.24 (m, 1H, ***H-2***), 4.20 – 4.12 (m, 1H, ***H-5***), 3.83 (dd, J = 9.8, 3.0 Hz, 1H, ***H-3***), 3.57 (t, J = 9.4 Hz, 1H, ***H-4***), 1.31 (d, J = 6.2 Hz, 3H, 6-C***H₃***); **^{13}C NMR**

(126 MHz, $CDCl_3$): δ 134.07 (Ar***C***S, SPh), 131.18 (SPh), 128.97 (SPh), 127.22 (SPh), 87.93 (***C-1***), 73.12 (***C-4***), 72.66 (***C-2***), 71.95 (***C-3***), 69.44 (***C-5***), 17.54 (***C-6***); **<u>HR-ESI-TOF/MS (m/z):</u>** $[M+Na]^+$ calcd for $C_{12}H_{16}O_4SNa$, 279.06670; found, 279.06489.

Phenyl 2,3,4-tri-*O*-benzyl-1-thio-α-L-rhamnopyranoside (2)

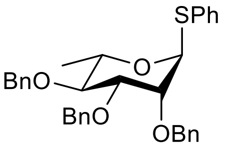

2

Thioglycoside **10** (70 mg, 0.27 mmol, 1.0 eq) was dissolved in anh. DMF (2.3 mL). The solution was cooled down to 0°C. NaH (69 mg, 60% wt, 1.7 mmol, 6.3 eq) was added, after which the mixture was stirred for 15 min. BnBr (0.20 mL, 1.7 mmol, 6.3 eq) was added. The solution was stirred at 0°C – R.T. for 2 hrs, after which the reaction was quenched with MeOH. The solution was diluted with H_2O and extracted with Et_2O (4 x 10 mL). The combined organic layers were washed with ice-cold brine (3 x 50 mL), dried over $MgSO_4$ and evaporated *in vacuo*. The residue was purified using silica-flash column chromatography (10 – 60% EtOAc in Hept), yielding monosaccharide **2** as a colourless oil (0.12 g, 0.27 mmol, 83%).

<u>TLC:</u> (EtOAc:Hept, 60:40 v/v): $R_f = 0.57$; **<u>^{1}H NMR</u>** (500 MHz, $CDCl_3$): δ 7.38 (dd, *J* = 8.2, 1.5 Hz, 2H, 2x Ar***H***, SPh) 7.36 – 7.21 (m, 18H, 15x Ar***H***, OBn; 3x Ar***H***, SPh), 5.49 (d, *J* = 1.7 Hz, 1H, ***H-1***), 4.96 (d, *J* = 10.8 Hz, 1H, PhC***H$_a$***H_b, 4-OBn), 4.71 (d, *J* = 12.4 Hz, 1H, PhC***H$_a$***H_b, 2-OBn), 4.67 – 4.57 (m, 4H, PhCH$_a$***H$_b$***, 2-OBn; PhC***H$_2$***, 3-OBn; PhCH$_a$***H$_b$***, 4-OBn), 4.14 (dq, *J* = 9.5, 6.2 Hz, 1H, ***C-5***), 3.99 (dd, *J* = 3.1, 1.8 Hz, 1H, ***H-2***), 3.83 (dd, *J* = 9.3, 3.0 Hz, 1H, ***H-3***), 3.68 (t, *J* = 9.4 Hz, 1H, ***C-4***), 1.35 (d, *J* = 6.2 Hz, 3H, 6-C***H$_3$***); **<u>^{13}C NMR</u>** (126 MHz, $CDCl_3$): δ 138.55 (Ar***C***CH_2, OBn), 138.25 (Ar***C***CH_2, OBn), 137.92 (Ar***C***CH_2, OBn), 134.72 (Ar***C***S, SPh), 131.34 (SPh), [129.01, 128.42, 128.40, 128.37, 128.02, 128.00, 127.82, 127.77, 127.75, 127.69, 127.67, 127.26 (SPh; OBn)], 85.92 (***C-1***), 80.52 (***C-4***), 80.04 (***C-3***), 76.57 (***C-2***), 75.45 (Ph***C***H_2, 4-OBn), 72.15 (Ph***C***H_2, 2-OBn), 72.12 (Ph***C***H_2, 3-OBn), 69.34 (***C-5***), 17.92 (***C-6***); **<u>HR-ESI-TOF/MS (m/z):</u>** $[M+Na]^+$ calcd for $C_{33}H_{34}O_4SNa$, 549.20755; found, 549.20666.

Phenyl 3-*O*-(naphthalene-2-ylmethyl)-1-thio-α-L-rhamnopyranoside (11)

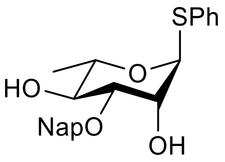

11

A suspension of thioglycoside **10** (3.89 g, 15.2 mmol, 1.0 eq) and Bu_2SnO (4.15 g, 16.7 mmol, 1.1 eq) in toluene (75 mL) was refluxed in a Dean-Stark apparatus for 3 hrs. The mixture was cooled to R.T., after which the solvent was evaporated *in vacuo*. The residue was dissolved in anh. DMF (75 mL). CsF (4.60 g, 30.3 mmol, 2.0 eq) and NapBr (3.67 g, 16.7 mmol, 1.1 eq) were added. The mixture was stirred for 16 hrs, after which it was diluted with EtOAc (200 mL). The solution was washed with aq. $NaHCO_3$ (sat.) (2x 100 mL), and brine (2x 100 mL), respectively. The combined aqueous layers were extracted with EtOAc. The combined organic layers were dried with Na_2SO_4, filtered over a pad of Celite and evaporated *in vacuo*. The residue was purified using silica-flash column chromatography (50% EtOAc in Hept), yielding monosaccharide **11** as a white solid (4.27 g, 10.8 mmol, 71%).

<u>TLC:</u> (EtOAc:Hept, 50:50 v/v): $R_f = 0.32$; **<u>^{1}H NMR</u>** (500 MHz, $CDCl_3$): δ 7.88 – 7.81 (m, 4H, Ar***H***, ONap), 7.52 – 7.47 (m, 3H, Ar***H***, ONap), 7.45 – 7.41 (m, 2H, Ar***H***, SPh), 7.30 – 7.23 (m, 3H, Ar***H***, SPh), 5.53 (d, <u>J</u> = 1.5 Hz, 1H, ***H-1***), 4.88 (d, *J* = 11.7 Hz, 1H, PhC***H$_a$***H_b, ONap), 4.76 (d, *J* = 11.7 Hz, 1H, PhC***H$_a$***H_b, ONap), 4.29 (td, J = 2.8, 1.5 Hz, 1H, ***H-2***), 4.14 (dq, *J* = 9.0, 6.3 Hz, 1H, ***H-5***), 3.71 (dd, J = 9.3, 3.1 Hz, 1H, ***H-3***), 3.66 (td, *J* = 9.2, 2.9 Hz, 1H, ***H-4***), 2.62 (d, *J* = 2.6 Hz, 1H, 2-O***H***), 2.27 (d, *J* = 2.9 Hz, 1H, 4-O***H***), 1.31 (d, *J* = 6.2 Hz, 3H, 6-C***H$_3$***); **<u>^{13}C NMR</u>** (126 MHz, $CDCl_3$): δ 134.74 (Ar***C***CH_2, ONap), 134.04 (Ar***C***S, SPh), 133.27 (Ar***C***C_2, ONap), 133.19 (Ar***C***C_2, ONap), 131.40 (SPh), 129.06 (SPh), [128.75, 127.98, 127.80 (ONap)], 127.41 (SPh), [127.07, 126.45, 126.33, 125.66 (ONap)], 87.33 (***C-1***), 79.89 (***C-3***), 71.98 (Ph***C***H_2, ONap), 71.90 (***C-4***), 69.50 (***C-2***), 69.16 (***C-5***), 17.55 (***C-6***); **<u>HR-ESI-TOF/MS (m/z):</u>** $[M+Na]^+$ calcd for $C_{23}H_{24}O_4SNa$, 419.12930; found, 419.12756.

Phenyl 2,4-*O*-di-benzyl-3-*O*-(naphthalene-2-ylmethyl)-1-thio-α-L-rhamnopyranoside (12)

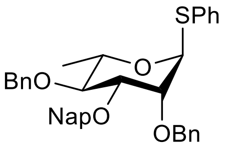

12

Thioglycoside **11** (9.84 g, 24.8 mmol, 1.0 eq) was dissolved in anh. DMF (35 mL). The solution was cooled down to 0°C. NaH (4.20 g, 60% Wt, 104 mmol, 4.2 eq) was added. The mixture was stirred for 25 min, after which BnBr (17.7 mL, 149 mmol, 6.0 eq) was added. The mixture was stirred for 16 hrs, after which the reaction was quenched with MeOH and diluted with H_2O. The solution was extracted with Et_2O (3x 300 mL). The combined organic layers were washed with NH_4Cl (sat.) (600 mL) and brine (500 mL), respectively. The organic layer was dried with $MgSO_4$, filtered and evaporated *in vacuo*. The residue was purified using silica-flash column chromatography (0-40% EtOAc in Hept), yielding monosaccharide **12** as a pale yellow oil (12.0 g, 20.8 mmol, 84%).

<u>TLC:</u> (EtOAc:Hept, 20:80 v/v): $R_f = 0.48$; **<u>^{1}H NMR</u>** (500 MHz, $CDCl_3$): δ 7.81 – 7.76 (m, 3H, 3x ArH, ONap), 7.75 – 7.70 (m, 1H, Ar***H***, ONap), 7.46 – 7.31 (m, 3H, 3x Ar***H***, ONap), 7.37 – 7.18 (m, 15H, 5x Ar***H***, SPh; 10x Ar***H***, OBn), 5.51 (d, *J* = 1.7 Hz, 1H, ***H-1***), 4.99 (d, *J* = 10.9 Hz, 1H, PhC***H$_a$***H_b, 4-OBn), 4.73 (d, *J* = 2.3 Hz, 2H, PhC***H$_2$***, ONap), 4.71 (d, *J* = 12.3 Hz, 1H, Ph***H$_a$***H_b, 2-OBn), 4.67 (d, *J* = 10.9 Hz, 1H, PhH_a***H$_b$***, 4-OBn), 4.63 (d, *J* = 12.3 Hz, 1H, PhH_a***H$_b$***, 2-OBn), 4.17 (dq, *J* = 9.4, 6.1 Hz, 1H, ***H-5***), 4.01 (dd, *J* = 3.1, 1.8 Hz, 1H, ***H-2***),

3.91 (dd, *J* = 9.3, 3.1 Hz, 1H, ***H-3***), 3.72 (t, *J* = 9.3 Hz, 1H, ***H-4***), 1.36 (d, *J* = 6.2 Hz, 3H, 6-C***H₃***); **^{13}C NMR** (126 MHz, $CDCl_3$): δ 138.56 (Ar***C***CH_2, 4-OBn), 137.89 (Ar***C***CH_2, 2-OBn), 135.71 (Ar***C***CH_2, ONap), 134.64 (Ar***C***S, SPh), 133.29 (Ar***C***C_2, ONap), 132.97 (Ar***C***C_2, ONap), 131.32 (SPh), [128.98, 128.38, 128.35, 128.12, 127.95, 127.92, 127.74, 127.67, 127.62, 127.24 (SPh; ONap; OBn)], [126.47, 126.07, 125.87, 125.83 (ONap)], 85.77 (***C-1***), 80.48 (***C-4***), 80.05 (***C-3***), 76.63 (***C-2***), 75.40 (Ph***C***H_2, 4-OBn), 72.13 (Ph***C***H_2, 2-OBn; Ar***C***H_2, ONap), 69.39 (***C-5***), 17.93 (***C-6***); **HR-ESI-TOF/MS (m/z):** $[M+Na]^+$ calcd for $C_{37}H_{36}O_4SNa$, 599.22320; found, 599.22145.

Phenyl 2,4-*O*-di-benzyl-1-thio-α-L-rhamnopyranoside (13)

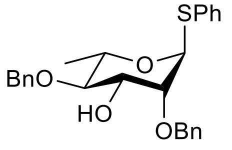

13

Thioglycoside **12** (21.3 g, 38.9 mmol, 1.0 eq) dissolved in DCM:H_2O (500 mL, 9:1 v/v). DDQ (12.6 g, 55.3 mmol, 1.5 eq) was added. The solution was vigorously stirred under exclusion of light for 90 min, after which DCM (1.0 L) and DDQ mixture (1.0 L) were added. The organic layer was extracted and washed with DDQ mixture (4x 1.0 L). The organic layer was dried with Na_2SO_4, filtered and evaporated *in vacuo*. The residue was purified using silica-flash column chromatography (0 – 40% EtOAc in Hept), yielding monosaccharide **13** as a pale yellow oil (12.7 g, 29.1 mmol, 72%).

TLC: (EtOAc:Hept, 20:80 v/v): R_f = 0.27; **^{1}H NMR** (500 MHz, $CDCl_3$): δ 7.44 – 7.41 (m, 2H, 2x Ar***H***, SPh), 7.39 – 7.24 (m, 13H, 3x Ar***H***, SPh; 10x Ar***H***, OBn), 5.55 (d, *J* = 1.4 Hz, 1H, ***H-1***), 4.92 (d, *J* = 11.0 Hz, 1H, PhC$\boldsymbol{H_a}H_b$, 4-OBn), 4.75 (d, *J* = 11.7 Hz, 1H, PhC$\boldsymbol{H_a}H_b$, 2-OBn), 4.67 (d, *J* = 11.0 Hz, 1H, PhC$H_a\boldsymbol{H_b}$, 4-OBn), 4.54 (d, *J* = 11.7 Hz, 1H, PhC$H_a\boldsymbol{H_b}$, 2-OBn), 4.16 (dq, *J* = 9.3, 6.2 Hz, 1H, ***H-5***), 4.00 (dd, *J* = 3.7, 1.4 Hz, 1H, ***H-2***), 3.96 (td, *J* = 9.1, 3.7 Hz, 1H, ***H-3***), 3.40 (t, J = 9.2 Hz, 1H, ***C-4***), 2.37 (d, J = 9.1 Hz, 1H, 3-O***H***), 1.34 (d, J = 6.3 Hz, 3H, 6-C***H₃***); **^{13}C NMR** (126 MHz, $CDCl_3$): δ 138.43 (Ar***C***CH_2, 4-OBn), 137.35 (Ar***C***CH_2, 2-OBn), 134.42 (Ar***C***S, SPh), 131.53 (SPh), [129.06, 128.64, 128.45, 128.17, 128.05, 127.97, 127.78, 127.40 (SPh; OBn)], 84.98 (***C-1***), 82.44 (***C-4***), 80.02 (***C-2***), 75.17 (Ph***C***H_2, 4-OBn), 72.40 (Ph***C***H_2, 2-OBn), 72.09 (***C-3***), 68.58 (***C-5***), 17.93 (***C-6***); **HR-ESI-TOF/MS (m/z):** $[M+Na]^+$ calcd for $C_{26}H_{28}O_4SNa$ 459.16060; found, 459.15976.

Phenyl 3-*O*-*p*-anisoyl-2,4-di-*O*-benzyl-1-thio-α-L-rhamnopyranoside (1)

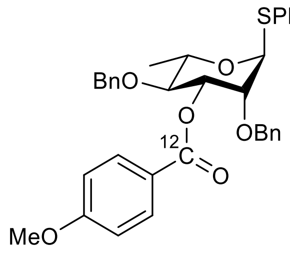

1

Thioglycoside **13** (11.6 g, 26.6 mmol, 1.0 eq) was added to a solution of EDCI-HCl (15.3 g, 79.8 mmol, 3.0 eq), *p*-methoxybenzoic acid (6.07 g, 39.9 mmol, 1.5 eq) and DMAP (650 mg, 5.32 mmol, 0.20 eq) in DCM (270 mL). The mixture was stirred for 16 hrs, after which additional DMAP (960 mg, 7.86 mmol, 0.30 eq) was added and refluxed for 6 hrs. The mixture was cooled to R.T. and diluted with DCM (250 mL). The solution was washed with H_2O (2 x 500 mL) and brine (800 mL), respectively. The organic layer was dried with $MgSO_4$, filtered and concentrated *in vacuo*. The residue was purified using silica-flash column chromatography (10% EtOAc in Hept), yielding monosaccharide **1** as a yellow oil (13.6 g, 23.8 mmol, 89.4 %).

TLC: (EtOAc:Tol, 6:94 v/v): R_f = 0.50; **^{1}H NMR** (500 MHz, $CDCl_3$): δ 8.04 – 8.01 (m, 2H, 2x Ar***H***, OAnis), 7.48 – 7.44 (m, 2H, 2x Ar***H***, SPh), 7.32 – 7.13 (m, 13H, 3x Ar***H***, SPh; 10x Ar***H***, OBn), 6.96 – 6.91 (m, 2H, 2x Ar***H***, OAnis), 5.52 (d, J = 1.8 Hz, 1H, ***H-1***), 5.44 (dd, *J* = 9.5, 3.3 Hz, 1H, ***H-3***), 4.79 (d, *J* = 10.9 Hz, 1H, Ph$\boldsymbol{H_a}H_b$, 4-OBn) 4.66 – 4.63 (m, 2H, Ph$\boldsymbol{H_a}H_b$, 2-OBn; Ph$H_a\boldsymbol{H_b}$, 4-OBn), 4.52 (d, *J* = 12.1 Hz, 1H, Ph$H_a\boldsymbol{H_b}$, 2-OBn) 4.30 (dq, *J* = 9.3, 6.1 Hz, 1H, ***H-5***), 4.23 (dd, J = 3.3, 1.9 Hz, 1H, ***H-2***), 3.89 – 3.84 (m, 4H, H-4, -OC***H₃***, OAnis) 1.38 (d, J = 6.2 Hz, 3H, 6-C***H₃***); **^{13}C NMR** (126 MHz, $CDCl_3$): δ 165.35 (***C***=O, OAnis), 163.58 (Ar***C***OMe), 137.94 (Ar***C***CH_2, OBn), 137.53 (Ar***C***CH_2, OBn), 134.57 (Ar***C***S, SPh), 131.82 (OAnis), 131.51 (SPh), 129.02 (SPh), [128.35, 128.32, 127.94, 127.82, 127.77, 127.75 (OBn)], 127.31 (SPh), 122.36 (Ar***C***CO_2, OAnis), 113.75 (OAnis), 85.60 (***C-1***), 79.18 (***C-4***), 77.67 (***C-2***), 75.15 (Ph***C***H_2, 4-OBn), 74.11 (***C-3***), 72.59 (Ph***C***H_2, 2-OBn), 69.15 (***C-5***), 55.48 (O***C***H_3), 17.99 (***C-6***); **HR-ESI-TOF/MS (m/z):** $[M+Na]^+$ calcd for $C_{34}H_{34}O_6SNa$, 593.19738; found, 593.19603.

Phenyl 3-*O*-*p*-anisoyl-2,4-di-*O*-benzyl-1-thio-α-L-rhamnopyranoside (3)

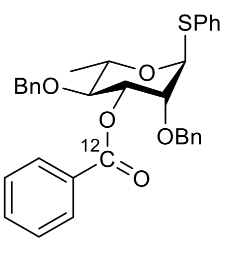

3

A mixture of benzoic acid (0.23 g, 1.9 mmol, 2.1 eq.) and CDI (0.33 g, 2.0 mmol, 2.2 eq) in anh. ACN (20 mL) was stirred vigorously at 65°C for 2 hrs. The solution was cooled down to R.T., after which a solution of thioglycoside **13** (0.40 g, 0.92 mmol, 1.0 eq) and DBU (0.29 g, 1.9 mmol, 1.0 eq) in anh. ACN (4.0 mL) was added. The mixture was heated to 60°C and stirred for 16 hrs. The mixture was cooled down to R.T. and subsequently poured into aq. $NaHCO_3$ (sat.) (50 mL). The mixture was extracted with DCM (3x 20 mL). The combined organic layers were washed with brine. The organic layer was dried with $MgSO_4$, filtered and concentrated *in vacuo*. The residue was purified using silica-flash column chromatography (20% EtOAc in Hept), yielding monosaccharide **3** as a white syrup (0.45 g, 0.82 mmol, 90%).

TLC: (EtOAc:Hept, 20:80 v/v): R_f = 0.44; **^{1}H NMR** (500 MHz, $CDCl_3$): δ 8.12 – 7.99 (m, 2H, 2x Ar***H***, OBz), 7.63 – 7.55 (m, 1H, Ar***H***, OBz), 7.50 – 7.43 (m, 4H, 2x Ar***H***, OBz; 2x Ar***H***, SPh), 7.33 – 7.28 (m, 2H, 2x Ar***H***, SPh), 7.28 – 7.11 (m, 11H, Ar***H***, SPh; 10x Ar***H***, OBn), 5.53 (d, *J* = 1.9 Hz, 1H, ***H-1***), 5.46 (dd, *J* = 9.4, 3.3 Hz, 1H, ***H-3***), 4.79 (d, *J* = 10.9 Hz, 1H, Ph$\boldsymbol{H_a}H_b$, 4-OBn), 4.70 – 4.64 (m, 2H, Ph$\boldsymbol{H_a}H_b$, 2-OBn; Ph$H_a\boldsymbol{H_b}$, 4-OBn), 4.51 (d, *J* = 12.2 Hz, 1H, Ph$H_a\boldsymbol{H_b}$, 2-OBn), 4.30 (dq, *J* = 9.4, 6.2 Hz, 1H, ***H-5***), 4.24 (dd, *J* = 3.3, 1.9 Hz, 1H, ***H-2***), 3.88 (t, *J* = 9.4 Hz, 1H, ***H-4***), 1.39 (d, *J* = 6.1 Hz, 3H, 6-C$\boldsymbol{H_3}$); **^{13}C NMR** (126 MHz, $CDCl_3$): δ 165.64 (***C***=O, OBz), 137.89 (Ar***C***CH_2, 4-OBn), 137.44 (Ar***C***CH_2, 2-OBn), 134.52 (Ar***C***S, SPh), 133.20 (OBz), 131.55 (SPh), 129.96 (Ar***C***CH_2, OBz), 129.78 (OBz), 129.05 (SPh), 128.48 (OBz), 128.37 (OBn), 128.34 (OBn), 127.92 (4-OBn), 127.86 (2-OBn), 127.81 (OBn), 127.79 (OBn), 127.37 (SPh), 85.52 (***C-1***), 79.16 (***C-4***), 77.54 (***C-2***), 75.21 (Ph$H_a\boldsymbol{H_b}$, 4-OBn), 74.39 (***C-3***), 72.53 (Ph$H_a\boldsymbol{H_b}$, 2-OBn), 69.17 (***C-5***), 18.00 (***C-6***); **HR-ESI-TOF/MS (m/z):** $[M+Na]^+$ calcd for $C_{33}H_{32}O_5SNa$, 563.18681; found, 563.18416.

Phenyl 3-*O*-*p*-anisoyl*-2,4-di-*O*-benzyl-1-thio-α-L-rhamnopyranoside (4)

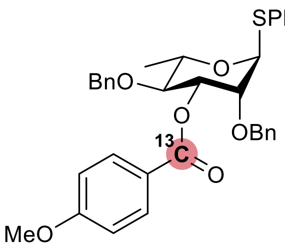

4

Thioglycoside **13** (25 mg, 57 µmol, 1.0 eq) was added to a solution of EDCI-HCl (33 mg, 0.17 mmol, 3 eq), $^{13}C_{=O}$-*p*-methoxybenzoic acid[14] (13 mg, 86 µmol, 1.5 eq) and DMAP (1.4 mg, 11 µmol, 0.20) in DCM (0.57 mL). The mixture was stirred for 72 hrs. The reaction mixture was diluted with DCM (10 mL). The solution was washed with H_2O (2x 4 mL) and brine (4 mL), respectively. The organic layer was dried with $MgSO_4$, filtered and concentrated *in vacuo*. The residue was purified using silica-flash column chromatography (10% EtOAc in Hept), yielding monosaccharide **4** as a yellow oil (28 mg, 57 µmol, 86%).

TLC: (EtOAc:Tol, 6:94 v/v): R_f = 0.50; **^{1}H NMR** (500 MHz, $CDCl_3$): δ 8.09 – 7.98 (m, 2H, 2x Ar***H***, OAnis), 7.49 – 7.42 (m, 2H, 2x Ar***H***, SPh), 7.36 – 7.11 (m, 13H, 3x Ar***H***, SPh; 10x Ar***H***, OBn), 7.02 – 6.88 (m, 2H, 2x Ar***H***, OAnis), 5.52 (d, *J* = 1.8 Hz, 1H, ***H-1***), 5.44 (dt, *J* = 9.5, 3.2 Hz, 1H. ***H-3***), 4.79 (d, *J* = 10.9 Hz, 1H, Ph$\boldsymbol{H_a}H_b$, 4-OBn), 4.71 – 4.59 (m, 2H, Ph$\boldsymbol{H_a}H_b$, 2-OBn; Ph$H_a\boldsymbol{H_b}$, 4-OBn), 4.52 (d, *J* = 12.2 Hz, 1H, Ph$H_a\boldsymbol{H_b}$, 2-OBn), 4.33 – 4.26 (m, 1H, ***H-5***), 4.23 (dd, *J* = 3.3, 1.9 Hz, 1H, ***H-2***), 3.94 – 3.80 (m, 4H, ***H-4***, -OC$\boldsymbol{H_3}$, OAnis), 1.38 (d, *J* = 6.2 Hz, 3H, 6-C$\boldsymbol{H_3}$); **^{13}C NMR** (126 MHz, $CDCl_3$): δ 165.38 (***C***=O, OAnis), 163.59 (Ar***C***OMe), 137.94 (Ar***C***CH_2, OBn), 137.53 (Ar***C***CH_2, OBn), 134.58 (Ar***C***S, SPh), 131.85 (OAnis), 131.52 (SPh), 129.04 (SPh), 128.37 (OBn), 128.34 (OBn), 127.96 (OBn), 127.84 (OBn), 127.79 (OBn), 127.77 (OBn), 122.37 (d, *J* = 80.5 Hz, Ar***C***CO_2, OAnis), 113.76 (d, *J* = 4.8 Hz, OAnis), 85.60 (***C-1***), 79.19 (***C-4***), 77.66 (***C-2***), 75.18 (Ph***C***H_2, 4-OBn), 74.10 (d, *J* = 2.5 Hz, ***C-3***), 72.57 (Ph***C***H_2, 2-OBn), 69.16 (***C-5***), 55.50 (O***C***H_3), 17.99 (***C-6***); **HR-ESI-TOF/MS (m/z):** $[M+Na]^+$ calcd for $C_{33}{}^{13}CH_{34}O_6SNa$, 594.20073, found, 594.19811.

Phenyl 3-*O*- benzoyl*-2,4-di-*O*-benzyl-1-thio-α-L-rhamnopyranoside (5)

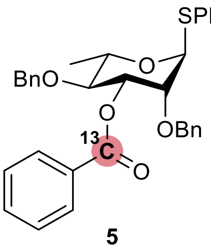

5

Thioglycoside **13** (25 mg, 57 µmol, 1.0 eq) was added to a solution of EDCI-HCl (33 mg, 0.17 mmol, 3.0 eq), $^{13}C_{=O}$- benzoic acid (10 mg, 86 µmol, 1.5 eq) and DMAP (1.4 mg, 11 µmol, 0.20 eq) in DCM (0.57 mL). The mixture was stirred for 48 hrs, after which it was diluted with DCM (10 mL). The solution was washed with H_2O (2 x 5 mL) and brine (5 mL), respectively. The organic layer was dried with $MgSO_4$, filtered and concentrated *in vacuo*. The residue was purified using silica-flash column chromatography (20% EtOAc in Hept), yielding monosaccharide **5** as a clear oil (24 mg, 44 µmol, 78 %).

TLC: (EtOAc:Tol, 6:94 v/v): R_f = 0.50; **^{1}H NMR** (500 MHz, $CDCl_3$): δ 8.09 – 8.02 (m, 2H, 2x Ar***H***, OBz), 7.63 – 7.56 (m, 1H, Ar***H***, OBz), 7.50 – 7.42 (m, 4H, 2x Ar***H***, OBz; 2x Ar***H***, SPh), 7.34 – 7.11 (m, 13H, 3x Ar***H***, SPh; 10x Ar***H***, OBn), 5.53 (d, *J* = 1.8 Hz, 1H, ***H-1***), 5.46 (dt, *J* = 9.5, 3.2 Hz, 1H, ***H-3***), 4.79 (d, *J* = 10.9 Hz, 1H, Ph$\boldsymbol{H_a}H_b$, 4-OBn), 4.74 – 4.61 (m, 2H, Ph$\boldsymbol{H_a}H_b$, 2-OBn; Ph$H_a\boldsymbol{H_b}$, 4-OBn), 4.51 (d, *J* = 12.1 Hz, 1H, Ph$H_a\boldsymbol{H_b}$, 2-OBn), 4.30 (dq, *J* = 9.3, 6.2 Hz, 1H, ***H-5***), 4.24 (dd, *J* = 3.3, 1.9 Hz, 1H, ***H-2***), 3.88 (t, *J* = 9.4 Hz, 1H, ***H-4***), 1.39 (d, *J* = 6.1 Hz, 3H, 6-C$\boldsymbol{H_3}$); **^{13}C NMR** (126 MHz, $CDCl_3$): δ 165.63 (***C***=O, OBz), 137.88 (Ar***C***CH_2, 4-OBn), 137.43 (Ar***C***CH_2, 2-OBn), 134.50 (Ar***C***S, SPh), 133.19 (OBz), 131.54 (SPh), 129.77 (d, *J* = 2.4 Hz, OBz), 129.76 (OBz), 129.05 (SPh), 128.50 (OBz), 128.46 (OBz), 128.36 (OBn), 128.33 (OBn), 127.92 (2-OBn), 127.86 (2-OBn), 127.81 (OBn), 127.78 (OBn), 127.37 (SPh), 85.52 (***C-1***), 77.53 (***C-2***), 75.20 (Ph$H_a\boldsymbol{H_b}$, 4-OBn), 74.38 (d, *J* = 2.7 Hz, ***C-3***), 72.53 (Ph$H_a\boldsymbol{H_b}$, 2-OBn), 69.17 (***C-5***), 17.99 (***C-6***); **HR-ESI-TOF/MS (m/z):** $[M+Na]^+$ calcd for $C_{32}{}^{13}CH_{32}O_5Na$, 564.19017; found, 564.18844.

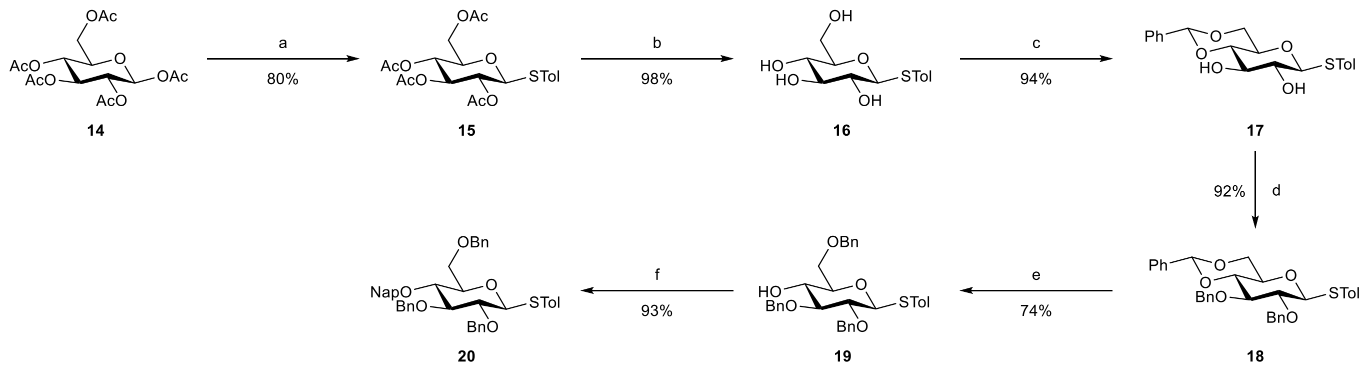

Supplementary Figure 20. Glucosyl donor **20** synthesis. [a] HSPh, $BF_3{\cdot}OEt_2$, DCM; [b] K_2CO_3, MeOH; [c] BDA, $NaHSO_4{\cdot}SiO_2$, ACN; [d] BnBr, NaH, DMF; [e] TES, TFA, DCM; [f] NapBr, NaH, DMF.

p-Tolyl 2,3,4,6-*O*-acetyl-1-thio-β-D-glucopyranoside (15)

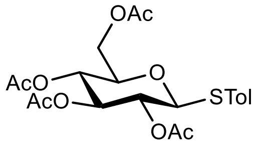

15

1,2,3,4,6-penta-O-acetyl-β-D-glucopyranoside (50.0 g, 128 mmol, 1.0 eq) and *p*-Toluenethiol (19.1 g, 154 mmol, 1.20 eq) were dissolved in anh. DCM (250 mL). The solution was cooled down to 0°C. $BF_3{\cdot}OEt_2$ (24.4 mL, 192 mmol, 1.5 eq) was carefully added. The mixture was allowed to warm up to R.T. and was stirred for 64 hours. The mixture was poured on ice, after which $NaHCO_3$ was added until no more formation of gas was observed. The solution was extracted with DCM. The organic layer was washed with aq. $NaHCO_3$ (sat.). The organic layer was dried with $MgSO_4$, filtered and evaporated *in vacuo*. The residue was recrystallized from EtOH, yielding monosaccharide **15** as a white solid (46.3 g, 102 mmol, 79.6%).

TLC: (EtOAc:Hept, 50:50 v/v): R_f = 0.50; **^{1}H NMR** (400 MHz, $CDCl_3$): δ 7.41 – 7.37 (m, 2H, 2x Ar***H***, STol), 7.15 – 7.11 (m, 2H, 2x Ar***H***, STol), 5.21 (t, *J* = 9.4 Hz, 1H, H-3), 5.02 (dd, *J* = 10.1, 9.5 Hz, 1H, ***H-4***), 4.93 (dd, *J* = 10.0, 9.2 Hz, 1H, ***H-2***), 4.64 (d, *J* = 10.1 Hz, 1H, ***H-1***), 4.24 – 4.15 (m, 2H, ***H-6a***, ***H-6b***), 3.70 (ddd, *J* = 10.1, 4.9, 2.7 Hz, 1H, ***H-5***), 2.35 (s, 3H, C***H3***, STol), 2.09 (s, 3H, C***H3***, OAc), 2.08 (s, 3H, C***H3***, OAc), 2.01 (s, 3H, C***H3***, OAc), 1.99 (s, 3H, C***H3***, OAc); **^{13}C NMR** (100 MHz, $CDCl_3$): δ 170.59 (***C***=O, OAc), 170.21 (***C***=O, OAc), 169.40 (***C***=O, OAc), 169.26 (***C***=O, OAc), 138.82 (Ar***C***CH3, STol), 133.85 (STol), 129.70 (STol), 127.56 (Ar***C***S, STol), 85.86 (***C-1***), 75.77 (***C-5***), 74.04 (***C-3***), 69.93 (***C-2***), 68.22 (***C-4***), 62.15 (***C-6***), 21.20 (-***C***H3, STol), 20.78 (***C***H3, OAc), 20.74 (***C***H3, OAc), 20.61 (***C***H3, OAc), 20.59 (***C***H3, OAc); **HR-ESI-TOF/MS (m/z):** $[M+Na]^+$ calcd. for $C_{21}H_{26}O_9SNa$, 477.11952; found, 477.11852.

p-Tolyl-1-thio-β-D-glucopyranoside (16)

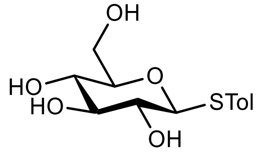

16

Thioglycoside **15** (46.3 g, 102 mmol, 1.0 eq) was dissolved in MeOH (1.00 L). K_2CO_3 (2.81 g, 4.87 mmol, 0.048 eq) was added. The solution was stirred for 16 hrs. DOWEX 50W X8(H^+) was added, after which the mixture was stirred for 30 min. The solution was filtered and evaporated *in vacuo* to yield monosaccharide **16** as a white solid (28.6 g, 99.9 mmol, 98.1%).

TLC: (MeOH:DCM, 20:80 v/v): R_f = 0.15; **^{1}H NMR** (500 MHz, MeOD-d4): δ 7.36 (d, *J* = 8.2 Hz, 2H, 2x Ar***H***, STol), 7.02 (d, *J* = 8.0 Hz, 2H, 2x Ar***H***, STol), 4.41 (d, *J* = 9.7 Hz, 1H, ***H-1***), 3.75 (dd, *J* = 12.2, 1.7 Hz, 1H, ***H-6a***), 3.56 (dd, *J* = 12.1, 5.2 Hz, 1H, ***H-6b***), 3.30 – 3.24 (m, 1H, H-3), 3.22 – 3.15 (m, 2H, ***H-4***, ***H-5***), 3.08 (dd, *J* = 9.7, 8.7 Hz, 1H, ***H-2***), 2.20 (s, 3H, C***H3***, STol); **^{13}C NMR** (126 MHz, MeOD-d4; solvent peak ref'd to 49.00): δ 138.72 (Ar***C***CH3, STol), 133.48 (STol), 131.11 (Ar***C***S, STol), 130.50 (STol), 89.59 (***C-1***), 81.94 (***C-4***), 79.60 (***C-3***), 73.63 (***C-2***), 71.32 (***C-5***), 62.85 (***C-6***), 21.10 (***C***H3, STol); **HR-ESI-TOF/MS (m/z):** $[M+Na]^+$ calcd. for $C_{13}H_{18}O_5SNa$, 309.07726; found, 309.07792.

p-Tolyl 4,6-*O*-benzylidene-1-thio-β-D-glucopyranoside (17)

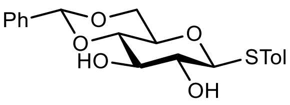

17

Thioglycoside **16** (28.0 g, 98.1 mmol, 1.0 eq), BDA (18.0 mL, 119.0 mmol, 1.22 eq) and $NaHSO_4{\cdot}SiO_2$ (5.01 g, 27.8 mmol, 0.28 eq) were added to anh. ACN (1.00 L). The solution was stirred for 90 min. The catalyst was removed via filtration and aq. $NaHCO_3$ (sat.) (100 mL) was added. The ACN was evaporated *in vacuo*. The aqueous layer was extracted with EtOAc (2x 250 mL). The solution was washed with brine (250 mL). The combined organic layers were dried with $MgSO_4$, filtered and

evaporated *in vacuo*. The residue was recrystallized from EtOH, yielding monosaccharide **17** as a white solid (34.5 g, 92.2 mmol, 93.9%).

<u>TLC:</u> (MeOH:DCM, 20:80 v/v): R_f = 0.63; **<u>^{1}H NMR</u>** (500 MHz, $CDCl_3$): δ 7.48 – 7.45 (m, 2H, 2x Ar***H***, $PhCHO_2$), 7.45 – 7.42 (m, 2H, 2x Ar***H***, STol), 7.38 – 7.34 (m, 3H, 3x Ar***H***, $PhCHO_2$), 7.15 (d, *J* = 7.9 Hz, 2H, 2x Ar***H***, STol), 5.51 (s, 1H, PhC***H***O_2), 4.55 (d, *J* = 9.7 Hz, 1H, ***H-1***), 4.39 – 4.34 (m, 1H, ***H-6***$_a$), 3.85 – 3.80 (m, 1H, C-3), 3.79 – 3.73 (m, 1H, ***H-6***$_b$), 3.51 – 3.46 (m, 2H, ***H-4***, ***H-5***), 3.42 (dd, *J* = 9.7, 8.5 Hz, 1H, ***H-2***) 2.80 (bs, 2H, 2-O***H***, 3-O***H***), 2.36 (s, 3H, PhC***H***$_3$, STol); **<u>^{13}C NMR</u>** (126 MHz, $CDCl_3$): δ 138.81 (Ar***C***CH_3, STol), 136.89 (Ar***C***C, $PhCHO_2$), 133.64 (STol), 129.88 (STol), 129.31 ($PhCHO_2$), 128.35 ($PhCHO_2$), 127.32 (Ar***C***S, STol), 126.30 ($PhCHO_2$), 101.89 (Ph***C***HO_2), 88.67 (***C-1***), 80.22 (***C-4***), 74.51 (***C-3***), 72.49 (***C-2***), 70.49 (***C-5***), 68.57 (***C-6***), 21.18 (***C***H_3, STol); **<u>HR-ESI-TOF/MS (m/z):</u>** $[M+Na]^+$ calcd. for $C_{20}H_{22}O_5SNa$, 397.10856; found, 397.10852.

_p_-Tolyl 2,3-di-_O_-benzyl-4,6-_O_-benzylidene-1-thio-β-D-glucopyranoside (18)

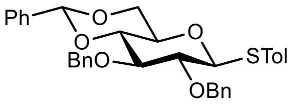

18

Thioglycoside **17** (10.4 g, 27.8 mmol, 1.0 eq) was dissolved in anh. DMF (200 mL). The solution was cooled down to 0°C. NaH (2.91 g, 60% Wt, 72.8 mmol, 2.6 eq) and BnBr (8.50 mL, 71.5 mmol, 2.6 eq) were added, respectively. The solution was stirred for 24 hrs, after which it was quenched with MeOH (10.0 mL) and stirred for an additional 15 min. The mixture was concentrated *in vacuo*. The residue was resuspended in $CHCl_3$ (250 mL) and subsequently washed with aq. $NaHCO_3$ (sat.) (250 mL). The organic layer was dried with $MgSO_4$, filtered and evaporated *in vacuo*. The residue was purified using silica-flash column chromatography (5 – 25% EtOAc in Hept), yielding monosaccharide **18** as a white solid (14.2 g, 25.6 mmol, 92.2%).

<u>TLC:</u> (EtOAc:Tol, 25:75 v/v): R_f = 0.40; **<u>^{1}H NMR</u>** (500 MHz, $CDCl_3$): δ 7.50 – 7.45 (m, 2H, 2x Ar***H***, $PhCHO_2$), 7.45 – 7.24 (m, 15H, 2x Ar***H***, STol; 3x Ar***H***, $PhCHO_2$; 10x Ar***H***, OBn), 7.11 (d, *J* = 7.9 Hz, 2H, 2x Ar***H***, STol), 5.58 (s, 1H, PhC***H***O_2), 4.93 (d, *J* = 11.2 Hz, 1H, PhC***H***$_a$H$_b$, 3-OBn), 4.87 (d, *J* = 10.3 Hz, 1H, PhC***H***$_a$H$_b$, 2-OBn), 4.81 (d, *J* = 10.3 Hz, 1H, PhCH$_a$***H***$_b$, 2-OBn), 4.77 (d, *J* = 11.2 Hz, 1H, PhCH$_a$***H***$_b$, 3-OBn), 4.69 (d, *J* = 9.8 Hz, 1H, ***H-1***), 4.38 (dd, *J* = 10.5, 5.0 Hz, 1H, ***H-6***$_a$), 3.86 – 3.76 (m, 2H, ***H-3***, ***H-6***$_b$), 3.68 (t, *J* = 9.4 Hz, 1H, ***H-4***), 3.52 – 3.41 (m, 2H, ***H-2***, ***H-5***), 2.34 (s, 3H, C***H***$_3$, STol); **<u>^{13}C NMR</u>** (126 MHz, $CDCl_3$): δ 138.31 (Ar***C***CH_2, 3-OBn), 138.20 (Ar***C***CH_3, STol), 138.10 (Ar***C***CH_2, 2-OBn), 137.27 (Ar***C***C, $PhCHO_2$), 133.04 (STol), 129.78 (STol), 129.14 (Ar***C***S, STol), 128.98 ($PhCHO_2$), [128.41, 128.40, 128.27, 128.22, 128.13, 127.87, 127.78 ($PhCHO_2$; STol; OBn)], 125.99 ($PhCHO_2$), 101.11 (Ph***C***HO_2), 88.51 (***C-1***), 83.06 (***C-3***), 81.48 (***C-4***), 80.40 (***C-2***), 75.85 (Ph***C***H_2, 2-OBn), 75.32 (Ph***C***H_2, 3-OBn), 70.20 (***C-5***), 68.72 (***C-6***), 21.14 (***C***H_3, STol); **<u>HR-ESI-TOF/MS (m/z):</u>** $[M+Na]^+$ calcd. $C_{34}H_{34}O_5SNa$, 577.20246; found 577.20214.

_p_-Tolyl 2,3,6-tri-_O_-benzyl-1-thio-β-D-glucopyranoside (19)

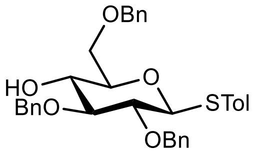

19

Thioglycoside **17** (6.0 g, 11 mmol, 1.0 eq) was dissolved in anh. DCM (60 mL). The solution was cooled down to 0°C. TES (8.6 mL, 54 mmol, 5.0 eq) and TFA (4.2 mL, 55 mmol, 5.0 eq) were added. The solution was stirred at 0°C – R.T. for 5 hrs. The solution was diluted with DCM (40 mL) and poured on ice. The mixture was washed with aq. $NaHCO_3$ (sat.) (3x 50 mL) and brine (50 mL), respectively. The organic layer was dried with $MgSO_4$, filtered and evaporated *in vacuo*. The residue was purified using silica-flash column chromatography (5 – 30% EtOAc in Hept), yielding monosaccharide **19** as a white solid (4.5 g, 8.0 mmol, 74%).

<u>TLC:</u> (EtOAc:Hept, 25:75 v/v): R_f = 0.22; **<u>^{1}H NMR</u>** (500 MHz, $CDCl_3$): δ 7.48 – 7.44 (m, 2H, 2x Ar***H***, STol), 7.43 – 7.40 (m, 2H, 2x Ar***H***, 2-OBn), 7.37 – 7.26 (m, 13H, 13x Ar***H***, OBn), 7.05 (d, *J* = 7.8 Hz, 2H, 2x Ar***H***, STol), 4.94 – 4.88 (m, 2H, PhC***H***$_a$H$_b$, 2-OBn; PhC***H***$_a$H$_b$, 3-OBn), 4.77 (d, *J* = 11.4 Hz, 1H, PhCH$_a$***H***$_b$, 3-OBn), 4.74 (d, *J* = 10.3 Hz, 1H PhCH$_a$***H***$_b$, 2-OBn), 4.62 (d, *J* = 9.6 Hz, 1H, ***H-1***), 4.60 – 4.53 (m, 2H, PhC***H***$_2$, 6-OBn), 3.76 (qd, *J* = 10.4, 4.6 Hz, 2H, 2x ***H-6***), 3.64 (td, *J* = 9.2, 2.2 Hz, 1H, ***H-4***), 3.52 (t, *J* = 8.7 Hz, 1H, ***H-3***), 3.48 – 3.42 (m, 2H, ***H-2***, ***H-5***), 2.55 (d, *J* = 2.3 Hz, 1H, 4-O***H***), 2.31 (s, 3H, C***H***$_3$, STol); **<u>^{13}C NMR</u>** (126 MHz, $CDCl_3$): δ 138.48 (Ar***C***CH_2, 3-OBn), 138.04 (Ar***C***CH_2, 2-OBn), 137.96 (Ar***C***CH_2, 6-OBn), 137.78 (Ar***C***CH_3, STol), 132.63 (STol), 129.79 (Ar***C***S, STol), 129.68 (STol), [128.62, 128.45, 128.42, 128.28, 127.96, 127.94, 127.90, 127.72, 127.71 (OBn)], 87.97 (***C-1***), 86.19 (***C-3***), 80.46 (***C-2***), 78.02 (***C-5***), 75.49 (Ph***C***H_2, 3-OBn), 75.35 (Ph***C***H_2, 2-OBn), 73.66 (Ph***C***H_2, 6-OBn), 71.77 (***C-4***), 70.44 (***C-6***), 21.11 (***C***H_3, STol); **<u>HR-ESI-TOF/MS (m/z):</u>** $[M+Na]^+$ calcd. for $C_{36}H_{36}O_5SNa$, 579.21811; found 579.21643.

p-Tolyl 2,3,6-tri-*O*-benzyl-4-*O*-(naphthalene-2-ylmethyl)-1-thio-β-D-glucopyranoside (20)

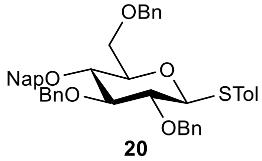

Thioglycoside **19** (4.22 g, 7.58 mmol, 1.0 eq) was dissolved in anh. DMF (70.0 mL). The solution was cooled down to 0°C. NaH (460 mg, 11.5 mmol, 60% Wt, 1.5 eq) and NapBr (2.51 g, 11.4 mmol, 1.5 eq) were added. The solution was stirred at 0°C – R.T. for 64 hrs. The solution was quenched with MeOH (5.0 mL) and diluted with EtOAc (300 mL). The solution was washed with H_2O (300 mL), aq. $NaHCO_3$ (sat.) (150 mL) and brine (150 mL), respectively. The organic layer was dried with Na_2SO_4, filtered and evaporated *in vacuo*. The residue was purified using silica-flash column chromatography (5 – 25% EtOAc in Hept) and recrystallized from EtOAc/Hept, yielding monosaccharide **20** as a white solid (4.89 g, 7.02 mmol, 92.6%).

TLC: (EtOAc:Hept, 25:75 v/v): R_f = 0.40; **^{1}H NMR** (500 MHz, $CDCl_3$): δ 7.82 – 7.78 (m, 1H, Ar***H***, ONap), 7.78 – 7.70 (m, 2H, 2x Ar***H***, ONap), 7.61 (s, 1H, Ar***H***, ONap), 7.49 (d, *J* = 8.2 Hz, 2x Ar***H***, STol), 7.47 – 7.44 (m, 2H, 2x Ar***H***, ONap), 7.43 – 7.39 (m, 2H, 2x Ar***H***, 2-OBn), 7.36 – 7.24 (m, 14H, 13x Ar***H***, OBn; Ar***H***, ONap), 7.03 (d, *J* = 7.9 Hz, 2H, 2x Ar***H***, STol), 4.96 (d, *J* = 11.1 Hz, 1H, ArC$\boldsymbol{H_a}$H$_b$, ONap), 4.93 – 4.89 (m, 2H, PhC$\boldsymbol{H_a}$H$_b$, 2-OBn; PhC$\boldsymbol{H_a}$H$_b$, 3-OBn), 4.86 (d, *J* = 10.9 Hz, 1H, PhCH$_a\boldsymbol{H_b}$, 3-OBn), 4.76 – 4.72 (m, 2H, PhCH$_a\boldsymbol{H_b}$, 2-OBn; ArCH$_a\boldsymbol{H_b}$, ONap), 4.63 – 4.57 (m, 2H, ***H-1***, PhC$\boldsymbol{H_a}$H$_b$, 6-OBn), 4.50 (d, *J* = 12.0 Hz, 1H, PhCH$_a\boldsymbol{H_b}$, 6-OBn), 3.80 (dd, *J* = 10.9, 2.0 Hz, 1H, ***H-6$_a$***), 3.77 – 3.67 (m, 3H, ***H-3***, ***H-4***, ***H-6$_b$***), 3.53 – 3.47 (m, 2H, ***H-2***, ***H-5***), 2.30 (s, 3H, C$\boldsymbol{H_3}$, STol); **^{13}C NMR** (126 MHz, $CDCl_3$, solvent peak ref'd to 77.16): δ 138.60 (Ar***C***CH$_2$, 3-OBn), 138.48 (Ar***C***CH$_2$, 6-OBn), 138.27 (Ar***C***CH$_2$, 2-OBn), 137.84 (Ar***C***CH$_3$, STol), 135.69 (Ar***C***CH$_2$, ONap), 133.39 (Ar***C***C$_2$, ONap), 133.12 (Ar***C***C$_2$, ONap), 132.86 (STol), 129.93 (Ar***C***S, STol), 129.80 (STol), [128.60, 128.57, 128.48, 128.38, 128.30, 128.06, 127.99, 127.93, 127.83, 127.80, 127.68 (STol; ONap; OBn)], [126.78, 126.22, 126.07 (ONap)], 87.83 (***C-1***), 86.96 (***C-3***), 80.98 (***C-2***), 79.24 (***C-5***), 77.96 (***C-4***), 75.97 (Ph***C***H$_2$, 3-OBn), 75.52 (Ph***C***H$_2$, 2-OBn), 75.22 (Ar***C***H$_2$, ONap), 73.58 (Ph***C***H$_2$, 6-OBn), 69.22 (***C-6***), 21.25 (***C***H$_3$, STol); **HR-ESI-TOF/MS (m/z):** [M+Na]$^+$ calcd for $C_{45}H_{44}O_5SNa$, 719.28071; found 719.27981.

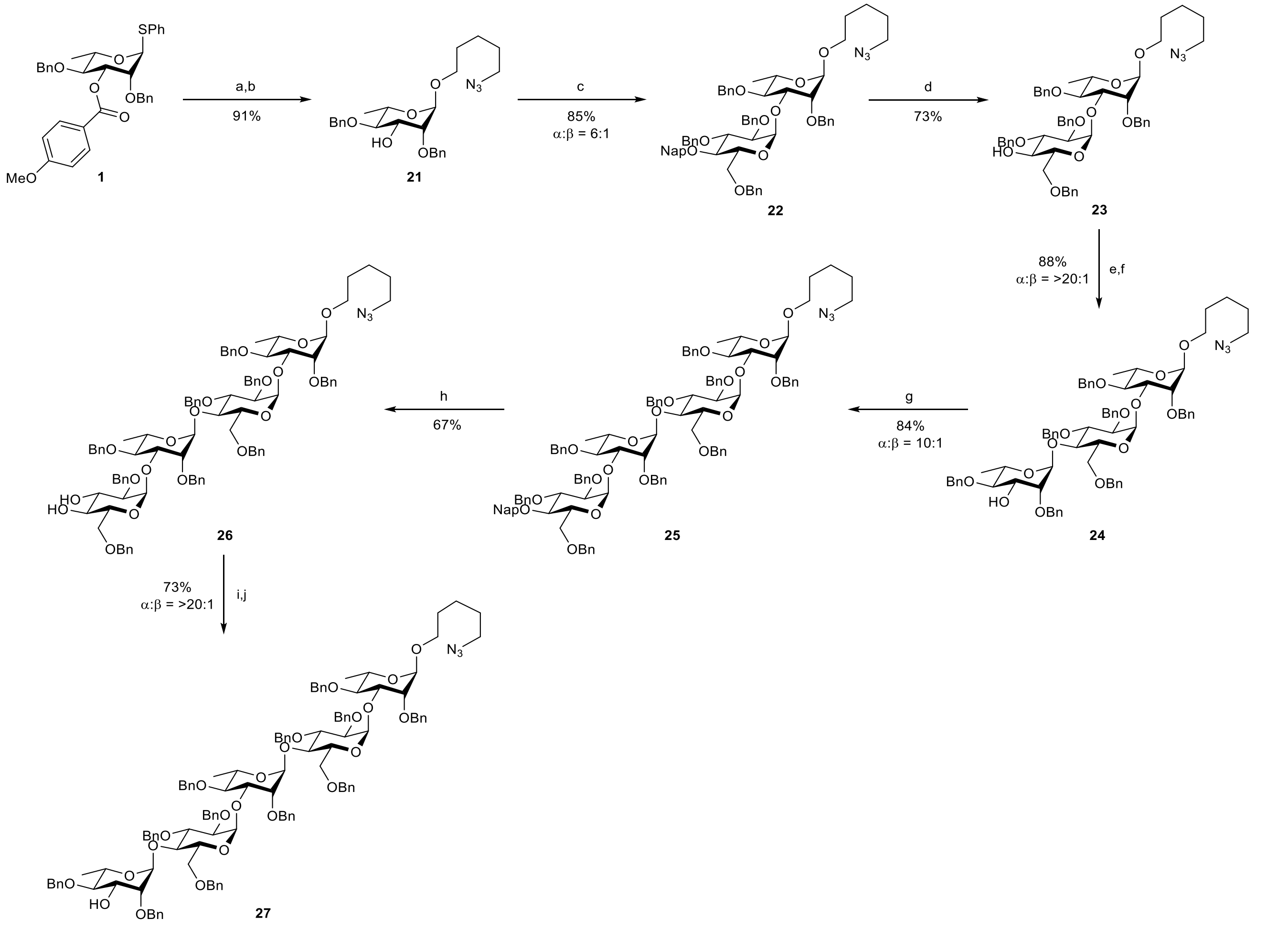

Supplementary Figure 21. Oligosaccharide **27** synthesis. [a] 5-azidopentanol, NIS, TfOH, DCM; [b] NaOMe, MeOH; [c] **20**, NIS, TMSOTf, DMF, DCM; [d] DDQ, DCM, H_2O; [e] **1**, NIS, TfOH, DCM; [f] NaOMe, MeOH, THF; [g] **20**, NIS, TMSOTf, DMF, DCM; [h] DDQ, DCM, H_2O; [i] **1**, NIS, TfOH, DCM; [j] NaOMe, MeOH, THF.

5-Azidopentyl 2,4-di-*O*-benzyl-α-D-rhamnopyranoside (21)

Thioglycoside **1** (3.1 g, 5.3 mmol, 1.0 eq) was dissolved in anh. DCM (75 mL). 5-Azidopentanol (0.85 mL, 6.5 mmol, 1.2 eq) was added. The solution was cooled down to 0°C. Molecular sieves (4Å) were added, after which the solution was stirred for 90 min. The solution was cooled down to -78°C. NIS (1.2 g, 5.4 mmol, 1.0 eq) and TfOH (24 µL, 0.27 mmol, 0.051 eq) were added, respectively. The solution was stirred at -78°C to 0°C for 30 min, turning bright red over time. The solution was quenched with TEA (3.0 mL). The mixture was filtered over celite, after which the filtrate was washed with 10% aq. $Na_2S_2O_3$. The organic layer was dried with $MgSO_4$, filtered and evaporated *in vacuo*. The residue was dissolved in anh. MeOH (75 mL). 2.0 molar NaOMe in MeOH (3.0 mL, 5.3 mmol, 1.0 eq) was added. The solution was stirred at R.T. for 64 hrs. DOWEX 50W X8(H^+) was added, after which the mixture was stirred for an additional 30 min. The solution was filtered and evaporated *in vacuo*. The residue was purified using silica-flash column chromatography (0 – 10% EtOAc in Tol), yielding monosaccharide **21** as a colourless oil (2.2 g, 4.9 mmol, 91%).

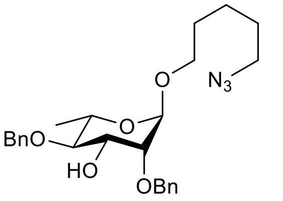

21

TLC: (EtOAc:Tol, 6:94 v/v): R_f = 0.24. **^{1}H NMR** (500 MHz, $CDCl_3$): δ 7.39 – 7.26 (m, 10H, 10x Ar***H***, OBn), 4.90 (d, *J* = 11.1 Hz, 1H, PhC***H$_a$***H$_b$, 4-OBn), 4.78 (d, *J* = 1.7 Hz, 1H, ***H-1***), 4.74 (d, *J* = 11.8 Hz, 1H, PhC***H$_a$***H$_b$, 2-OBn), 4.66 (d, *J* = 11.0 Hz, 1H, PhCH$_a$***H$_b$***, 4-OBn), 4.59 (d, *J* = 11.7 Hz, 1H, PhCH$_a$***H$_b$***, 2-OBn), 3.93 (td, *J* = 9.2, 3.7 Hz, 1H, ***H-3***), 3.71 (dd, *J* = 3.8, 1.7 Hz, 1H, ***H-2***), 3.69 – 3.61 (m, 2H, ***H-5***, OC***H$_a$***H$_b$CH$_2$), 3.38 – 3.29 (m, 2H, ***H-4***, OCH$_a$***H$_b$***CH$_2$), 3.26 (t, *J* = 6.9 Hz, 2H, C***H$_2$***N$_3$), 2.28 (d, *J* = 9.3 Hz, 1H, 3-O***H***), 1.63 – 1.52 (m, 4H, OCH$_2$C***H$_2$***, C***H$_2$***CH$_2$N$_3$), 1.44 – 1.37 (m, 2H, C***H$_2$***CH$_2$CH$_2$N$_3$), 1.33 (d, *J* = 6.3 Hz, 3H, 6-C***H$_3$***); **^{13}C NMR** (126 MHz, $CDCl_3$): δ 138.52 (Ar***C***CH$_2$, 4-OBn), 137.78 (Ar***C***CH$_2$, 2-OBn), [128.59, 128.44, 128.08, 128.03, 128.00, 127.77 (OBn)], 98.89 (***C-1***), 82.35 (***C-4***), 78.77 (***C-2***), 75.17 (Ph***C***H$_2$, 4-OBn), 73.08 (Ph***C***H$_2$, 2-OBn), 71.72 (***C-3***), 67.19 (O***C***H$_2$CH$_2$), 67.17 (***C-5***), 51.31 (***C***H$_2$N$_3$), 29.00 (OCH$_2$***C***H$_2$), 28.65 (***C***H$_2$CH$_2$N$_3$), 23.45 (***C***H$_2$CH$_2$CH$_2$N$_3$), 18.04 (***C-6***); **HR-ESI-TOF/MS (m/z):** $[M+Na]^+$ calcd. for $C_{25}H_{33}N_3O_5Na$, 478.23179; found, 478.23140.

5-Azidopentyl [2,3,6-tri-*O*-benzyl-4-*O*-(naphthalene-2-ylmethyl)-α-D-gluco-pyranosyl]-(1→3)-2,4-di-*O*-benzyl-α-L-rhamnopyranoside (22)

Donor **20** (1.6 g, 2.3 mmol, 1.4 eq) and anh. DMF (1.9 mL, 25 mmol, 16 eq) were dissolved in anh. DCM (10 mL). The solution was cooled down to 0°C. Molecular sieves (4Å) were added, after which the solution was stirred for 90 min. NIS (0.50 g, 2.2 mmol, 1.4 eq) and TMSOTf (0.40 mL, 2.2 mmol, 1.4 eq) were added, respectively. The solution was stirred at 0°C for 60 min, after which a solution of acceptor **21** (0.71 g, 1.6 mmol, 1.0 eq) in anh. DCM (10 mL) was added *via* a canula. The mixture was stirred at 0°C – R.T. for 20 hrs, after which TLC showed full consumption of the acceptor. The reaction was quenched with TEA (0.50 mL) and stirred for an additional 10 min. The solution was filtered over celite and diluted with DCM. The solution was washed with 10% aq. $Na_2S_2O_3$. The organic layer was dried with $MgSO_4$, filtered and evaporated *in vacuo*. The residue was purified using silica-flash column chromatography (0 – 25% EtOAc in PE$^{100°C-140°C}$), yielding disaccharide **22** as a pale yellow oil (1.4 g, 1.3 mmol, 85%, 6:1 α/β).

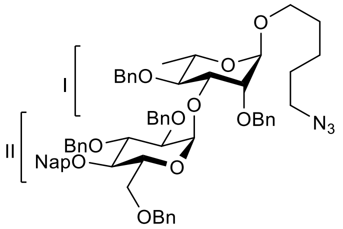

22

TLC: (EtOAc:Tol, 8:92 v/v): R_f = 0.46; **^{1}H NMR** (500 MHz, $CDCl_3$): δ 7.83 – 7.80 (m, 1H, Ar***H***, ONap), 7.74 (d, *J* = 8.4 Hz, 1H, Ar***H***, ONap), 7.70 (dd, 1H, Ar***H***, ONap), 7.51 (s, 1H, Ar***H***, ONap), 7.48 – 7.43 (m, 2H, 2x Ar***H***), 7.37 – 7.33 (m, 2H, 2x Ar***H***, OBn), 7.32 – 7.18 (m, 21H, 21x Ar***H***, OBn), 7.12 – 7.05 (m, 3H, 3x Ar***H***, OBn), 5.20 (d, *J* = 3.5 Hz, 1H, ***H-1^{II}***), 4.99 – 4.94 (m, 2H, PhC***H$_a$***H$_b$, 3^{II}-OBn; PhC***H$_a$***H$_b$, ONap), 4.91 (d, *J* = 10.5 Hz, 1H, PhC***H$_a$***H$_b$, 4^{I}-OBn), 4.88 – 4.82 (m, 2H, PhC***H$_a$***CH$_b$, 2^{I}-OBn; PhCH$_a$***H$_b$***, 3^{II}-OBn), 4.75 (s, 2H, PhC***H$_2$***, 2^{II}-OBn), 4.69 (d, *J* = 2.1 Hz, 1H, ***H-1^{I}***), 4.61 – 4.53 (m, 4H, PhCH$_a$***H$_b$***, 4^{I}-OBn; PhCH$_a$***H$_b$***, ONap; PhC***H$_a$***H$_b$, 6^{II}-OBn), 4.27 (d, *J* = 12.1 Hz, 1H, PhCH$_a$***H$_b$***, 6^{II}-OBn), 4.16 (t, *J* = 9.4 Hz, 1H, ***H-3^{II}***), 4.12 – 4.06 (m, 2H, ***H-3^{I}***, ***H-5^{II}***), 3.86 (t, *J* = 2.5 Hz, 1H, ***H-2^{I}***), 3.80 (t, *J* = 9.5 Hz, 1H, ***H-4^{II}***), 3.70 – 3.63 (m, 3H, ***H-2^{II}***, ***H-4^{I}***, ***H-5^{I}***), 3.62 – 3.56 (m, 2H, ***H-6a^{II}***, OC***H$_a$***H$_b$CH$_2$), 3.45 (dd, *J* = 10.8, 2.1 Hz, 1H, ***H-6b^{II}***), 3.30 (dt, *J* = 9.8, 6.4 Hz, 1H, OCH$_a$***H$_b$***CH$_2$), 3.23 (t, *J* = 6.9 Hz, 2H, C***H$_2$***N$_3$), 1.60 – 1.48 (m, 4H, OCH$_2$C***H$_2$***, C***H$_2$***CH$_2$N$_3$), 1.39 – 1.31 (m, 5H, 6^{I}-C***H$_3$***, C***H$_2$***CH$_2$CH$_2$N$_3$); **^{13}C NMR** (126 MHz, $CDCl_3$): δ 138.75 (Ar***C***CH$_2$, OBn), 138.60 (Ar***C***CH$_2$, OBn), 138.17 (Ar***C***CH$_2$, OBn), 137.98 (Ar***C***CH$_2$, OBn), 137.90 (Ar***C***CH$_2$, OBn), 136.10 (Ar***C***CH$_2$, ONap), 133.24 (Ar***C***C$_2$,ONap), 132.89 (Ar***C***C$_2$,ONap), [128.48, 128.36, 128.34, 128.29, 128.23, 128.03, 127.99, 127.89, 127.87, 127.69, 127.65, 127.64, 127.53, 127.46 (OBn; ONap)], [126.26, 125.97, 125.77 (ONap)], 98.16 (***C-1^{I}***), 95.05 (***C-1^{II}***), 82.28 (***C-3^{II}***), 80.17 (***C-4^{I}***), 79.60 (***C-2^{II}***), 77.86 (***C-4^{II}***), 76.19 (***C-3^{I}***), 75.62 (Ph***C***H$_2$, 4^{I}-OBn), 75.53 (Ph***C***H$_2$, 3^{II}-OBn), 75.49 (***C-2^{I}***), 75.00 (Ph***C***H$_2$, ONap), 73.36 (2C, Ph***C***H$_2$, 2^{II}-OBn; Ph***C***H$_2$, 6^{II}-OBn), 73.29 (Ph***C***H$_2$, 2^{I}-OBn), 70.40 (***C-5^{II}***), 68.28 (***C-***

5^{I}), 68.16 (***C-6^{II}***), 67.18 (O*C*H$_2$CH$_2$), 51.28 (***C***H$_2$N$_3$), 28.97 (OCH$_2$***C***H$_2$), 28.62 (***C***H$_2$CH$_2$N$_3$), 23.38 (***C***H$_2$CH$_2$CH$_2$N$_3$), 18.05 (***C-6^{I}***); **<u>HR-ESI-TOF/MS (m/z):</u>** [M+Na]$^+$ calcd. for $C_{63}H_{69}N_3O_{10}Na$, 1050.48806; found, 1050.48554.

5-Azidopentyl [2,3,6-tri-*O*-benzyl-α-D-glucopyranosyl]-(1→3)-2,4-di-*O*-benzyl-α-L-rhamnopyranoside (23)

Compound **22** (2.51 g, 2.07 mmol, 1.0 eq) was dissolved in DCM:H_2O (50 mL, 9:1 v/v). DDQ (942 mg, 4.15 mmol, 1.9 eq) was added. The mixture was vigourously stirred under exclusion of light for 3 hrs, after which DDQ mixture (5.0 mL) was added. The organic layer was extracted and washed with DDQ mixture (3x 50 mL, aq. $NaHCO_3$ (sat.) (50 mL) and brine (50 mL), respectively. The organic layer was dried with MgSO4, filtered and evaporated *in vacuo*. The residue was purified using silica-flash column chromatography (0-25% EtOAc in PE$^{100-140}$), yielding disaccharide **23** as a colourless oil (1.27 g, 1.43 mmol, 69.1%).

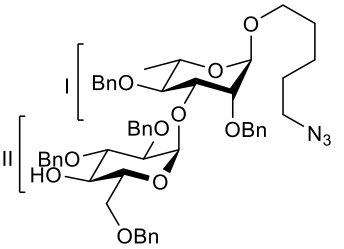

23

<u>TLC:</u> (EtOAc:Tol, 8:92 v/v): R_f = 0.22; **<u>^{1}H NMR</u>** (500 MHz, $CDCl_3$): δ 7.36 – 7.22 (m, 25H, 25x Ar***H***), 5.18 (d, *J* = 3.5 Hz, 1H, ***H-1^{II}***), 4.94 (d, *J* = 11.4 Hz, 1H, PhC***H$_a$***H$_b$, 3^{II}-OBn), 4.88 (d, *J* = 10.9 Hz, 1H, PhC***H$_a$***H$_b$, 4^{I}-OBn), 4.82 (d, *J* = 11.7 Hz, 1H, PhC***H$_a$***CH$_b$, 2^{I}-OBn), 4.75 (d, *J* = 11.6 Hz, 1H, PhCH$_a$***H$_b$***, 3^{II}-OBn), 4.73 – 4.69 (m, 3H, ***H-1^{I}***, PhC***H$_2$***, 2^{II}-OBn), 4.62 – 4.58 (m, 2H, PhCH$_a$C***H$_b$***, 2^{I}-OBn; PhCH$_a$***H$_b$***, 4^{I}-OBn), 4.50 (d, *J* = 12.1 Hz, 1H, PhC***H$_a$***H$_b$, 6^{II}-OBn), 4.39 (d, *J* = 12.1 Hz, 1H, PhCH$_a$***H$_b$***, 6^{II}-OBn), 4.10 (dd, *J* = 8.7, 2.9 Hz, 1H, ***H-3^{I}***), 3.99 (dt, *J* = 10.0, 3.7 Hz, 1H, ***H-5^{II}***), 3.90 (t, *J* = 9.3 Hz, 1H, ***H-3^{II}***), 3.86 (t, *J* = 2.6 Hz, 1H, ***H-2^{I}***), 3.71 – 3.57 (m, 5H, ***H-2^{II}***, ***H-4^{I}***, ***H-4^{II}***, ***H-5^{I}***, OC***H$_a$***H$_b$CH$_2$), 3.53 (qd, *J* = 10.7, 3.9 Hz, 2H, 2x ***H6^{II}***), 3.30 (dt, *J* = 10.0, 6.4 Hz, 1H, OCH$_a$***H$_b$***CH$_2$), 3.21 (t, *J* = 6.9 Hz, 2H, C***H$_2$***N$_3$), 2.11 (d, *J* = 3.0 Hz, 1H, 4^{II}-O***H***), 1.59 – 1.47 (m, 4H, OCH$_2$C***H$_2$***, C***H$_2$***CH$_2$N$_3$), 1.39 – 1.29 (m, 5H, 6^{I}-C***H$_3$***, C***H$_2$***CH$_2$CH$_2$N$_3$); **<u>^{13}C NMR</u>** (126 MHz, $CDCl_3$): δ 138.73 (Ar***C***CH$_2$, OBn), 138.55 (Ar***C***CH$_2$, OBn), 138.21 (Ar***C***CH$_2$, OBn), 138.07 (Ar***C***CH$_2$-, OBn), [128.53, 128.38, 128.35, 128.28, 128.26, 128.25, 128.04, 127.94, 127.79, 127.73, 127.70, 127.68, 127.61, 127.54, 127.49 (OBn)], 98.12 (***C-1^{I}***), 95.05 (***C-1^{II}***), 81.33 (***C-3^{II}***), 80.13 (***H-4^{I}***), 79.34 (***C-2^{II}***), 76.18 (***C-3^{I}***), 75.57 (***C-2^{I}***), 75.25 (Ph***C***H$_2$, 4^{I}-OBn), 75.15 (Ph***C***H$_2$, 3^{II}-OBn), 73.39 (Ph***C***H$_2$, 6^{II}-OBn), 73.25 (Ph***C***H$_2$, 2^{I}-OBn), 73.08 (Ph***C***H$_2$, 2^{II}-OBn), 71.08 (***C-4^{II}***), 70.17 (***C-5^{II}***), 69.33 (***C-6^{II}***), 68.27 (C-5^{I}), 67.20 (O***C***H$_2$CH$_2$), 51.26 (***C***H$_2$N$_3$), 28.95 (OCH$_2$***C***H$_2$), 28.60 (***C***H$_2$CH$_2$N$_3$), 23.37 (***C***H$_2$CH$_2$CH$_2$N$_3$), 18.05 (***C-6^{I}***); **<u>HR-ESI-TOF/MS (m/z):</u>** [M+Na]$^+$ calcd. for $C_{52}H_{61}N_3O_{10}Na$, 910.42546; found, 910.42731.

5-Azidopentyl [2,4-di-*O*-benzyl-α-L-rhamnopyranosyl]-(1→4)-[2,3,6-tri-*O*-benzyl-α-D-glucopyranosyl]-(1→3)-2,4-di-*O*-benzyl-α-L-rhamnopyranoside (24)

Donor **1** (1.04 g, 1.82 mmol, 1.4 eq) and acceptor **23** (1.13 g, 1.28 mmol, 1.0 eq) were dissolved in anh. DCM (30 mL). The solution was cooled down to 0°C. Molecular sieves (4Å) were added, after which the solution was stirred for 90 min. The solution was cooled down to -78°C. NIS (402 mg, 1.79 mmol, 1.4 eq) and TfOH (7 µL, 79 µmol, 0.062 eq) were added, respectively. The solution was stirred at -78°C to 0°C for 60 min, turning bright red over time. The solution was quenched with TEA (0.50 mL) and stirred for an additional 10 min. The mixture was filtered over celite, after which the filtrate was washed with 10% aq. $Na_2S_2O_3$. The organic layer was dried with $MgSO_4$, filtered and evaporated *in vacuo*. The residue was dissolved in MeOH:THF (50 mL, 3:2 v/v). 5.4 molar NaOMe in MeOH (0.50 mL, 2.7 mmol, 2.1 eq) was added. The solution was stirred for 64 hrs. The solution was heated to 40°C, after which it was stirred for an additional 20 hrs. DOWEX 50W X8(H$^+$) was added, after which the mixture was stirred for an additional 15 min. The solution was filtered and evaporated *in vacuo*. The residue was purified using silica-flash column chromatography (0 – 8% EtOAc in Tol), yielding trisaccharide **24** as a colourless oil (1.36 g, 1.12 mmol, 87.5%).

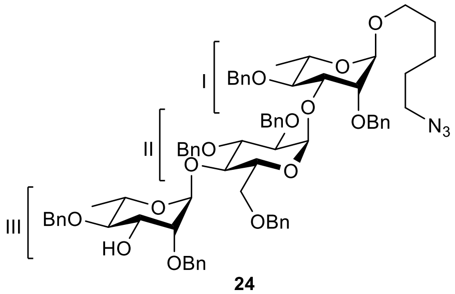

24

<u>TLC:</u> (EtOAc:Tol, 8:92 v/v): R_f = 0.24; **<u>^{1}H NMR</u>** (500 MHz, $CDCl_3$): δ 7.38 – 7.17 (m, 35H, 35x Ar***H***, OBn), 5.15 (d, *J* = 3.5 Hz, 1H, ***H-1^{II}***), 5.04 (d, *J* = 1.7 Hz, 1H, ***H-1^{III}***), 5.00 (d, *J* = 10.6 Hz, 1H, PhC***H$_a$***H$_b$, 3^{II}-OBn), 4.90 (d, *J* = 11.0 Hz, 1H, PhC***H$_a$***H$_b$, 4^{I}-OBn), 4.85 – 4.80 (m, 2H, PhC***H$_a$***H$_b$, 3^{III}-OBn; PhC***H$_a$***H$_b$, 2^{I}-OBn), 4.77 (d, *J* = 10.6 Hz, 1H, PhCH$_a$***H$_b$***, 3^{II}-OBn), 4.72 – 4.68 (m, 3H, ***H-1^{I}***, PhC***H$_2$***, 2^{II}-OBn), 4.63 (d, *J* = 10.9 Hz, 1H, PhCH$_a$***H$_b$***, 4^{I}-OBn), 4.60 – 4.54 (m, 2H, PhCH$_a$***H$_b$***, 2^{I}-OBn; PhCH$_a$***H$_b$***, 4^{III}-OBn), 4.50 – 4.45 (m, 2H, PhC***H$_a$***H$_b$, 2^{III}-OBn; PhC***H$_a$***H$_b$, 6^{II}-OBn), 4.33 (d, *J* = 12.0 Hz, 1H, PhCH$_a$***H$_b$***, 6^{II}-OBn), 4.25 (d, *J* = 11.8 Hz, 1H, PhCH$_a$***H$_b$***, 2^{III}-OBn), 4.08 (dd, *J* = 8.9, 2.9 Hz, 1H, ***H-3^{I}***), 3.98 – 3.93 (m, 2H, ***H-3^{II}***, ***H-5^{II}***), 3.92 – 3.82 (m, 4H, ***H-2^{I}***, ***H-4^{II}***, ***H-3^{III}***, ***H-5^{III}***), 3.72 – 3.62 (m, 3H, ***H-2^{II}***, ***H-4^{I}***, ***H-5^{I}***), 3.62 – 3.57 (m, 1H, OC***H$_a$***H$_b$CH$_2$), 3.49 (dd, *J* = 3.7, 1.6 Hz, 1H, ***H-2^{III}***), 3.45 (dd, *J* = 11.4, 1.9 Hz, 1H, ***H-6$_a$^{II}***), 3.36 – 3.28 (m, 2H, ***H-6$_b$^{II}***, OCH$_a$***H$_b$***CH$_2$), 3.27 – 3.21 (m, 3H, ***H-4^{III}***, C***H$_2$***N$_3$), 2.23 (d, *J* = 8.9 Hz, 1H, 3^{III}-O***H***), 1.60 – 1.49 (m, 4H, C***H$_2$***CH$_2$N$_3$, OCH$_2$C***H$_2$***), 1.41 – 1.33 (m, 5H, 6^{I}-

C***H₃***, C***H₂***CH$_2$CH$_2$N$_3$), 0.98 (d, *J* = 6.2 Hz, 3H, 6^{III}-C***H₃***); **<u>^{13}C NMR</u>** (126 MHz, CDCl$_3$): δ 138.84 (Ar***C***CH$_2$, OBn), 138.53 (Ar***C***CH$_2$, OBn), 138.46 (Ar***C***CH$_2$, OBn), 138.21 (Ar***C***CH$_2$, OBn), 137.89 (Ar***C***CH$_2$, OBn), 137.83 (Ar***C***CH$_2$, OBn), [128.50, 128.48, 128.36, 128.31, 128.29, 128.22, 128.16, 128.04, 128.00, 127.87, 127.81, 127.77, 127.73, 127.71, 127.69, 127.60, 127.56, 127.52, 127.49, 127.48, 127.32 (OBn)], 98.02 (***C-1^{I}***), 97.46 (***C-1^{III}***), 94.45 (***C-1^{II}***), 82.36 (***C-4^{III}***), 80.28 (***C-3^{II}***), 80.08 (***C-2^{II}***), 79.95 (***C-4^{I}***), 78.43 (***C-2^{III}***), 76.06 (***C-3^{I}***), 75.54 (***C-2^{I}***), 75.38 (Ph***C***H$_2$, 4^{II}-OBn), 75.33 (Ph***C***H$_2$, 3^{II}-OBn), 75.10 (***C-4^{II}***), 74.85 (Ph***C***H$_2$, 4^{III}-OBn), 73.43 (Ph***C***H$_2$, 6^{II}-OBn), 73.36 (Ph***C***H$_2$, 2^{II}-OBn), 73.30 (Ph***C***H$_2$, 2^{I}-OBn), 72.51 (Ph***C***H$_2$, 2^{III}-OBn), 71.44 (***C-3^{III}***), 70.38 (***C-5^{II}***), 68.84 (***C-6^{II}***), 68.35 (***C-5^{I}***), 67.94 (***C-5^{III}***), 67.21 (O***C***H$_2$CH$_2$), 51.28 (***C***H$_2$N$_3$), 28.96 (OCH$_2$***C***H$_2$), 28.62 (***C***H$_2$CH$_2$N$_3$), 23.38 (***C***H$_2$CH$_2$CH$_2$N$_3$), 18.00 (***C-6^{I}***), 17.87 (***C-6^{III}***); **<u>HR-ESI-TOF/MS (m/z):</u>** [M+Na]$^+$ calcd. for $C_{72}H_{83}N_3O_{14}Na$, 1236.57727; found, 1236.57710.

5-Azidopentyl [2,3,6-tri-*O*-benzyl-4-*O*-(naphthalene-2-ylmethyl)-α-D-glucopyranosyl]-(1→3)-[2,4-di-*O*-benzyl-α-L-rhamnopyranosyl]-(1→4)-[2,3,6-tri-*O*-benzyl-α-D-glucopyranosyl]-(1→3)-2,4-di-*O*-benzyl-α-L-rhamnopyranoside (25)

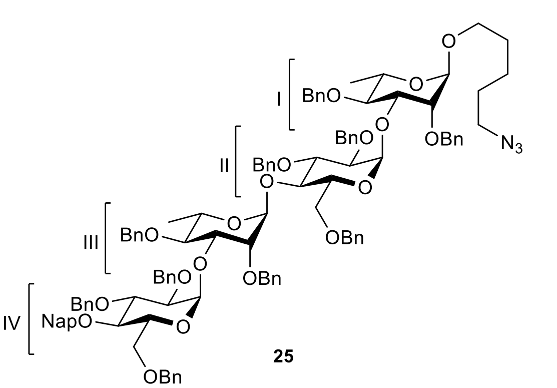

25

Donor **20** (0.64 g, 0.92 mmol, 1.3 eq) and anh. DMF (0.80 mL, 10 mmol, 15 eq) were dissolved in anh. DCM (8.0 mL). The solution was cooled down to 0°C. Molecular sieves (4Å) were added, after which the solution was stirred for 90 min. NIS (0.21 g, 0.94 mmol, 1.4 eq) and TMSOTf (0.17 mL, 0.94 mmol, 1.4 eq) were added, respectively. The solution was stirred at 0°C for 60 min, after which a solution of acceptor **24** (0.84 g, 0.69 mmol, 1.0 eq) in anh. DCM (4.0 mL) was added via a canula. The mixture was stirred at 0°C – R.T. for 20 hrs, after which TLC showed full consumption of the acceptor. The reaction was quenched with TEA (0.30 mL) and stirred for an additional 10 min. The solution was filtered over celite and diluted with DCM. The solution was washed with 10% aq. Na$_2$S$_2$O$_3$. The organic layer was dried with MgSO$_4$, filtered and evaporated *in vacuo*. The residue was purified using size-exclusion chromatography (Biorad S-X1 support) and silica-flash column chromatography (0 – 20% EtOAc in PE$^{100°C-140°C}$), yielding tetrasaccharide **25** as a colourless oil (1.1 g, 0.60 mmol, 88%, 10:1 α/β). **<u>TLC</u>:** (EtOAc:Tol, 10:90 v/v): R$_f$ = 0.46; **<u>^{1}H NMR</u>** (500 MHz, CDCl$_3$): δ 7.83 (dd, *J* = 6.1, 3.4 Hz, 1H, Ar***H***, ONap), 7.74 (d, *J* = 8.4 Hz, 1H, Ar***H***, ONap), 7.71 (dd, *J* = 6.1, 3.3 Hz, Ar***H***, ONap), 7.51 – 7.43 (m, 3H, 3x Ar***H***, ONap), 7.39 – 7.03 (m, 51H, Ar***H***, ONap; 50x Ar***H***, OBn), 5.16 (d, *J* = 3.6 Hz, 1H, ***H-1^{II}***), 5.10 (d, *J* = 3.7 Hz, 1H, ***H-4^{IV}***), 5.09 (d, *J* = 2.2 Hz, 1H, ***H-1^{III}***), 4.96 – 4.90 (m, 3H, PhCH$_a$***H$_b$***, 3^{IV}-OBn; PhC***H$_a$***H$_b$, 4^{I}-OBn; PhC***H$_a$***H$_b$, ONap), 4.90 – 4.78 (m, 5H, PhC***H$_a$***H$_b$, 2^{I}-OBn; PhC***H₂***, 3^{II}-OBn; PhCH$_a$***H$_b$***, 3^{IV}-OBn; PhC***H$_a$***H$_b$, 4^{III}-OBn), 4.72 – 4.66 (m, 3H, ***H-1^{I}***, PhC***H₂***, 2^{II}-OBn), 4.66 – 4.51 (m, 7H, PhCH$_a$***H$_b$***, 2^{I}-OBn; PhC***H$_a$***H$_b$, 2^{III}-OBn; PhC***H₂***, 2^{IV}-OBn; PhCH$_a$***H$_b$***, 4^{I}-OBn; PhCH$_a$***H$_b$***, 4^{III}-OBn; PhCH$_a$***H$_b$***, ONap), 4.44 – 4.37 (m, 3H, PhCH$_a$***H$_b$***, 2^{III}-OBn; PhC***H$_a$***H$_b$, 6^{II}-OBn; PhC***H$_a$***H$_b$, 6^{IV}-OBn), 4.32 (d, *J* = 11.9 Hz, 1H, PhCH$_a$***H$_b$***, 6^{II}-OBn), 4.19 – 4.10 (m, 3H, ***H-3^{III}***, ***H-3^{IV}***, PhCH$_a$***H$_b$***, 6^{IV}-OBn), 4.09 – 4.03 (m, 2H, ***H-3^{I}***, ***H-5^{IV}***), 4.02 – 3.90 (m, 4H, ***H-3^{II}***, ***H-4^{II}***, ***H-5^{II}***, ***H-5^{III}***), 3.88 (t, *J* = 2.6 Hz, 1H, ***H-2^{I}***), 3.83 – 3.74 (m, 2H, ***H-2^{III}***, ***H-4^{IV}***), 3.71 – 3.56 (m, 6H, ***H-2^{II}***, ***H-2^{IV}***, ***H-4^{I}***, ***H-4^{III}***, ***H-5^{I}***, OC***H$_a$***H$_b$CH$_2$), 3.46 – 3.35 (m, 3H, ***H-6$_a$^{I}***, ***H-6$_b$^{II}***, ***H-6$_a$^{IV}***), 3.29 (dt, *J* = 10.0, 6.4 Hz, 1H, OCH$_a$***H$_b$***CH$_2$), 3.26 – 3.18 (m, 3H, ***H-6$_b$^{IV}***, C***H₂***N$_3$), 1.58 – 1.47 (m, 4H, C***H₂***CH$_2$N$_3$, OCH$_2$C***H₂***), 1.39 – 1.28 (m, 5H, 6^{I}-C***H₃***, C***H₂***CH$_2$CH$_2$N$_3$), 1.14 (d, *J* = 6.1 Hz, 3H, 6^{III}-C***H₃***); **<u>^{13}C NMR</u>** (126 MHz, CDCl$_3$): δ 138.70 (Ar***C***CH$_2$, OBn), 138.51 (Ar***C***CH$_2$, OBn), 138.47 (Ar***C***CH$_2$, OBn), 138.34 (Ar***C***CH$_2$, OBn), 138.23 (Ar***C***CH$_2$, OBn), 138.22 (Ar***C***CH$_2$, OBn), 138.13 (Ar***C***CH$_2$, OBn), 138.00 (Ar***C***CH$_2$, OBn), 137.77 (Ar***C***CH$_2$, OBn), 136.14 (Ar***C***CH$_2$, ONap), 133.18 (Ar***C***C$_2$, ONap), 132.83 (Ar***C***C$_2$, ONap), 128.47 (OBn), [128.32, 128.31, 128.28, 128.24, 128.21, 128.19, 128.12, 128.09, 128.06, 127.95, 127.87, 127.81, 127.75, 127.64, 127.53, 127.49, 127.39, 127.27, 127.20 (OBn; ONap)], [126.22 126.02, 125.94, 125.73 (ONap)], 98.53 (***C-1^{III}***), 98.07 (***C-1^{I}***), 95.51 (***C-1^{II}***), 94.07 (***C-1^{IV}***), 82.23 (***C-3^{IV}***), 80.76 (***C-3^{II}***), 80.19 (***C-4^{I}***), 79.99 (***C-2^{II}***), 79.76 (***C-4^{III}***), 79.55 (***C-2^{IV}***), 77.74 (***C-4^{IV}***), 76.93 (***C-3^{I}***), 75.83 (***C-2^{I}***), 75.58 (PhCH$_2$-, 3^{II}-OBn), 75.53 (Ph***C***H$_2$, 4^{III}-OBn), 75.49 (Ph***C***H$_2$, 3^{IV}-OBn), 75.35 (***C-3^{III}***), 75.24 (Ph***C***H$_2$, 4^{I}-OBn), 75.05 (***C-2^{III}***), 74.91 (Ph***C***H$_2$, ONap), 74.60 (***C-4^{II}***), 73.24 (2C, Ph***C***H$_2$, 2^{II}-OBn; Ph***C***H$_2$, 6^{IV}-OBn), 73.18 (Ph***C***H$_2$, 2^{I}-OBn), 73.15 (Ph***C***H$_2$, 6^{II}-OBn), 73.07 (Ph***C***H$_2$, 2^{IV}-OBn), 72.84 (Ph***C***H$_2$, 2^{III}-OBn), 70.68 (***C-5^{II}***), 70.14 (***C-5^{IV}***), 69.05 (***C-5^{III}***), 68.82 (***C-6^{II}***), 68.20 (***C-5^{I}***), 67.94 (***C-6^{IV}***), 67.19 (O***C***H$_2$CH$_2$), 51.21 (***C***H$_2$N$_3$), 28.95 (OCH$_2$***C***H$_2$), 28.58 (***C***H$_2$CH$_2$N$_3$), 23.32 (***C***H$_2$CH$_2$CH$_2$N$_3$), 18.05 (***C-6^{I}***), 18.02 (***C-6^{III}***); **<u>HR-ESI-TOF/MS (m/z):</u>** [M+Na]$^+$ calcd. for $C_{110}H_{119}N_3O_{19}Na$, 1808.83354; found, 1808.83038.

5-Azidopentyl [2,3,6-tri-*O*-benzyl-α-D-glucopyranosyl]-(1→3)-[2,4-di-*O*-benzyl-α-L-rhamnopyranosyl]-(1→4)-[2,3,6-tri-*O*-benzyl-α-D-glucopyranosyl]-(1→3)-2,4-di-*O*-benzyl-α-L-rhamnopyranoside (26)

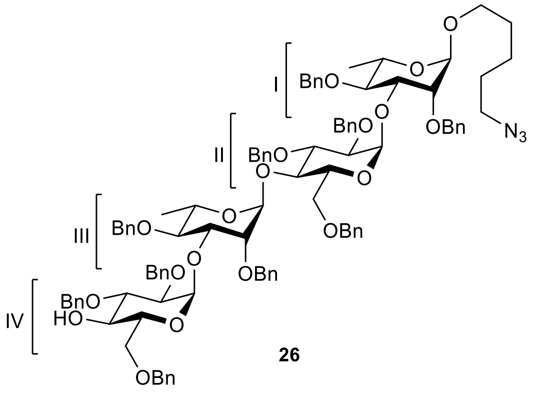

26

Compound **25** (0.24 g, 0.14 mmol, 1.0 eq) was dissolved in DCM:H_2O (5.0 mL, 9:1 v/v). The solution was cooled down to 12°C. DDQ (38 mg, 0.17 mmol, 1.2 eq) was added. The mixture was vigourously stirred under exclusion of light for 2 hrs, after which DCM (5.0 mL) and DDQ mixture (10 mL) were added. The organic layer was extracted and washed with H_2O (10 mL) and aq. $NaHCO_3$ (sat.) (10 mL), respectively. The organic layer was dried with MgSO4, filtered and evaporated *in vacuo*. The residue was purified using silica-flash column chromatography (0-10% EtOAc in Tol), yielding tetrasaccharide **26** as a colourless oil (0.15 g, 91 µmol, 67%).

<u>TLC:</u> (EtOAc:Tol, 10:90 v/v): R_f = 0.36; **<u>^{1}H NMR</u>** (500 MHz, $CDCl_3$): δ 7.41 – 7.10 (m, 50H, 50x Ar*H*, OBn), 5.16 (d, *J* = 3.5 Hz, 1H, ***H-1***^{II}), 5.10 (d, *J* = 2.1 Hz, 1H, ***H-1***^{III}), 5.08 (d, *J* = 3.5 Hz, 1H, ***H-1***^{IV}), 4.93 – 4.83 (m, 4H, PhC***Ha***Hb, 3^{II}-OBn; PhC***Ha***Hb, 3^{IV}-OBn; PhC***Ha***Hb, 4^{I}-OBn; PhC***Ha***Hb, 4^{III}-OBn), 4.83 – 4.78 (m, 2H, PhC***Ha***Hb, 2^{I}-OBn; PhCHa***Hb***, 3^{II}-OBn), 4.74 – 4.67 (m, 4H, ***H-1***^{I}, PhC***H₂***, 2^{II}-OBn; PhCHa***Hb***, 3^{IV}-OBn), 4.63 – 4.53 (m, 6H, PhCHa***Hb***, 2^{I}-OBn; PhC***Ha***Hb, 2^{III}-OBn; PhC***H₂***, 2^{IV}-OBn; PhCHa***Hb***, 4^{I}-OBn; PhCHa***Hb***, 4^{III}-OBn), 4.41 – 4.34 (m, 3H, PhCHa***Hb***, 2^{III}-OBn; PhC***Ha***Hb, 6^{II}-OBn; PhC***Ha***Hb, 6^{IV}-OBn), 4.32 (d, *J* = 12.1 Hz, 1H, PhCHa***Hb***, 6^{II}-OBn), 4.29 (d, *J* = 12.2 Hz, 1H, PhCHa***Hb***, 6^{IV}-OBn), 4.11 (dd, *J* = 9.2, 2.8 Hz, 1H, ***H-3***^{III}), 4.07 (dd, *J* = 8.6, 3.0 Hz, 1H, ***H-3***^{I}), 4.02 – 3.93 (m, 4H, ***H-3***^{II}, ***H-5***^{II}, ***H-5***^{III}, ***H-5***^{IV}), 3.93 – 3.83 (m, 3H, ***H-2***^{I}, ***H-3***^{IV}, ***H-4***^{II}), 3.77 (t, *J* = 2.5 Hz, 1H, ***H-2***^{III}), 3.71 – 3.57 (m, 6H, ***H-2***^{II}, ***H-4***^{I}, ***H-4***^{III} ***H-4***^{IV}, ***H-5***^{I}, OC***Ha***HbCH₂), 3.55 (dd, *J* = 9.6, 3.4 Hz, 1H, ***H-2***^{IV}), 3.45 – 3.33 (m, 4H, ***H-6a***^{II}, ***H-6b***^{II}, ***H-6a***^{IV}, ***H-6b***^{IV}), 3.30 (dt, *J* = 10.0, 6.5 Hz, 1H, OCHa***Hb***CH₂), 3.21 (t, *J* = 6.9 Hz, 2H, C***H₂***N₃), 2.04 (d, *J* = 3.2 Hz, 1H, 4^{IV}-O***H***), 1.59 – 1.47 (m, 4H, C***H₂***CH₂N₃, OCH₂C***H₂***), 1.39 – 1.30 (m, 5H, 6^{I}-C***H₃***, C***H₂***CH₂CH₂N₃), 1.12 (d, *J* = 6.1 Hz, 3H, 6^{III}-C***H₃***); **<u>^{13}C NMR</u>** (126 MHz, $CDCl_3$): δ 138.70 (Ar***C***CH₂, OBn), 138.48 (Ar***C***CH₂, OBn), 138.44 (Ar***C***CH₂, OBn), 138.34 (Ar***C***CH₂, OBn), 138.24 (Ar***C***CH₂, OBn), 138.17 (Ar***C***CH₂, OBn), 138.05 (Ar***C***CH₂, OBn), 137.99 (Ar***C***CH₂, OBn), 137.88 (Ar***C***CH₂, OBn), [128.47, 128.45, 128.31, 128.30, 128.27, 128.24, 128.21, 128.19, 128.14, 128.06, 128.02, 127.95, 127.93, 127.74, 127.71, 127.67, 127.64, 127.63, 127.59, 127.56, 127.49, 127.42, 127.38, 127.31, 127.16 (OBn)], 98.43 (***C-1***^{III}), 98.06 (***C-1***^{I}), 95.50 (***C-1***^{II}), 93.76 (***C-1***^{IV}), 81.28 (***C-3***^{IV}), 80.80 (***C-3***^{II}), 80.17 (***C-4***^{I}), 79.97 (***C-2***^{II}), 79.70 (***C-4***^{III}), 79.19 (***C-2***^{IV}), 76.91 (***C-3***^{I}), 75.82 (***C-2***^{I}), 75.55 (Ph***C***H₂, 3^{II}-OBn), 75.22 (Ph***C***H₂, 4^{I}-OBn), 75.11 (Ph***C***H₂, 3^{IV}-OBn; Ph***C***H₂, 4^{III}-OBn), 75.07 (***C-3***^{III}), 74.81 (***C-2***^{III}), 74.48 (***C-4***^{II}), 73.35 (Ph***C***H₂, 6^{IV}-OBn), 73.28 (Ph***C***H₂, 2^{II}-OBn), 73.18 (Ph***C***H₂, 2^{I}-OBn), 73.11 (Ph***C***H₂, 6^{II}-OBn), 72.82 (Ph***C***H₂, 2^{IV}-OBn), 72.77 (Ph***C***H₂, 2^{III}-OBn), 71.30 (***C-4***^{IV}), 70.65 (***C-5***^{II}), 69.72 (***C-5***^{IV}), 69.35 (***C-6***^{IV}), 69.00 (***C-5***^{III}), 68.81 (***C-6***^{II}), 68.19 (***C-5***^{I}), 67.19 (O***C***H₂CH₂), 51.21 (***C***H₂N₃), 28.95 (OCH₂***C***H₂), 28.58 (***C***H₂CH₂N₃), 23.33 (***C***H₂CH₂CH₂N₃), 18.03 (***C-6***^{I}), 18.01 (***C-6***^{III}); **<u>HR-ESI-TOF/MS (m/z):</u>** $[M+Na]^+$ calcd. for $^{12}C_{98}{}^{13}C_1H_{111}N_3O_{19}Na$, 1669.77430; found, 1669.77732.

5-Azidopentyl [2,4-di-*O*-benzyl-α-L-rhamnopyranosyl]-(1→4)-[2,3,6-tri-*O*-benzyl-α-D-glucopyranosyl]-(1→3)-[2,4-di-*O*-benzyl-α-L-rhamnopyranosyl]-(1→4)-[2,3,6-tri-*O*-benzyl-α-D-glucopyranosyl]-(1→3)-2,4-di-*O*-benzyl-α-L-rhamnopyranoside (27)

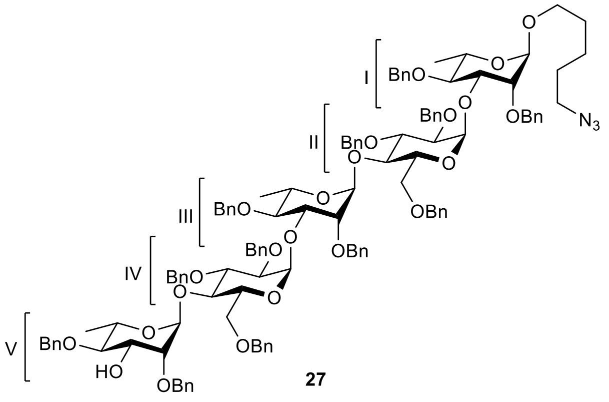

27

Donor **1** (62 mg, 0.11 mmol, 2.1 eq) and acceptor **26** (85 mg, 52 µmol, 1.0 eq) were dissolved in anh. DCM (3.0 mL). The solution was cooled down to 0°C. Molecular sieves (4Å) were added, after which the solution was stirred for 90 min. The solution was cooled down to -78°C. NIS (25 mg, 0.11 mmol, 2.2 eq) and TfOH (1.0 µL, 11 µmol, 0.22 eq) were added, respectively. The solution was stirred at -78°C for 15 min, after which it was stirred at -78°C to 0°C for 30 min, turning bright red over time. The solution was quenched with TEA (0.20 mL) and stirred for an additional 10 min. The mixture was filtered over celite, after which the filtrate was washed with 10% aq. $Na_2S_2O_3$ (10 mL). The organic layer was dried with $MgSO_4$, filtered and evaporated *in vacuo*. The residue was dissolved in MeOH:THF (5.0 mL, 3:2 v/v). 5.4 molar NaOMe in MeOH (0.10 mL, 0.54 mmol, 10 eq) was added. The solution was heated to 45°C and stirred for 20 hrs. DOWEX 50W X8(H^+) was added, after which the mixture was stirred for an additional 15 min. The solution was filtered and evaporated *in vacuo*. The residue was purified using size-exclusion chromatography (Biorad S-X1 support), yielding pentasaccharide **27** as a colourless oil (74 mg, 37 µmol, 73%).

TLC: (EtOAc:Tol, 10:90 v/v): R_f = 0.35; **^{1}H NMR** (500 MHz, $CDCl_3$): δ 7.44 – 7.07 (m, 60H, 60x Ar***H***, OBn), 5.17 (d, *J* = 3.6 Hz, 1H), 5.11 (s, 1H, ***H-1***^{III}), 5.05 (d, *J* = 3.6 Hz, 1H, ***H-1***^{IV}), 5.00 (s, 1H, ***H-1***^{V}), 4.97 (d, *J* = 10.7 Hz, 1H, PhC***H**a*H$_b$, 3^{IV}-OBn), 4.94 (d, *J* = 10.8 Hz, 1H, PhC***H**a*H$_b$, 4^{I}-OBn), 4.89 – 4.78 (m, 5H, PhC***H**a*H$_b$, 2^{I}-OBn; PhC***H₂***, 3^{II}-OBn; PhC***H**a*H$_b$, 4^{III}-OBn; PhC***H**a*H$_b$, 4^{V}-OBn), 4.75 – 4.65 (m, 4H, ***H-1***^{I}, PhC***H***$_2$, 2^{II}-OBn; PhCH$_a$***H**b*, 3^{IV}-OBn), 4.63 – 4.53 (m, 7H, PhCH$_a$***H**b*, 2^{I}-OBn; PhC***H**a*H$_b$, 2^{III}-OBn; PhC***H***$_2$, 2^{IV}-OBn; PhCH$_a$***H**b*, 4^{I}-OBn; PhCH$_a$***H**b*, 4^{III}-OBn; PhCH$_a$***H**b*, 4^{V}-OBn), 4.46 (d, *J* = 11.8 Hz, 1H, PhC***H**a*H$_b$, 2^{V}-OBn), 4.41 (d, *J* = 12.0 Hz, 1H, PhC***H**a*H$_b$, 6^{II}-OBn), 4.37 – 4.31 (m, 3H, PhCH$_a$***H**b*, 2^{III}-OBn; PhCH$_a$***H**b*, 6^{II}-OBn; PhC***H**a*H$_b$, 6^{IV}-OBn), 4.29 – 4.20 (m, 2H, PhCH$_a$***H**b*, 2^{V}-OBn; PhCH$_a$***H**b*, 6^{IV}-OBn), 4.13 – 4.06 (m, 2H, ***H-3***^{I}, ***H-3***^{III}), 4.02 (dd, *J* = 9.4, 6.0 Hz, 1H, ***H-5***^{III}), 4.00 – 3.94 (m, 2H, ***H-3***^{II}, ***H-5***^{II}), 3.93 – 3.78 (m, 7H, ***H-2***^{I}, ***H-3***^{IV}, ***H-3***^{V}, ***H-4***^{II}, ***H-4***^{IV}, ***H-5***^{IV}, ***H-5***^{V}), 3.75 (d, *J* = 2.7 Hz, 1H, ***H-2***^{III}), 3.72 – 3.55 (m, 6H, ***H-2***^{II}, ***H-2***^{IV}, ***H-4***^{I}, ***H-4***^{III}, ***H-5***^{I}, OC***H**a*H$_b$CH$_2$), 3.49 – 3.35 (m, 3H, ***H-2***^{V}, ***H-6a***^{II}, ***H-6b***^{II}), 3.34 – 3.26 (m, 2H, ***H-6a***^{IV}, OCH$_a$***H**b*CH$_2$), 3.26 – 3.18 (m, 3H, ***H-4***^{V}, C***H***$_2$N$_3$), 3.15 (dd, *J* = 11.1, 2.9 Hz, 1H, ***H-6b***^{IV}), 2.22 (d, *J* = 8.9 Hz, 1H, 3^{V}-O***H***), 1.61 – 1.47 (m, 4H, C***H***$_2$CH$_2$N$_3$, OCH$_2$C***H***$_2$), 1.35 (m, 5H, 6^{I}-C***H***$_3$, C***H***$_2$CH$_2$CH$_2$N$_3$), 1.17 (d, *J* = 6.0 Hz, 3H, 6^{III}-C***H***$_3$), 0.96 (d, *J* = 6.1 Hz, 3H, 6^{V}-C***H***$_3$); **^{13}C NMR** (126 MHz, $CDCl_3$): δ 138.88 (Ar***C***CH$_2$, OBn), 138.52 (Ar***C***CH$_2$, OBn), 138.45 (Ar***C***CH$_2$, OBn), 138.40 (Ar***C***CH$_2$, OBn), 138.34 (Ar***C***CH$_2$, OBn), 138.17 (Ar***C***CH$_2$, OBn), 138.03 (Ar***C***CH$_2$, OBn), 137.89 (Ar***C***CH$_2$, OBn), 137.87 (Ar***C***CH$_2$, OBn), 137.80 (Ar***C***CH$_2$, OBn), [128.47, 128.34, 128.29, 128.27, 128.22, 128.16, 128.12, 128.09, 128.02, 127.96, 127.85, 127.76, 127.69, 127.63, 127.61, 127.58, 127.55, 127.51, 127.44, 127.36, 127.31, 127.28, 127.17 (OBn)], 98.32 (***C-1***^{III}), 98.08 (***C-1***^{I}), 97.37 (***C-1***^{V}), 95.37 (***C-1***^{II}), 93.29(***C-1***^{IV}), 82.37 (***C-4***^{V}), 80.90 (***C-3***^{II}), 80.30 (***C-4***^{I}), 80.17 (***C-2***^{IV}, ***C-3***^{IV}), 80.02 (***C-2***^{II}), 79.56 (***C-4***^{III}), 78.39 (***C-2***^{V}), 76.83 (***C-3***^{I}), 75.85 (***C-2***^{I}), 75.52 (Ph***C***H$_2$, 3^{II}-OBn), 75.35 – 75.10 (***C-3***^{III}, Ph***C***H$_2$, 3^{IV}-OBn; Ph***C***H$_2$, 4^{I}-OBn; Ph***C***H$_2$, 4^{III}-OBn), 74.98 (***C-4***^{IV}), 74.91 (***C-2***^{III}), 74.86 (Ph***C***H$_2$, 2^{III}-OBn), 74.44 (***C-4***^{II}), 73.42 (Ph***C***H$_2$, 6^{IV}-OBn), 73.28 (Ph***C***H$_2$, 2^{II}-OBn), 73.22 (Ph***C***H$_2$, 6^{II}-OBn), 73.18 (Ph***C***H$_2$, 2^{I}-OBn), 73.05 (Ph***C***H$_2$, 2^{IV}-OBn), 72.86 (Ph***C***H$_2$, 2^{III}-OBn), 72.43 (Ph***C***H$_2$, 2^{V}-OBn), 71.40 (***C-3***^{V}), 70.70 (***C-5***^{II}), 70.23 (***C-5***^{IV}), 69.14 (***C-5***^{III}), 68.93 (***C-6***^{II}), 68.67 (***C-6***^{IV}), 68.25 (***C-5***^{I}), 67.86 (***C-5***^{V}), 67.24 (-O***C***H$_2$CH$_2$-), 51.25 (-***C***H$_2$N$_3$), 28.98 (-OCH$_2$***C***H$_2$-), 28.60 (-***C***H$_2$CH$_2$N$_3$), 23.36 (-***C***H$_2$CH$_2$CH$_2$N$_3$), 18.08 (***C-6***^{I}), 17.99(***C-6***^{III}), 17.85 (***C-6***^{V}); **HR-ESI-TOF/MS (m/z):** $[M+Na]^+$ calcd. for $C_{119}H_{133}N_3O_{23}Na$, 1994.92275; found, 1994.92630.

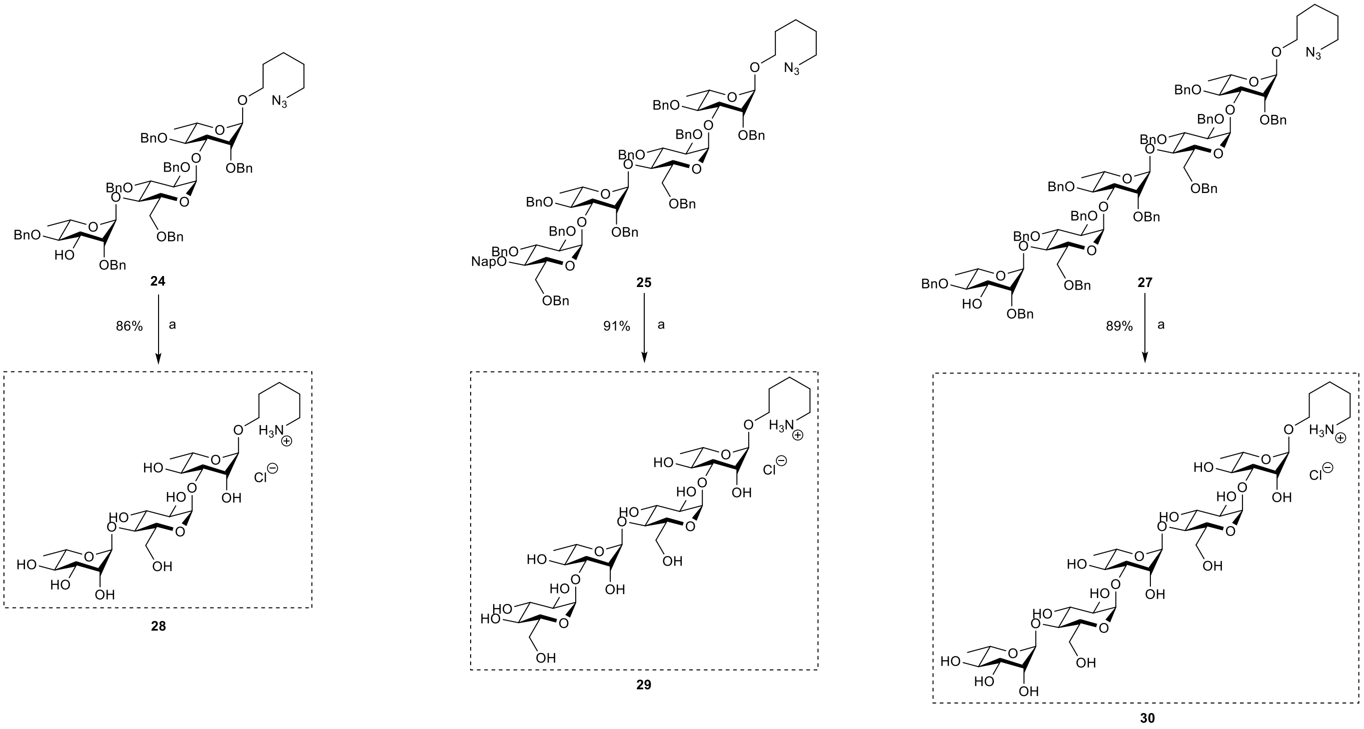

Supplementary Figure 22. Oligosaccharide **28 - 30** synthesis. [a] Pd/C, H_2, THF, ^{t}BuOH, PBS (pH 4).

5-Aminopentyl [α-L-rhamnopyranosyl]-(1→4)-[α-D-glucopyranosyl]-(1→3)-α-L-rhamnopyranoside HCl salt (28)

Trisaccharide **24** (55 mg, 45 µmol, 1.0 eq) was dissolved in THF:^{t}BuOH:PBS buffer pH4 (4.0 mL, 6:1:3 v/v). The solution was purged with argon. Pd/C (Evonik Noblyst, 0.30 g, 10% wt) was suspended in DMF:H_2O (1.0 mL, 4:1 v/v). Concentrated HCl (0.20 mL) was added, after which the suspension was stirred for 15 min. The catalyst was filtered, washed with mQ H_2O and added to the trisaccharide solution. The mixture was purged with H_2 and subsequently stirred vigorously, under a H_2 atmosphere, for 24 hrs. The mixture was filtered over celite. The celite was washed with mQ H_2O, after which the filtrate was evaporated *in vacuo*. The residue was purified using size-exclusion chromatography (Biorad Bio-gel P2 support), yielding deprotected trisaccharide **28** as a white solid (22 mg, 39 µmol, 86%).

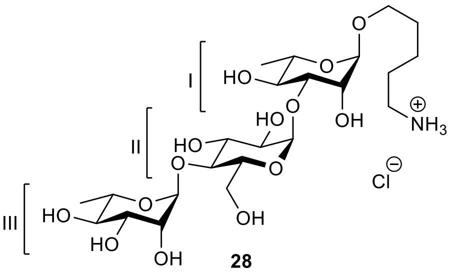

28

TLC: (H_2O:ACN, 20:80 v/v): R_f = 0.0; **^{1}H NMR** (500 MHz, D_2O; solvent peak ref'd to 4.79): δ 5.09 (d, *J* = 3.8 Hz, 1H, ***H-1^{II}***), 4.90 (d, *J* = 1.8 Hz, 1H, ***H-1^{III}***), 4.87 (d, *J* = 1.9 Hz, 1H, ***H-1^{I}***), 4.15 (dd, *J* = 3.2, 2.0 Hz, 1H, ***H-2^{I}***), 4.11 – 4.03 (m, 2H, ***H-5^{II}***, ***H-5^{III}***), 4.01 (dd, *J* = 3.4, 1.8 Hz, 1H, ***H-2^{III}***), 3.88 (t, *J* = 9.5 Hz, 1H, ***H-3^{II}***), 3.84 – 3.80 (m, 2H, ***H-3^{I}***, ***H-6$_a^{II}$***), 3.80 – 3.71 (m, 4H, ***H-3^{III}***, ***H-5^{I}***, ***H-6$_b^{II}$***, OC$\boldsymbol{H_a}$H$_b$CH$_2$), 3.66 – 3.54 (m, 4H, ***H-2^{II}***, ***H-4^{I}***, ***H-4^{II}***, OCH$_a$$\boldsymbol{H_b}CH_2$), 3.48 (t, *J* = 9.7 Hz, 1H, ***H-4^{III}***), 3.03 (dd, *J* = 8.6, 6.7 Hz, 2H, C$\boldsymbol{H_2}$N$^+$H$_3$), 1.77 – 1.65 (m, 4H, C$\boldsymbol{H_2}$CH$_2$N$^+$H$_3$, OCH$_2$C$\boldsymbol{H_2}$), 1.53 – 1.43 (m, 2H, C$\boldsymbol{H_2}$CH$_2$CH$_2$N$^+$H$_3$), 1.33 (d, *J* = 6.4 Hz, 3H, 6^{I}-C$\boldsymbol{H_3}$), 1.29 (d, *J* = 6.3 Hz, 3H, 6^{III}-C$\boldsymbol{H_3}$); **^{13}C NMR** (126 MHz, D_2O): δ 100.91 (***C-1^{III}***), 99.39 (***C-1^{I}***), 95.57 (***C-1^{II}***), 77.25 (***C-4^{II}***), 75.95 (***C-3^{I}***), 72.00 (***C-4^{III}***), 71.69 (***C-2^{II}***), 71.65 (***C-3^{II}***), 70.85 (***C-5^{II}***), 70.50 (***C-2^{III}***), 70.32 (***C-4^{I}***), 70.28 (***C-3^{III}***), 69.17 (***C-5^{III}***), 68.80 (***C-5^{I}***), 67.59 (-O**C**H$_2$CH$_2$-), 66.99 (***C-2^{I}***), 59.98 (***C-6^{II}***), 39.47 (**C**H$_2$N$^+$H$_3$), 28.10 (OCH$_2$**C**H$_2$), 26.60 (**C**H$_2$CH$_2$N$^+$H$_3$), 22.52 (**C**H$_2$CH$_2$CH$_2$N$^+$H$_3$), 16.77 (***C-6^{I}***), 16.56 (***C-6^{III}***); **HR-ESI-TOF/MS (m/z):** [M+H]$^+$ calcd. for $C_{23}H_{44}N_1O_{16}$, 558.27618; found, 558.27548.

5-Aminopentyl [α-D-glucopyranosyl]-(1→3)-[α-L-rhamnopyranosyl]-(1→4)-[α-D-glucopyranosyl]-(1→3)-α-L-rhamnopyranoside HCl salt (29)

Tetrasaccharide **25** (88 mg, 49 µmol, 1.0 eq) was dissolved in THF:^{t}BuOH:PBS buffer pH4 (5.0 mL, 6:1:3 v/v). The solution was purged with argon. Pd/C (Evonik Noblyst, 300 mg, 10% wt) was suspended in DMF:H_2O (1.0 mL, 4:1 v/v). Concentrated HCl (0.20 mL) was added, after which the suspension was stirred for 15 min. The catalyst was filtered, washed with mQ H_2O and added to the tetrasaccharide solution. The mixture was purged with H_2 and subsequently stirred vigorously, under a H_2 atmosphere, for 114 hrs. The mixture was filtered over celite. The celite was washed with mQ H_2O, after which the filtrate was evaporated *in vacuo*. The residue was purified using size-exclusion chromatography (Biorad Bio-gel P2 support), yielding deprotected tetrasaccharide **29** as a white solid (32 mg, 44 µmol, 91%).

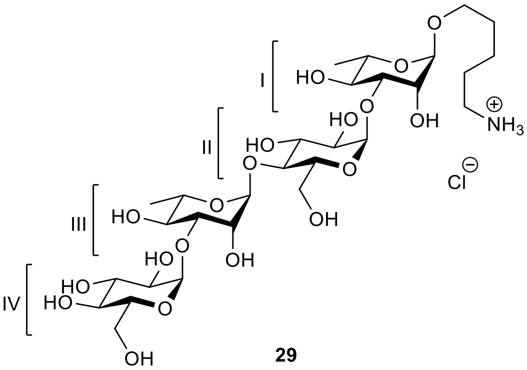

29

TLC: (H_2O:ACN, 20:80 v/v): R_f = 0.0; **^{1}H NMR** (500 MHz, D_2O; solvent peak ref'd to 4.79): δ 5.10 – 5.07 (m, 2H, ***H-1^{II}***, ***H-1^{IV}***), 4.95 (d, *J* = 2.0 Hz, 1H, ***H-1^{III}***), 4.86 (d, *J* = 1.9 Hz, 1H, ***H-1^{I}***), 4.21 (t, *J* = 2.6 Hz, 1H, ***H-2^{III}***), 4.15 (t, *J* = 2.5 Hz, 1H, ***H-2^{I}***), 4.12 – 4.06 (m, 2H, ***H-5^{II}***, ***H-5^{III}***), 3.99 (dt, *J* = 10.2, 3.4 Hz, 1H, ***H-5^{IV}***), 3.90 – 3.71 (m, 10H, ***H-3^{I}***, ***H-3^{II}***, ***H-3^{III}***, ***H-3^{IV}***, ***H-5^{I}***, ***H-6$_a^{II}$***, ***H-6$_b^{II}$***, ***H-6$_a^{IV}$***, ***H-6$_b^{IV}$***, OC$\boldsymbol{H_a}$H$_b$CH$_2$), 3.66 – 3.54 (m, 6H, ***H-2^{II}***, ***H-2^{IV}***, ***H-4^{I}***, ***H-4^{II}***, ***H-4^{III}***, OCH$_a$$\boldsymbol{H_b}CH_2$), 3.49 (t, *J* = 9.6 Hz, 1H, ***H-4^{IV}***), 3.02 (t, *J* = 7.6 Hz, 2H, C$\boldsymbol{H_2}$N$^+$H$_3$), 1.76 – 1.64 (m, 4H, C$\boldsymbol{H_2}$CH$_2$N$^+$H$_3$, OCH$_2$C$\boldsymbol{H_2}$), 1.53 – 1.42 (m, 2H, C$\boldsymbol{H_2}$CH$_2$CH$_2$N$^+$H$_3$), 1.33 (d, *J* = 6.3 Hz, 3H, 6^{I}-C$\boldsymbol{H_3}$), 1.31 (d, *J* = 6.3 Hz, 3H, 6^{III}-C$\boldsymbol{H_3}$); **^{13}C NMR** (126 MHz, D_2O): δ 100.46 (***C-1^{III}***), 99.36 (***C-1^{I}***), 95.62 (***C-1^{IV}***), 95.56 (***C-1^{II}***), 77.22 (***C-4^{II}***), 75.94 (***C-3^{I}***), 75.56 (***C-3^{III}***), 72.97 (***C-3^{IV}***), 71.71 (2C, ***C-2^{II}***, ***C-5^{IV}***), 71.63 (***C-3^{II}***), 71.46 (***C-2^{IV}***), 70.81 (***C-5^{II}***), 70.28 (***C-4^{I}***), 70.21 (***C-4^{III}***), 69.37 (***C-4^{IV}***), 69.25 (***C-5^{III}***), 68.78 (***C-5^{I}***), 67.57 (O**C**H$_2$CH$_2$), 67.17 (***C-2^{III}***), 66.98 (***C-2^{I}***), 60.25 (***C-6^{IV}***), 59.95 (***C-6^{II}***), 39.44 (**C**H$_2$N$^+$H$_3$), 28.08 (OCH$_2$**C**H$_2$), 26.59 (**C**H$_2$CH$_2$N$^+$H$_3$), 22.51 (**C**H$_2$CH$_2$CH$_2$N$^+$H$_3$), 16.74 (***C-6^{I}***), 16.66 (***C-6^{III}***); **HR-ESI-TOF/MS (m/z):** [M+H]$^+$ calcd. for $C_{29}H_{54}N_1O_{19}$, 720.32900; found, 720.32780.

5-Aminopentyl [α-L-rhamnopyranosyl]-(1→4)-[α-D-glucopyranosyl]-(1→3)-[α-L-rhamnopyranosyl]-(1→4)-[α-D-glucopyranosyl]-(1→3)-α-L-rhamnopyranoside HCl salt (30)

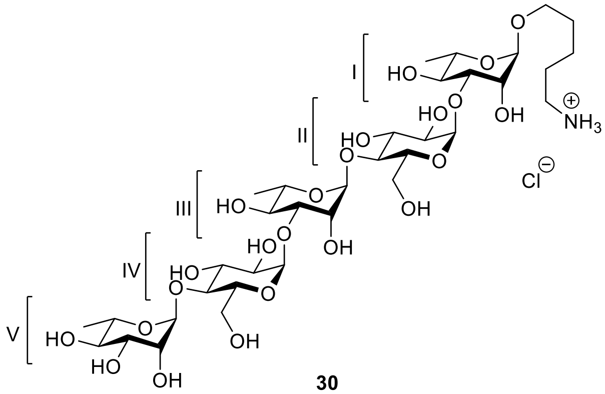

30

Pentasaccharide **27** (62 mg, 31 µmol, 1.0 eq) was dissolved in THF:^{t}BuOH:PBS buffer pH4 (6.0 mL, 8:1:3 v/v). The solution was purged with argon. Pd/C (Evonik Noblyst, 200 mg, 10% wt) was suspended in DMF:H_2O (1.0 mL, 4:1 v/v). Concentrated HCl (0.20 mL) was added, after which the suspension was stirred for 15 min. The catalyst was filtered, washed with mQ H_2O and added to the pentasaccharide solution. The mixture was purged with H_2 and subsequently stirred vigorously, under a H_2 atmosphere, for 70 hrs. The mixture was filtered over celite. The celite was washed with mQ H_2O, after which the filtrate was evaporated *in vacuo*. The residue was purified using size-exclusion chromatography (Biorad Bio-gel P2 support), yielding deprotected pentasaccharide **30** as a white solid (24 mg, 28 µmol, 89%).

TLC: (H_2O:ACN, 20:80 v/v): R_f = 0.00; **^{1}H NMR** (500 MHz, D_2O; solvent peak ref'd to 4.79): δ 5.11 – 5.07 (m, 2H, ***H-1***^{II}, ***H-1***^{IV}), 4.95 (d, *J* = 1.8 Hz, 1H, ***H-1***^{III}), 4.91 (d, *J* = 1.7 Hz, 1H, ***H-1***^{V}), 4.87 (d, *J* = 1.9 Hz, 1H, ***H-1***^{I}), 4.21 (t, *J* = 2.5 Hz, 1H, ***H-2***^{III}), 4.15 (t, *J* = 2.5 Hz, 1H, ***H-2***^{I}), 4.13 – 4.03 (m, 4H, ***H-5***^{II}, ***H-5***^{III}, ***H-5***^{IV}, ***H-5***^{V}), 4.01 (t, *J* = 2.6 Hz, 1H, ***H-2***^{V}), 3.92 – 3.85 (m, 2H, ***H-3***^{II}, ***H-3***^{IV}), 3.86 – 3.71 (m, 9H, ***H-3***^{I}, ***H-3***^{III}, ***H-3***^{V}, ***H-5***^{I}, ***H-6a***^{II}, ***H-6b***^{II}, ***H-6a***^{IV}, ***H-6b***^{IV}, $OCH_aH_bCH_2$), 3.67 – 3.54 (m, 7H, ***H-2***^{II}, ***H-2***^{IV}, ***H-4***^{I}, ***H-4***^{II}, ***H-4***^{III}, ***H-4***^{IV}, $OCH_aH_bCH_2$), 3.48 (t, *J* = 9.7 Hz, 1H, ***H-4***^{V}), 3.03 (t, *J* = 7.6 Hz, 2H, $CH_2N^+H_3$), 1.77 – 1.63 (m, 4H, $CH_2CH_2N^+H_3$, OCH_2CH_2), 1.54 – 1.42 (m, 2H, $CH_2CH_2CH_2N^+H_3$), 1.33 (d, *J* = 6.3 Hz, 3H, 6^{I}-CH_3), 1.31 (d, *J* = 6.4 Hz, 3H, 6^{III}-CH_3), 1.29 (d, *J* = 6.3 Hz, 3H, 6^{V}-CH_3); **^{13}C NMR** (126 MHz, D_2O): δ 100.89 (***C-1***^{V}), 100.48 (***C-1***^{III}), 99.37 (***C-1***^{I}), 95.55 (***C-1***^{II}, ***C-1***^{IV}), 77.25 (***C-4***^{II}), 77.14 (***C-4***^{IV}), 75.93 (***C-3***^{I}), 75.73 (***C-3***^{III}), 71.98 (***C-4***^{V}), 71.69 (2C, ***C-2***^{II}, ***C-2***^{IV}), 71.64 (***C-3***^{II}, ***C-3***^{IV}), 70.82 (2C, ***C-5***^{II}, ***C-5***^{IV}), 70.48 (***C-2***^{V}), 70.31 - 70.26 (2C, ***C-4***^{I}, ***C-4***^{III}), 70.23 (***C-3***^{V}), 69.27 (***C-5***^{III}), 69.15 (***C-5***^{V}), 68.78 (***C-5***^{I}), 67.58 (OCH_2CH_2), 67.24 (C-2^{III}), 66.98 (***C-2***^{I}), 59.96 - 59.88 (2C, ***C-6***^{II}, ***C-6***^{IV}), 39.45 ($CH_2N^+H_3$), 28.09 (OCH_2CH_2), 26.59 ($CH_2CH_2N^+H_3$), 22.51 ($CH_2CH_2CH_2N^+H_3$), 16.76 (***C-6***^{I}), 16.67 (***C-6***^{III}), 16.55 (***C-6***^{V}); **HR-ESI-TOF/MS (m/z):** $[M+H]^+$ calcd. for $C_{35}H_{64}N_1O_{23}$, 866.38691; found, 866.38778.

<u>Supplementary NMR data</u>

1D Selective ^{19}F EXSY data at [$^{-}$OTf] = 0.08 M

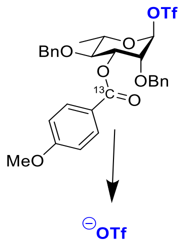

Spectrum number	Mixing Time [s]	α-triflate (-76.08 ppm) resonance intensity	$^{-}$OTf (-79.08 ppm) resonance intensity
1	0.008	14217.9	43.4886
2	0.016	13299.5	227.54
3	0.024	13225.6	354.167
4	0.032	12544.7	461.232
5	0.04	12041.3	606.699
6	0.048	11773.3	711.493
7	0.056	11392.8	828.526
8	0.064	10910.1	866.998
9	0.072	10527	1026.38
10	0.08	10185.6	1146.74

Temperature = -80 °C

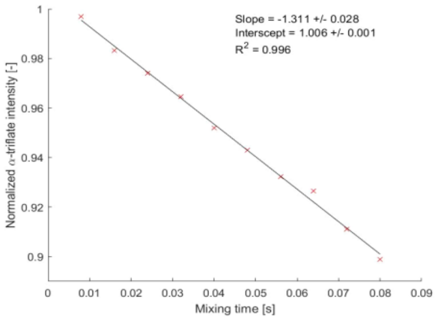

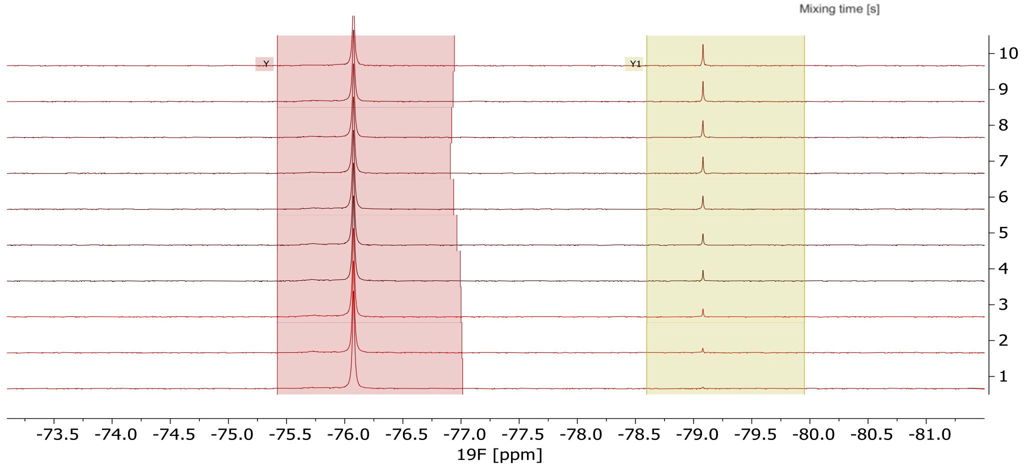

Supplementary Figure 23: EXSY data at [$^{-}$OTf] = 0.08 M.

1D Selective ^{19}F EXSY data at [$^{-}$OTf] = 0.12 M

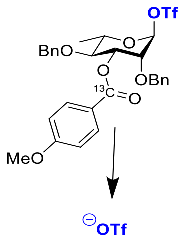

Spectrum number	Mixing Time [s]	α-triflate (-76.08 ppm) resonance intensity	$^{-}$OTf (-79.09 ppm) resonance intensity
1	0.008	25989.3	101.266
2	0.016	24449.8	143.701
3	0.024	24200.8	398.744
4	0.032	23046.4	762.021
5	0.04	22347.5	913.856
6	0.048	21617.8	1078.46
7	0.056	20824.2	1291.59
8	0.064	20076.8	1535.91
9	0.072	19275.6	1663.57
10	0.08	18666.7	1947.74

Temperature = -80 °C

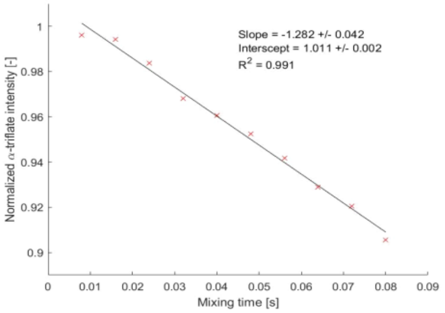

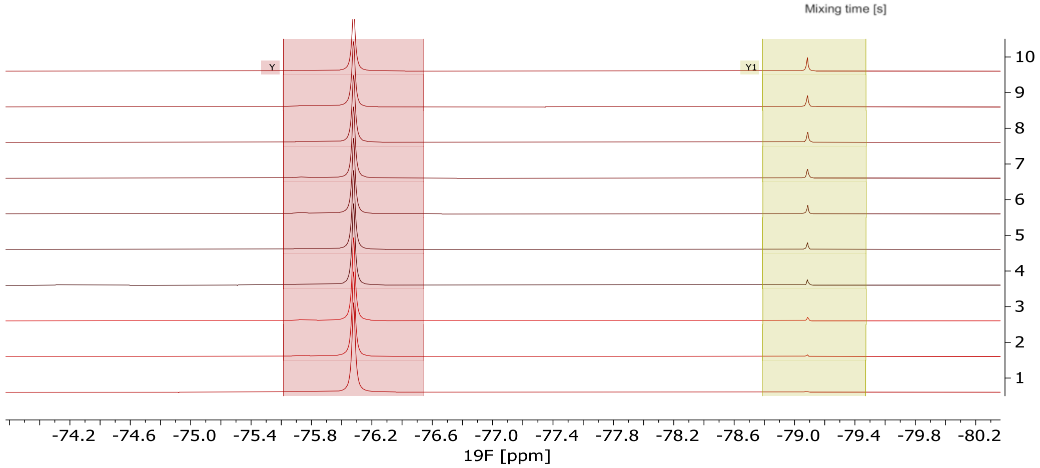

Supplementary Figure 24: EXSY data at [$^{-}$OTf] = 0.12 M.

1D Selective ^{19}F EXSY data at [$^{-}$OTf] = 0.17 M

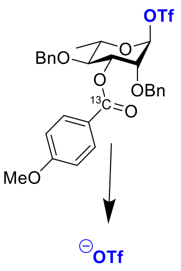

Spectrum number	Mixing Time [s]	α-triflate (-76.09 ppm) resonance intensity	$^{-}$OTf (-79.09 ppm) resonance intensity
1	0.016	21126.8	257.351
2	0.024	20798.7	385.146
3	0.032	19594.2	724.366
4	0.04	19131	888.371
5	0.056	17822.4	1381.31
6	0.064	17178.7	1535.43
7	0.072	16318.5	1747.98
8	0.08	15796.7	1948.84

Temperature = -80 °C

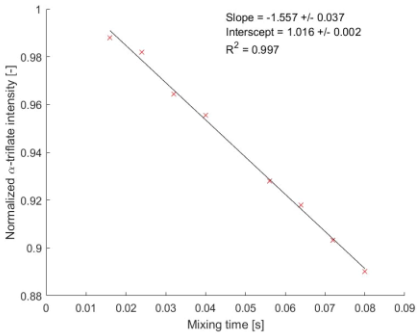

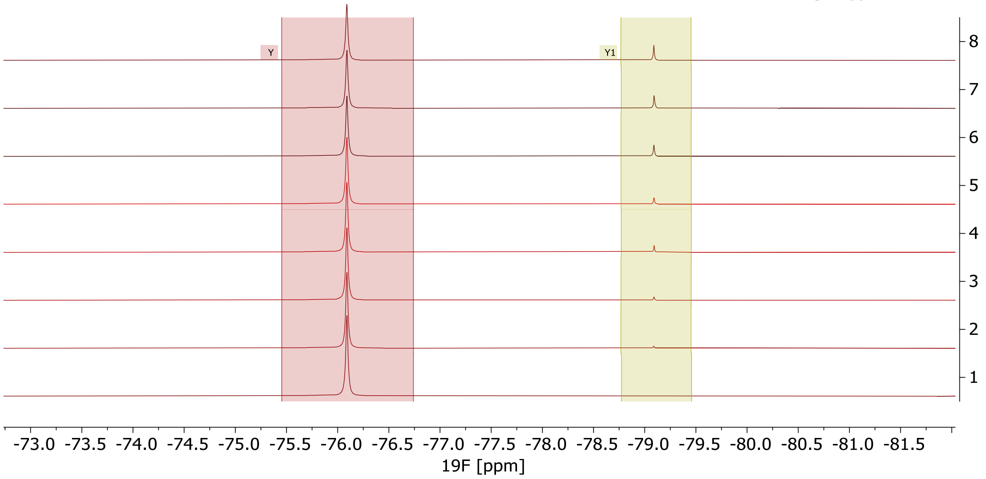

Supplementary Figure 25: EXSY data at [$^{-}$OTf] = 0.17 M.

1D Selective ^{19}F EXSY data at [$^{-}$OTf] = 0.24 M

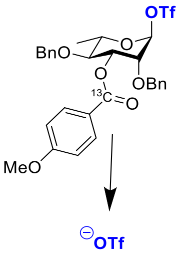

Spectrum number	Mixing Time [s]	α-triflate (-76.11 ppm) resonance intensity	$^{-}$OTf (-79.09 ppm) resonance intensity
1	0.006	16637.8	0.633379
2	0.012	16459.9	275.254
3	0.018	16023.4	275.112
4	0.024	15713.9	557.241
5	0.03	15272	695.911
6	0.036	14739.5	886.801
7	0.042	14213.9	1019.46
8	0.048	13693.3	1216.59
9	0.054	13368.1	1339.05
10	0.06	13000	1422.28

Temperature = -80 °C

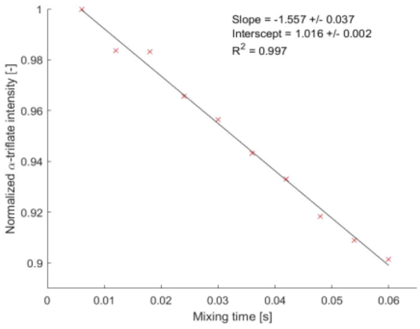

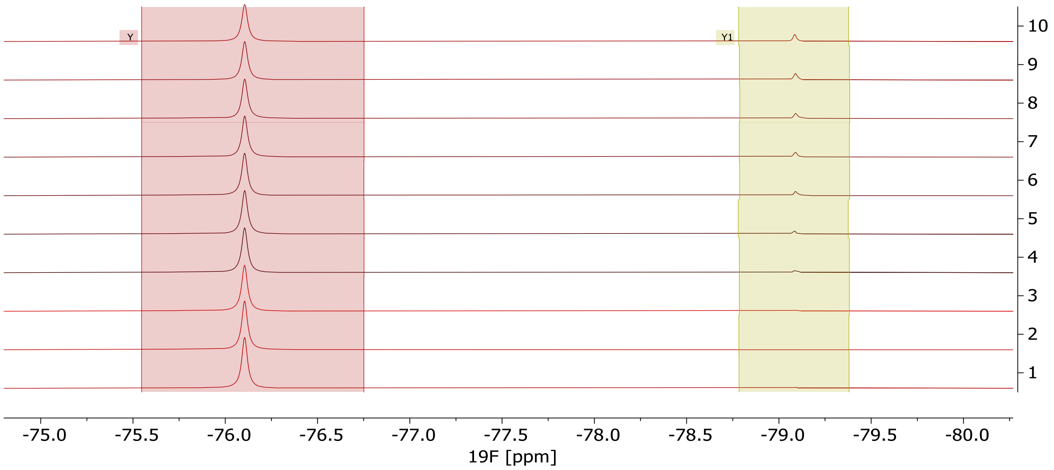

Supplementary Figure 26: EXSY data at [$^{-}$OTf] = 0.24 M.

1D Selective ^{13}C CEST data at [$^{-}$OTf] = 0.08 M

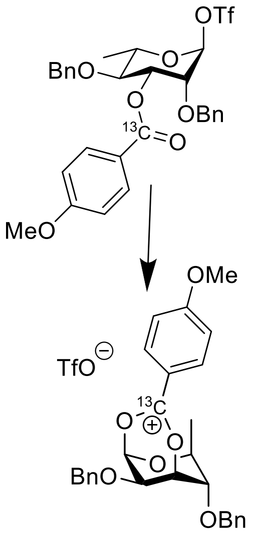

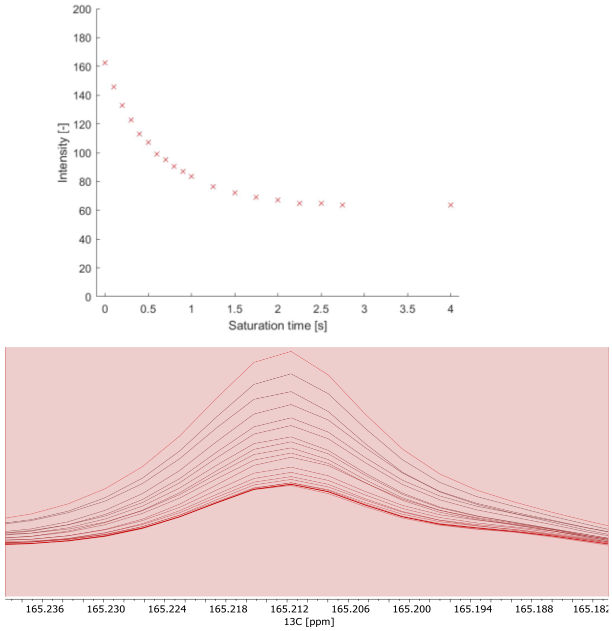

Entry	Saturation time [s]	Intensity ^{13}C=O (165 ppm)
1	0	162.476
2	0.1	145.671
3	0.2	132.846
4	0.3	122.856
5	0.4	112.924
6	0.5	107.159
7	0.6	98.8676
8	0.7	95.1117
9	0.8	90.6244
10	0.9	86.8177
11	1	83.6415
12	1.25	76.3374
13	1.5	72.284
14	1.75	69.2604
15	2	67.1716
16	2.25	64.6266
17	2.5	64.6532
18	2.75	63.8029
19	4	63.4341

Temperature = -80 °C

Supplementary Figure 27: CEST data at [$^{-}$OTf] = 0.08 M.

1D Selective ^{13}C CEST data at [$^{-}$OTf] = 0.12 M

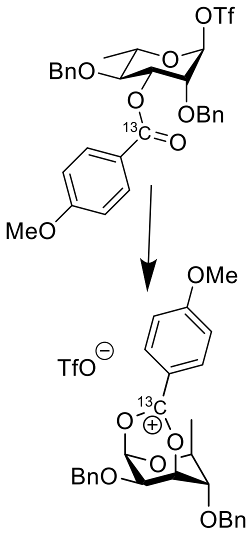

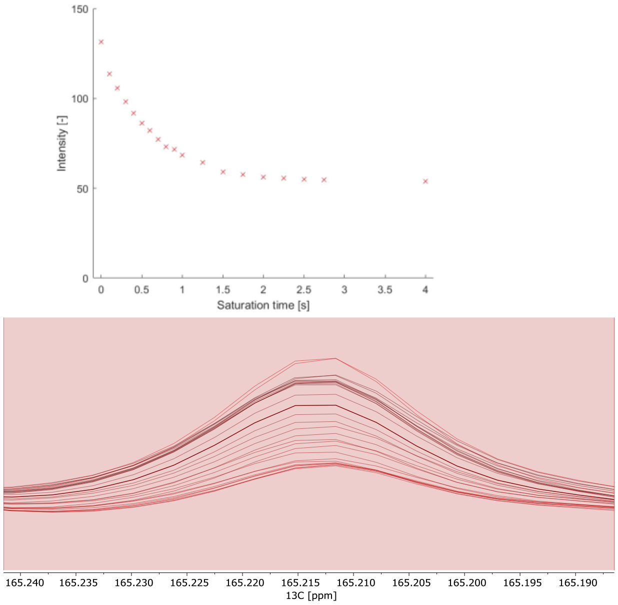

Entry	Saturation time [s]	Intensity ^{13}C=O (165 ppm)
1	0	131.457
2	0.1	113.657
3	0.2	105.818
4	0.3	98.2338
5	0.4	91.8315
6	0.5	86.3153
7	0.6	82.1893
8	0.7	77.2872
9	0.8	73.2212
10	0.9	71.6326
11	1	68.5129
12	1.25	64.2287
13	1.5	59.1381
14	1.75	57.751
15	2	56.3056
16	2.25	55.7077
17	2.5	54.9714
18	2.75	54.5737
19	4	53.9603

Temperature = -80 °C

Supplementary Figure 28: CEST data at [$^{-}$OTf] = 0.12 M.

1D Selective ^{13}C CEST data at [$^{-}$OTf] = 0.17 M

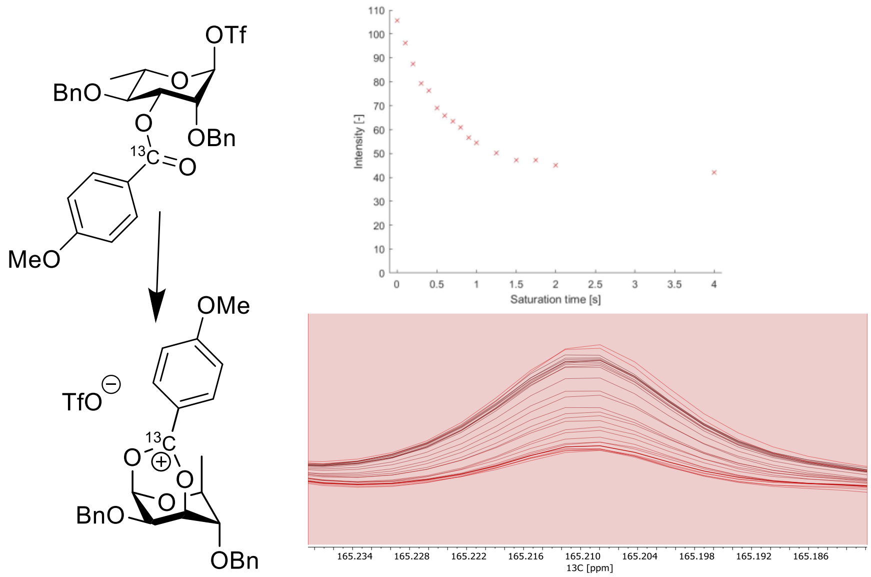

Entry	Saturation time [s]	Intensity ^{13}C=O (165 ppm)
1	0	107.261
2	0.1	96.1412
3	0.2	87.3515
4	0.3	79.3619
5	0.4	76.3545
6	0.5	69.0948
7	0.6	65.888
8	0.7	63.4971
9	0.8	60.8037
10	0.9	56.5242
11	1	54.3807
12	1.25	50.1739
13	1.5	47.1978
14	1.75	47.1098
15	2	45.0104
16	4	42.0081

Temperature = -80 °C

Supplementary Figure 29: CEST data at [$^{-}$OTf] = 0.17 M.

1D Selective ^{13}C CEST data at [$^{-}$OTf] = 0.23 M

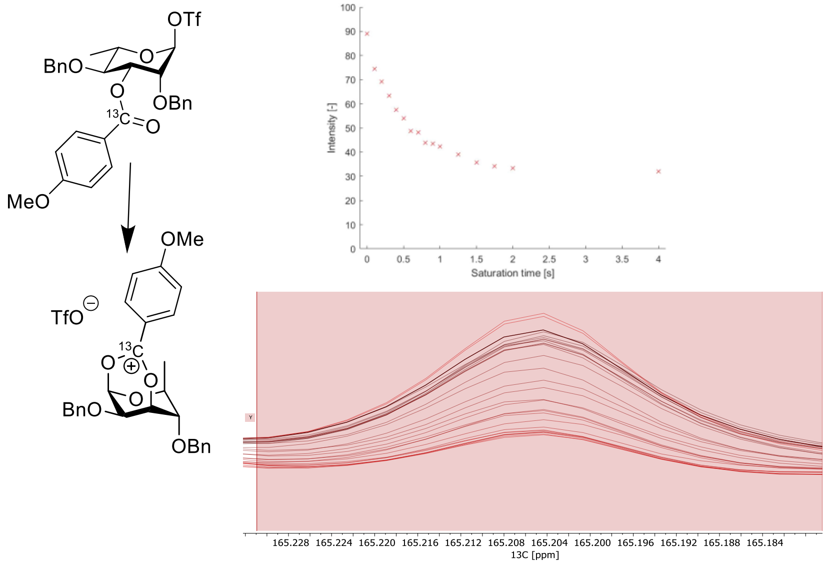

Entry	Saturation time [s]	Intensity ^{13}C=O (165 ppm)
1	0	88.9547
2	0.1	74.4011
3	0.2	69.0996
4	0.3	63.3745
5	0.4	57.5102
6	0.5	54.0396
7	0.6	48.7371
8	0.7	48.242
9	0.8	43.8162
10	0.9	43.4532
11	1	42.3805
12	1.25	39.0117
13	1.5	35.7445
14	1.75	34.1352
15	2	33.3503
16	4	32.0429

Temperature = -80 °C

Supplementary Figure 30: CEST data at [$^{-}$OTf] = 0.23 M.

Synthesis NMR spectra

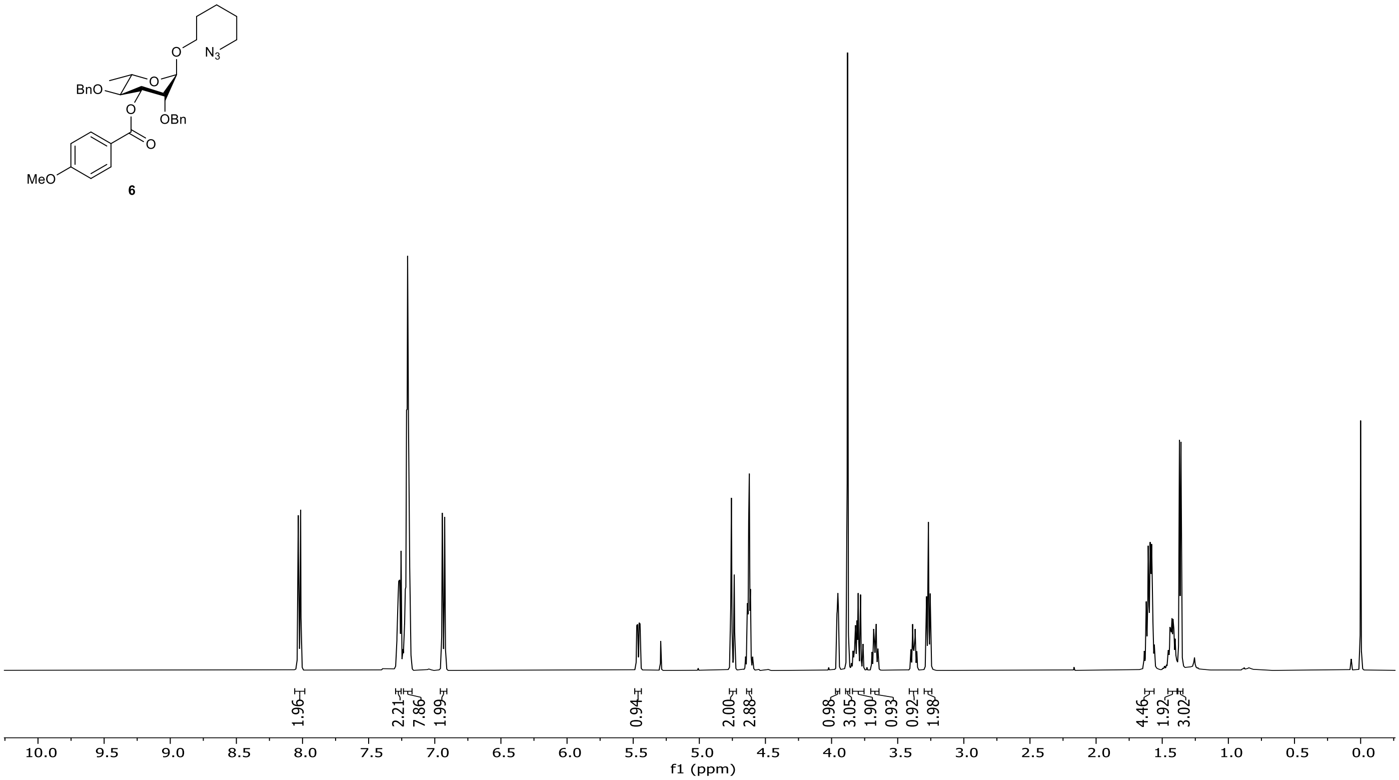

Supplementary Figure 31: ^{1}H NMR (500 MHz, $CDCl_3$) of compound **6**.

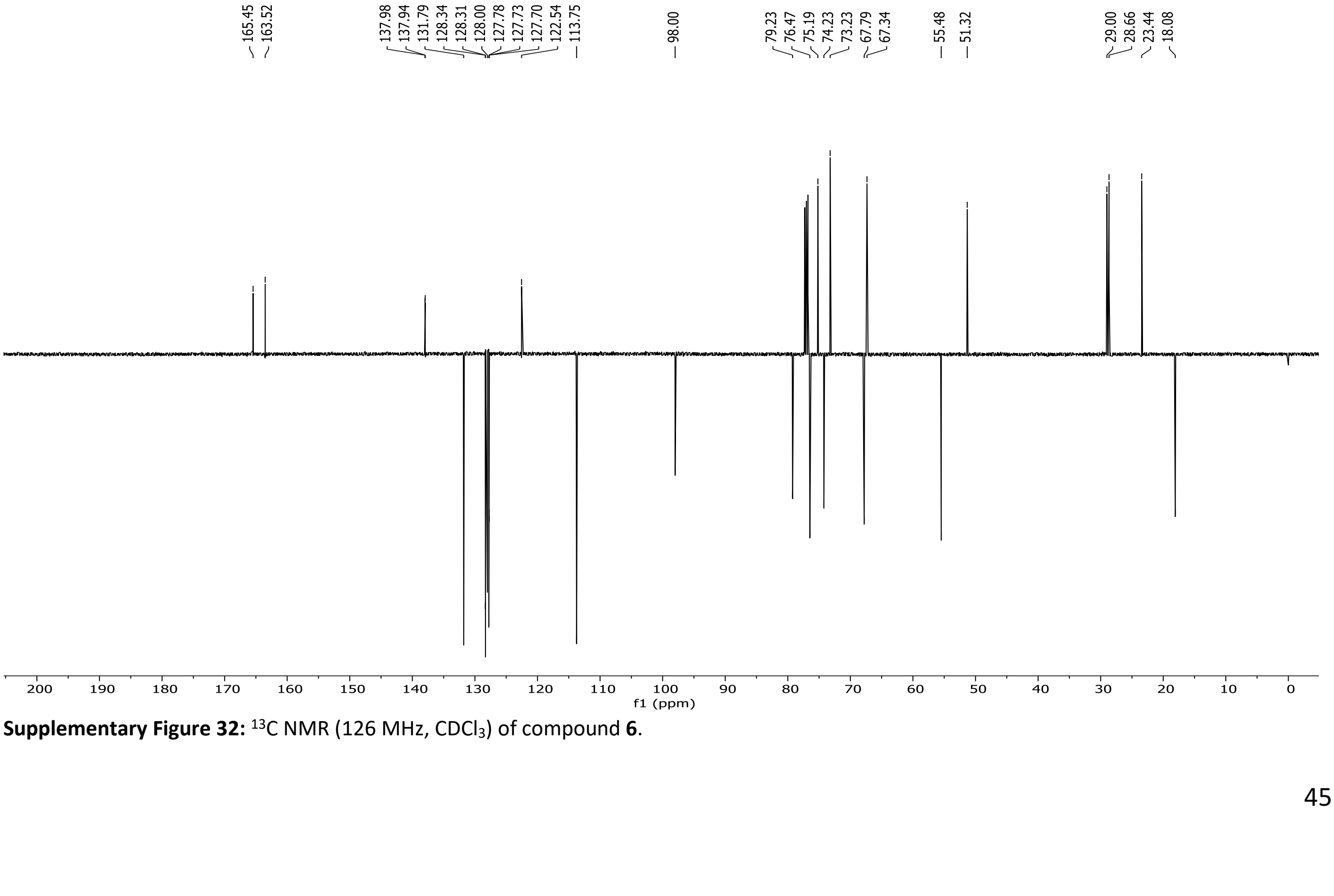

Supplementary Figure 32: ^{13}C NMR (126 MHz, $CDCl_3$) of compound **6**.

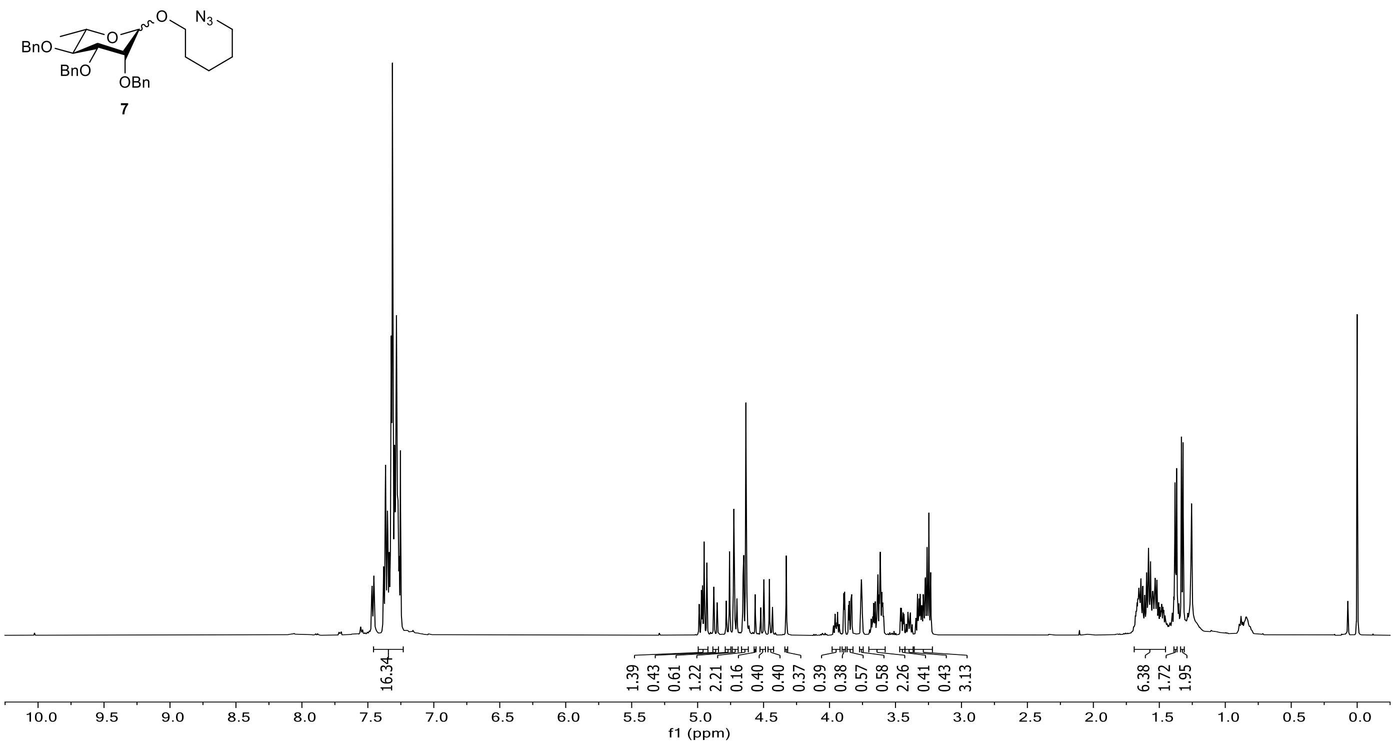

Supplementary Figure 33: ^{1}H NMR (500 MHz, $CDCl_3$) of compound **7**.

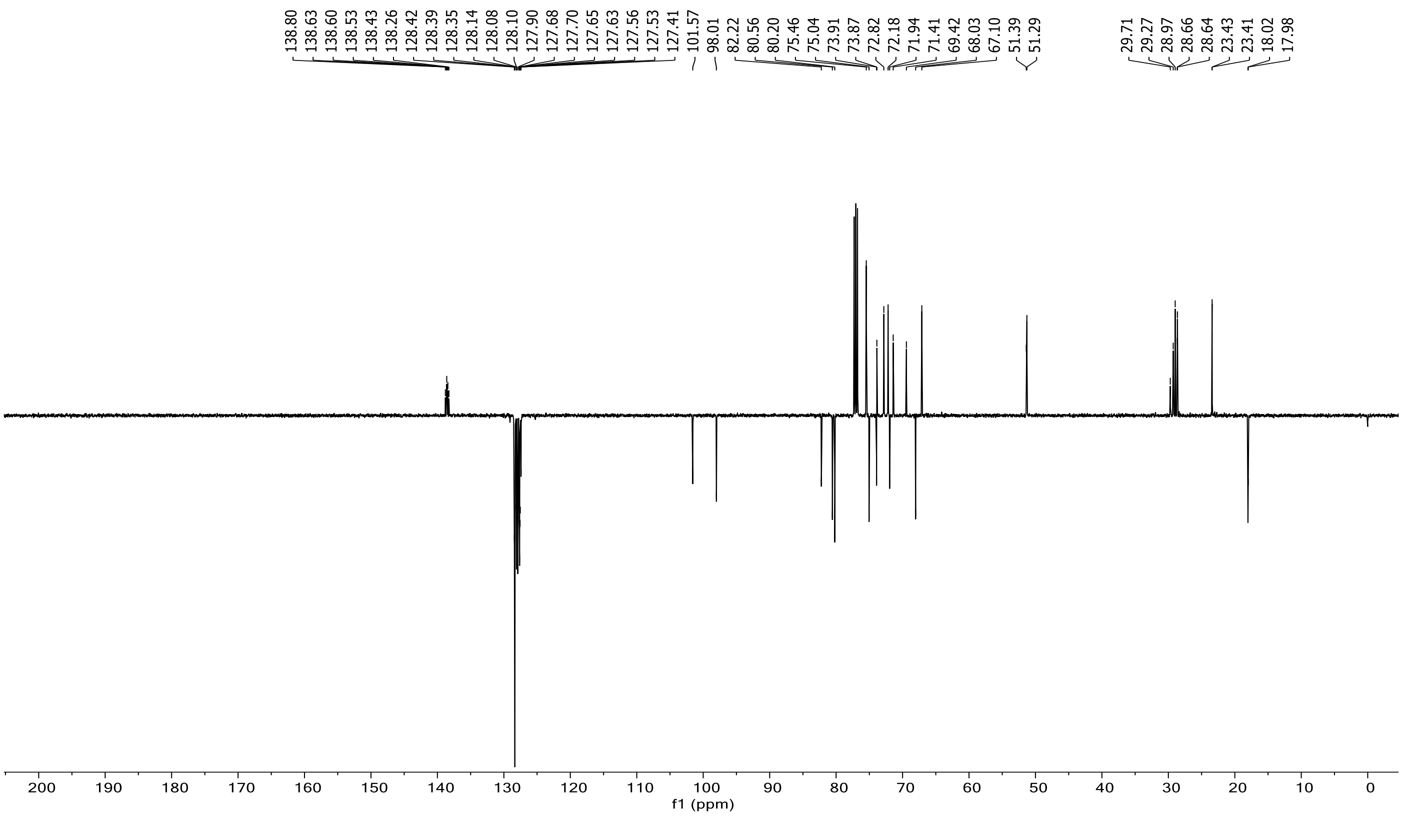

Supplementary Figure 34: ^{13}C NMR (126 MHz, $CDCl_3$) of compound **7**.

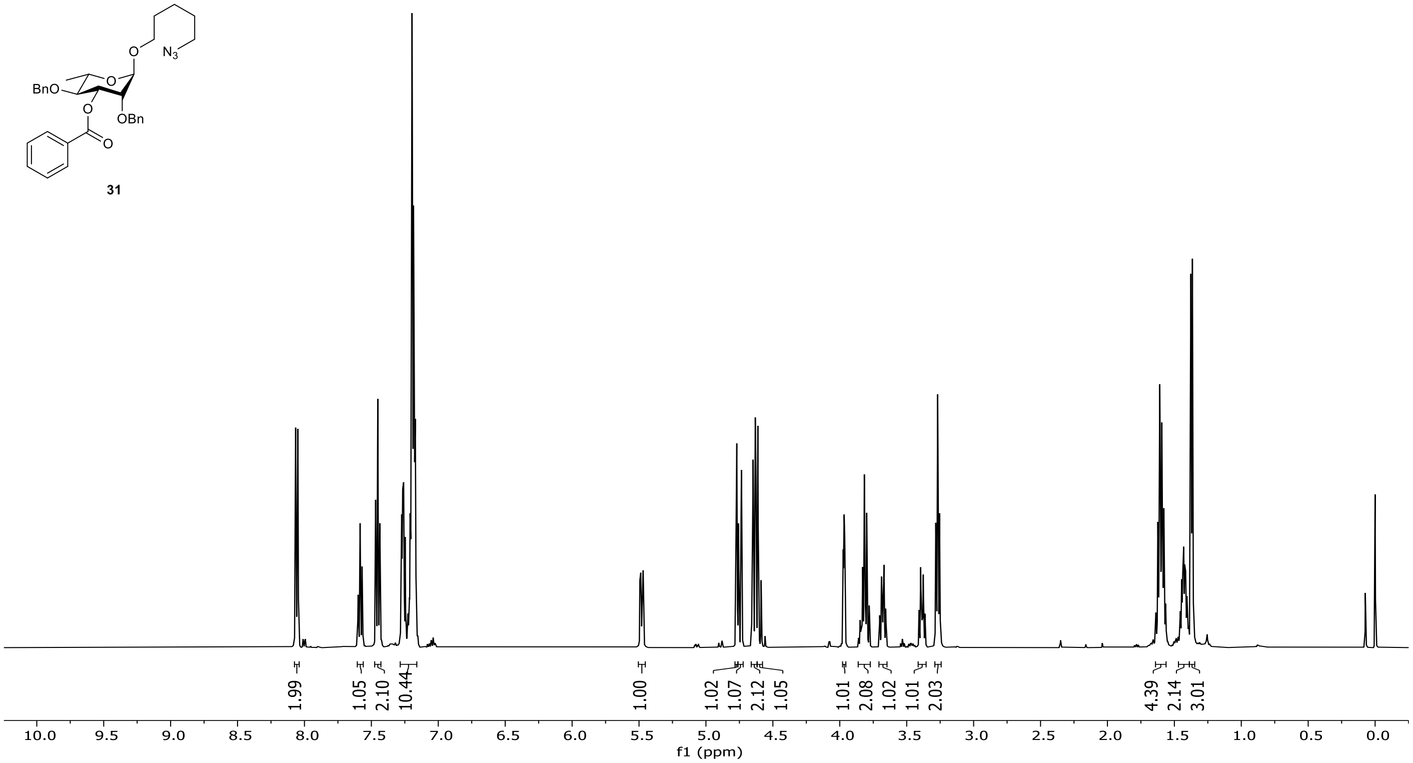

Supplementary Figure 35: ^{1}H NMR (500 MHz, $CDCl_3$) of compound **31**.

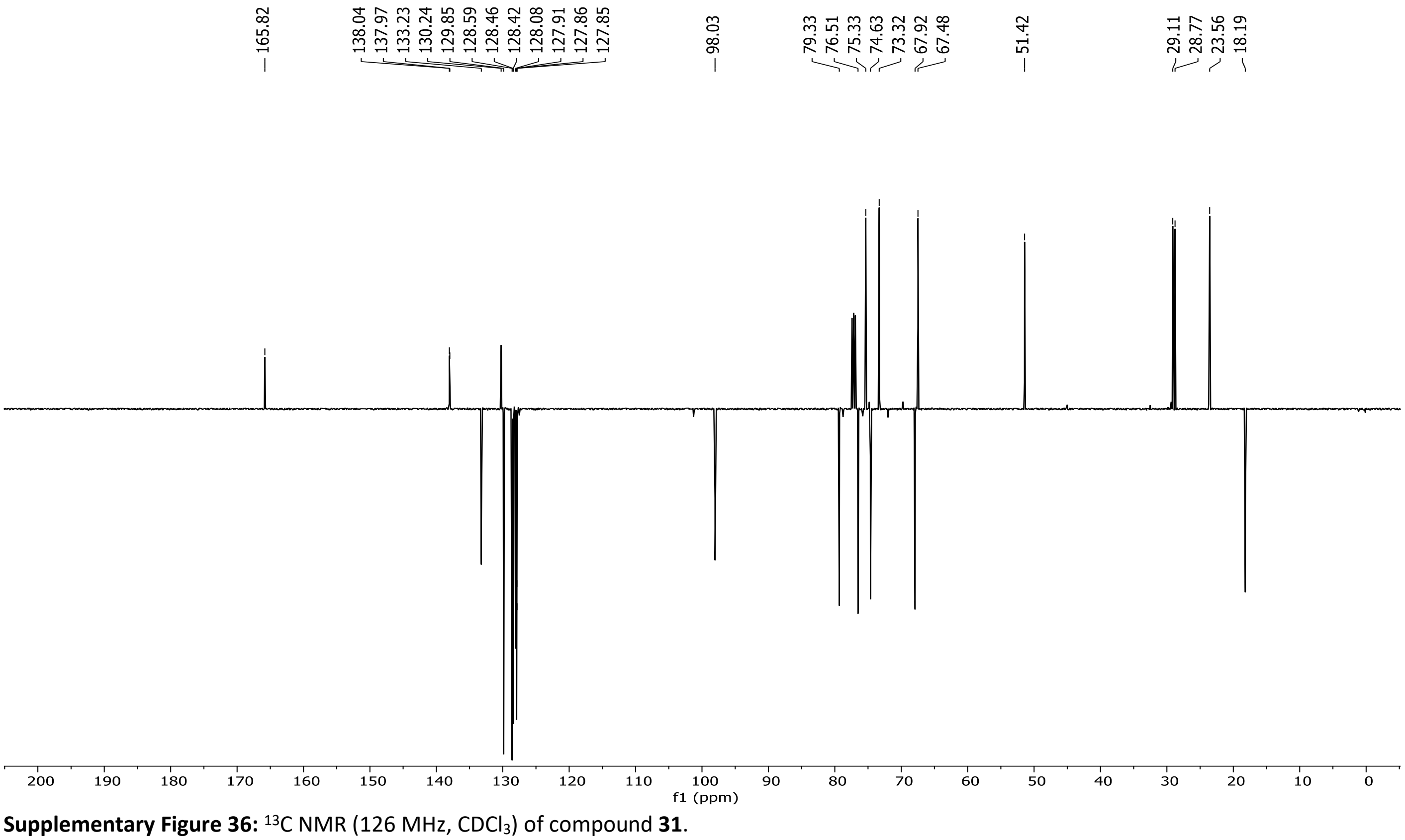

Supplementary Figure 36: ^{13}C NMR (126 MHz, $CDCl_3$) of compound **31**.

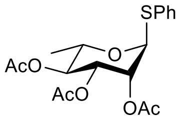

9

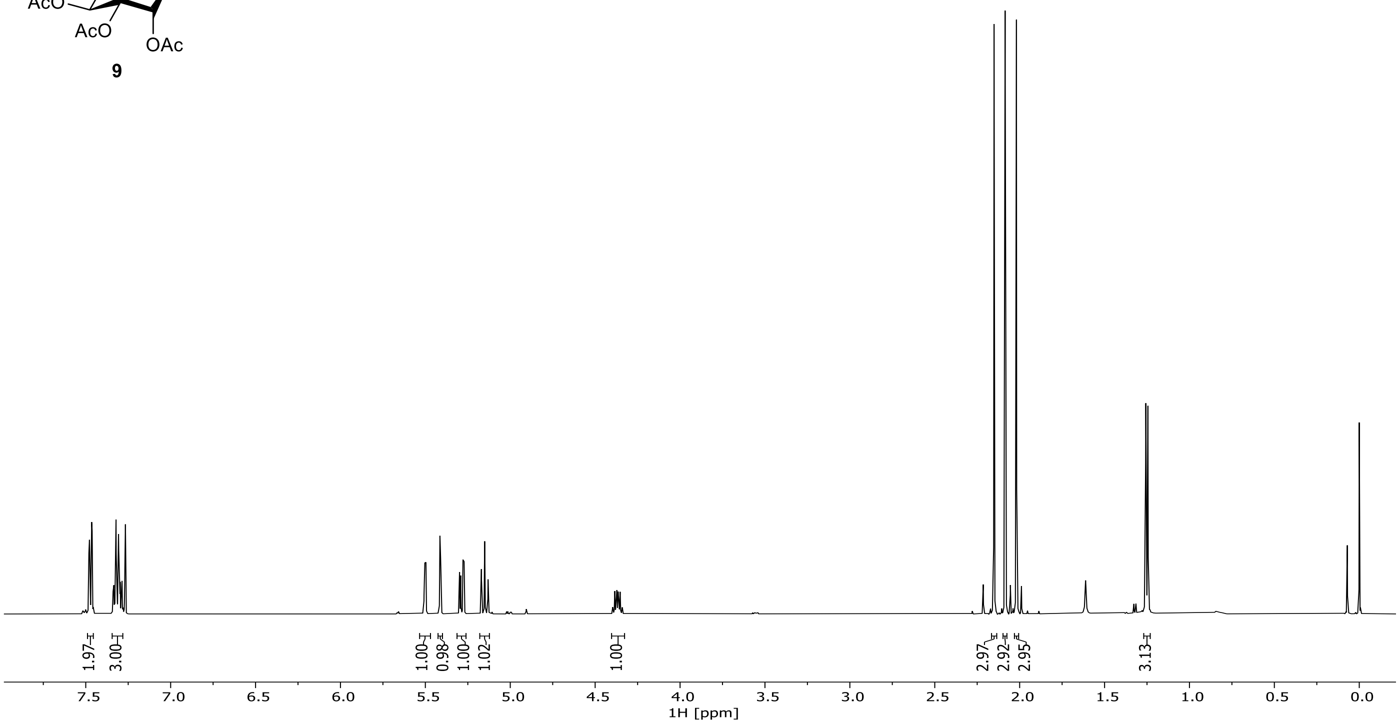

Supplementary Figure 37: ^{1}H NMR (500 MHz, $CDCl_3$) of compound **9**.

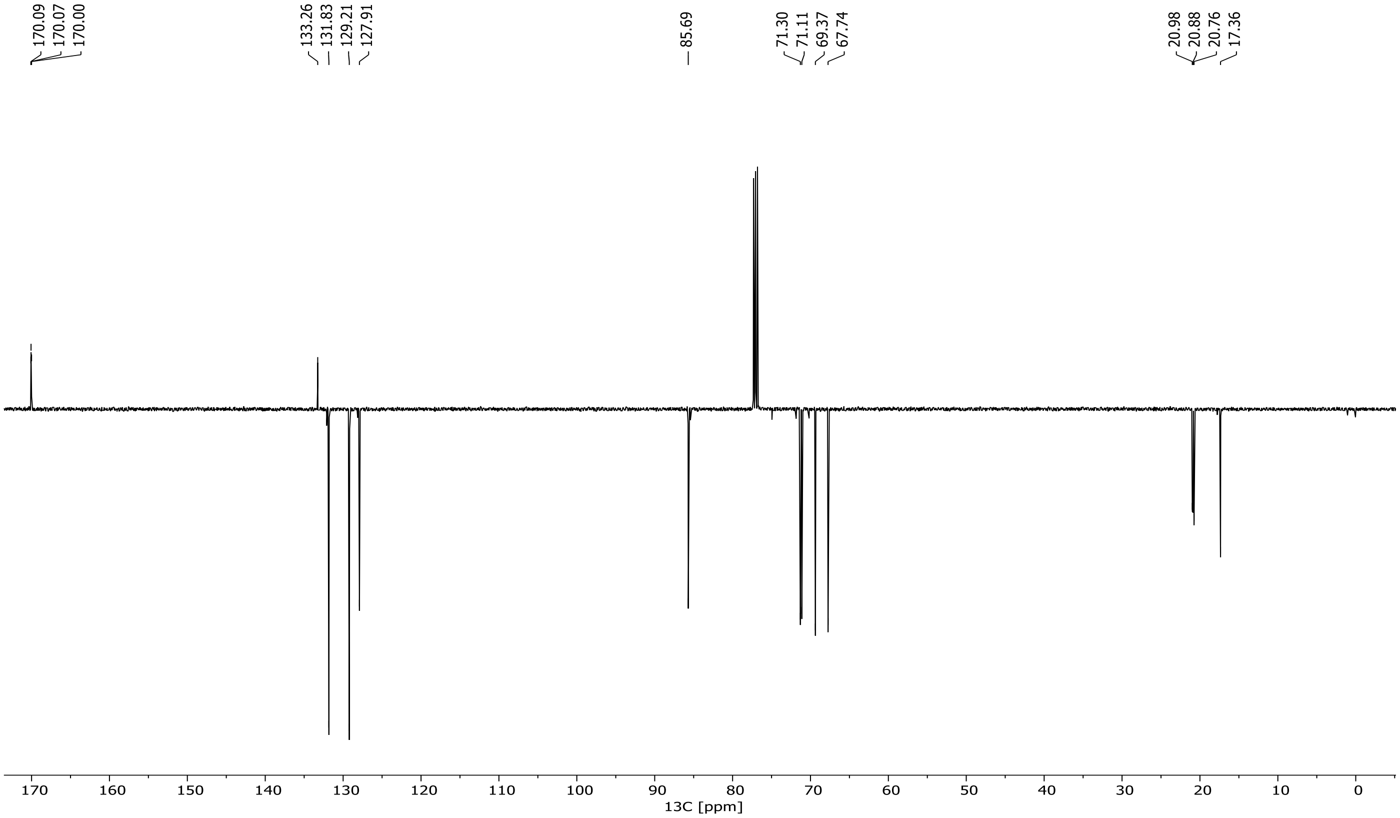

Supplementary Figure 38: ^{13}C NMR (126 MHz, $CDCl_3$) of compound **9**.

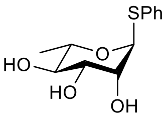

10

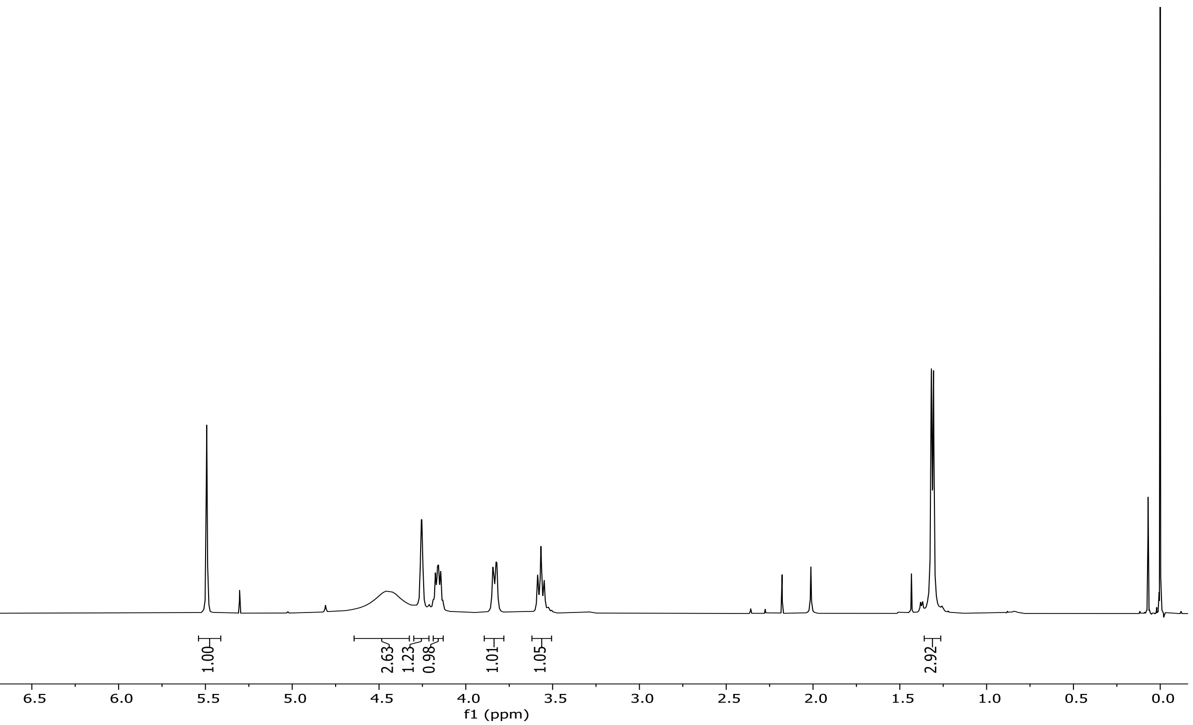

Supplementary Figure 39: ^{1}H NMR (500 MHz, $CDCl_3$) of compound **10**.

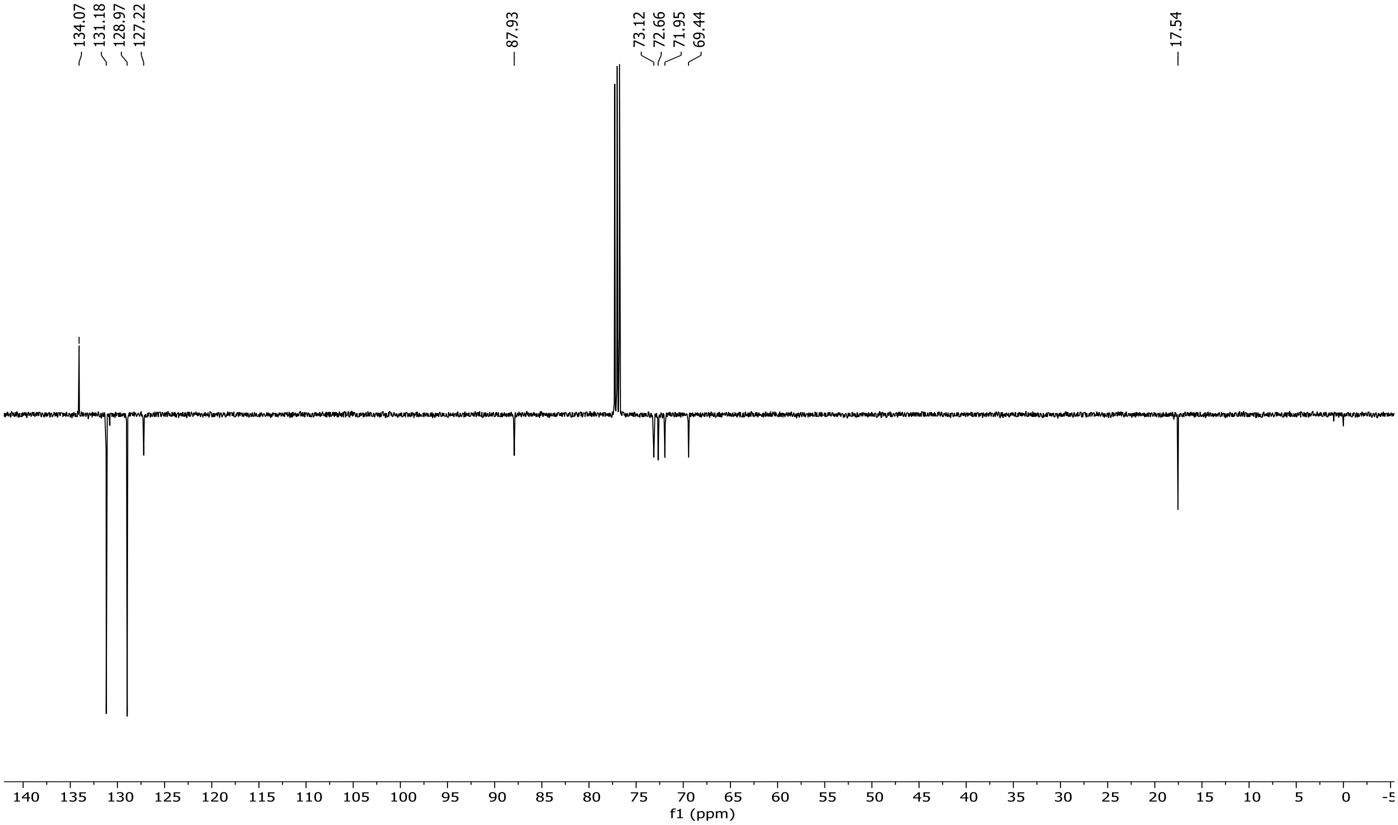

Supplementary Figure 40: ^{13}C NMR (126 MHz, $CDCl_3$) of compound **10**.

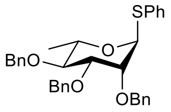

2

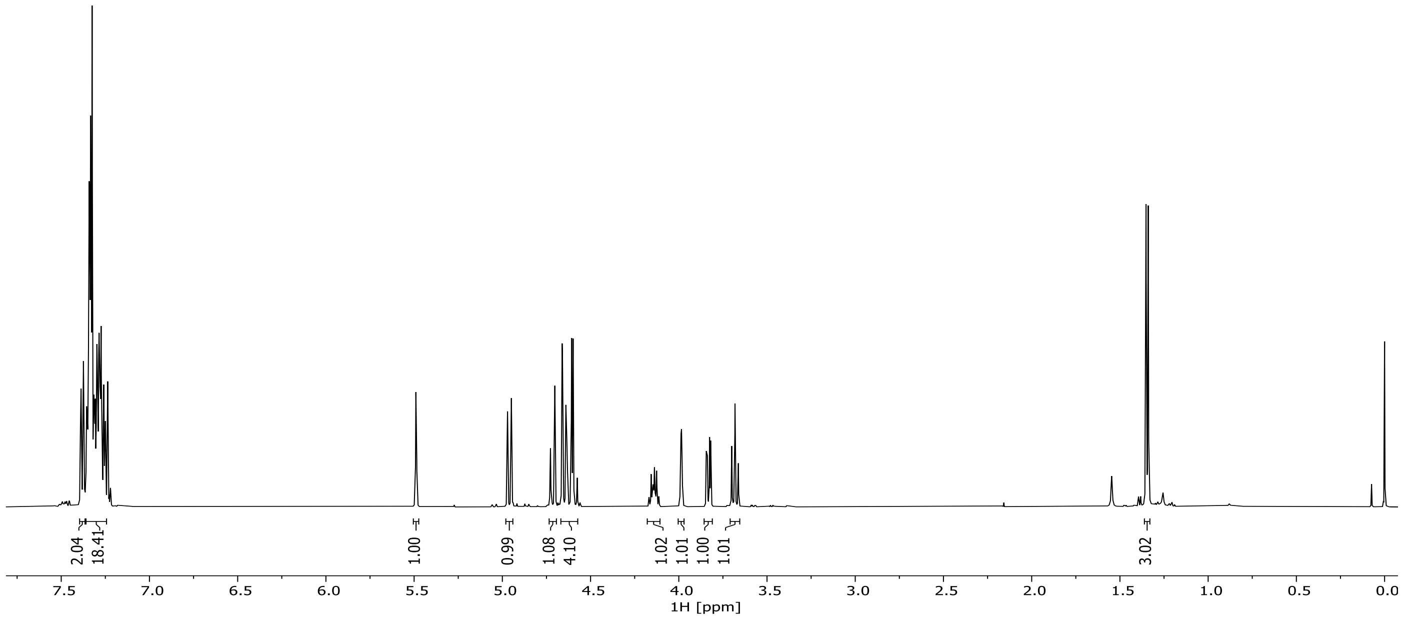

Supplementary Figure 41: ^{1}H NMR (500 MHz, $CDCl_3$) of compound **2**.

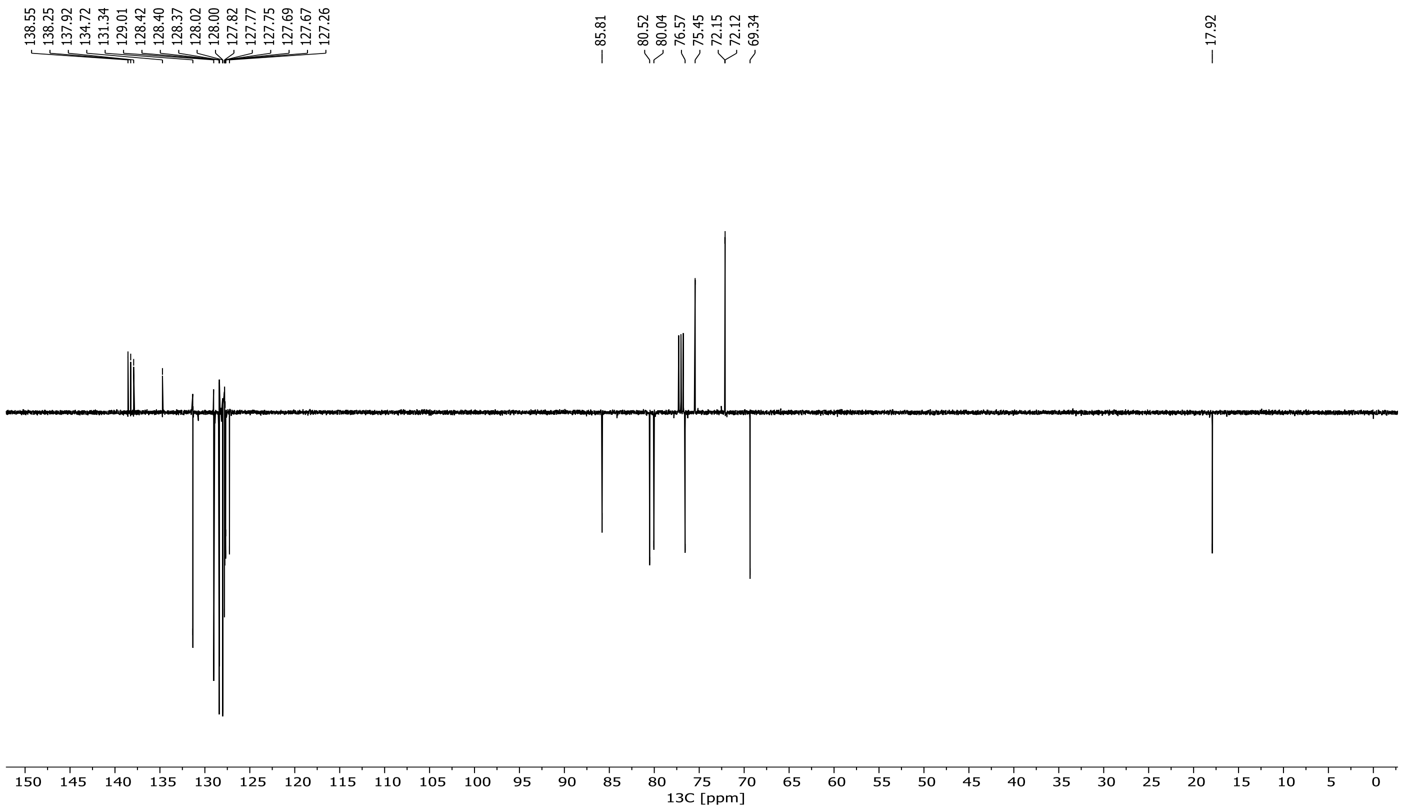

Supplementary Figure 42: ^{13}C NMR (126 MHz, $CDCl_3$) of compound **2**.

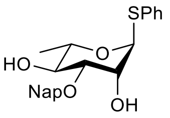

11

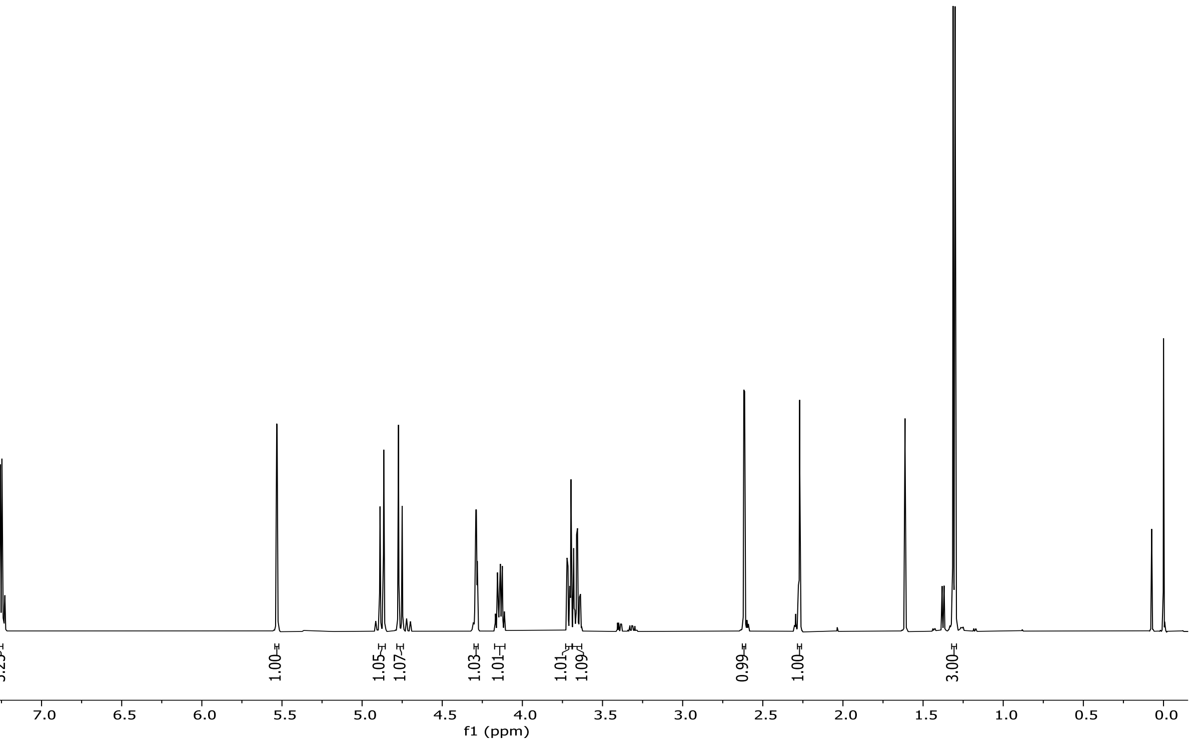

Supplementary Figure 43: ^{1}H NMR (500 MHz, $CDCl_3$) of compound **11**.

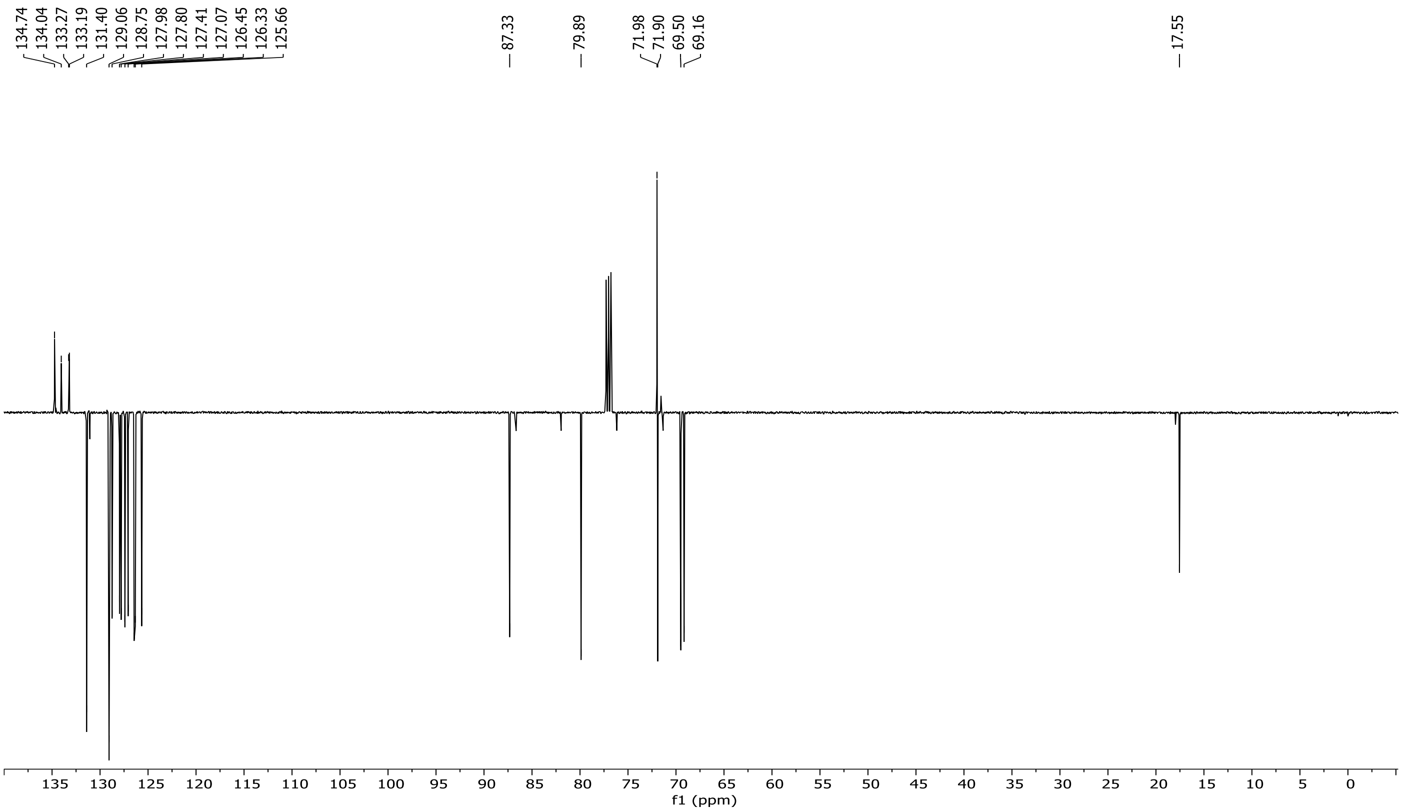

Supplementary Figure 44: ^{13}C NMR (126 MHz, $CDCl_3$) of compound **11**.

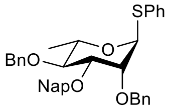

12

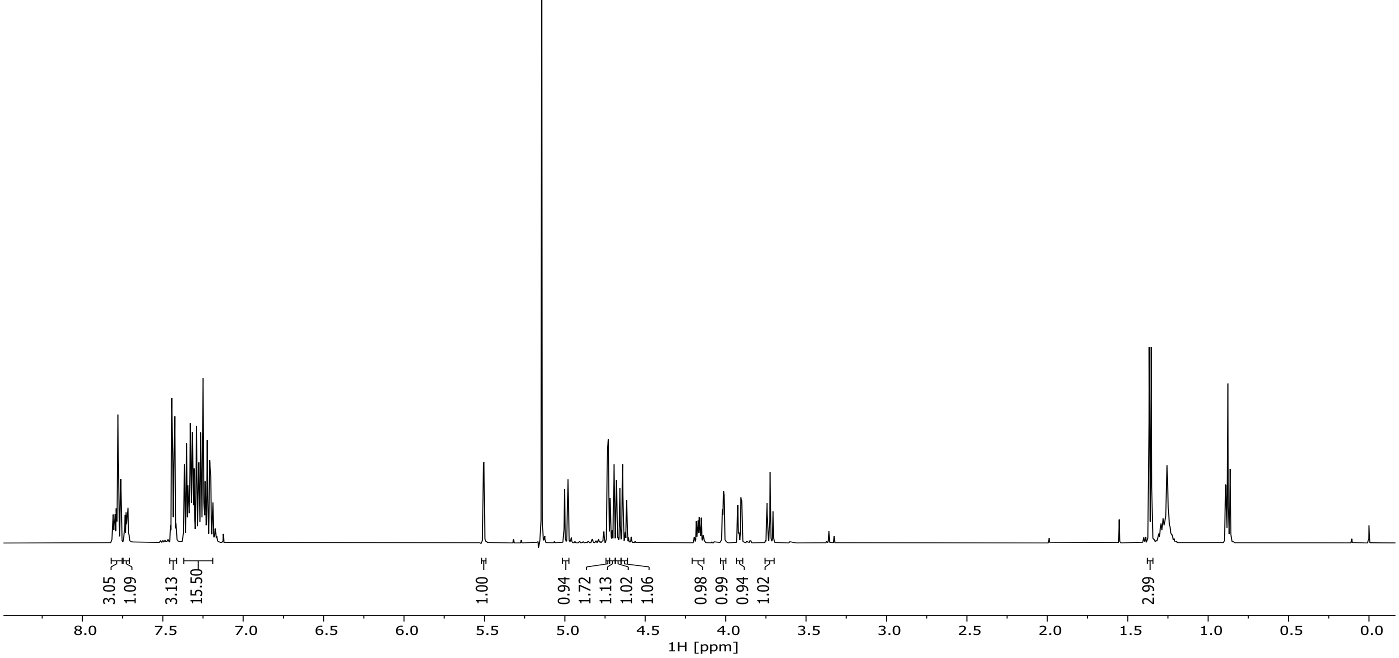

Supplementary Figure 45: ^{1}H NMR (500 MHz, $CDCl_3$) of compound **12**.

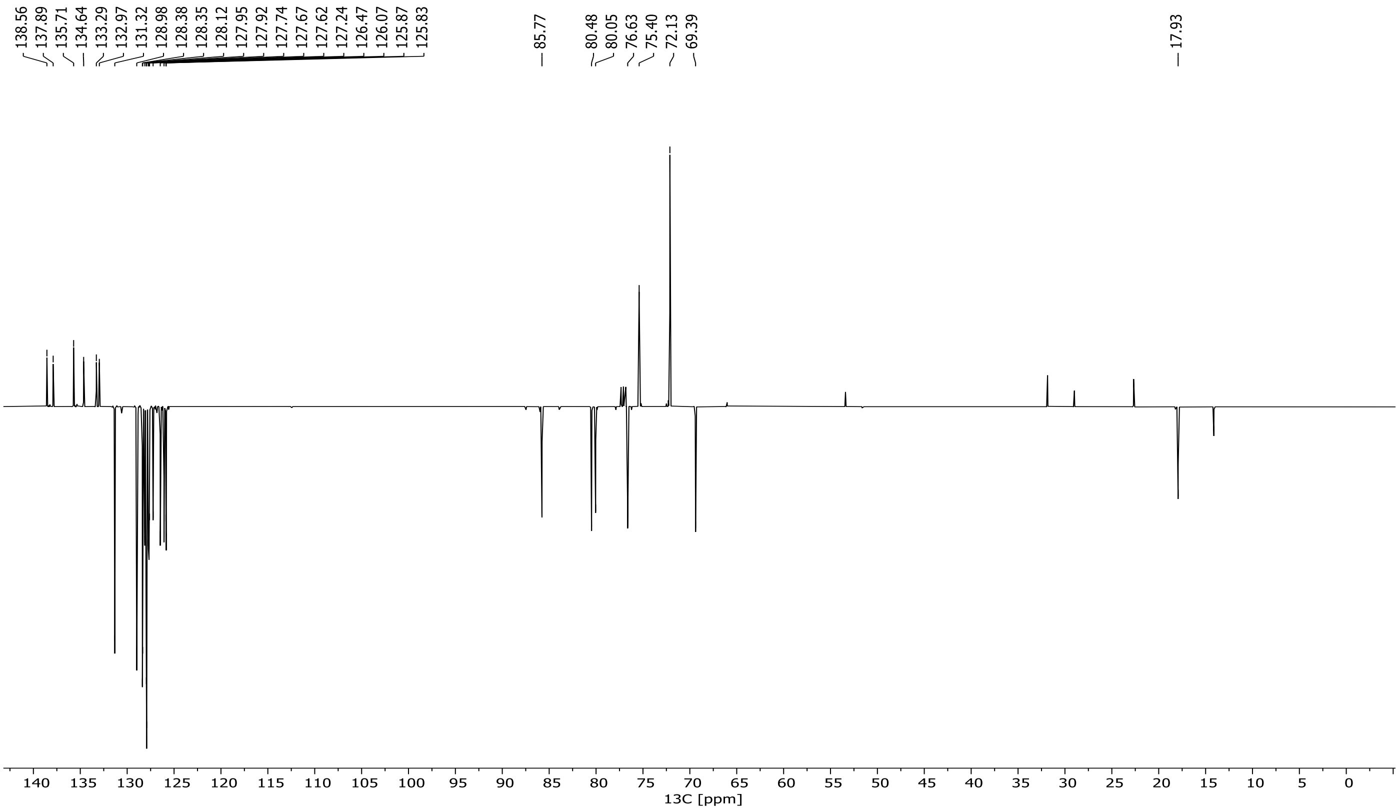

Supplementary Figure 46: ^{13}C NMR (126 MHz, $CDCl_3$) of compound **12**.

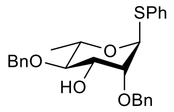

13

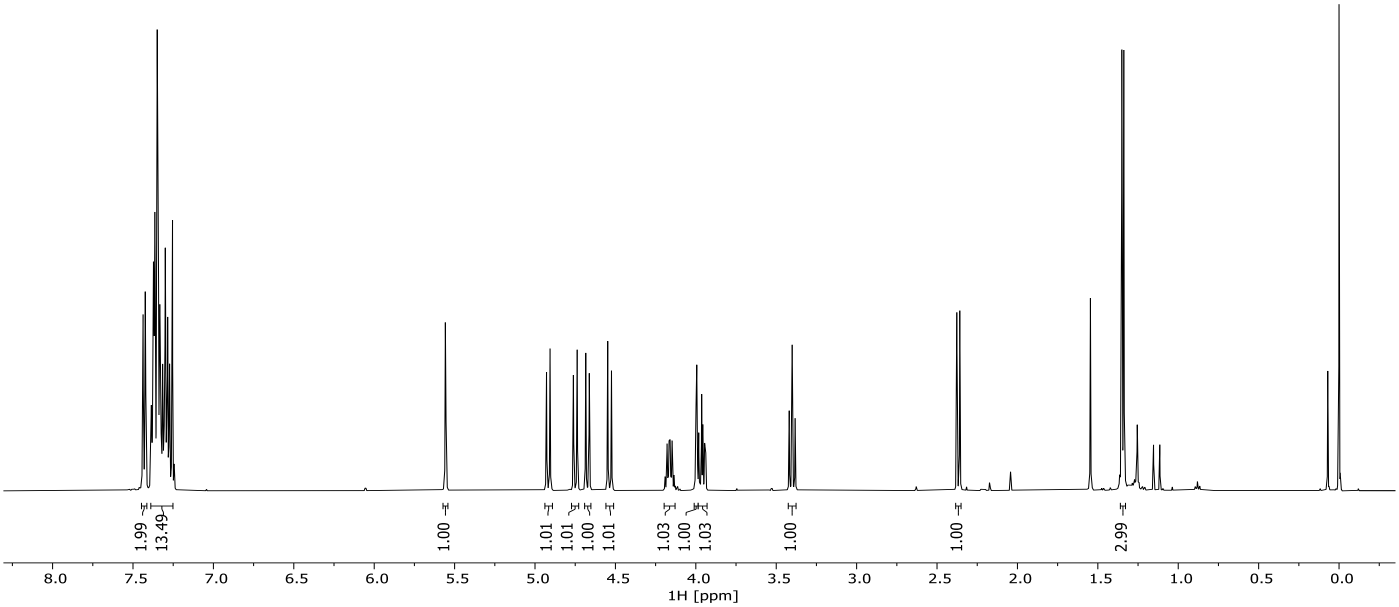

Supplementary Figure 46: ^{1}H NMR (500 MHz, $CDCl_3$) of compound **13**.

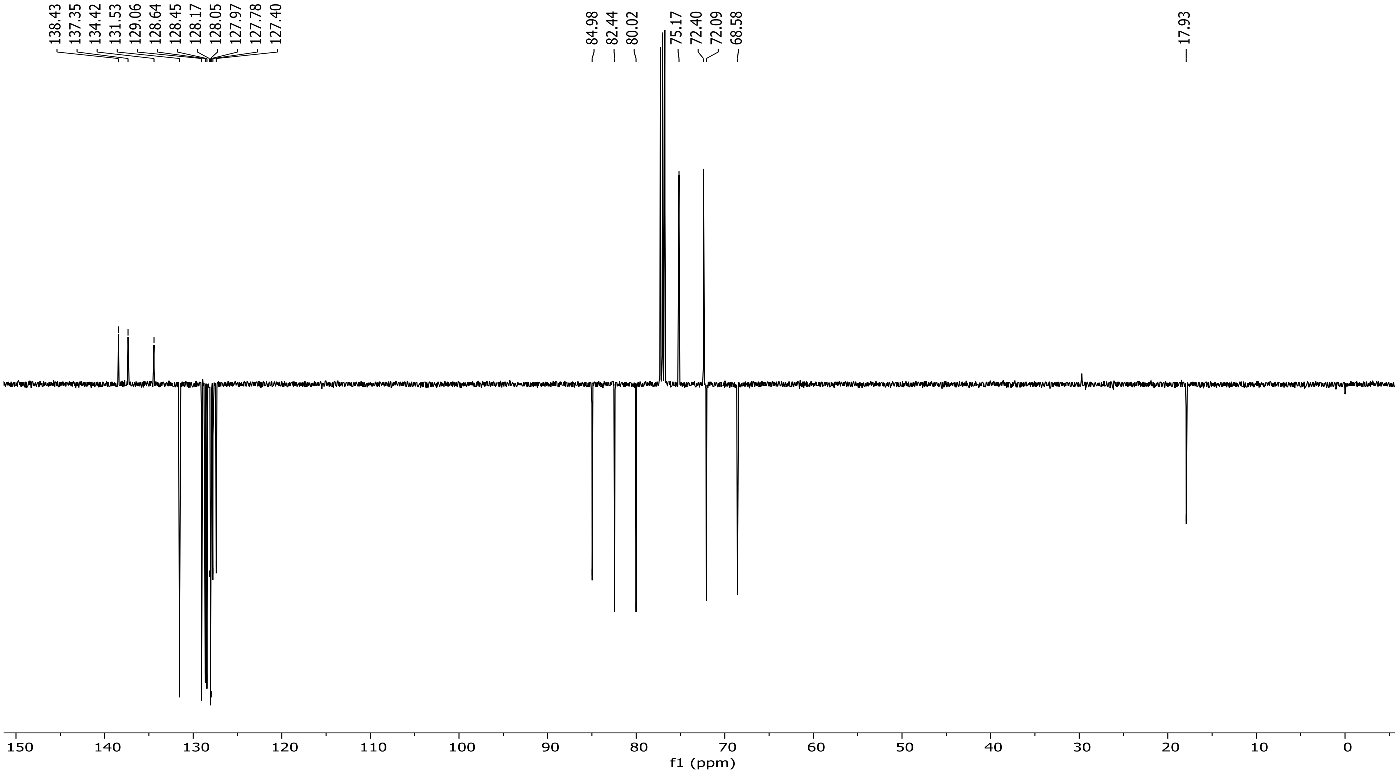

Supplementary Figure 47: ^{13}C NMR (126 MHz, $CDCl_3$) of compound **13**.

Phenyl 3-*O*-*p*-anisoyl-2,4-di-*O*-benzyl-1-thio-α-L-rhamnopyranoside (1)

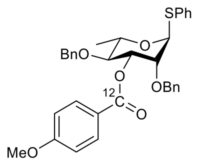

1

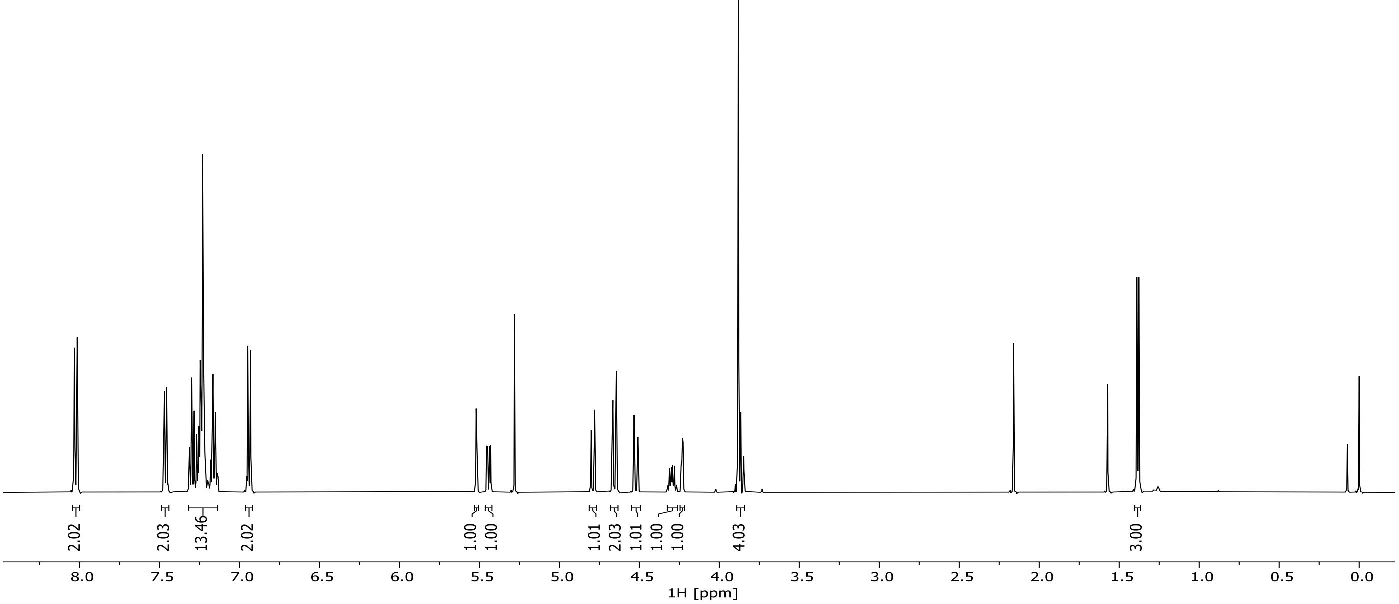

Supplementary Figure 48: ^{1}H NMR (500 MHz, $CDCl_3$) of compound **1**.

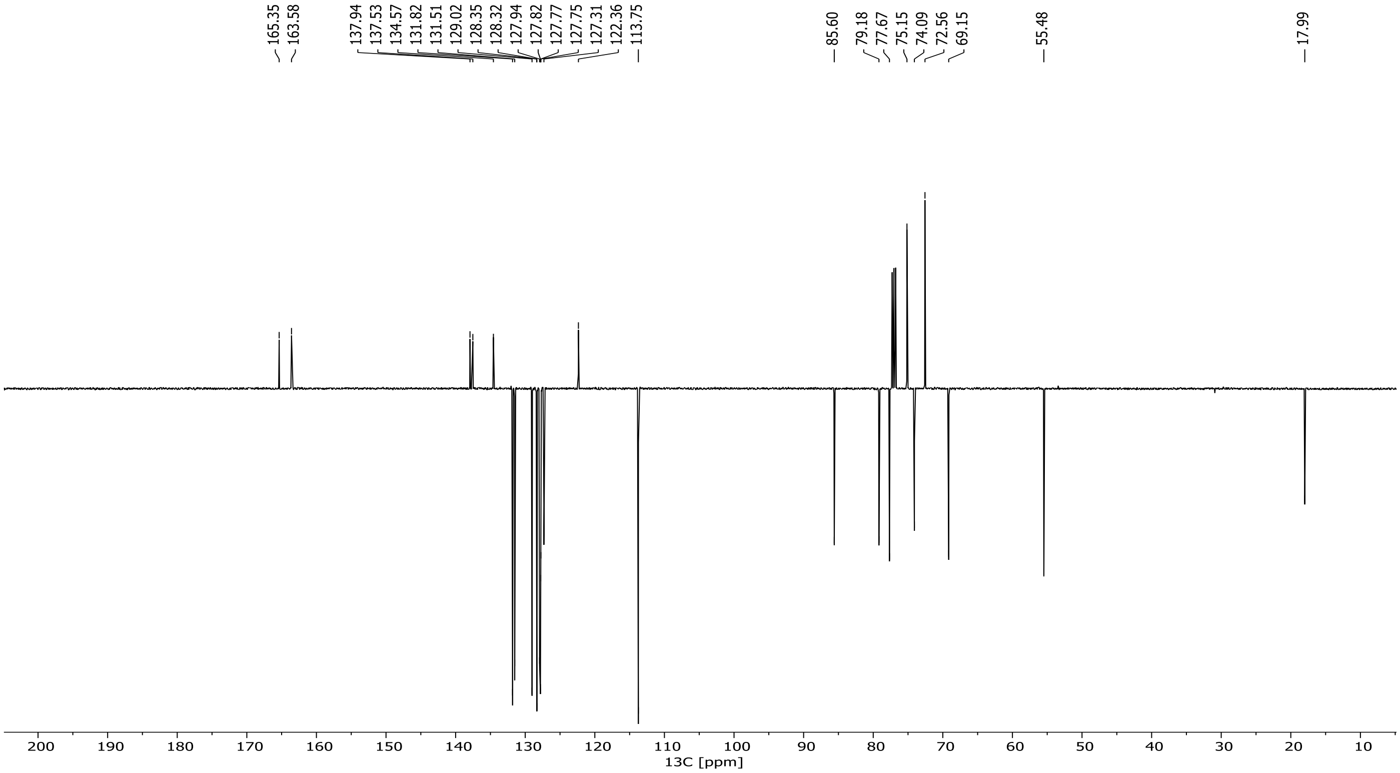

Supplementary Figure 49: ^{13}C NMR (126 MHz, $CDCl_3$) of compound **1**.

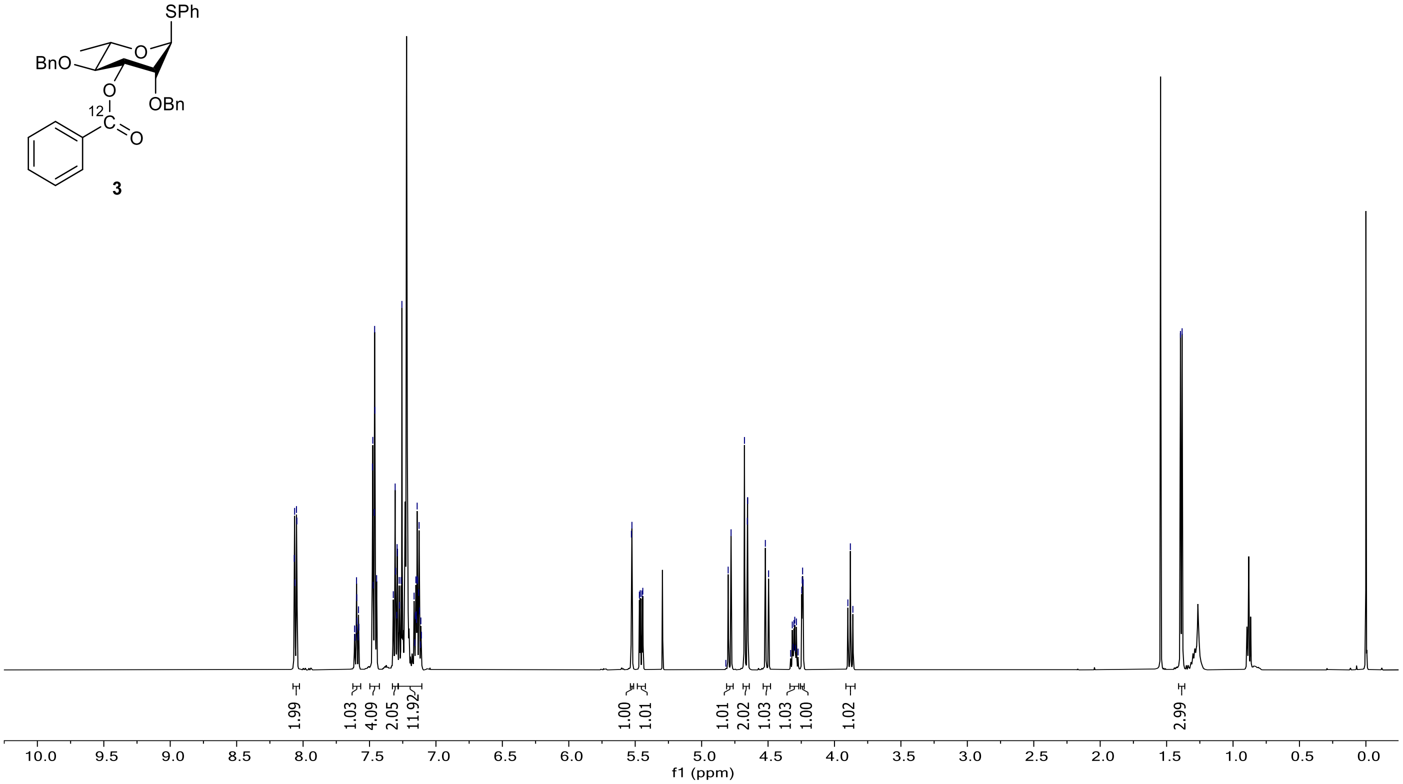

Supplementary Figure 50: ^{1}H NMR (500 MHz, $CDCl_3$) of compound **3**.

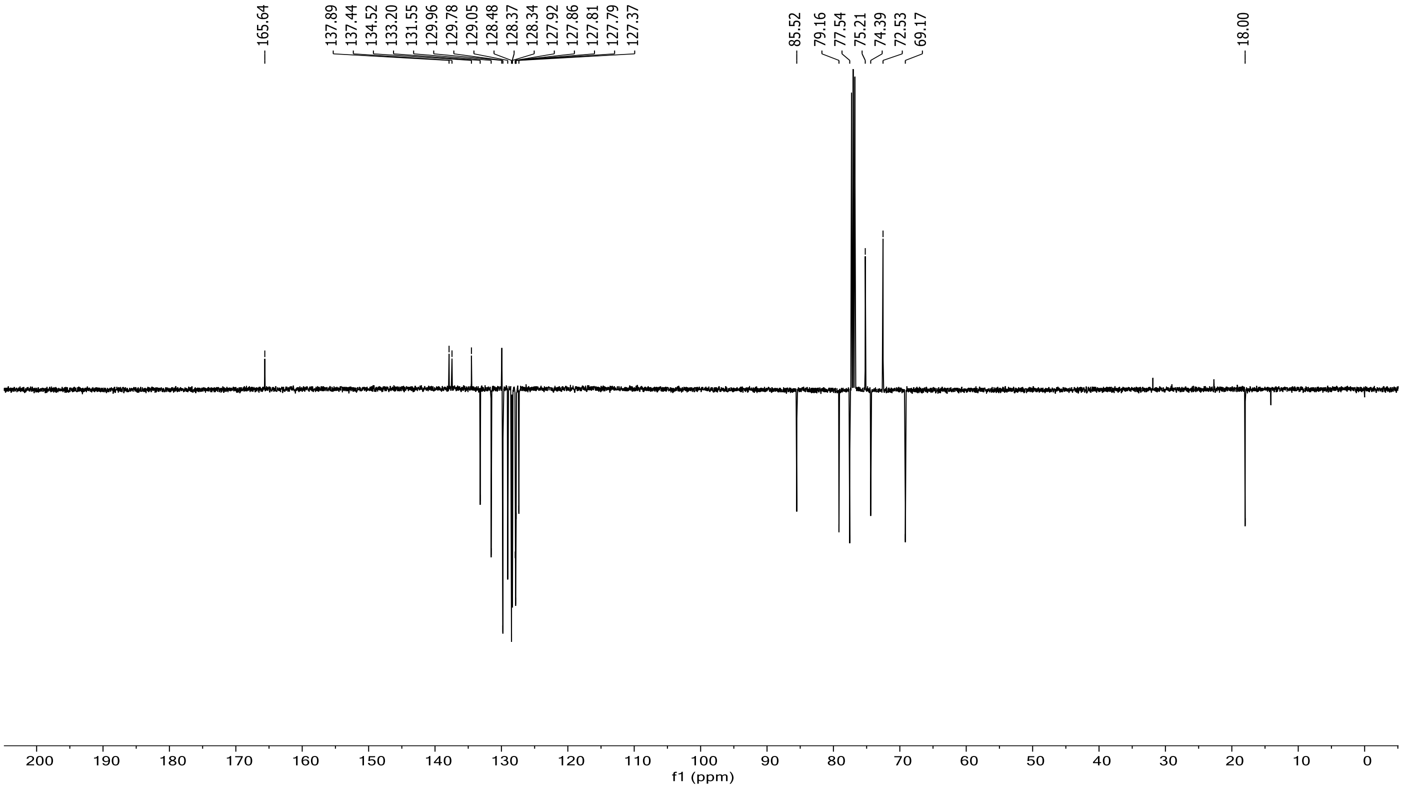

Supplementary Figure 51: ^{13}C NMR (126 MHz, $CDCl_3$) of compound **3**.

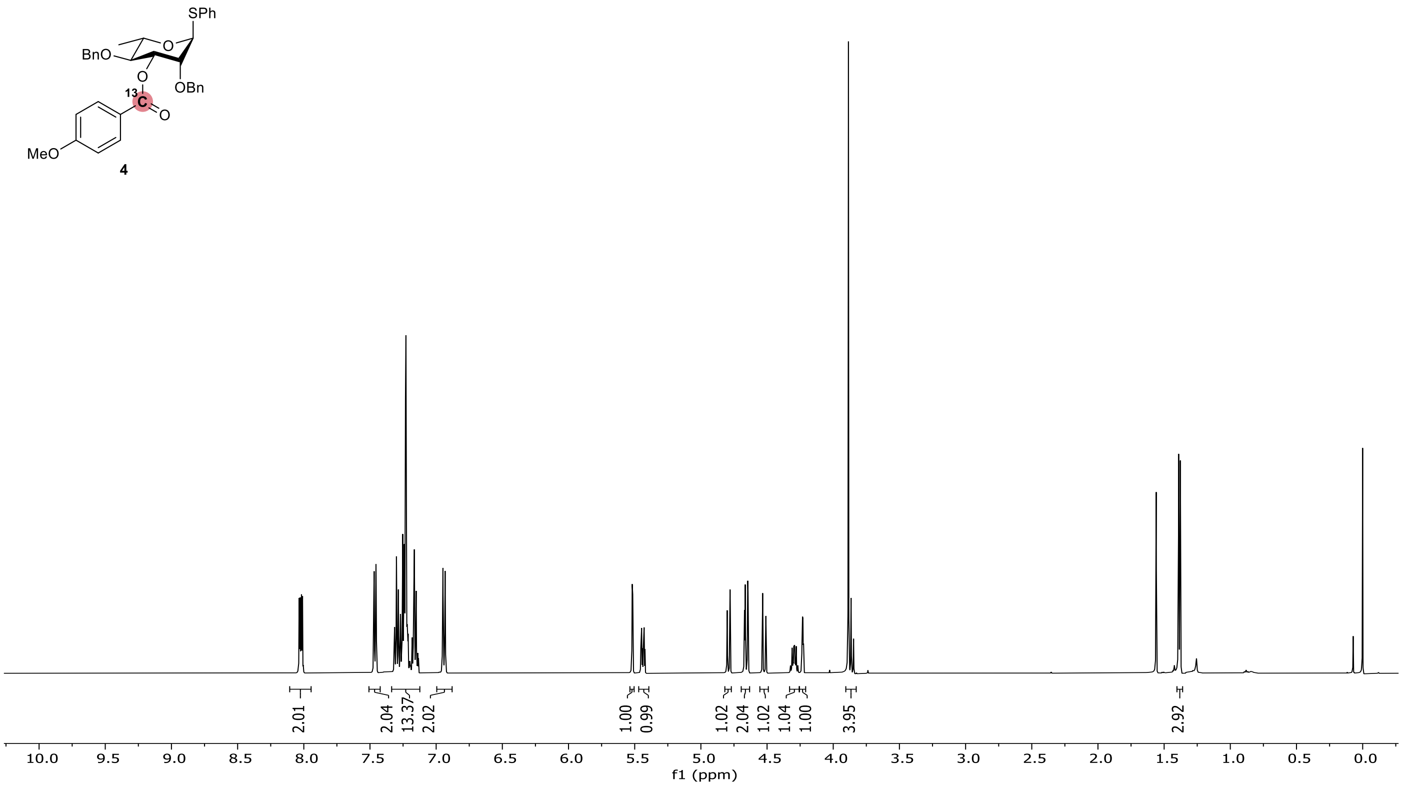

Supplementary Figure 52: ^{1}H NMR (500 MHz, $CDCl_3$) of compound **4**.

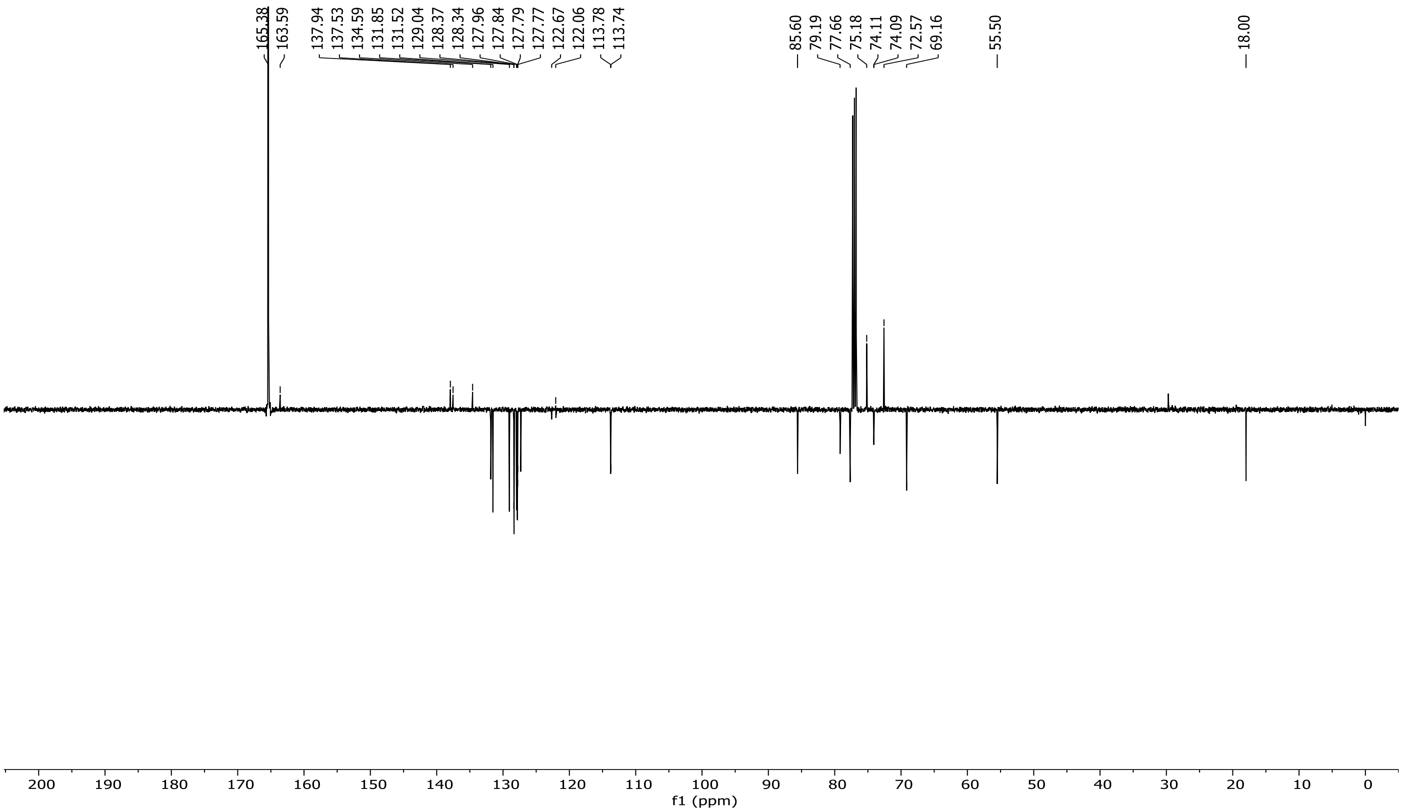

Supplementary Figure 53: ^{13}C NMR (126 MHz, $CDCl_3$) of compound **4**.

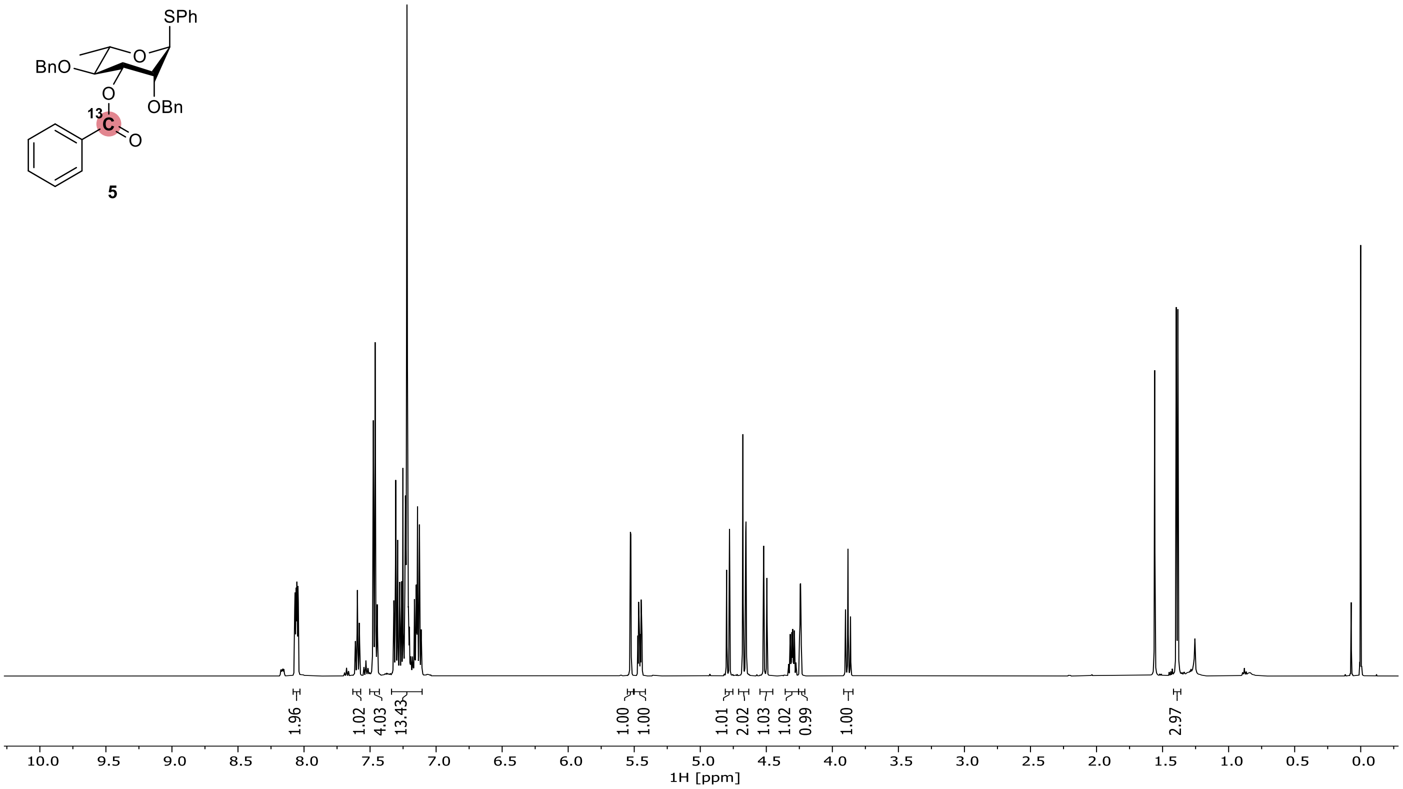

Supplementary Figure 54: ^{1}H NMR (500 MHz, $CDCl_3$) of compound **5**.

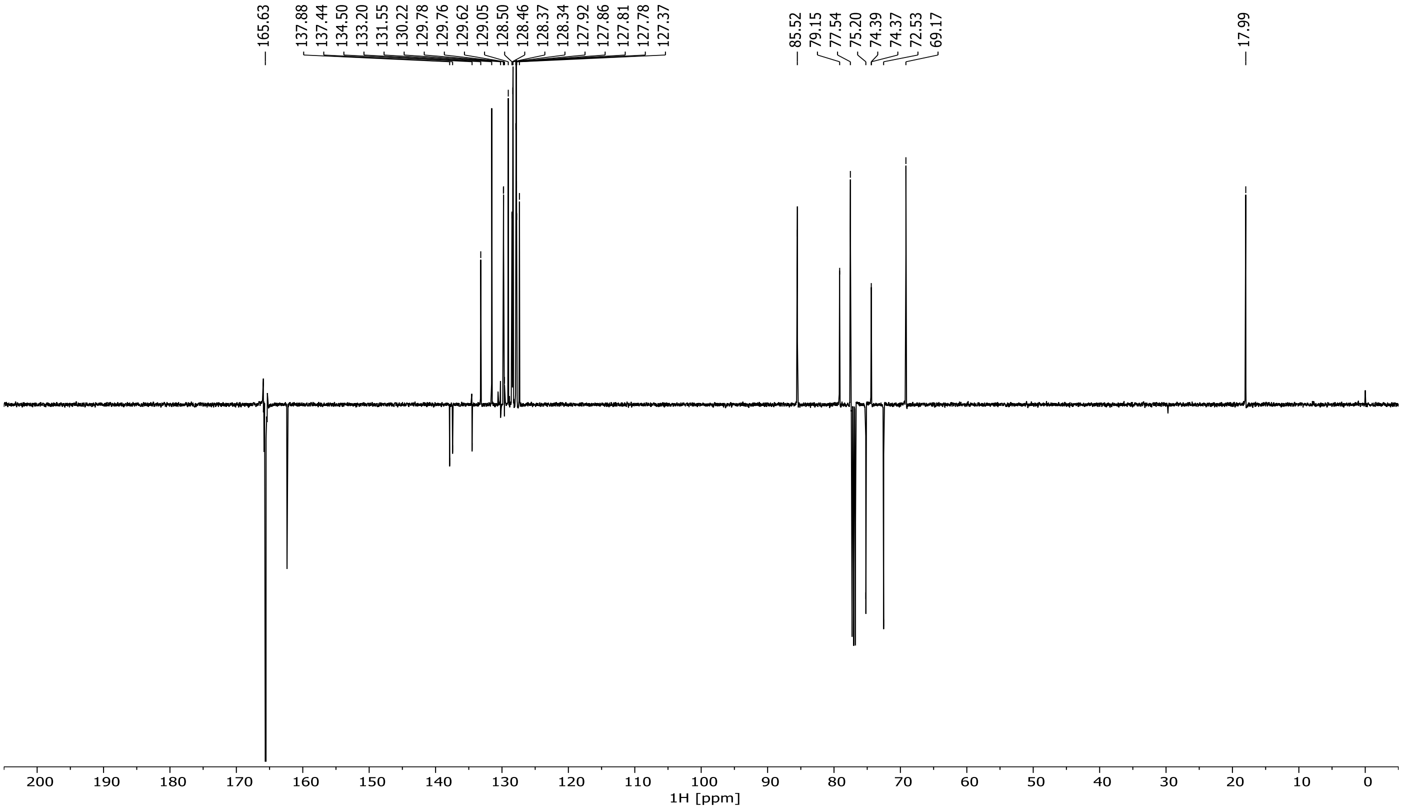

Supplementary Figure 55: ^{13}C NMR (126 MHz, $CDCl_3$) of compound **5**.

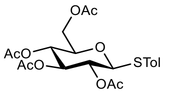

15

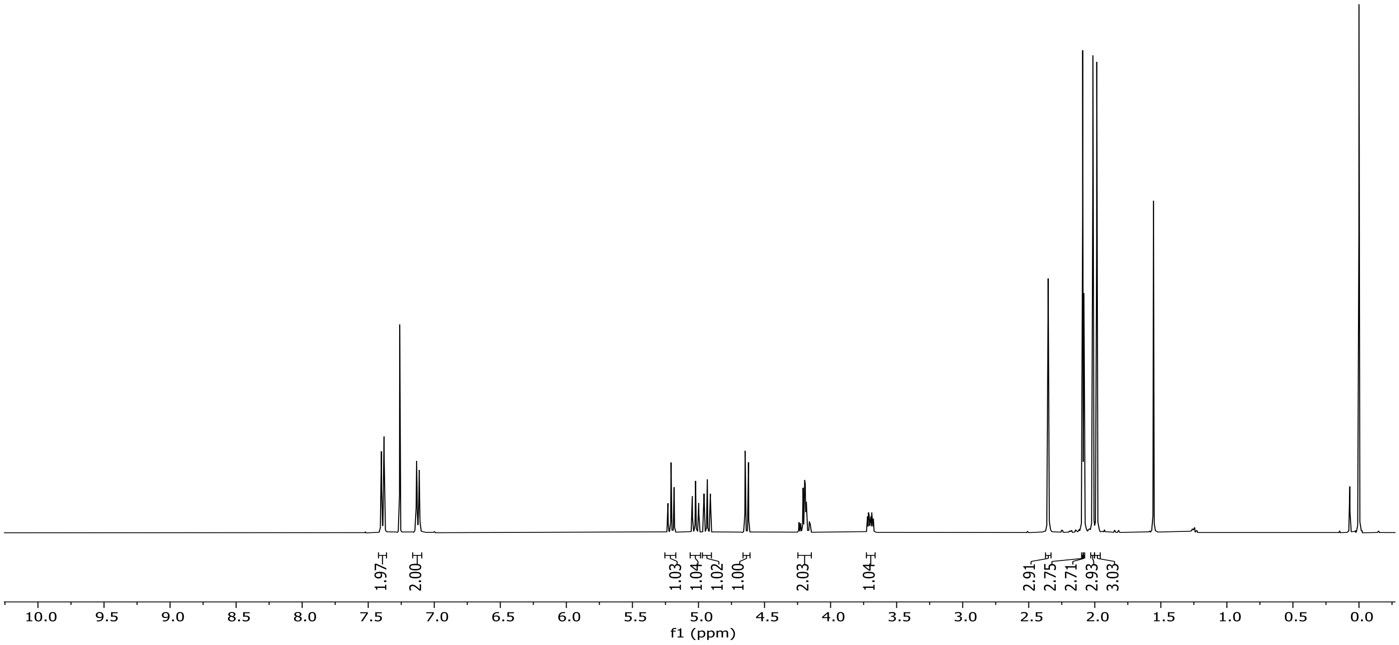

Supplementary Figure 56: ^{1}H NMR (500 MHz, $CDCl_3$) of compound **15**.

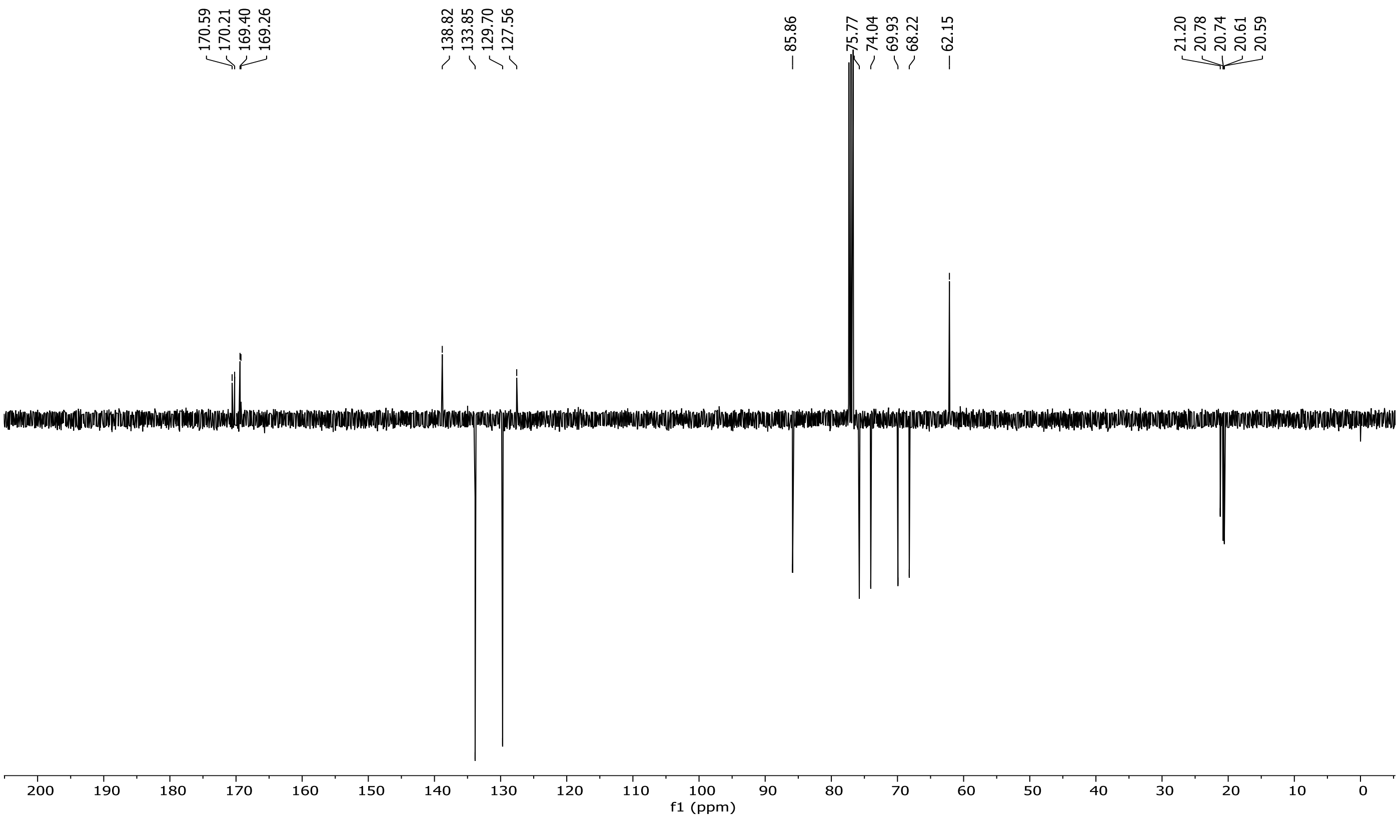

Supplementary Figure 57: ^{13}C NMR (126 MHz, $CDCl_3$) of compound **15**.

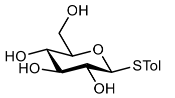

16

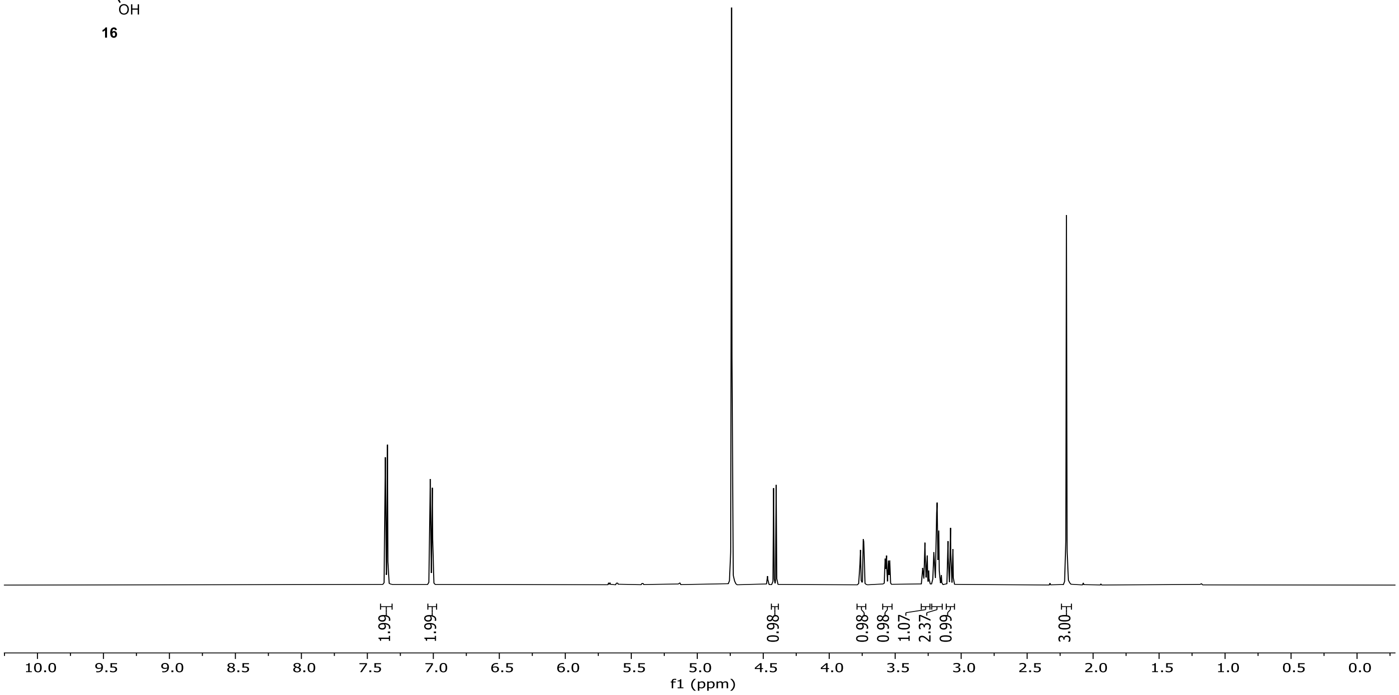

Supplementary Figure 58: ^{1}H NMR (500 MHz, MeOD) of compound **16**.

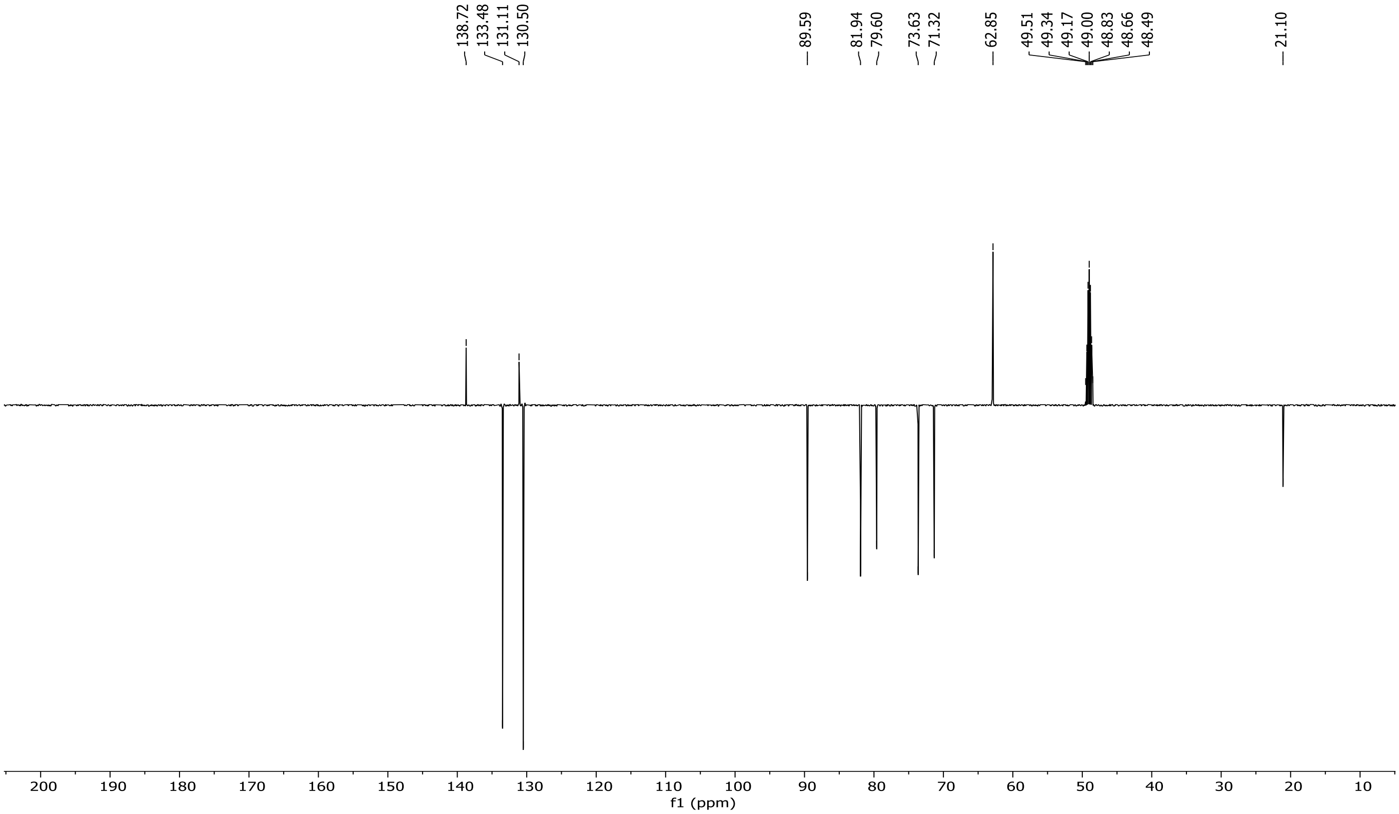

Supplementary Figure 59: ^{13}C NMR (126 MHz, MeOD) of compound **16**.

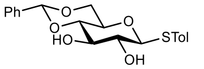

17

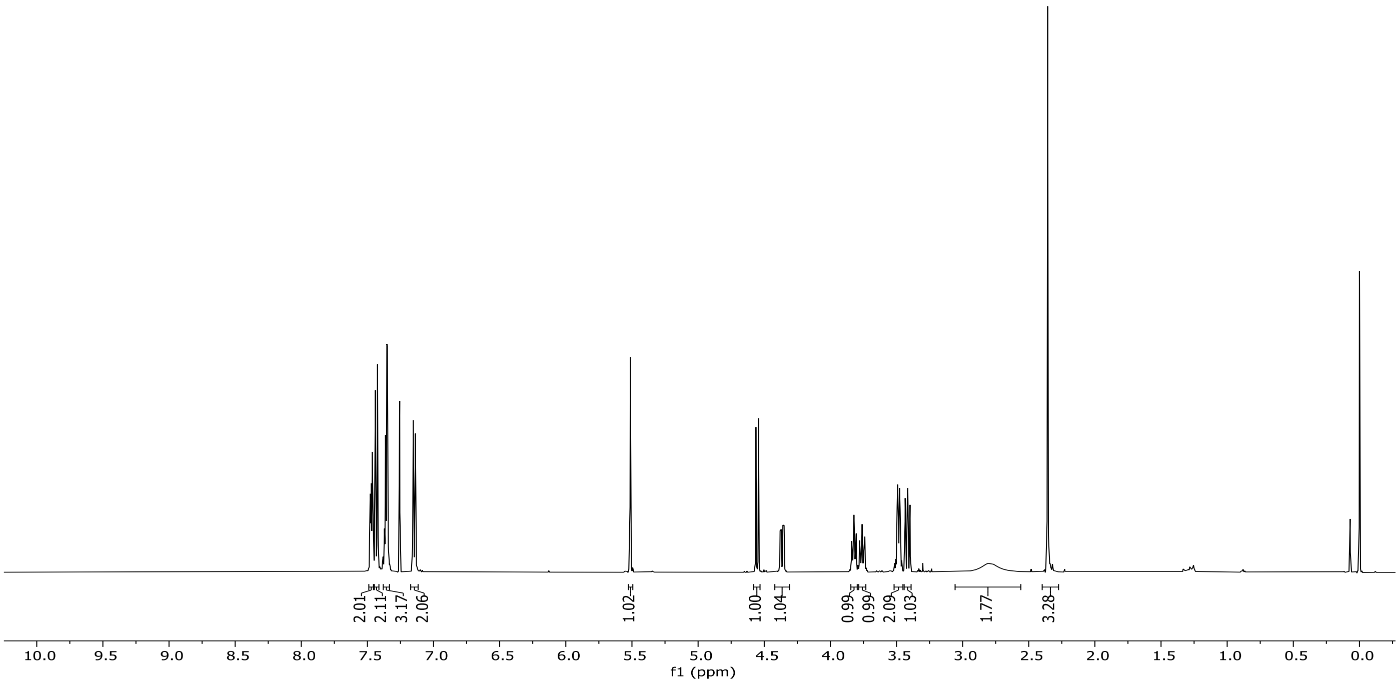

Supplementary Figure 60: ^{1}H NMR (500 MHz, $CDCl_3$) of compound **17**.

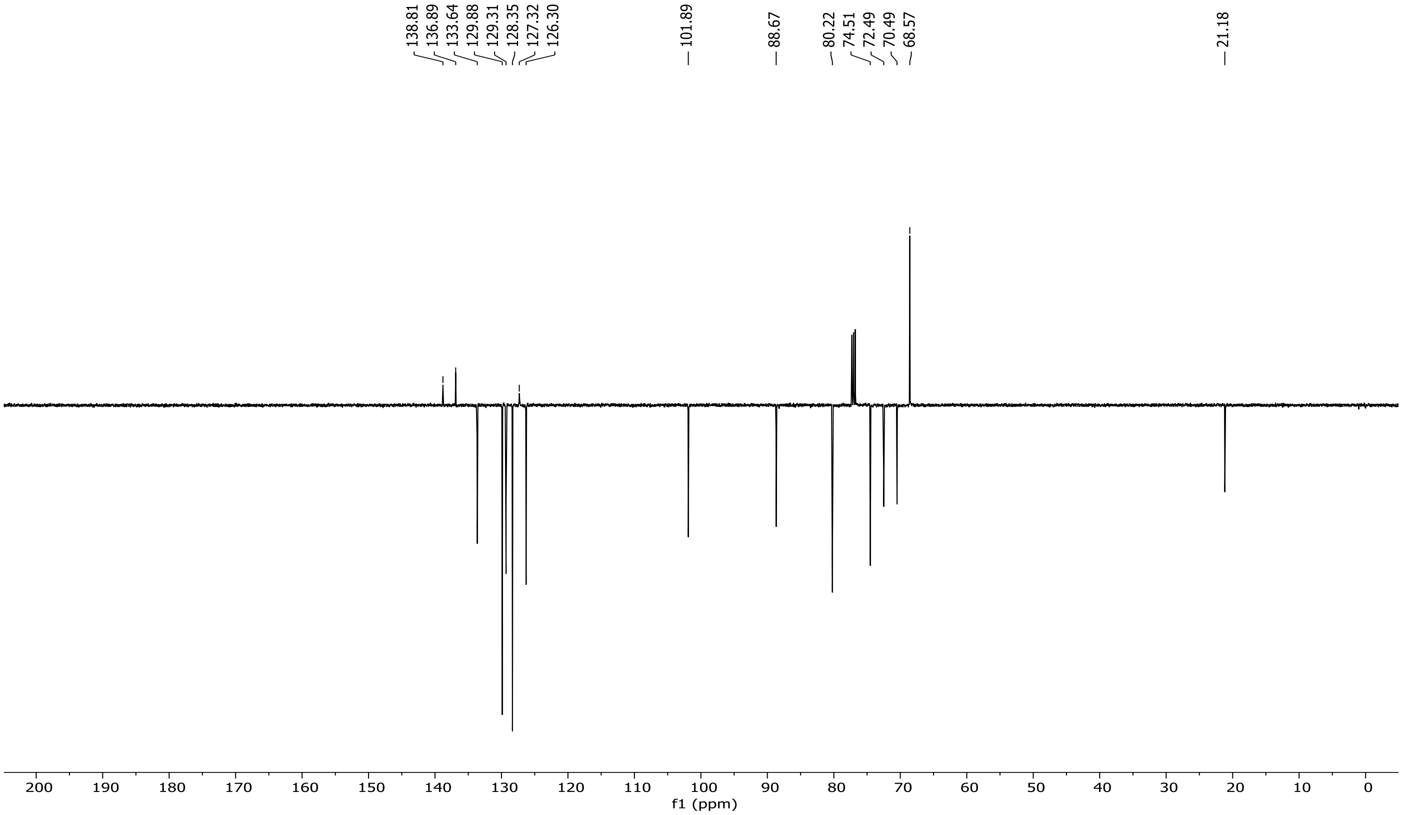

Supplementary Figure 61: ^{13}C NMR (126 MHz, $CDCl_3$) of compound **17**.

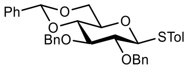

18

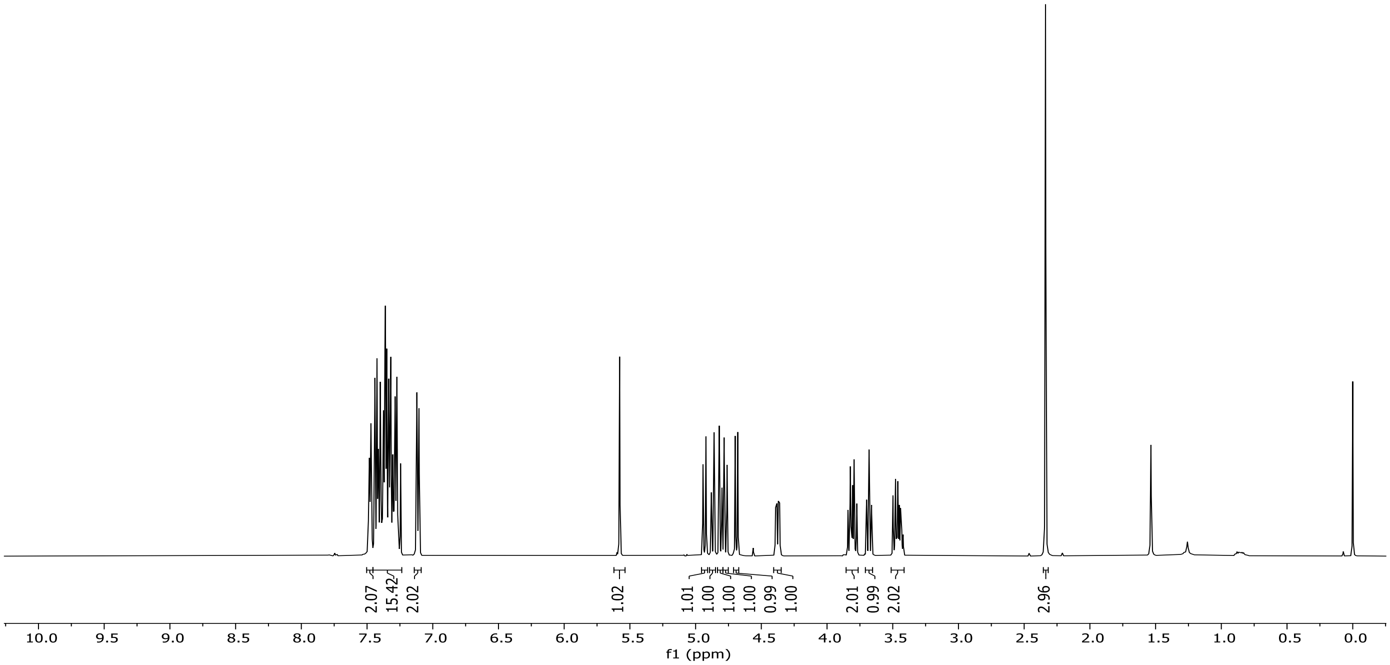

Supplementary Figure 62: ^{1}H NMR (500 MHz, $CDCl_3$) of compound **18**.

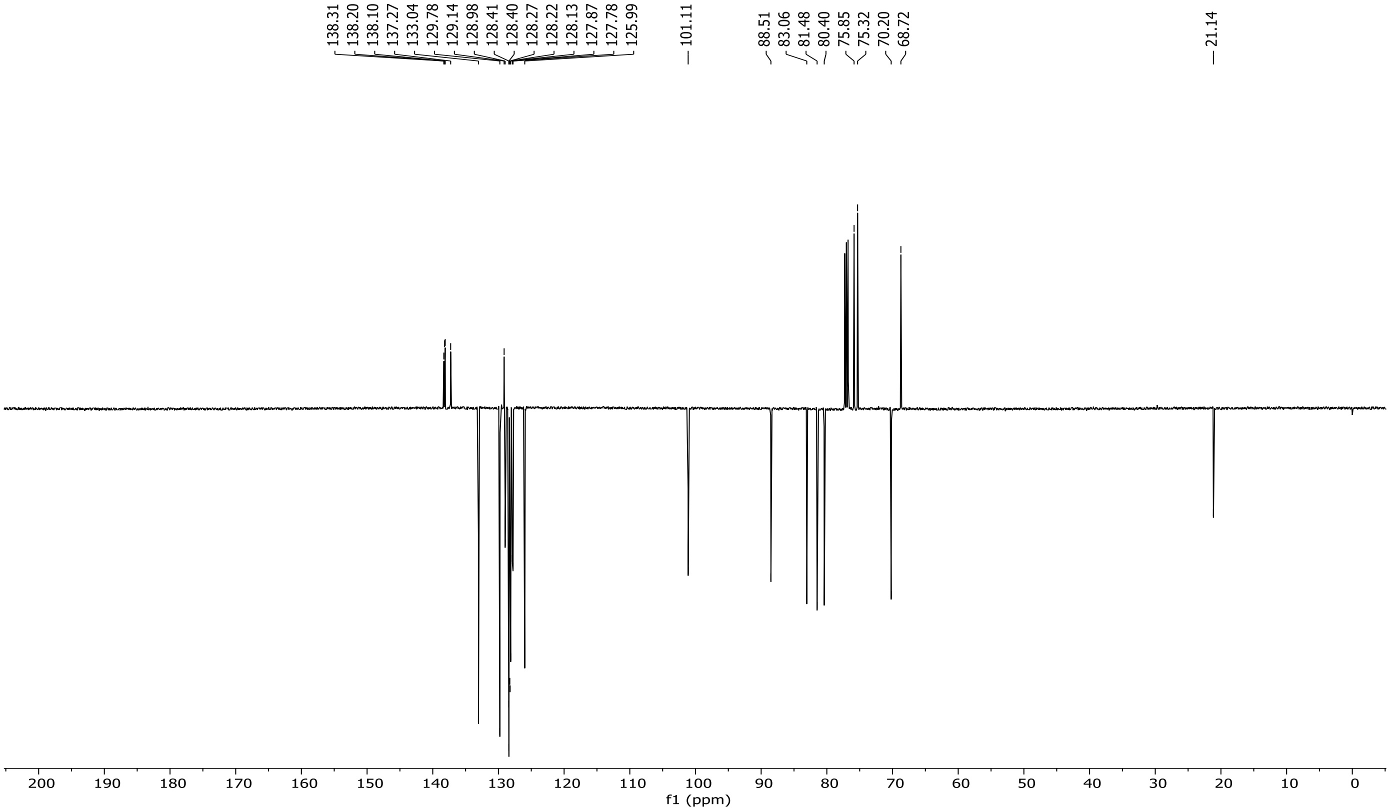

Supplementary Figure 63: ^{13}C NMR (126 MHz, $CDCl_3$) of compound **18**.

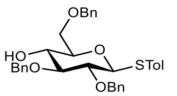

19

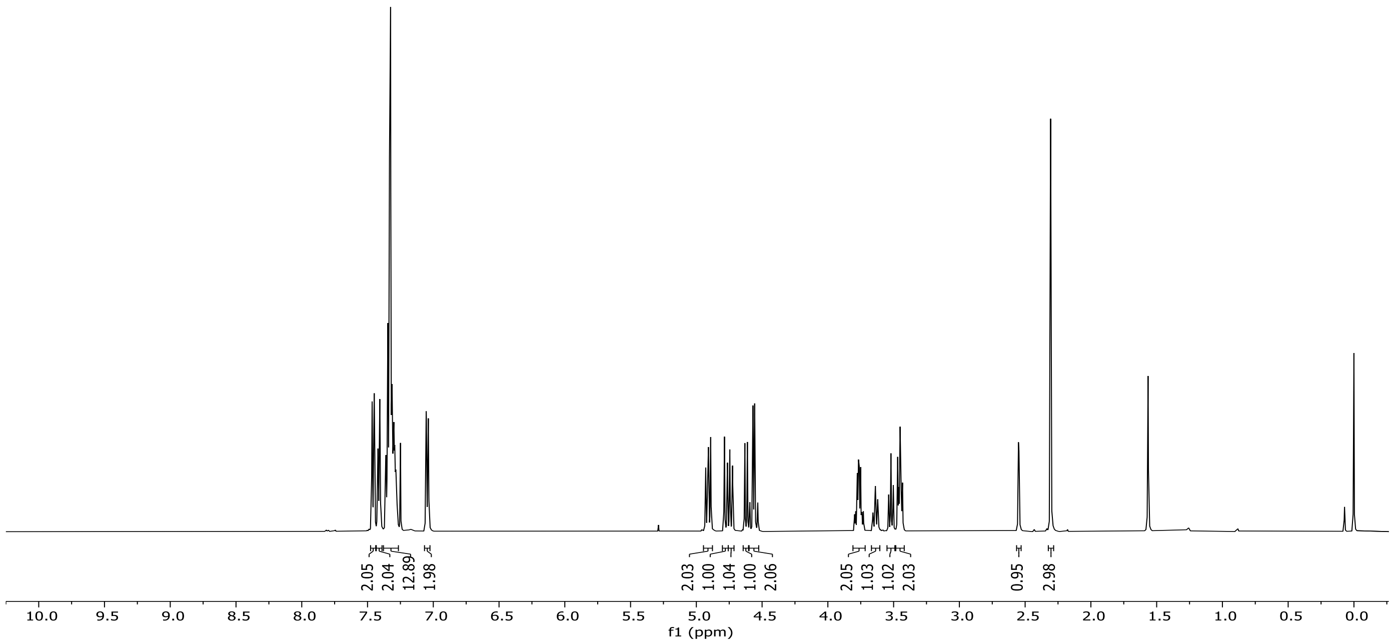

Supplementary Figure 64: ^{1}H NMR (500 MHz, $CDCl_3$) of compound **19**.

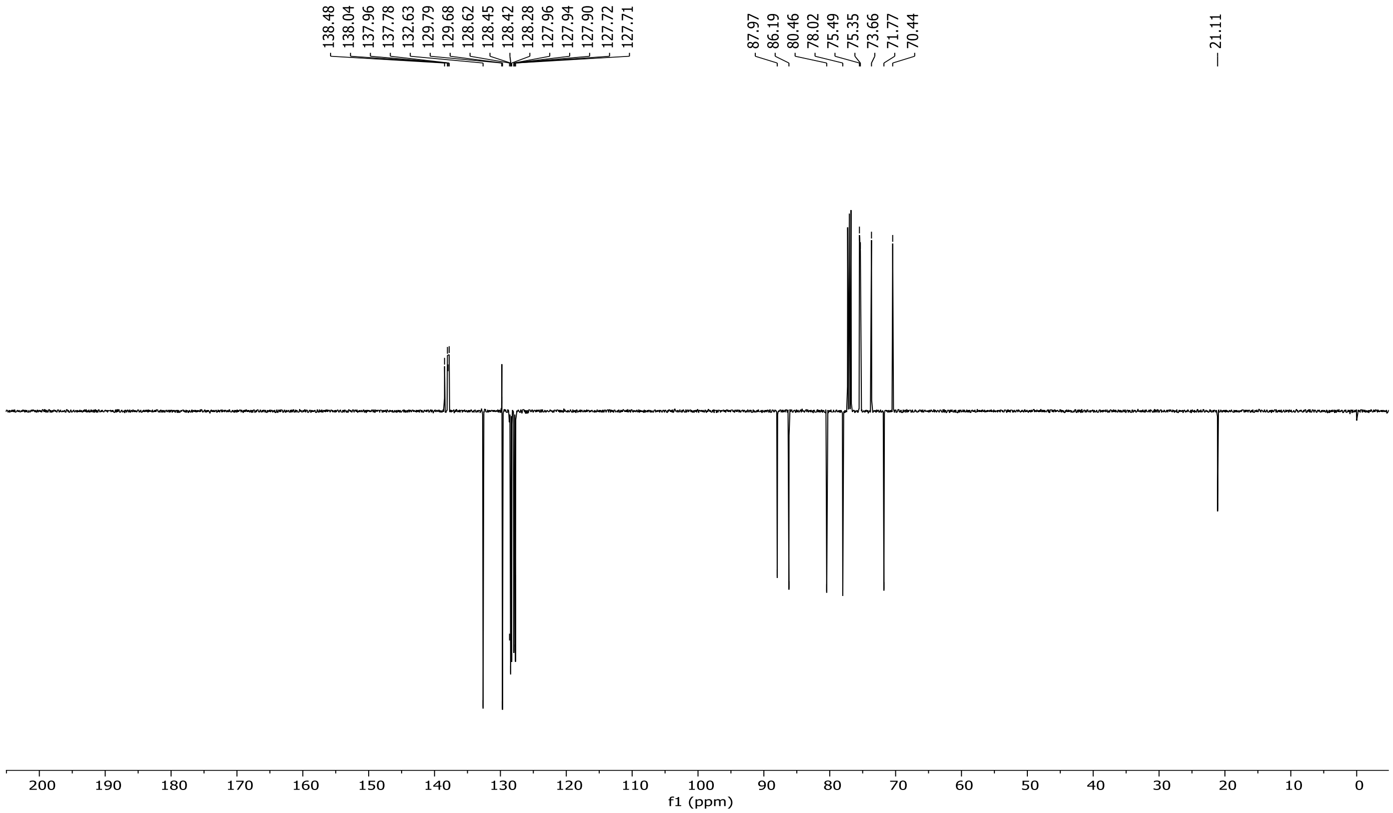

Supplementary Figure 65: ^{13}C NMR (126 MHz, $CDCl_3$) of compound **19**.

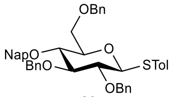

20

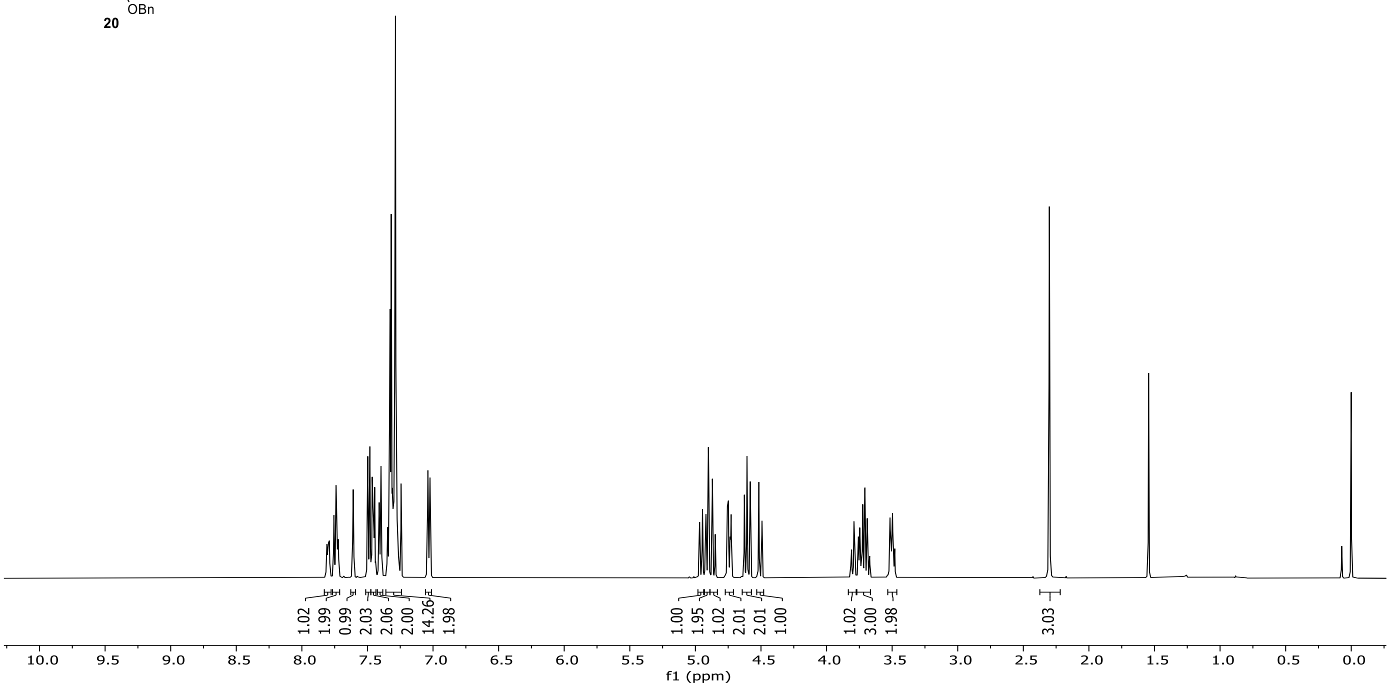

Supplementary Figure 66: ^{1}H NMR (500 MHz, $CDCl_3$) of compound **20**.

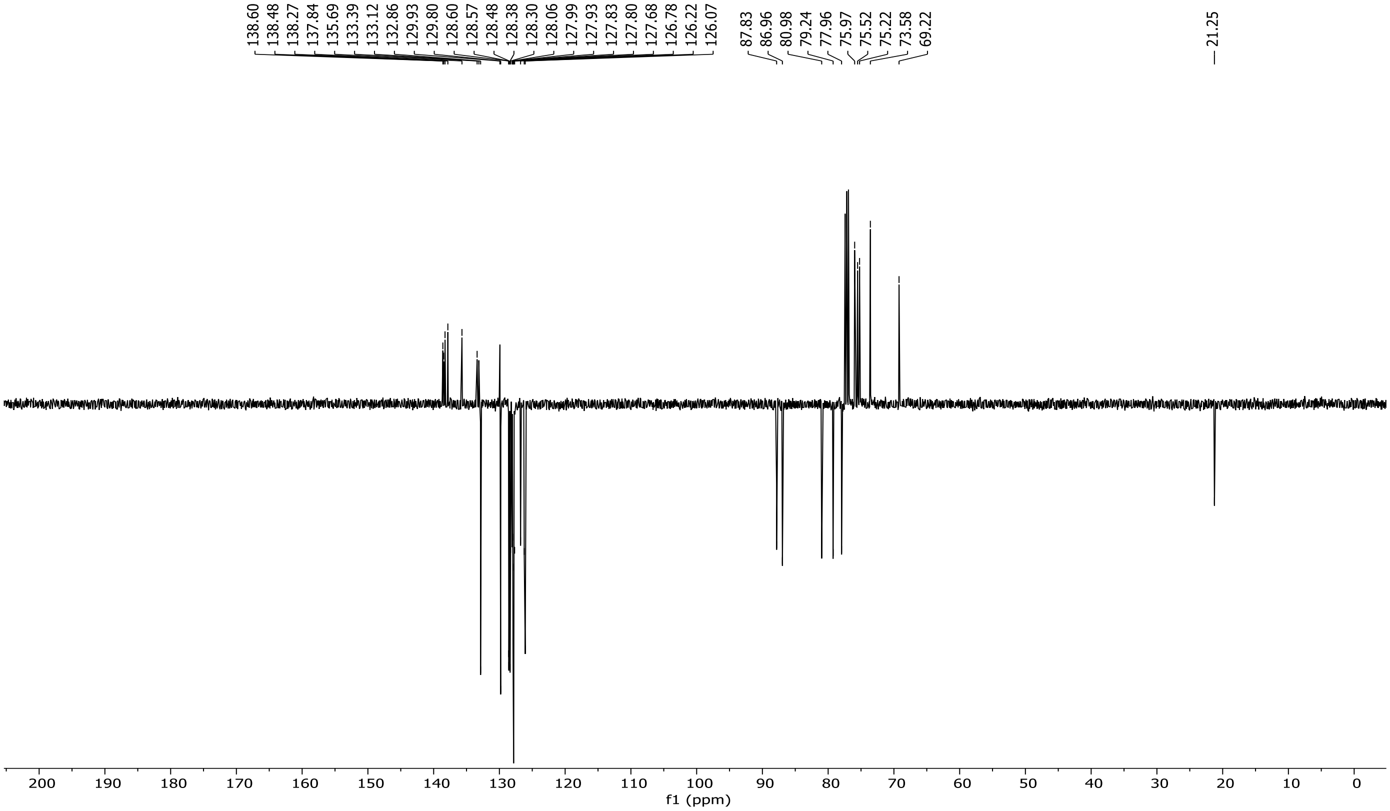

Supplementary Figure 67: ^{13}C NMR (126 MHz, $CDCl_3$) of compound **20**.

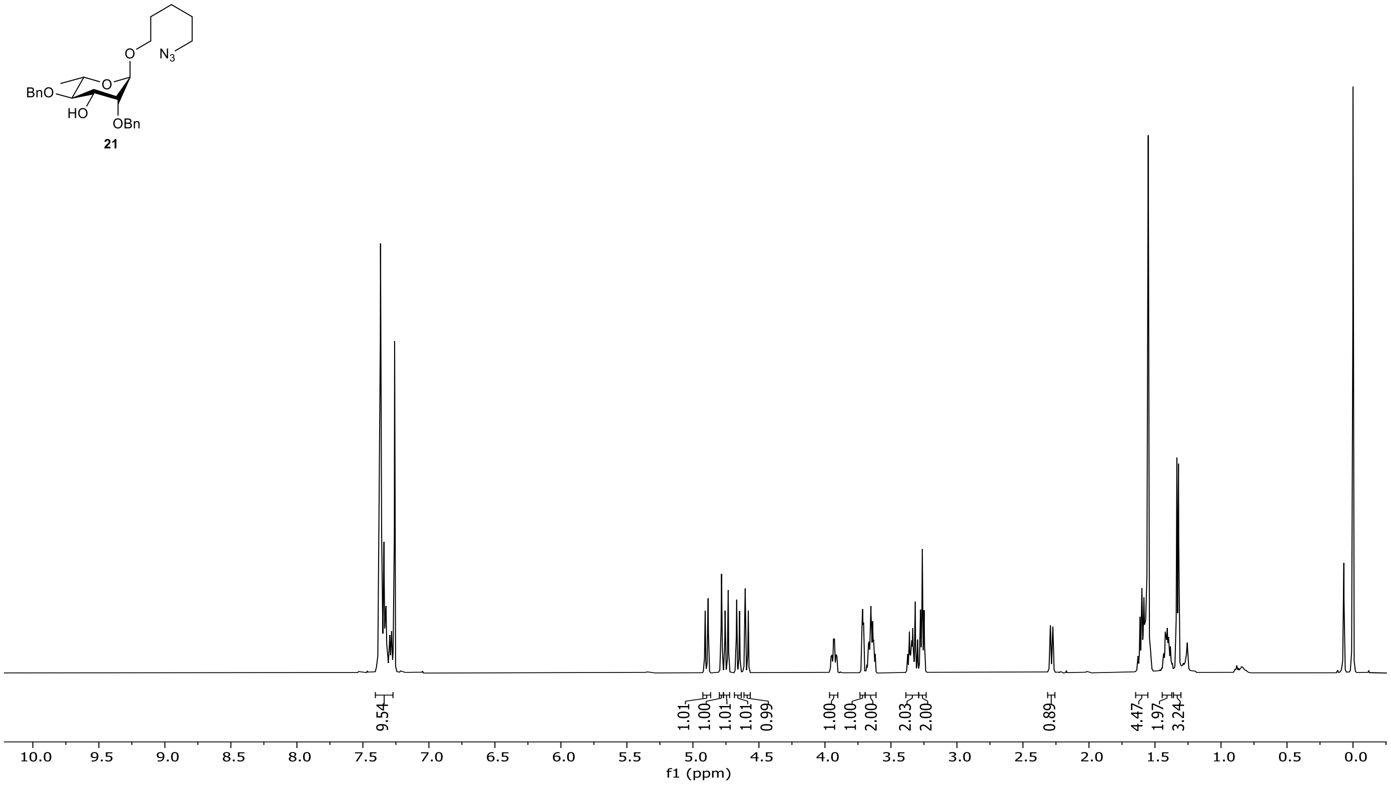

Supplementary Figure 68: ^{1}H NMR (500 MHz, $CDCl_3$) of compound **21**.

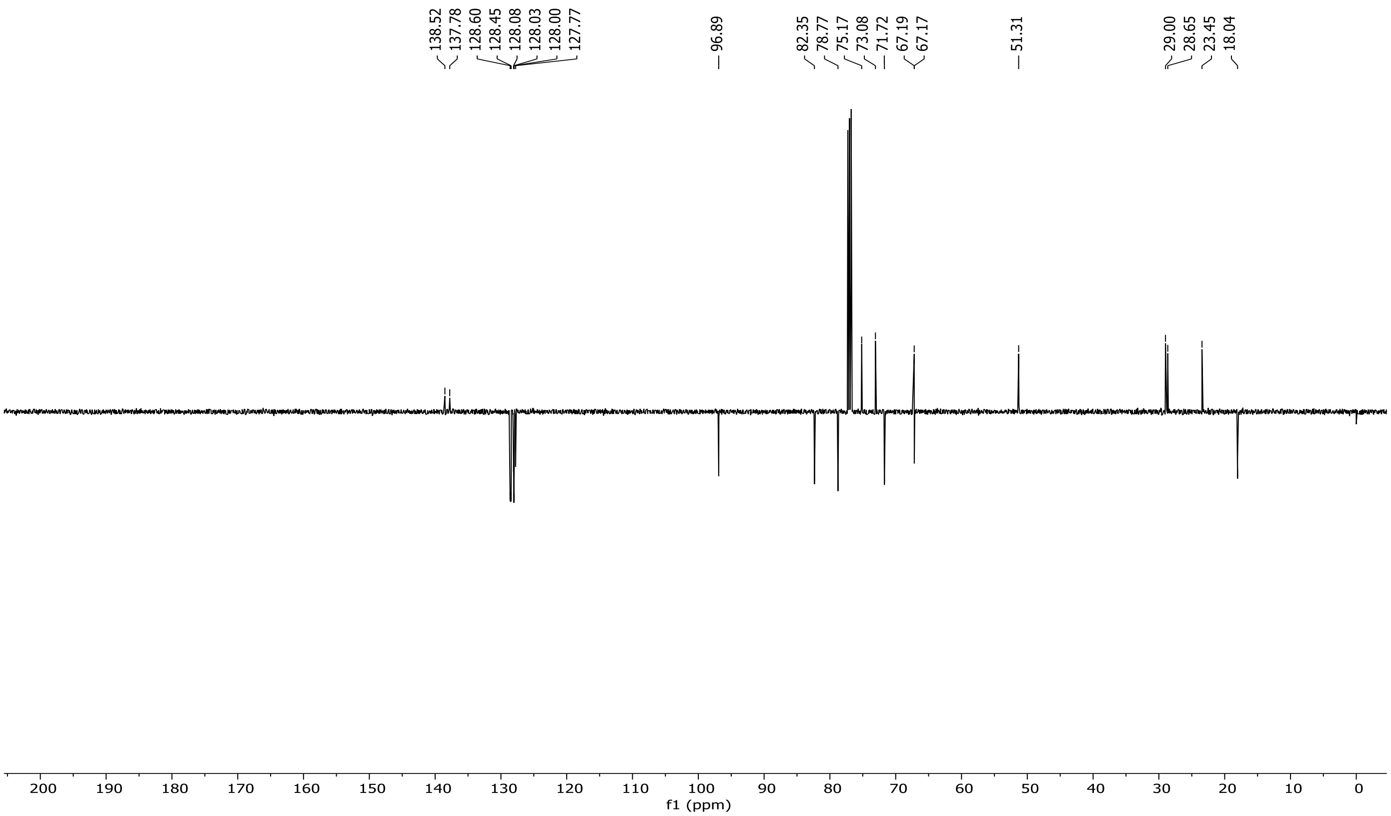

Supplementary Figure 69: ^{13}C NMR (126 MHz, $CDCl_3$) of compound **21**.

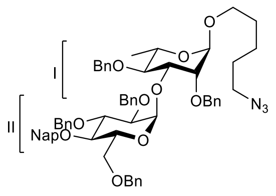

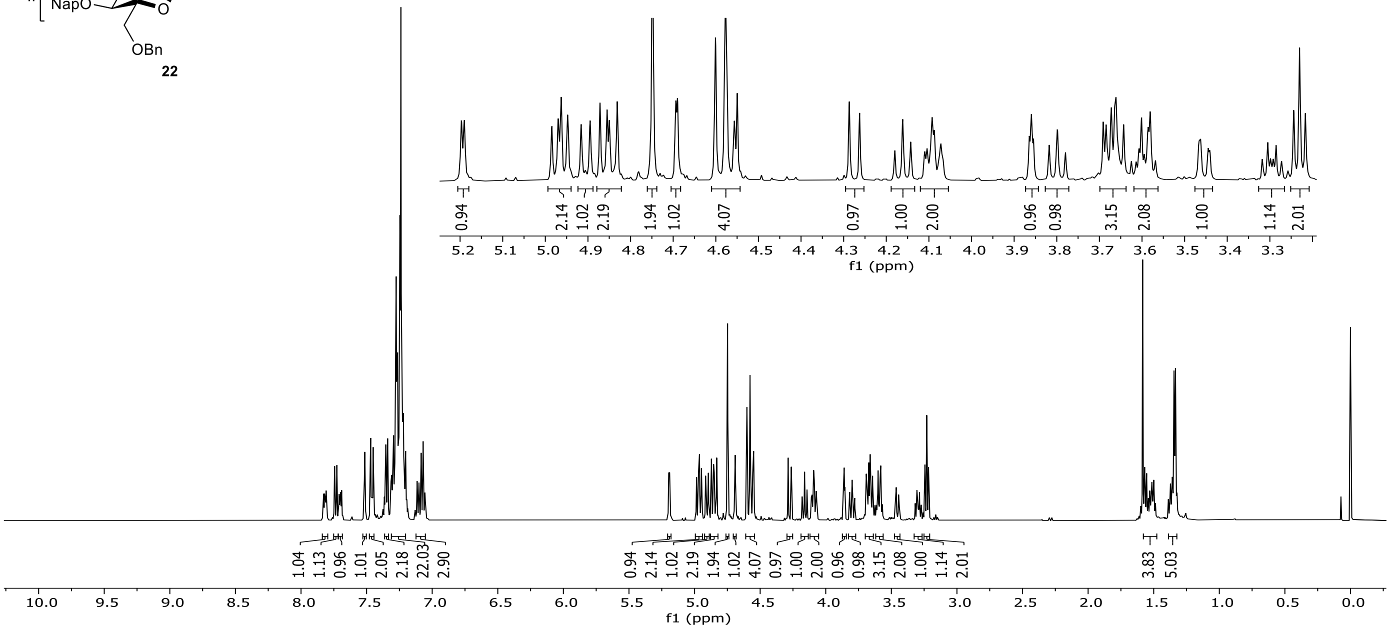

Supplementary Figure 70: ^{1}H NMR (500 MHz, $CDCl_3$) of compound **22**.

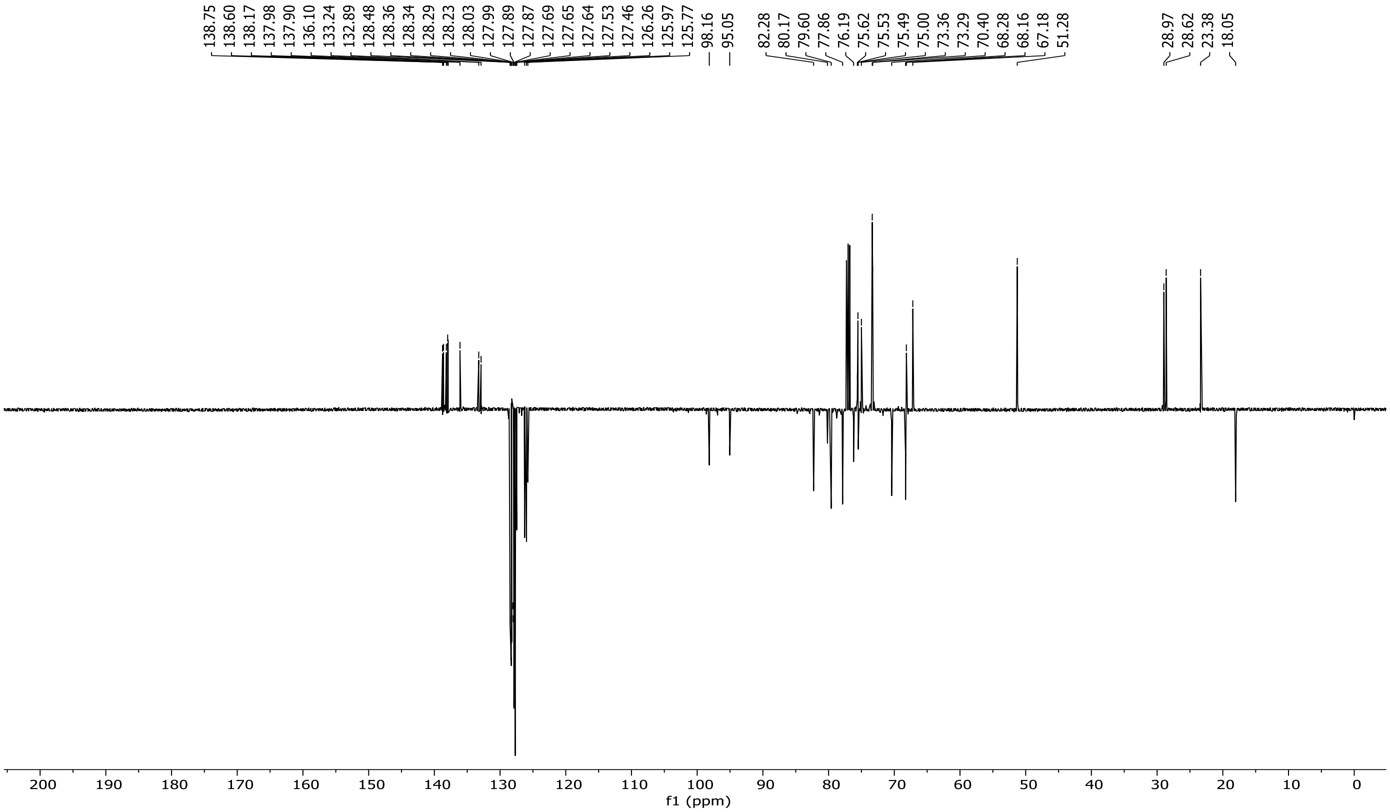

Supplementary Figure 71: ^{13}C NMR (126 MHz, $CDCl_3$) of compound **22**.

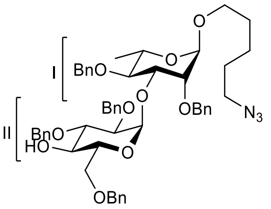

23

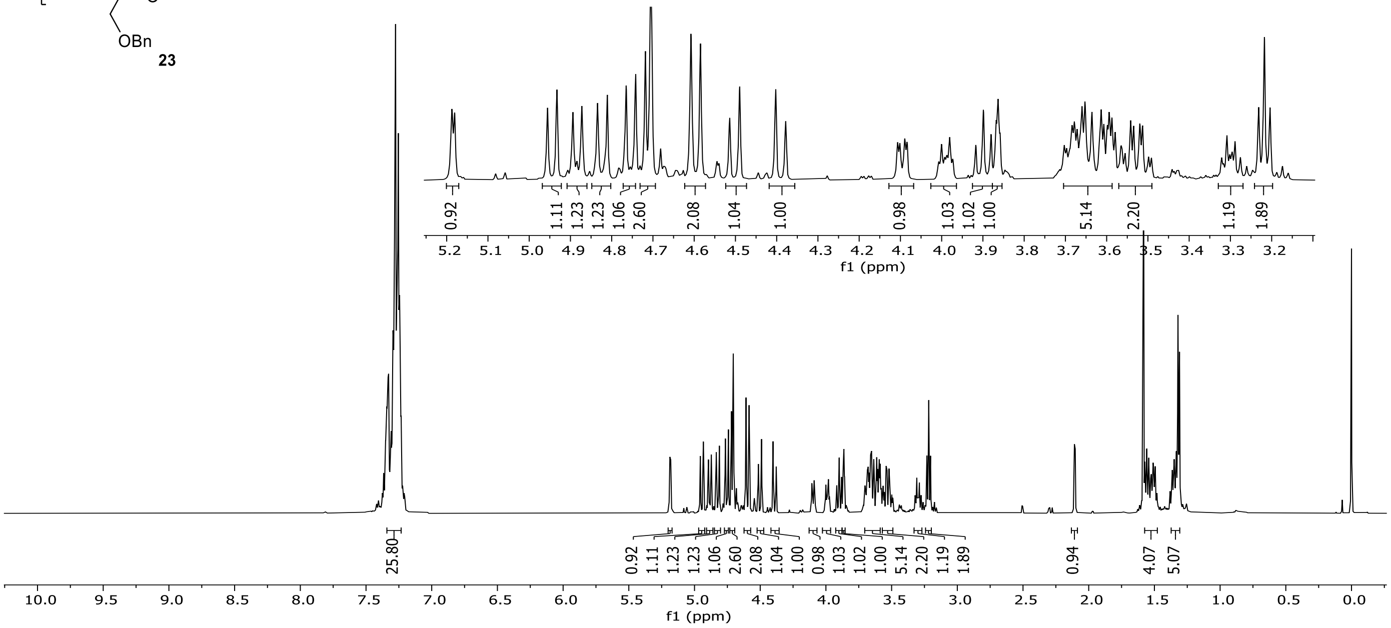

Supplementary Figure 72: ^{1}H NMR (500 MHz, $CDCl_3$) of compound **23**.

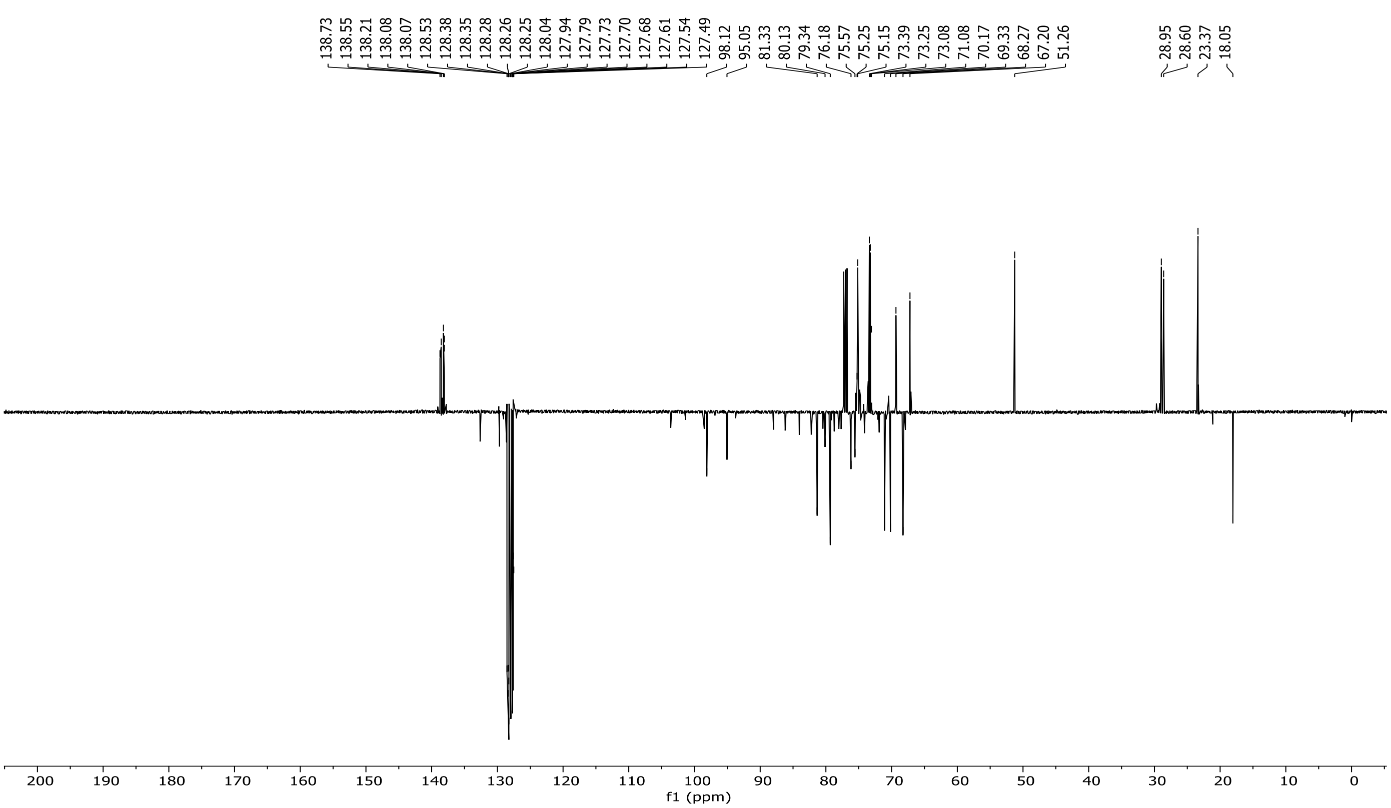

Supplementary Figure 73: ^{13}C NMR (126 MHz, $CDCl_3$) of compound **23**.

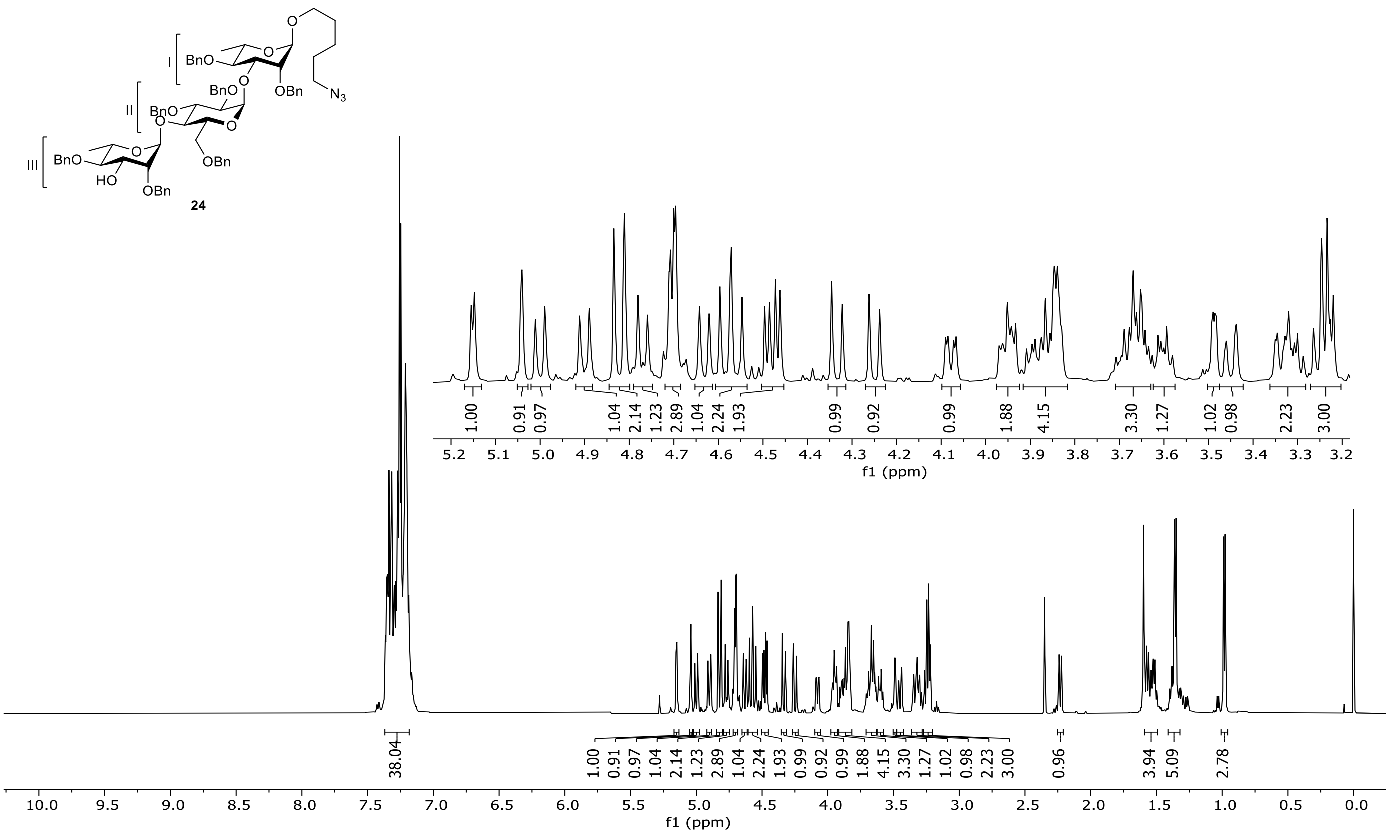

Supplementary Figure 74: ^{1}H NMR (500 MHz, $CDCl_3$) of compound **24**.

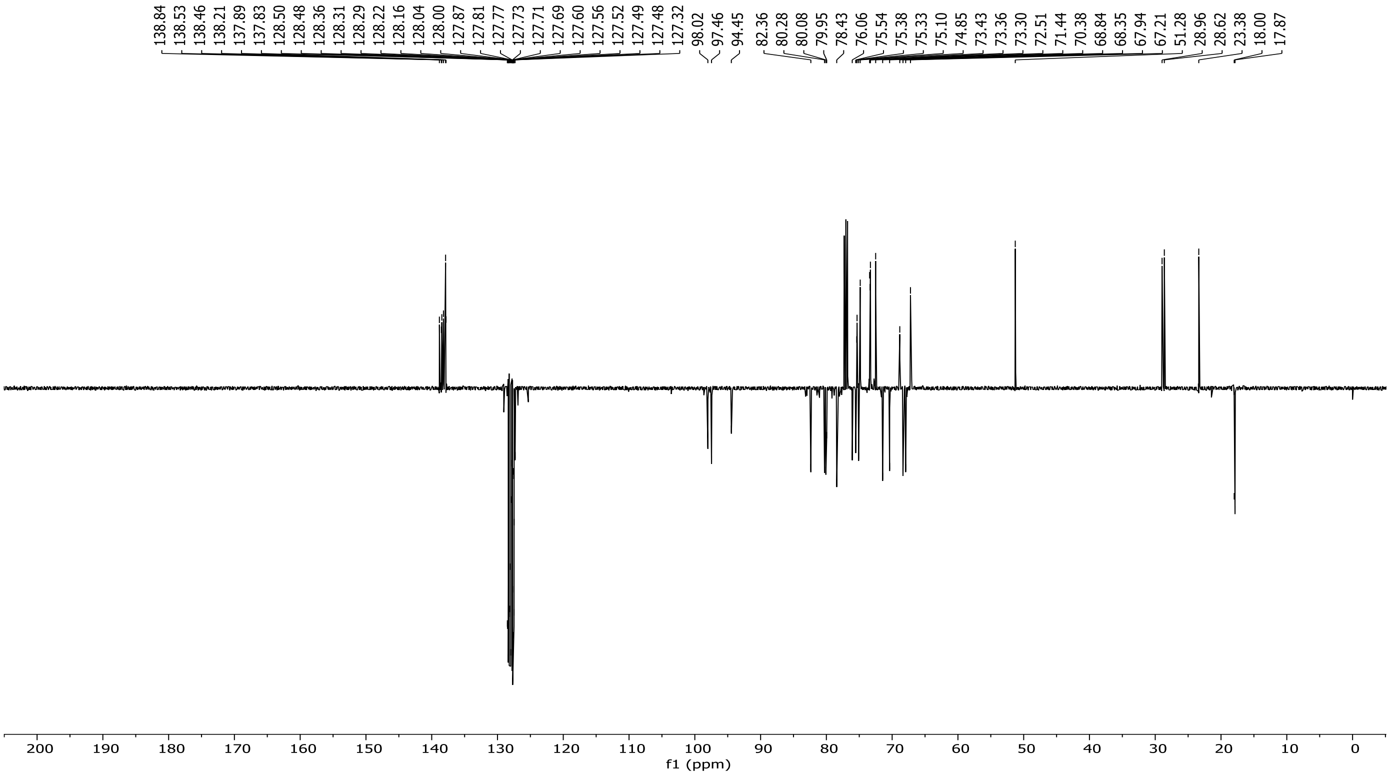

Supplementary Figure 75: ^{13}C NMR (126 MHz, $CDCl_3$) of compound **24**.

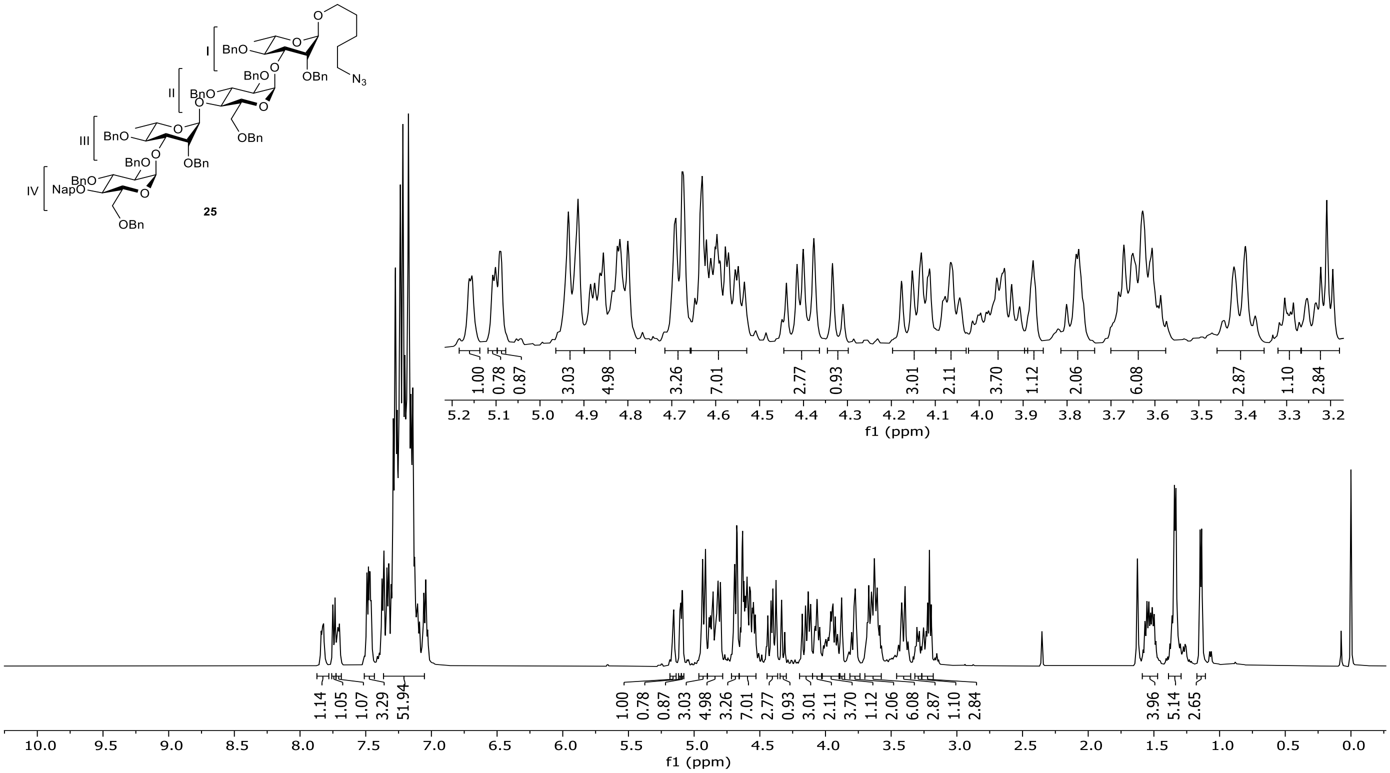

Supplementary Figure 76: ^{1}H NMR (500 MHz, $CDCl_3$) of compound **25**.

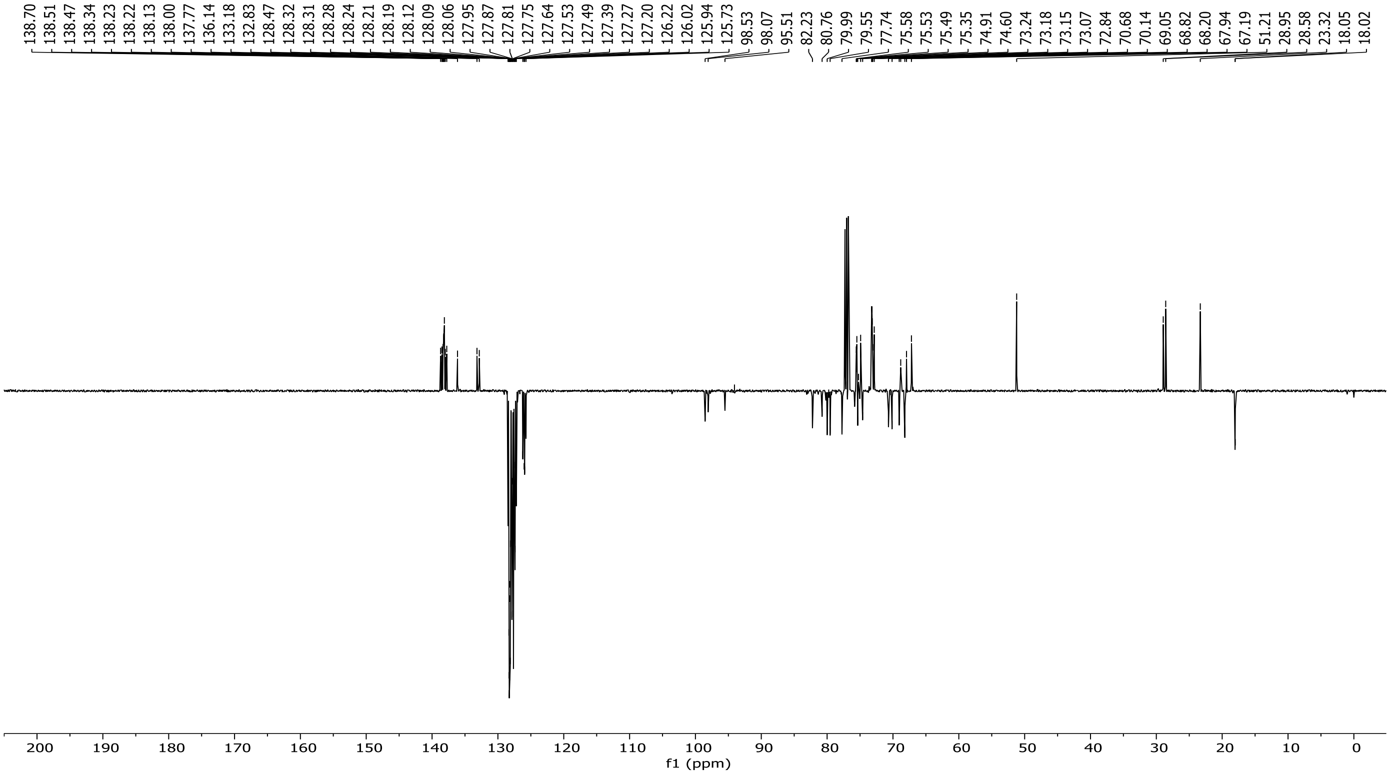

Supplementary Figure 77: ^{13}C NMR (126 MHz, $CDCl_3$) of compound **25**.

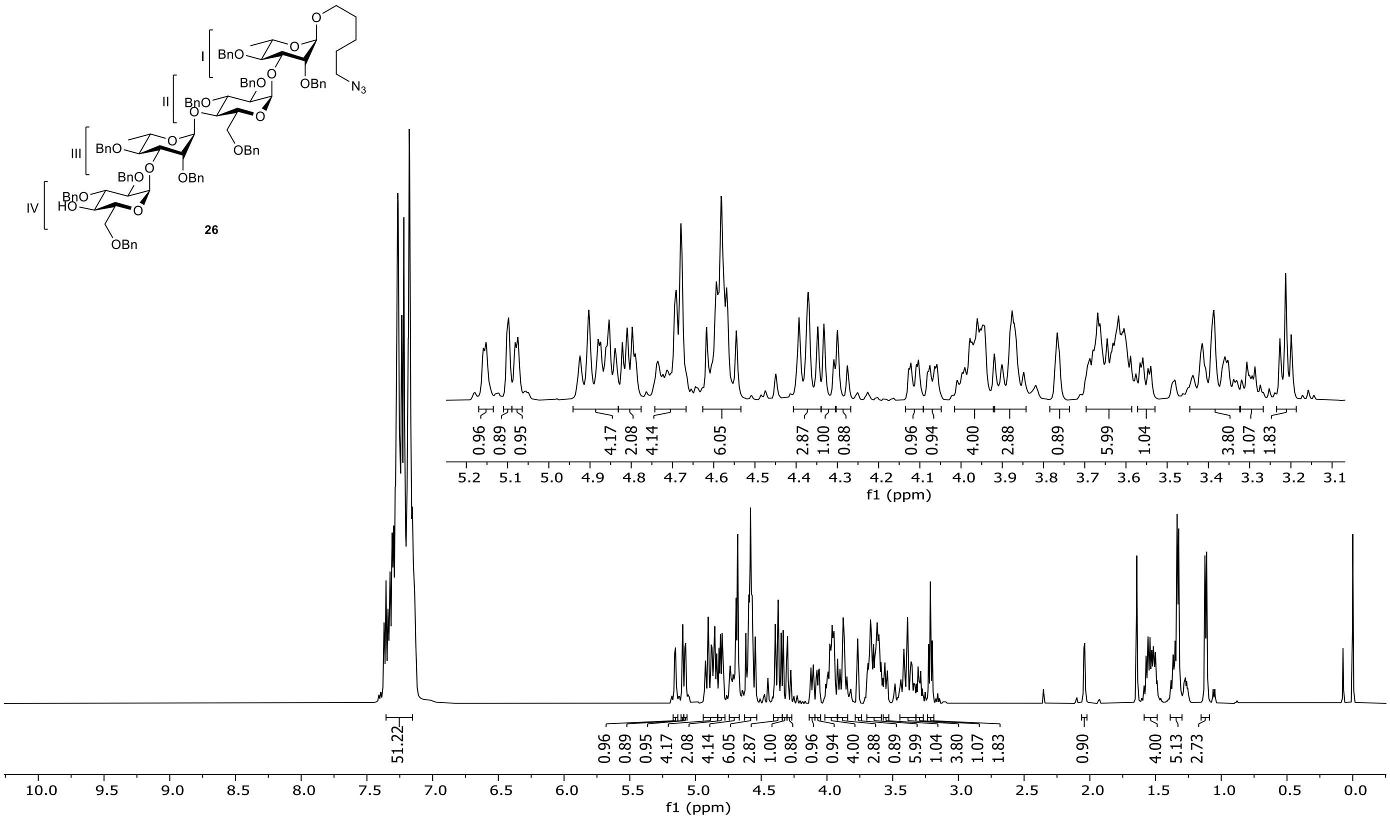

Supplementary Figure 78: ^{1}H NMR (500 MHz, $CDCl_3$) of compound **26**.

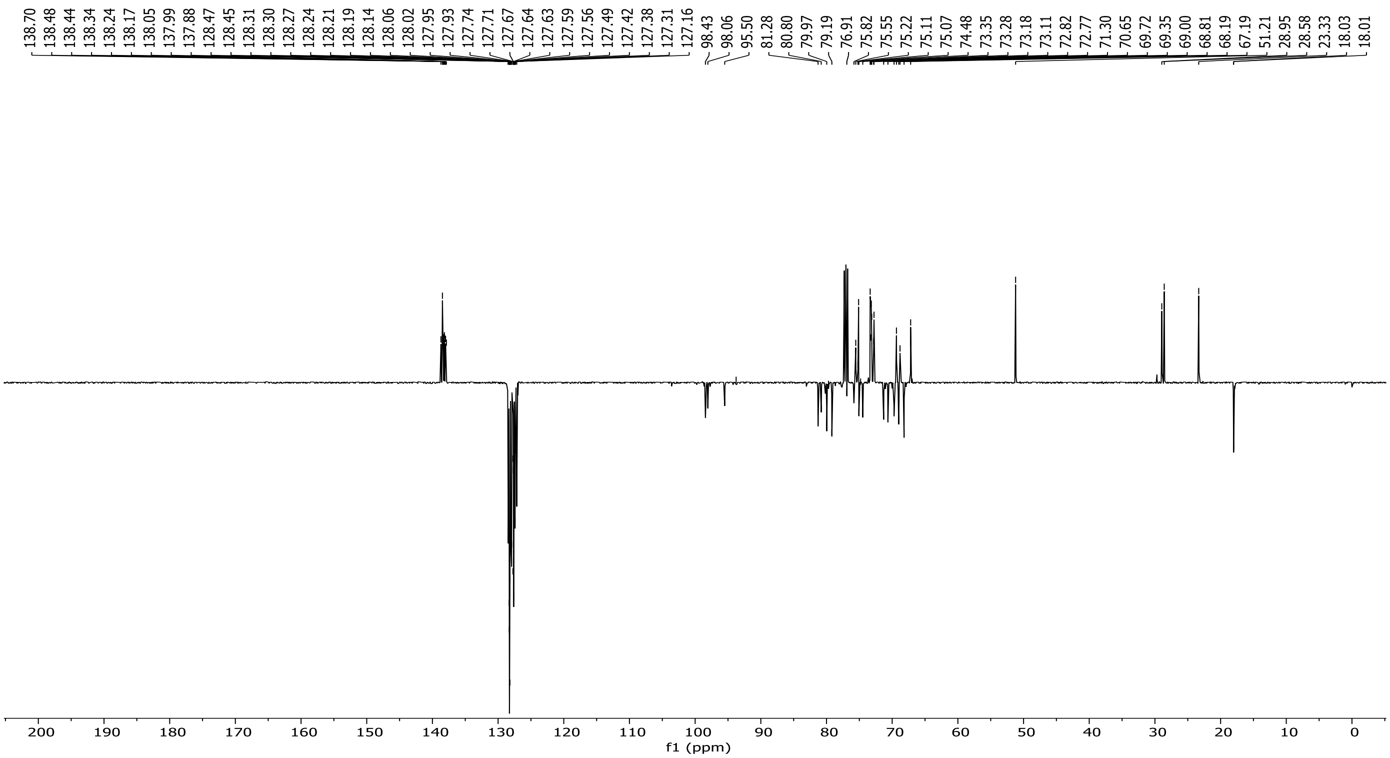

Supplementary Figure 79: ^{13}C NMR (126 MHz, $CDCl_3$) of compound **26**.

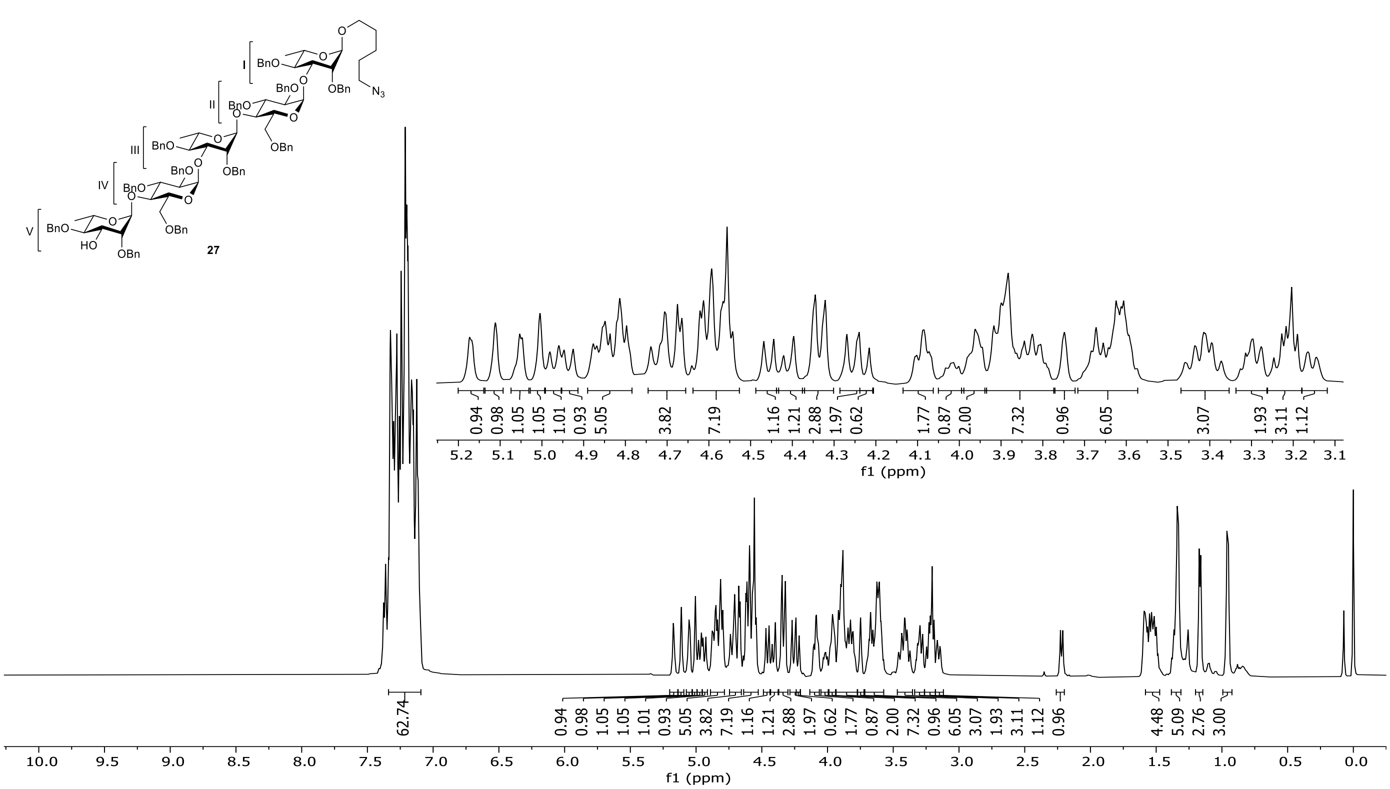

Supplementary Figure 80: ^{1}H NMR (500 MHz, $CDCl_3$) of compound **27**.

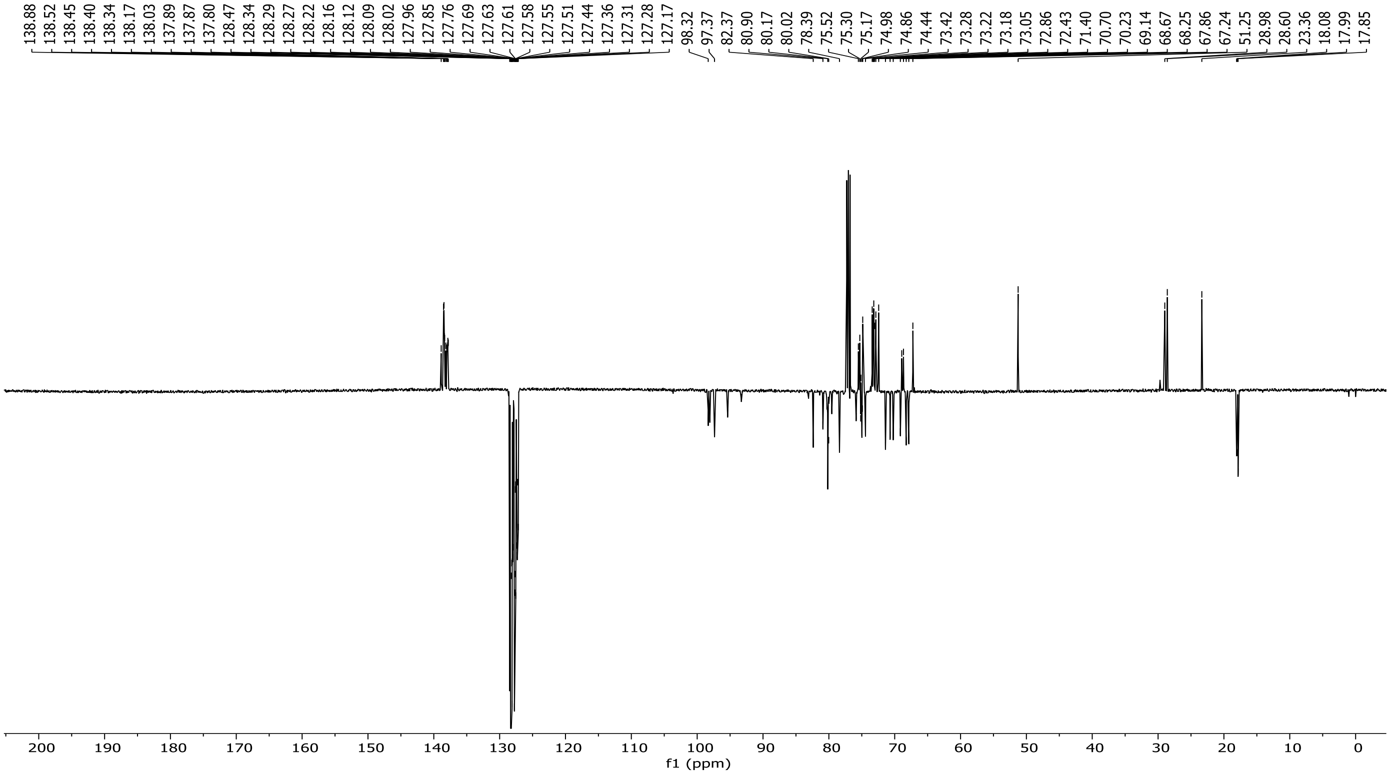

Supplementary Figure 81: ^{13}C NMR (126 MHz, $CDCl_3$) of compound **27**.

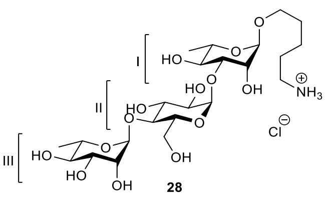

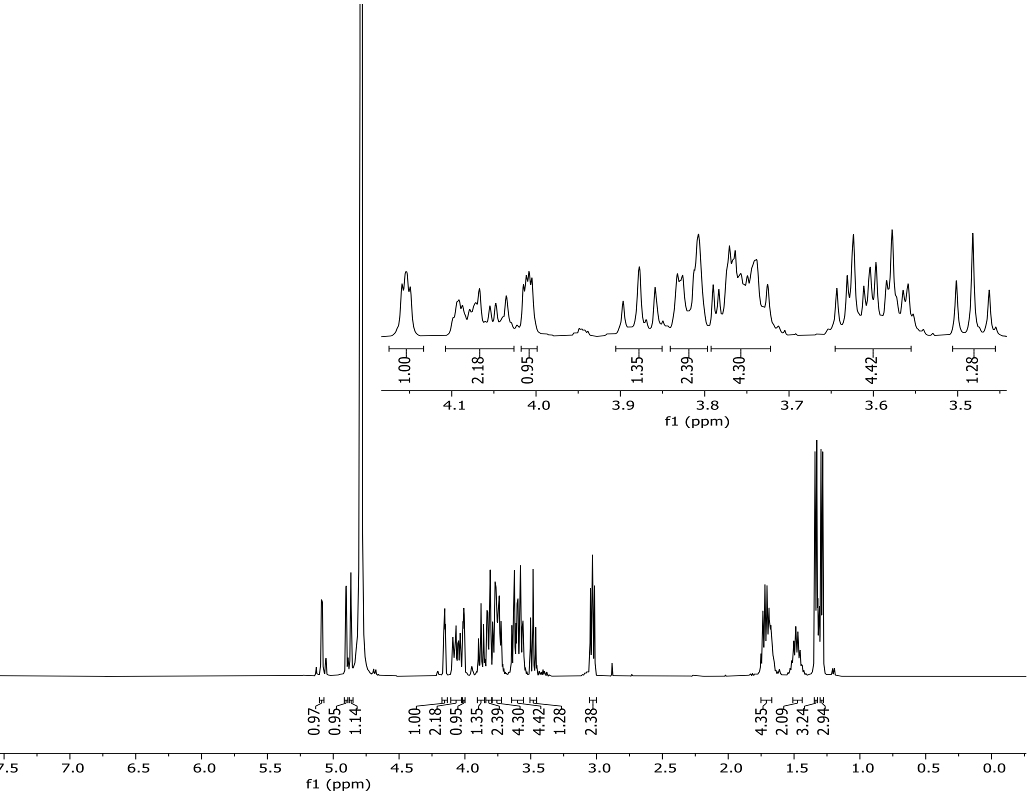

Supplementary Figure 82: ^{1}H NMR (500 MHz, D_2O) of compound **28**.

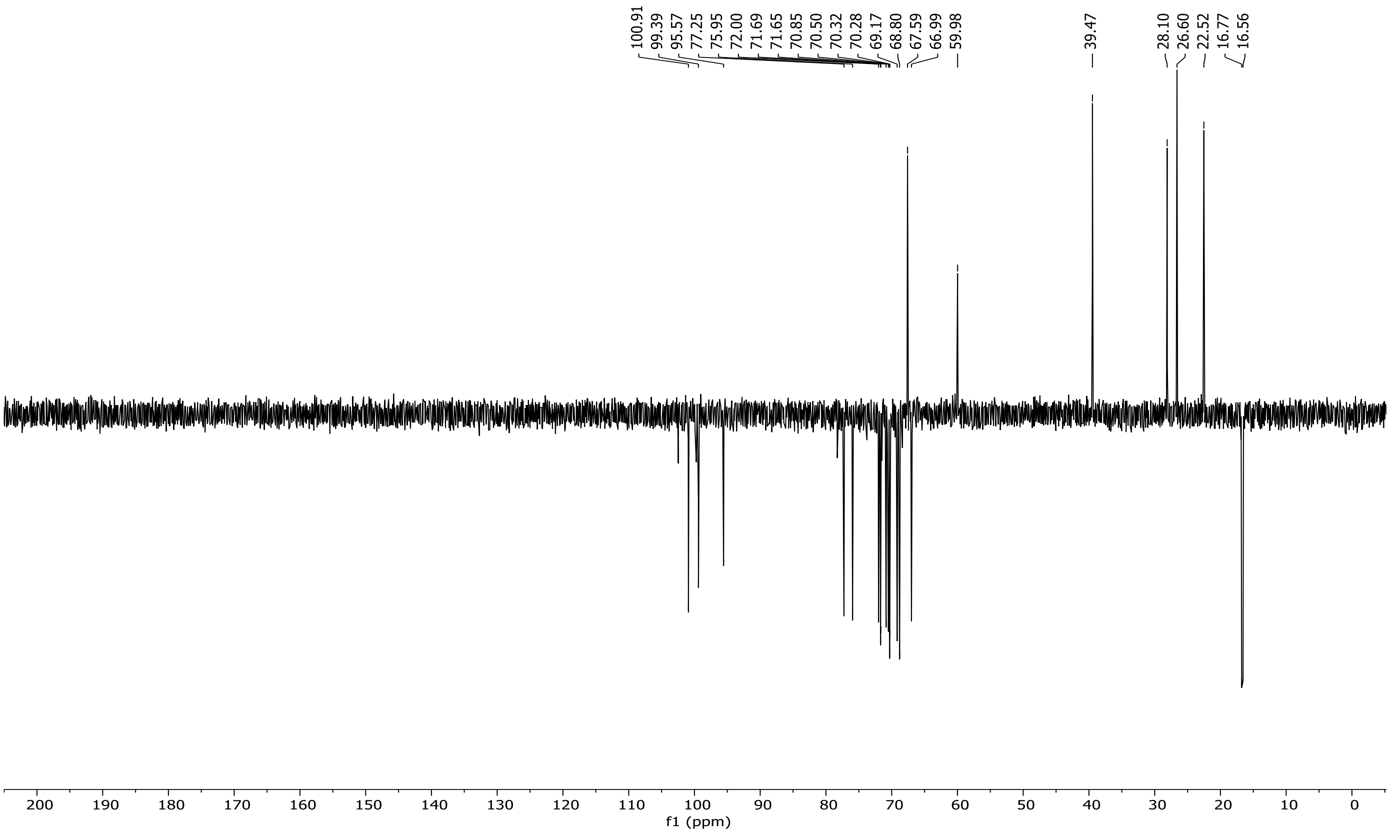

Supplementary Figure 83: ^{13}C NMR (126 MHz, D_2O) of compound **28**.

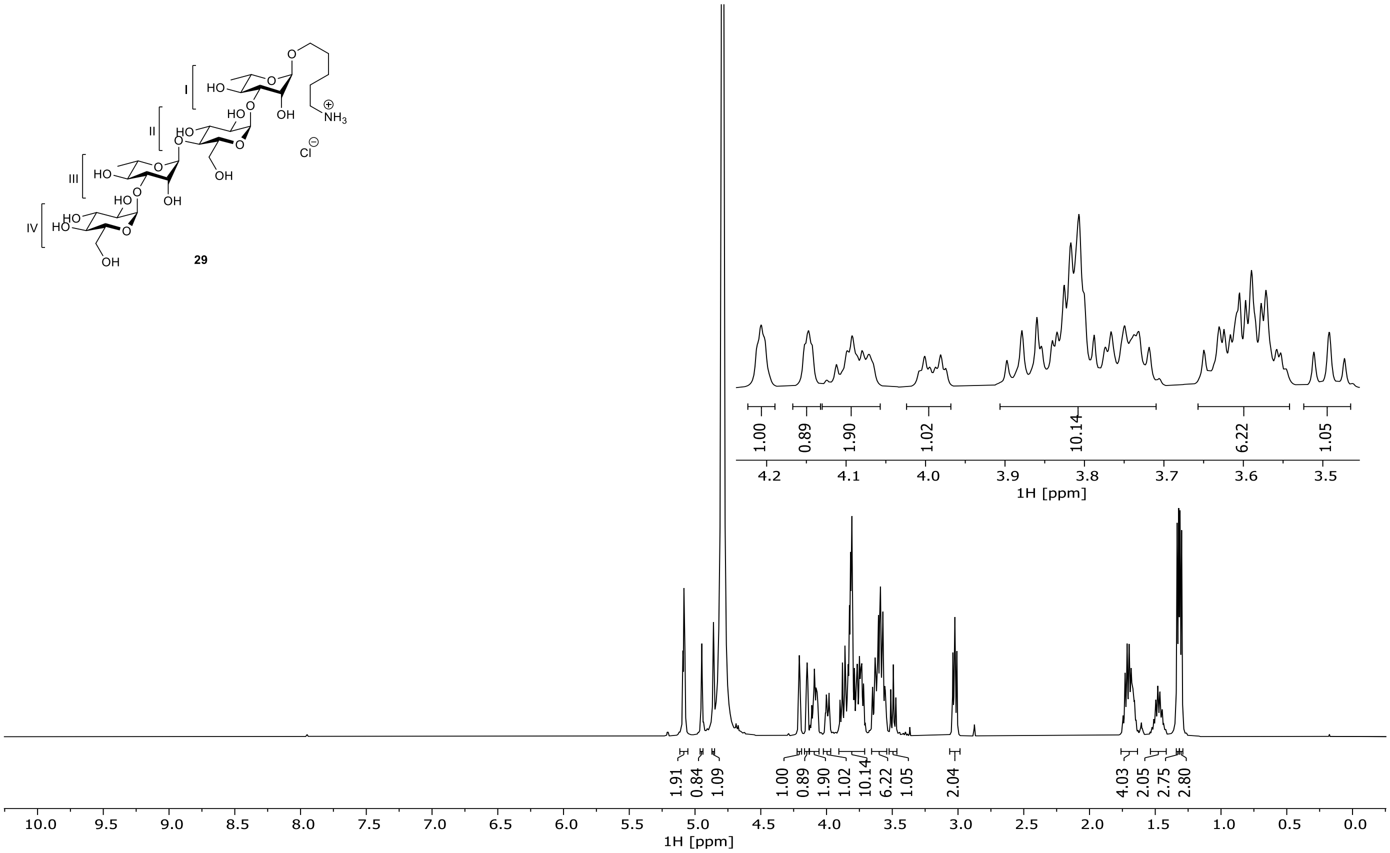

29

Supplementary Figure 84: ^{1}H NMR (500 MHz, D_2O) of compound **29**.

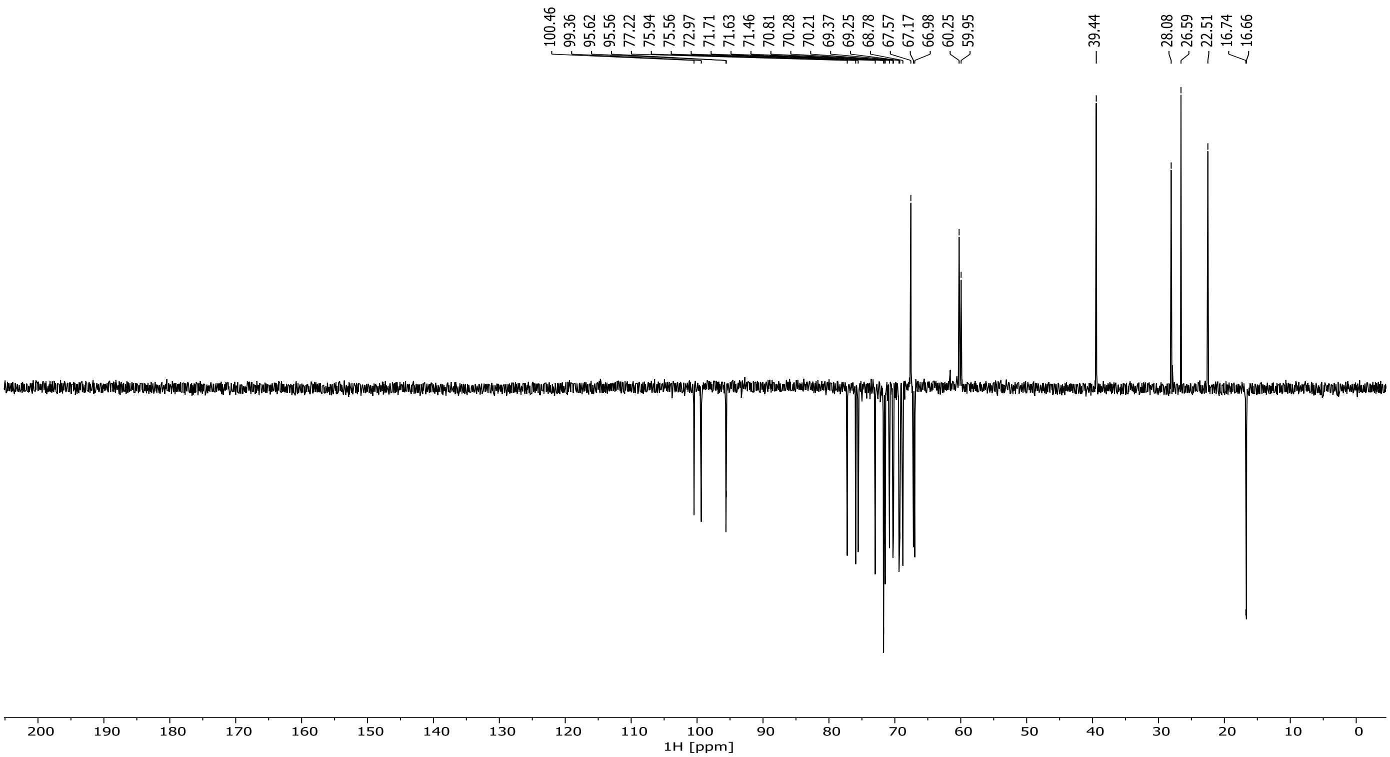

Supplementary Figure 85: ^{13}C NMR (126 MHz, D_2O) of compound **29**.

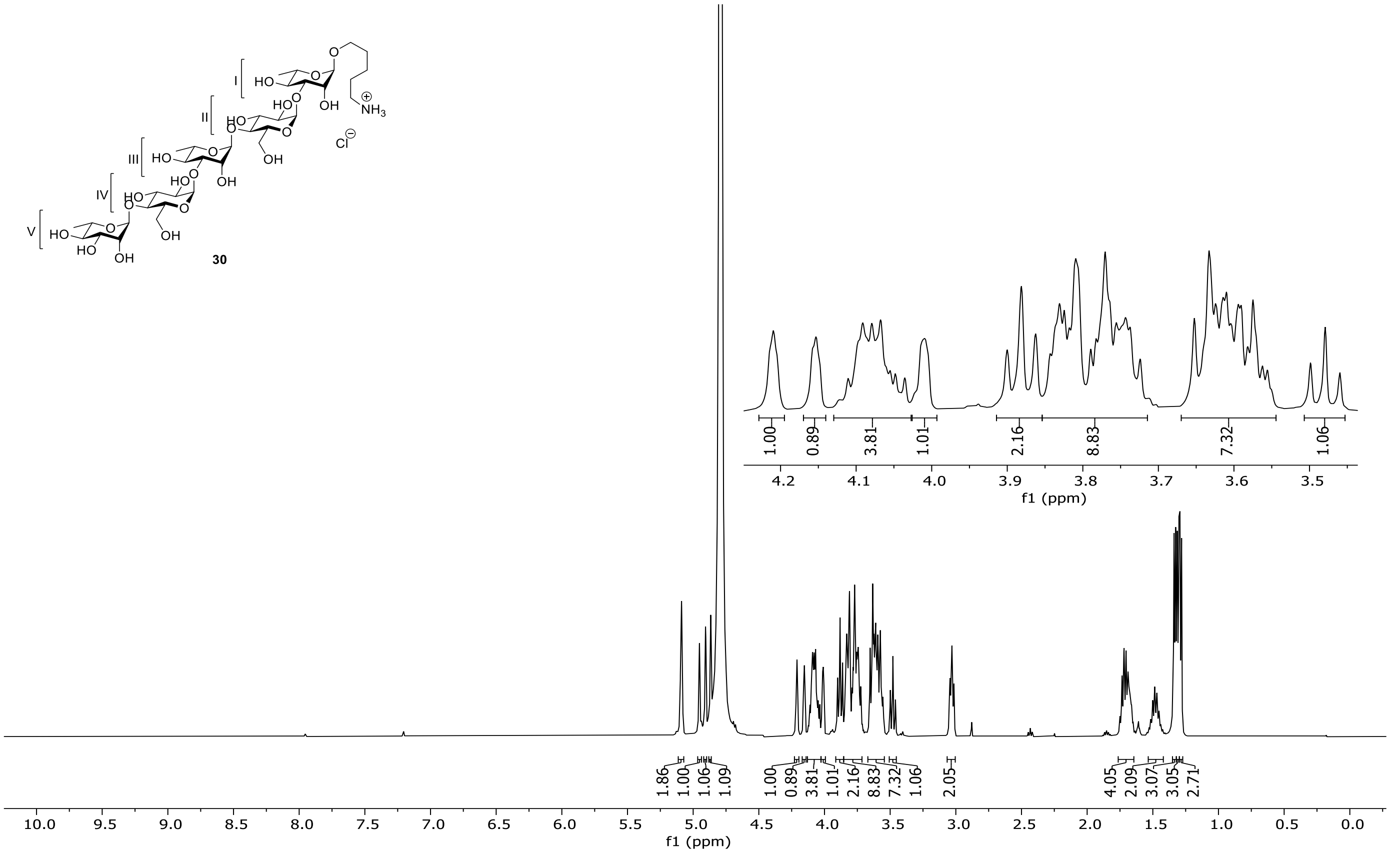

Supplementary Figure 86: ^{1}H NMR (500 MHz, D_2O) of compound **30**.

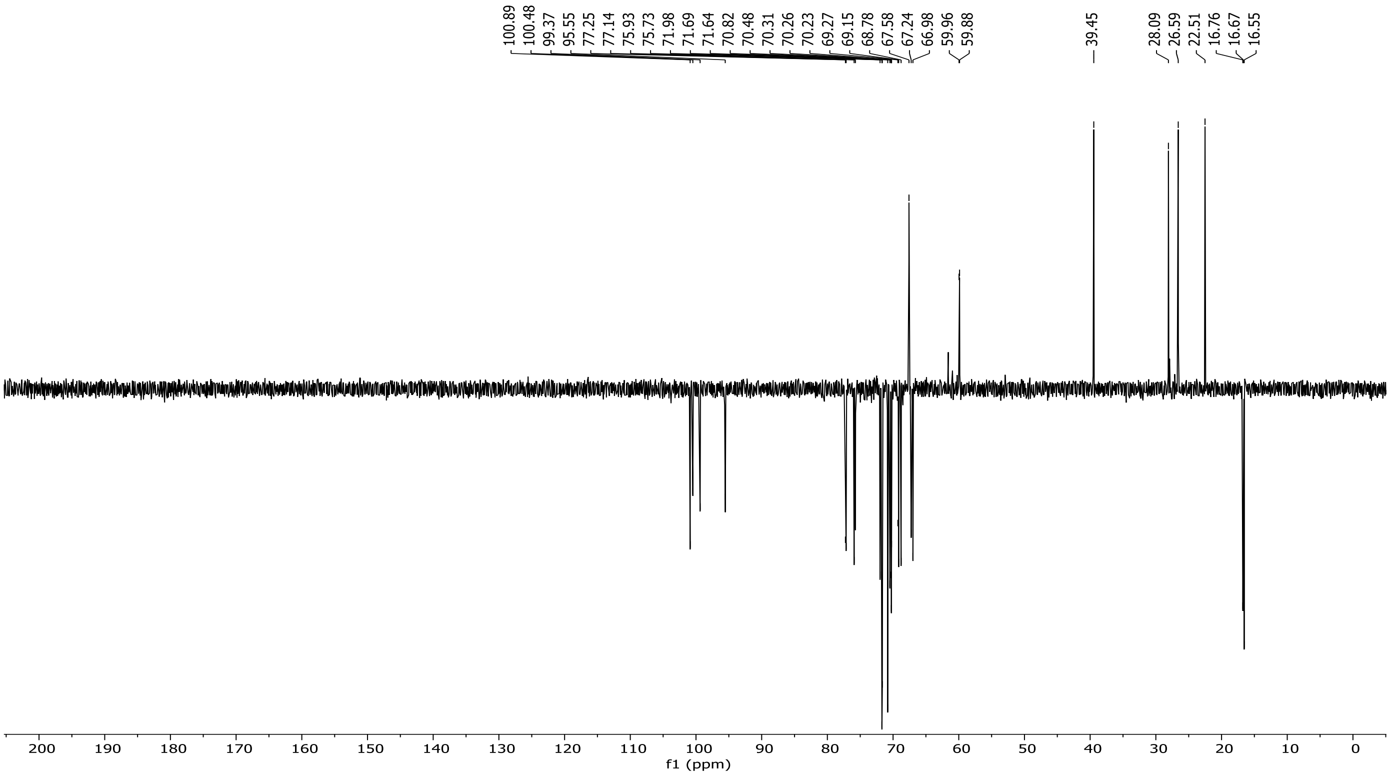

Supplementary Figure 87: ^{13}C NMR (126 MHz, D_2O) of compound **30**.

Supplementary References

[1] J. Martens, G. Berden, C.R. Gebhardt, J. Oomens, *Rev. Sci. Instrum.* **2016**, *87*;

[2] D. Oepts, A.F.G. van der Meer, P.W. van Amersfoort, *Infared Phys. Technol.* **1995**, *36*, 297-308.

[3] T. Hansen, H. Elferink, J.M. van Hengst, K.J. Houthuijs, W.A. Remmerswaal, A. Kromm, G. Berden, S. van der Vorm, A.M. Rijs, H.S. Overkleeft, D.V. Filippov, F.P.J.T. Rutjes, G.A. van der Marel, J. Martens, J. Oomens, J.D.C. Codée, T.J. Boltje *Nat. Commun.* **2020**, *11*, 2664.

[4] G. Landrum, RDKit: Open-source cheminformatics **2006**.

[5] Gaussian 16, Revision A.03, M.J. Frisch, G.W. Trucks, H.B. Schlegel, G.E. Scuseria, M.A. Robb, J.R. Cheeseman, G. Scalmani, V. Barone, G. A. Petersson, H. Nakatsuji, X. Li, M. Caricato, A.V. Marenich, J. Bloino, B.G. Janesko, R. Gomperts, B. Mennucci, H.P. Hratchian, J.V. Ortiz, A.F. Izmaylov, J.L. Sonnenberg, D. Williams-Young, F. Ding, F. Lipparini, F. Egidi, J. Goings, B. Peng, A. Petrone, T. Henderson, D. Ranasinghe, V.G. Zakrzewski, J. Gao, N. Rega, G. Zheng, W. Liang, M. Hada, M. Ehara, K. Toyota, R. Fukuda, J. Hasegawa, M. Ishida, T. Nakajima, Y. Honda, O. Kitao, H. Nakai, T. Vreven, K. Throssell, J.A. Montgomery, Jr., J.E. Peralta, F. Ogliaro, M.J. Bearpark, J.J. Heyd, E.N. Brothers, K.N. Kudin, V.N. Staroverov, T.A. Keith, R. Kobayashi, J. Normand, K. Raghavachari, A.P. Rendell, J.C. Burant, S.S. Iyengar, J. Tomasi, M. Cossi, J.M. Millam, M. Klene, C. Adamo, R. Cammi, J.W. Ochterski, R.L. Martin, K. Morokuma, O. Farkas, J.B. Foresman, and D.J. Fox, *Gaussian, Inc., Wallingford CT*, **2016**.

[6] I. Tvaroska, F.R. Taravel, *Advances in carbohydrate chemistry and biochemistry* **1995**, *51*, 15-61;

[7] W.A. Bubb, *Concepts in Magnetic Resonance Part A: An Educational Journal* **2003**, *19*, 1-19.

[8] S. Heikkinen, M.M. Toikka, P.T. Karhunen, I.A. Kilpeläinen, *J. Am. Chem. Soc.* **2003**, *125*, 4362-4367;

[9] H. Koskela, I. Kilpeläinen, S. Heikkinen, *J. Magn. Reson.* **2005**, *174*, 237-244.

[10] A.S. Serianni, J. Pierce, S.G. Huang, R. Barker *J. Am. Chem. Soc.* **1982***,* 104, 4037-4044.

[11] H.M. McConnell, *J. Chem. Phys.* **1958***,* 28, 430-431.

[12] F.F.J. de Kleijne, H. Elferink, S.J. Moons, P. B. White, T.J. Boltje, *Angew. Chem. Int. Ed.* **2022**, *61*, e202109874.

[13] K. Bock, C. Pedersen, *J. Chem. Soc., Perkin Trans. 2* **1974**, 293-297.

[14] F.F.J. de Kleijne, F. ter Braak, D. Piperoudis; P. Moons, S. Moons, H. Elferink, P.B. White, T.J. Boltje, *J. Am. Chem. Soc.* **2023** 145, 26190-26201.
